# Structure–Property Relationships to Guide the Selection of Fluorinated Ethers for Li–S Batteries

**DOI:** 10.1021/jacs.6c04480

**Published:** 2026-07-26

**Authors:** Mark Stockham, Neubi F. Xavier, Jana B. Fritzke, Samuel D.S. Fitch, Liam Furness, Vikram K. Bharti, Nikolay Zhelev, Qiong Cai, Clare P. Grey, Nuria Garcia-Araez

**Affiliations:** † School of Chemistry and Chemical Engineering, 7423 University of Southampton, Southampton SO17 1BJ, United Kingdom; ‡ School of Chemistry and Chemical Engineering, 3660 University of Surrey, Guildford GU2 7XH, United Kingdom; § Yusuf Hamied Department of Chemistry, 2152 University of Cambridge, Cambridge CB2 1EW, United Kingdom; ∥ The Faraday Institution, Harwell Campus, Didcot OX11 0RA, United Kingdom; ⊥ Chemistry of Interfaces, Department of Civil Environmental and Natural Resources Engineering, Luleå University of Technology, Luleå SE-97187, Sweden

## Abstract

A variety of fluorinated ethers have been used to improve key performance metrics in lithium–sulfur batteries, such as specific energy and cycle life. However, most previous articles employed only one fluorinated ether, and there is currently a lack of criteria or consensus about which fluorinated ether provides the largest performance improvement. This stems from a lack of fundamental understanding of the correlation between the chemical formula of the fluorinated ether and its effect on lithium–sulfur battery reactions. In this work, we systematically investigated nine fluorinated ethers and tested their effects in lithium–sulfur batteries representative of commercially relevant conditions. Electrochemical measurements were complemented by *ex situ* and *operando* NMR characterization of the lithium metal anode reactions, *ex situ* SEM and EDX imaging of cycled lithium and sulfur electrodes, and ab initio calculations and molecular dynamics simulations of the electrolytes, thus revealing the key motifs in the chemical structure of the fluorinated ethers that lead to performance improvements. The best-performing fluorinated ethers were those that interact with lithium ions with a strength similar to that of the other solvent(s) in the electrolyte, and as a result, fast solvent exchange reactions and high lithium-ion mobility are promoted. This benefited both the lithium and the sulfur electrode reactions, with the suppression of lithium dendrites, the formation of a more protective lithium SEI, and the promotion of polysulfide reactions that avoid the passivation of the carbon in the sulfur electrode. The best results were obtained with TFMP (1,1,2,2-tetrafluoro-3-methoxypropane), which had not been used before in lithium–sulfur batteries.

## Graphical abstract

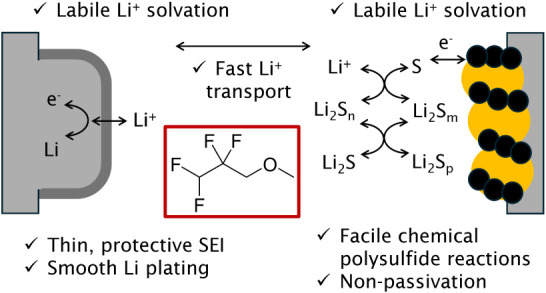

## 1 Introduction

Intensive research has been performed on Li–S batteries, particularly in the past decade, due to their promise to outperform the state-of-the-art Li-ion batteries in aspects such as high specific energy, low-temperature performance, prospective low cost, and the fact that they do not require the use of scarce metals such as nickel or cobalt.^1–5^ In addition, polysulfides, which are formed during the operation of Li–S batteries, have been shown to positively suppress the formation of dendrites or mossy structures on the lithium metal electrode, thus making Li–S batteries one of the most promising contenders for next-generation lithium metal anode batteries.^6,7^

The investigation of Li–S battery electrolytes has been identified as a key area of development to achieve performance improvements.^8–12^ Specifically, fluorinated ethers (also known as hydrofluoroethers) are receiving increasing attention as electrolyte cosolvents to boost Li–S battery performance, and a wide variety of fluorinated ethers have been investigated in recent years. Table S1 presents an overview of Li–S battery studies that combined fluorinated and nonfluorinated ethers, as in the present study, and Table S2 summarizes selected studies using fluorinated ethers as diluents in sparingly solvating electrolytes. However, most studies investigated only a single fluorinated ether, and the comparison between results from different investigations is hampered by the fact that the experimental conditions (e.g., sulfur electrode formulation, electrolyte-to-sulfur ratio, etc.) are different. Therefore, currently, there is a lack of understanding of how to select the best fluorinated ether electrolyte to achieve optimal performance.

A few previous studies investigated several fluorinated ethers and compared their effects on Li–S battery performance. Amine and coworkers showed that fluorinated ethers with more fluoroalkyl groups closer to the ether oxygen had the lowest lithium solvating power and the highest ability to mitigate polysulfide dissolution, but they also markedly decreased the electrolyte conductivity.^13^ Zhang and coworkers also reported that increasing the degree of fluorination in the fluorinated ethers contributed to improve the coulombic efficiency of Li–S cells due to the suppression of the polysulfide shuttle,^14^ and Mandal and coworkers showed that substitution of a −CF_2_CF_2_H end group with a −CF_3_ group decreased the electrolyte viscosity and thus enhanced the conductivity.^15^ On the other hand, fluorinated ethers with fluorination distant from the ether oxygen were found to improve the lithium metal anode electrochemistry due to the promotion of an anion-rich solvation around the lithium ions, which suppressed side reactions.^16,17^

In summary, previous work concluded that fluorination close to the ether oxygen of the solvent molecule is beneficial for suppressing polysulfide dissolution, whereas fluorination far away from the ether oxygen is beneficial for the lithium metal anode electrochemistry. Clearly, a balance between these two effects is needed. More recently, Cui and coworkers have shown that fluorinated ethers with selective fluorination and moderate polysulfide solubility lead to optimal performance of Li–S cells with a single solvent electrolyte.^18^ Kim and coworkers developed electrolytes with moderate polysulfide solubility by systematically mixing fluorinated and nonfluorinated ethers, leading to Li–S batteries with impressive performance.^19^

In this work, we investigate a total of nine fluorinated ethers, shown in Figure 1, as electrolyte cosolvents in Li–S batteries representative of commercial applications (i.e., built with sulfur electrodes with high sulfur content and sulfur loading, and lean electrolyte conditions). The fluorinated ethers were selected to include the most used fluorinated ether from the Li–S battery literature (TTE, see Figure 1 and Tables S1-S2) as well as others with reported high capacity (BTFE, ETFE, TFEE, and FDMB, see Table S1), and additional compounds with variations in the chain length, position, degree of fluorination, and symmetry/asymmetry of the molecular structure. *Ex situ* NMR (nuclear magnetic resonance) and SEM (scanning electron microscopy) with EDX (energy-dispersive X-ray spectroscopy) characterization of the cycled electrodes, and *operando* NMR of the lithium metal anode reactions, provided evidence of how the fluorinated ethers affected the variations in the electrode compositions and morphologies induced during cycling, thus shedding light on the processes leading to enhanced capacity retention. Ab initio calculations and molecular dynamics simulations were performed to study the effects of the fluorinated ethers on lithium-ion solvation and to quantify the lithium-ion transference number, where the latter was found to be the key driver affecting the rank of Li–S battery performance. Overall, this work provides an unprecedented understanding of the relationship between the chemical structure of the fluorinated ethers and their effect on Li–S battery performance, and, to the best of our knowledge, the best-performing fluorinated ether (TFMP, see Figure 1) has not been used in Li–S batteries before.

**Figure 1:**
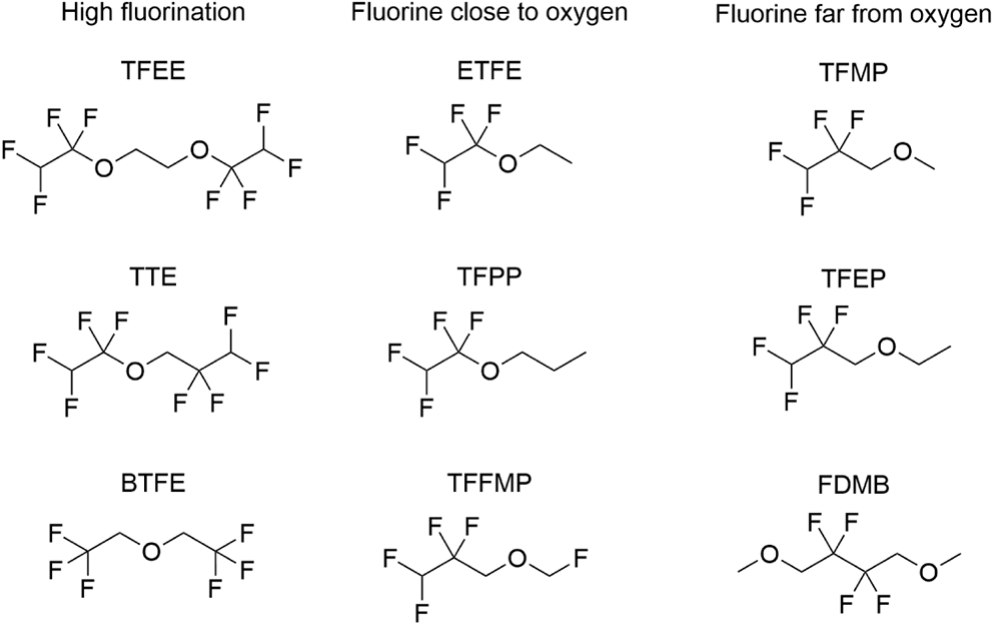
Chemical formulas of the fluorinated ethers employed in this work (see their names in the list of abbreviations).

## 2 Results and Discussion

### 2.1 Battery Performance

The introduction of fluorinated ethers in Li–S batteries can lead, with some formulations, to significant performance improvements. For example, Figure 2 shows an electrolyte formulation with TFMP (green data; 1 M LiTFSI + 0.25 M LiNO_3_ in DOL:DME:TFMP with a solvent ratio of 0:3:2) that enhances the battery longevity without compromising the capacity and also improves coulombic efficiency, as compared to the baseline electrolyte (open black symbols), where the latter is the electrolyte most often used in Li–S batteries (1 M LiTFSI + 0.25 M LiNO_3_ in DOL:DME 1:1). As shown in Figure S1, TFMP leads to an enhanced voltage profile stability during long-term cycling, which can be ascribed to a more balanced coordination of lithium ions in the electrolyte, as discussed in Section 2.2.

**Figure 2:**
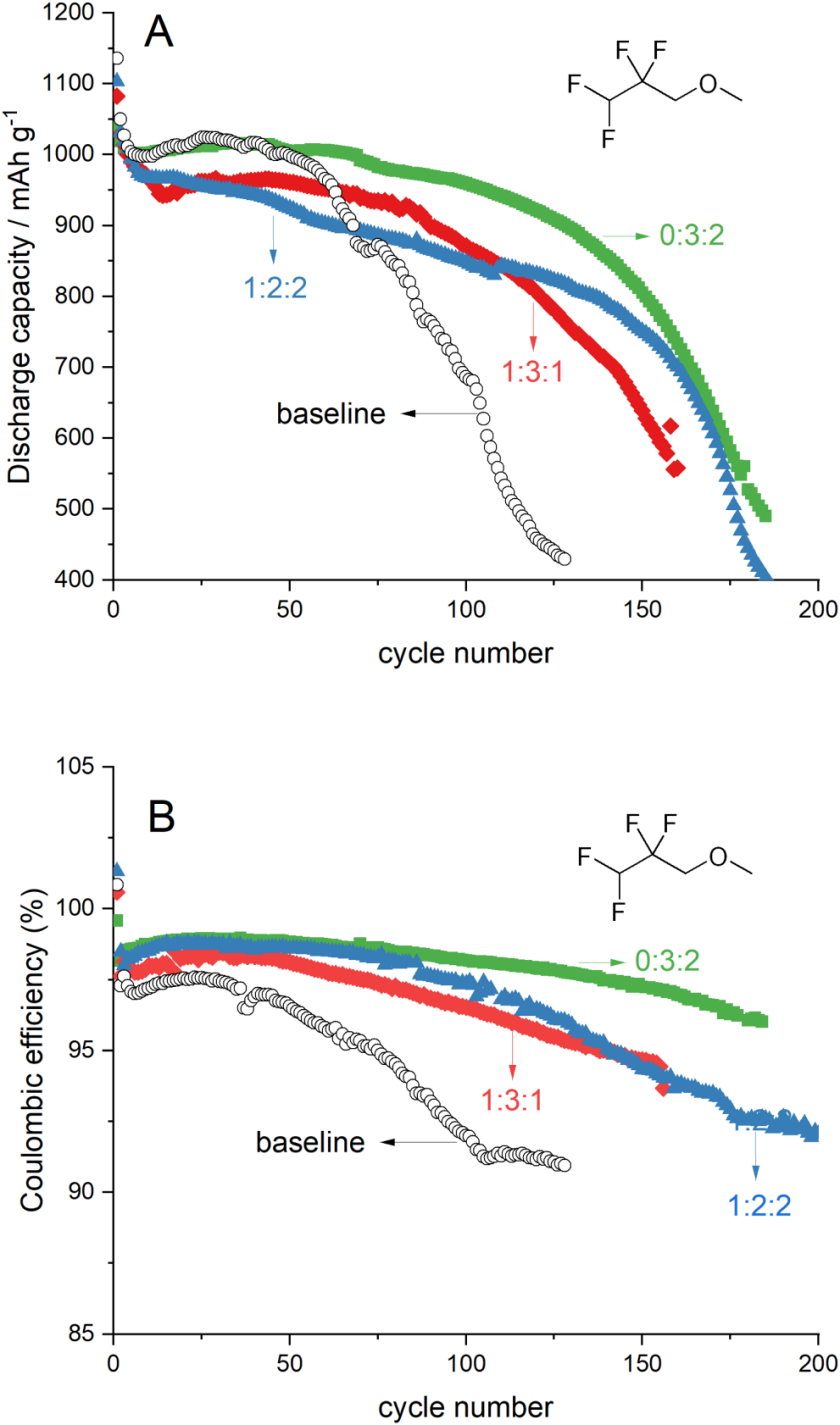
Improvement of Li–S cell longevity (A) and efficiency (B) via the incorporation of the fluorinated ether TFMP, whose chemical structure is shown in the figure. Results were obtained with 1 M LiTFSI + 0.25 M LiNO_3_ in DOL:DME:TFMP with solvent ratios of 0:3:2 (green), 1:3:1 (red), and 1:2:2 (blue), and with the baseline electrolyte 1 M LiTFSI + 0.25 M LiNO_3_ in DOL:DME 1:1 (black open circles), with C/5 cycling between 1.8 and 2.6 V.

However, when used in other solvent ratios, TFMP can lead to compromised performance improvements, as shown in Figure 2, with improvements in longevity and coulombic efficiency but with compromises in capacity (blue and red data) compared to the baseline. These results show that the substitution of DOL by TFMP (from red to green data) or by DME (from blue to green data) results in capacity improvements, whereas substitution of DME by TFMP (from red to blue data) produces minor changes in capacity (although the coulombic efficiency is improved). These trends reveal the critical role of both DME and TFMP to achieve high capacity, longevity, and efficiency, and indeed, the lower DME content of a simpler 1:1 formulation (or the absence of DME in a 1:1 formulation with DOL and TFMP) leads to inferior performance, as shown in Figure S2.

A critical area for improvement for the commercial realization of Li–S batteries is their power capability,^20^ which is often made even worse by the addition of fluorinated ethers. Unfortunately, many studies lack the comparison of rate capability testing with a benchmark electrolyte (see Table S3). Fortunately, as shown in Figure 3, the incorporation of TFMP in the electrolyte produces significant rate capability improvements with respect to the baseline electrolyte, since the enhancement in capacity in the presence of TFMP increases as the C-rate increases. A moderate content of the fluorinated ether in the electrolyte (20%, red data) produced a better rate capability than a higher fluorinated ether content (40%, green data), but both outperformed the baseline electrolyte due to an enhanced utilization of the low-voltage plateau, as shown in Figure S3.

**Figure 3:**
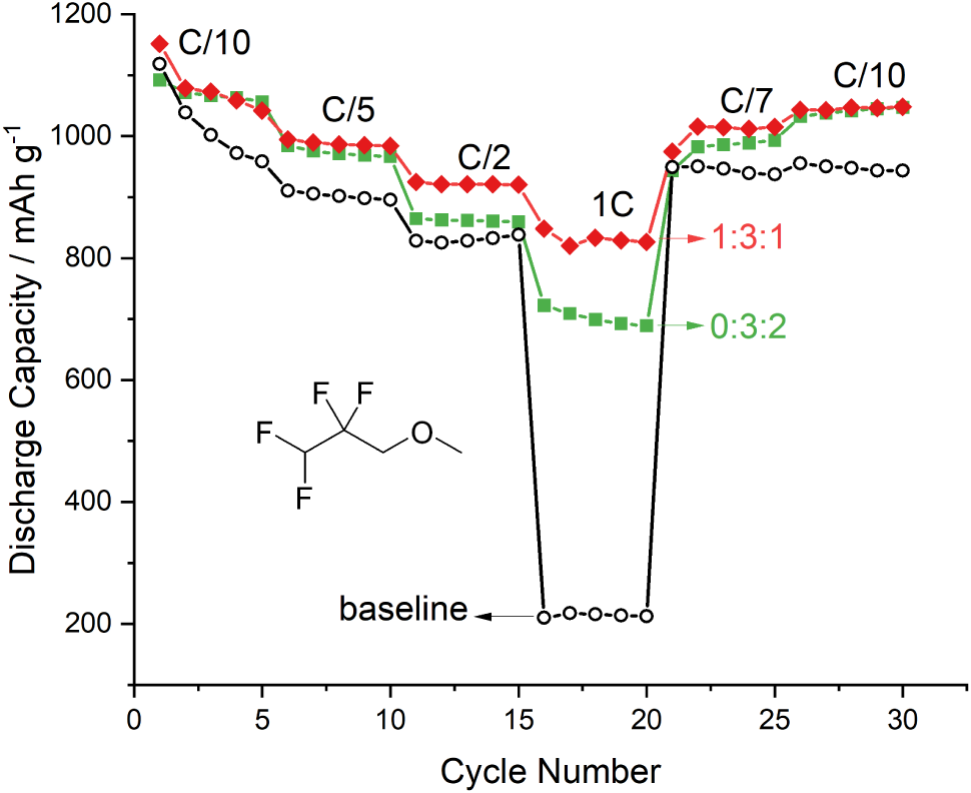
Improvement of Li–S cell rate capability using a TFMP fluorinated ether. The experimental conditions are as shown in Figure 2, except that the C-rate was varied every five cycles, as indicated in the figure.

Although the results in Figures 2 and 3 clearly show that TFMP is an advantageous cosolvent for Li–S batteries, the question of whether there are better fluorinated ethers remains. To address this question, the rate capability of Li–S batteries with five selected fluorinated ethers was evaluated. A moderate fluorinated ether content (20%) was employed, since it produced superior rate capability results. Figure 4 shows that all studied fluorinated ethers surpass the rate capability of the baseline electrolyte, although the best performance was achieved with fluorinated ethers such as TFMP (red) and TFEP (orange) that had no fluorination on the carbon next to the oxygen ether.

**Figure 4:**
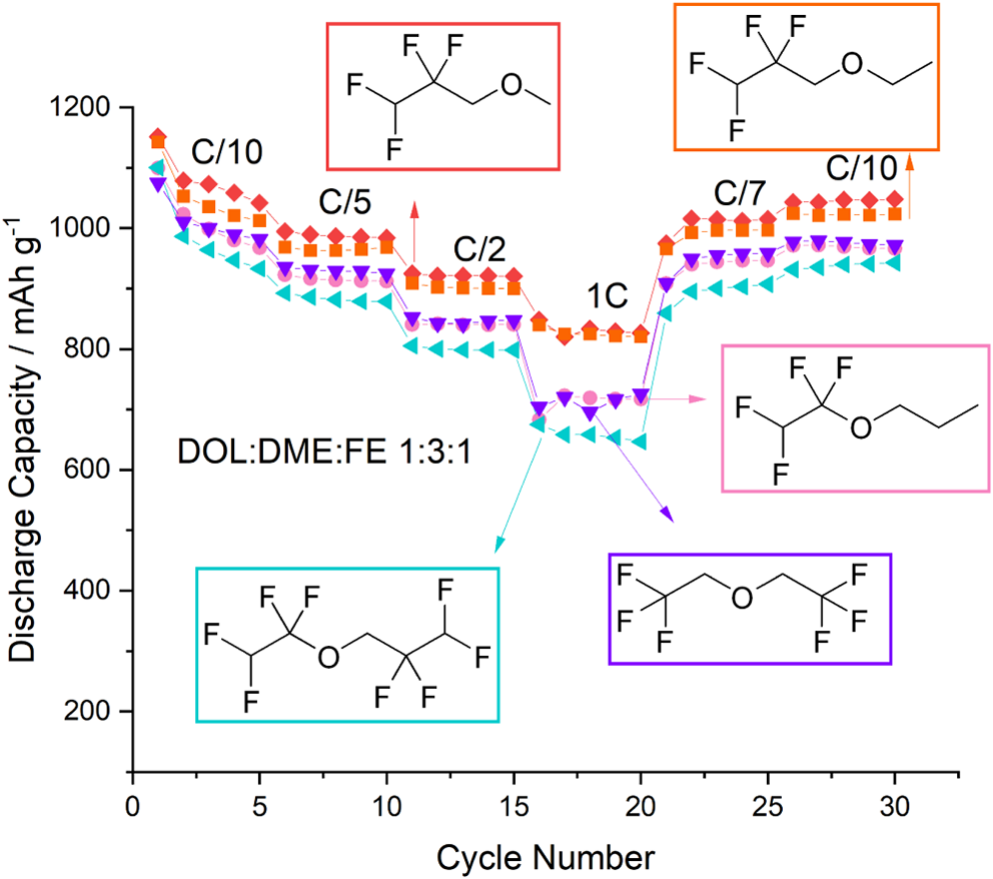
The effect of the fluorinated ether chemical formula on the rate capability of Li–S batteries. All conditions are as in Figure 3, but with electrolytes made using fluorinated ethers with chemical structures shown in the figure: TFMP (red), TFEP (orange), TFPP (pink), BTFE (purple), and TTE (light blue), using a solvent ratio of DOL:DME:FE equal to 1:3:1, where FE is the fluorinated ether.

The long-term cycling stability of Li–S batteries, in the presence of a moderate content of fluorinated ether (20%), was also studied, showing that most fluorinated ethers produce similar results (Figure S4), albeit shorter battery lifetimes were obtained with fluorinated ethers that have fluorination on the carbon next to the oxygen ether (Figure S5). As discussed in Section 2.2, when the fluorinated ethers are present in low or moderate concentrations, their effect on the electrolyte properties is only minor, since fluorinated ethers exhibit weak intermolecular interactions, and consequently, most fluorinated ether solvents produce similar performance (Figure S4). However, when the fluorinated ethers have fluorination close to the oxygen ether, their noninteracting nature is exacerbated, which has detrimental effects on the electrolyte properties (see Section 2.2) and thus results in poorer battery performance (Figure S5).

On the other hand, the use of a high content of the fluorinated ether in the electrolyte is advantageous because it enables improved performance, as shown in Figure 2, where the best-performing formulation had 40% of fluorinated ether. As shown in Figures 5 and 6, at high concentrations, the chemical formula of the fluorinated ether produces a very significant effect on battery performance. Specifically, the best-performing fluorinated ethers are those that have no fluorination in the carbon next to the oxygen ether, such as TFMP (red) and TFEP (orange), consistent with the rate capability trends shown in Figure 4. In the next section, we employ ab initio calculations of bonding strengths and molecular dynamic simulations of the electrolytes’ properties to provide an understanding of how and why the differences in the chemical formula of the fluorinated ethers affect the desired Li–S battery reactions.

**Figure 5:**
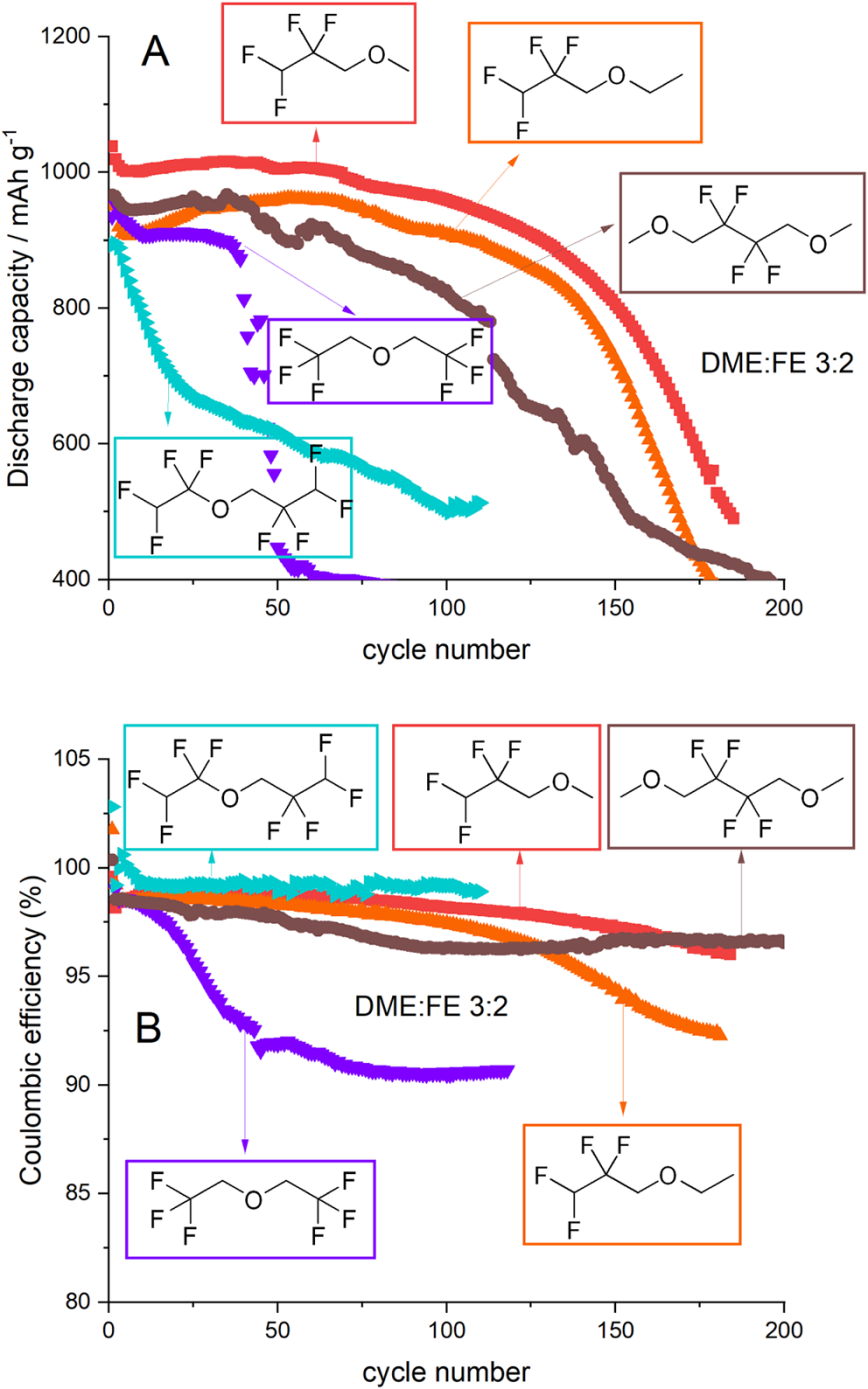
Effect of the fluorinated ether chemical formula on the long-term cycling of Li–S batteries. All conditions are the same as in Figure 2, but with TFMP (red), TFEP (orange), FDMB (brown), BTFE (purple), and TTE (light blue), using a solvent ratio of DME:FE equal to 3:2, where FE is the fluorinated ether.

**Figure 6:**
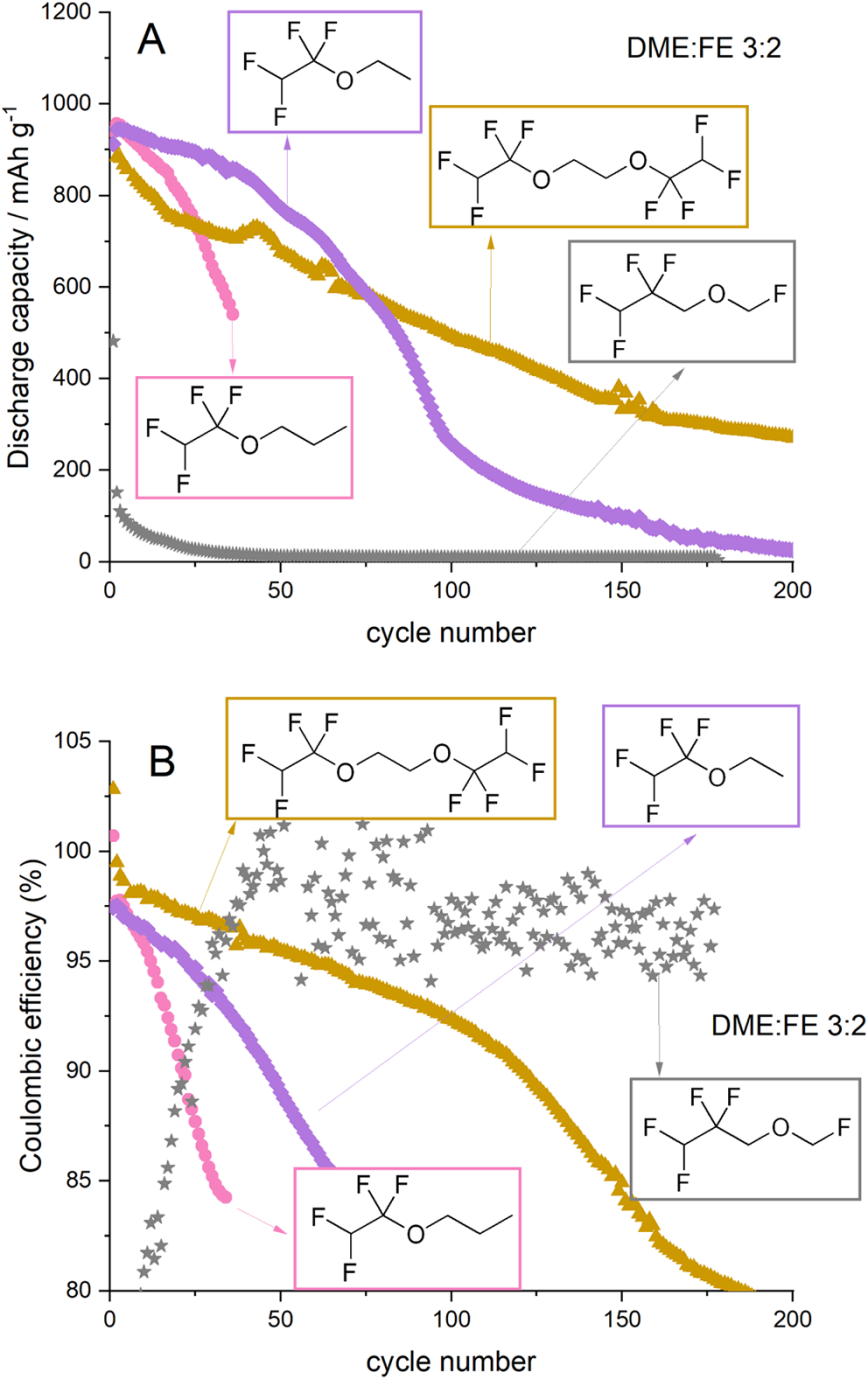
As in Figure 5, but with the fluorinated ethers TFPP (pink), ETFE (lilac), TFEE (gold), and TFFMP (gray).

### 2.2 Critical Electrolyte Properties

Ab initio simulations were performed to evaluate the binding energy of the different fluorinated ethers when coordinated with either lithium ions or with a lithium polysulfide (specifically, Li_2_S_4_, as it was found to be a key intermediate in polysulfide redox reactions^21^). Figure 7A,B shows that fluorinated ethers with no fluorination in the carbon next to the oxygen ether (known as the “alpha” carbon) exhibit the highest binding energies, whereas fluorinated ethers with fluorination in the alpha carbon exhibit lower binding energies, regardless of their degree of fluorination. The high number of fluorinated ethers studied here, unprecedented in the literature, enables us to demonstrate that the fluorination location (Figure 7C) has a much stronger influence on the binding energies than the degree of fluorination (Figure 7D). Of note, TFFMP has a single fluorination in the alpha carbon, but that still results in low binding energies. A special case is BTFE, which has no fluorination in the alpha carbon but a very high degree of fluorination, and Figure 7A,B shows that it exhibits low binding energies.

**Figure 7:**
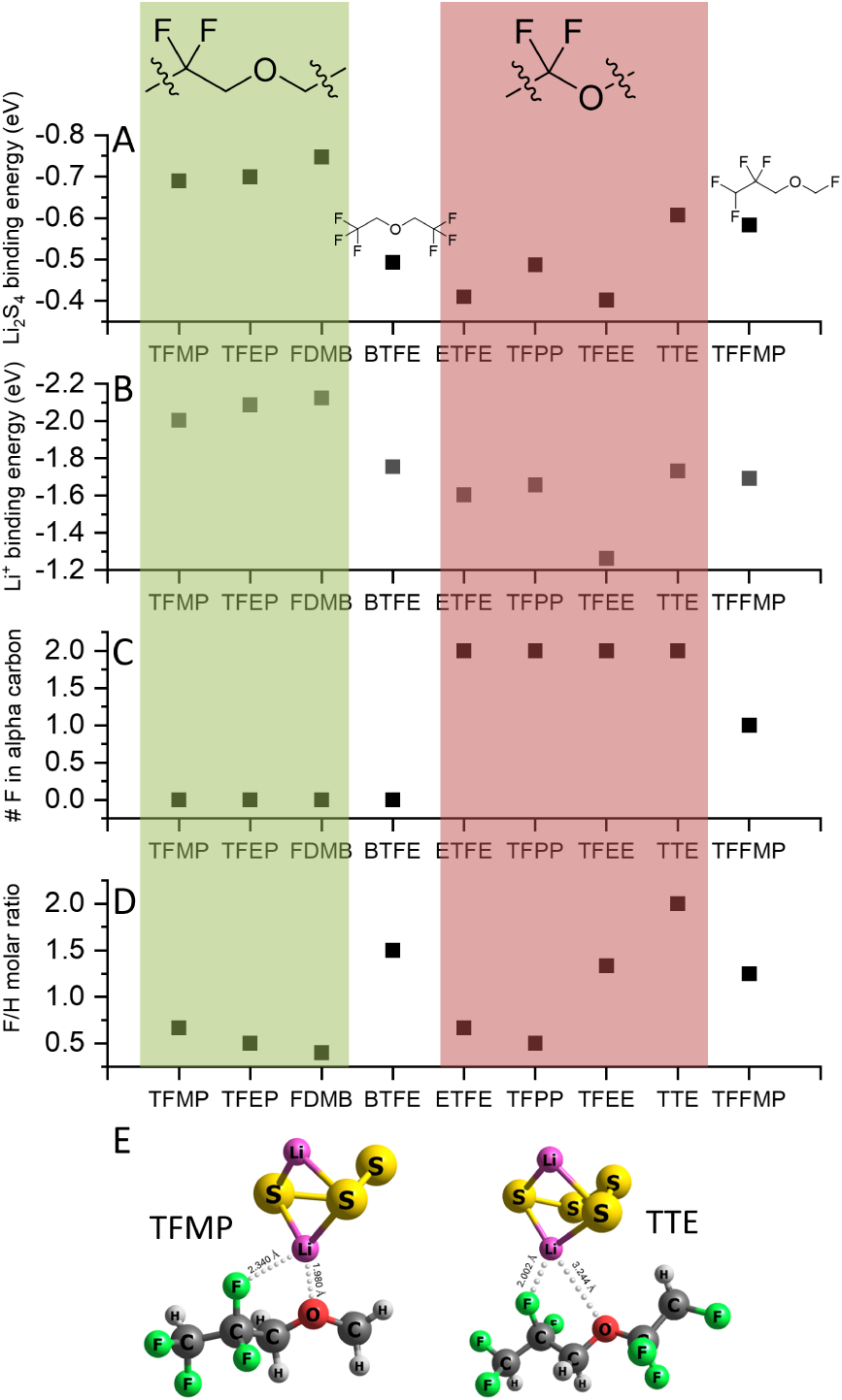
Influence of the chemical structure of fluorinated ethers on the binding energies of Li^+^ ions (A) or Li_2_S_4_ (B), as computed via ab initio simulations in optimized coordination structures, as illustrated using TFMP and TTE in (E). The number of fluorine atoms in the alptha carbon (C) and the hydrogen-to-fluorine molar ratio (D) of the fluorinated ethers are also shown.

The key role of the binding energies in affecting battery performance was elucidated via molecular dynamics simulations. The electrolytes with a high concentration of fluorinated ethers (40%, Figure 8A) exhibit a higher lithium transference number when the fluorinated ether has no fluorination on the alpha carbon. In contrast, the electrolytes with a moderate concentration of the fluorinated ether (20%, Figure 8B) exhibit smaller values of the lithium transference number, even for the fluorinated ethers that have no fluorination on the alpha carbon, with the only exception of TFMP, which is the best-performing fluorinated ether found here. Although these differences in transference numbers are moderate, they reveal a mechanism to enhance lithium-ion mobility with important consequences for battery performance (see further discussion in Section S4). Of note, whereas FDMB exhibits the highest lithium binding energy, the lithium transference numbers of FDMB-containing electrolytes are not as high as those of TFMP-containing electrolytes, which we attribute to the fact that the symmetry of the chemical formula of FDMB leads to more rigid interactions and, thus, lower lithium-ion mobility.

**Figure 8:**
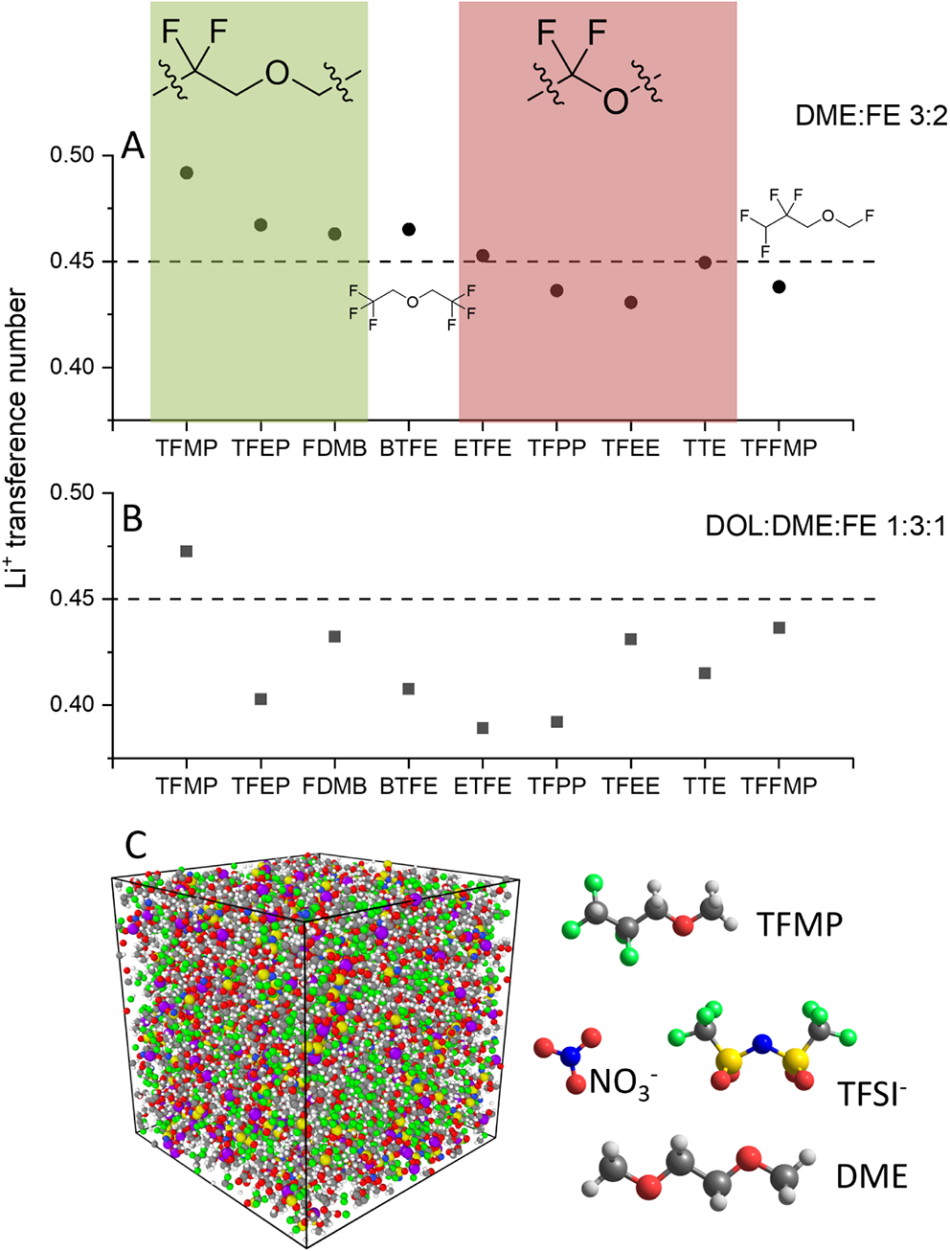
The influence of the chemical structure of fluorinated ethers on the lithium transference number, as obtained from molecular dynamic simulations of 1 M LiTFSI + 0.25 M LiNO_3_ in DOL:DME:FE with solvent ratios of 0:3:2 (A) and 1:3:1 (B), where FE stands for fluorinated ether. A snapshot of molecular dynamic simulations of the best-performing fluorinated ether electrolyte formulation (with a DME:TFMP 3:2 solvent mixture) is shown in (C).

The high lithium transference number is achieved by fluorinated ethers with strong bonding to lithium ions because, in that way, the pathways for lithium-ion hopping between different coordination environments exhibit lower activation energy, as the bonding strength of lithium ions to the different solvents in the electrolyte is better balanced. Since nonfluorinated ethers, such as DME, bind lithium metal ions strongly, the fluorinated ether cosolvents need to bind lithium ions sufficiently strongly so that the reactions involving the exchange of solvents coordinating the lithium ion are fast (Figure 9), thus leading to a labile and dynamic lithium-ion solvation. Because of the weak intermolecular interactions of the fluorinated ether, the lithium ions solvated by fluorinated ethers can exhibit fast vehicular motion, thus contributing to the acceleration of lithium-ion transport.

**Figure 9:**
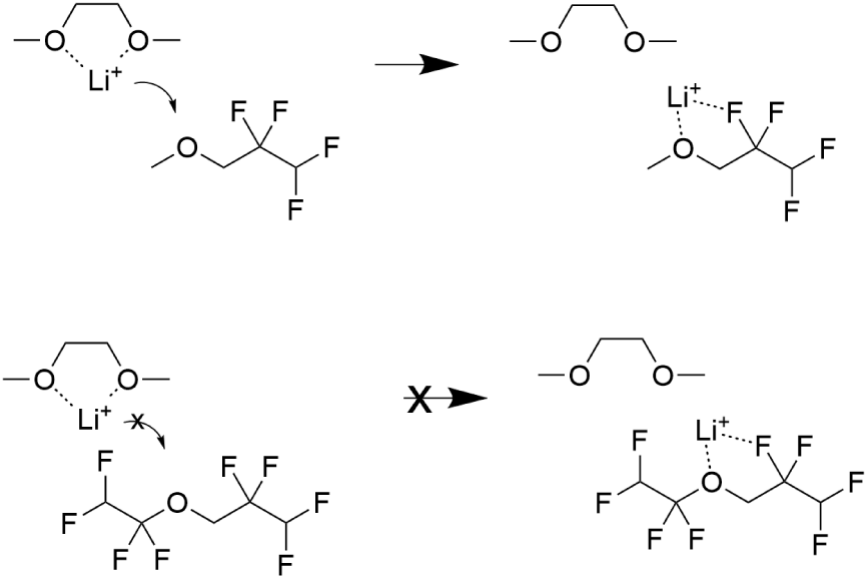
Solvent-exchange reactions of lithium ions coordinated to nonfluorinated and fluorinated ethers, where the latter have either high (upper panel) or low (lower panel) binding to lithium ions. TFMP and TTE are used as examples of fluorinated ethers with high or low binding to lithium ions, respectively, and DME is used as the nonfluorinated ether. The crosses on the arrows in the lower panel indicate unfavorable/sluggish reactions.

However, high lithium-ion mobility is, per se, not sufficient to lead to improved battery performance. The results of the molecular dynamic simulations show that the absolute value of the lithium-ion diffusion coefficient (Figure S6) does not show a good correlation with the Li–S battery performance trends. This is in agreement with the fact that the fluorinated ether with the lowest boiling point (ETFE, 56–58 °C),^22^ and thus the lowest viscosity (0.49 mPa s),^22^ does not lead to the highest performance improvements, as would be expected if the fluorinated ether solvents were only acting as diluents. In addition, the calculations of Sand’s time and limiting current densities also suggest that the present experiments are not limited by long-range diffusion (see details in Section S5).^23,24^ On the other hand, the distance between the fluorinated ether and lithium ions or Li_2_S_4_ (Figure S7) does not show a strong correlation with the observed trends in battery performance either, so we conclude that the lithium transference number (Figure 8), which critically depends on the rate of lithium-ion solvent exchange reactions (Figure 9), is the most important bulk electrolyte property affecting battery performance.

We propose that the fast lithium-ion solvent exchange reactions, enabled by fluorinated ethers with strong bonding to lithium ions, are at the core of the observed improved performance because they intrinsically promote more spatially homogeneous electrode reactions. Indeed, previous *operando* ^7^Li NMR experiments revealed that lithium microstructures (e.g., mossy and dendritic lithium) form at currents much lower than the limiting current at which long-range diffusion limitations trigger dendrite formation, and they were tentatively attributed to local reaction heterogeneity.^25^ Later, ^6,7^Li NMR isotope exchange measurements further demonstrated that fast lithium-ion transport through the lithium SEI is key to achieving good performance, as it minimizes reaction inhomogeneities.^26,27^ Here, we propose that fast lithium-ion solvent exchange reactions, accelerated by fluorinated ethers, promote high lithium-ion mobility through the SEI and, consequently, flatter lithium deposits.

Previous work identified the depletion of polysulfide species near the sulfur electrode as the main factor limiting the capacities achievable by Li–S batteries, since high polysulfide mobility enables polysulfide chemical reactions that lead to Li_2_S formation without surface passivation of the sulfur electrode, such as^28^

1 $$Li_{2}S_{n}+Li_{2}S_{m}\to Li_{2}S+Li_{2}S_{n+m-1}$$

However, recent work has shown that polysulfides are strongly bonded to lithium ions,^29^ and therefore, here we propose that a high mobility of lithium ions in the electrolyte is a prerequisite for achieving high polysulfide mobility. In addition, labile and dynamic lithium-ion solvation, enabled by the fluorinated ether-promoted solvent exchange reactions (Figure 9), is also expected to promote the kinetics of the polysulfide chemical reactions, since the latter involves the transfer of lithium ions between polysulfides (Figure S8).

Importantly, the critical effect of the similarity in the bonding strengths of lithium ions with all the solvents in the electrolyte, deduced from the present ab initio calculations and molecular dynamics simulations, is likely to apply to any electrolyte formulation. Indeed, for weakly solvating acetonitrile-based electrolytes, improved Li–S battery performance was observed with electrolytes containing more weakly coordinating fluorinated ethers (e.g., TTE) compared to electrolytes with stronger coordinating fluorinated ethers (e.g., BTFE).^30^ The following section discusses how these critical electrolyte properties facilitate Li–S battery reactions.

### 2.3 Probing the Individual Electrode’s Reactions

The influence of fluorinated ethers on lithium metal anode reactions was studied via *ex situ* and *operando* NMR measurements, using electrolyte formulations with three selected fluorinated ethers: TFMP (the best-performing fluorinated ether, with no fluorination in the alpha carbon), TTE (the most-used fluorinated ether in the Li–S literature, with fluorination in the alpha carbon), and TFPP (with a similar chemical formula as TFMP but with fluorination in the alpha carbon; see Figure 1).

For the study of the effect of the fluorinated ether on the composition of the lithium metal SEI, *ex situ* solid-state magic angle spinning (MAS) NMR measurements were performed on lithium electrodeposits formed by first plating lithium metal directly onto a copper current collector and then transferring the deposits to an inert support to avoid measurement interferences, such as eddy currents caused by MAS. A high sensitivity toward the detection of SEI components is achieved by the use of thin lithium electrodeposits (with an average bulk thickness of ca. 5 μm). Interestingly, the^19^F NMR spectra of the lithium electrodeposits, formed in fluorinated ether electrolytes and in the baseline electrolyte, are all very similar (Figure 10A) and reveal the presence of only residual LiTFSI and LiF, thus evidencing that the fluorinated ethers do not undergo degradation reactions that lead to new fluorinated SEI decomposition products. In contrast, previous studies detected the formation of fluorocarbon compounds from the degradation of fluorinated ethers, but only after more aggressive cycling conditions in Li-NMC cells.^31^

**Figure 10:**
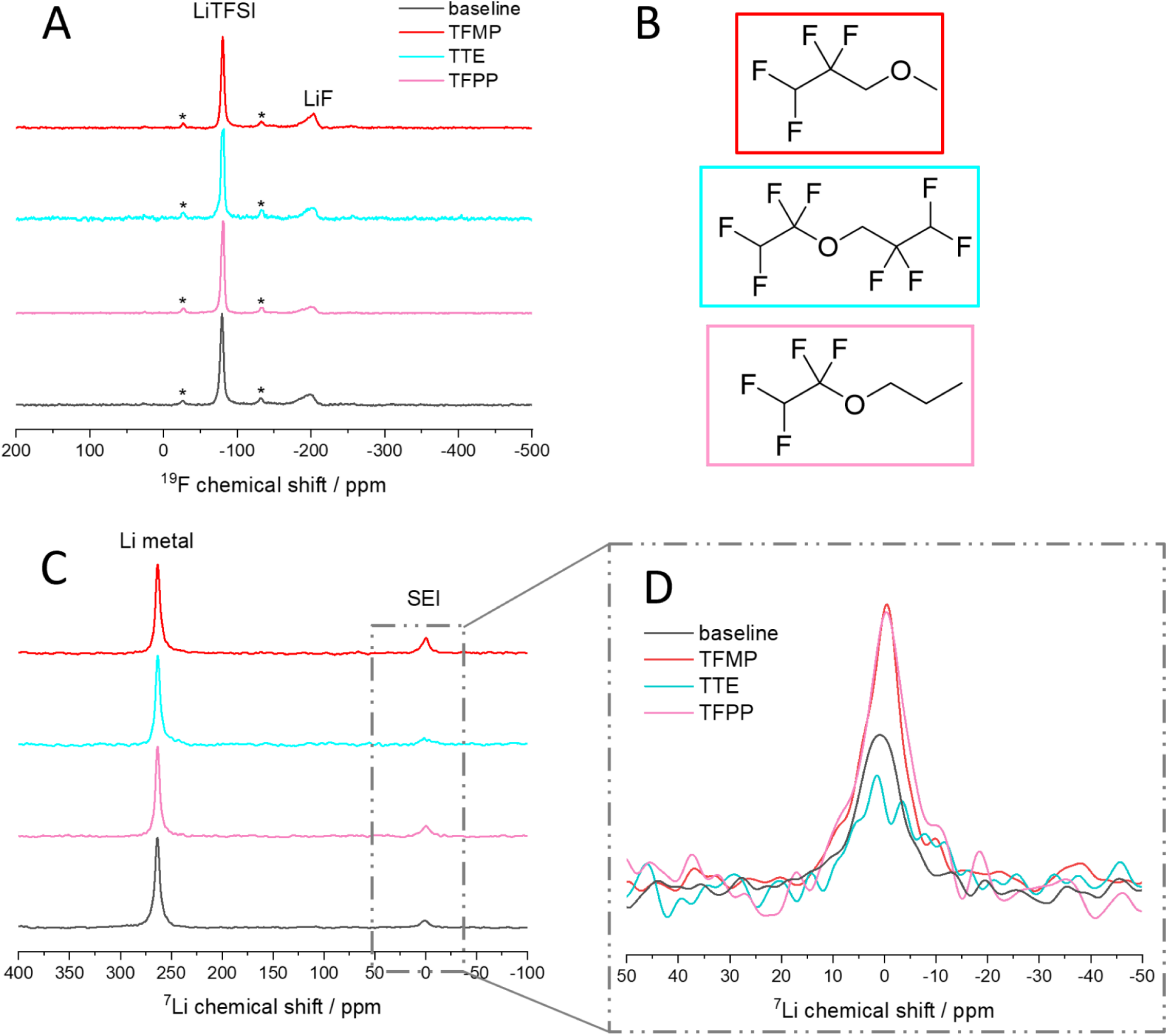
Investigation of the composition of the SEI of lithium metal anodes by *ex situ* solid-state ^19^F (A) and ^7^Li (C, D) MAS NMR measurements of lithium metal deposits formed on a copper current collector in the baseline electrolyte and in electrolytes with fluorinated ethers with the chemical formulas (B) shown in the figure: TFMP (red), TTE (light blue), and TFPP (pink), using a DME:FE solvent ratio of 3:2, where FE is the fluorinated ether. Spinning sidebands are denoted with an asterisk (*).

The ^7^Li MAS NMR spectra (Figure 10C) exhibit a main peak at around ca. 260 ppm due to metallic lithium and a smaller peak close to 0 ppm due to diamagnetic lithium species present in the SEI. Close inspection (Figure 10D) reveals that the latter signal has a lower chemical shift (−0.3 ppm) for the lithium deposits formed with TFMP and TFPP fluorinated ether electrolytes, whereas the shift is higher (1.8 ppm) in the baseline electrolyte and with TTE. These differences can be attributed to a higher concentration of SEI salts such as LiF (−1.0 ppm) and LiTFSI (−1.3 ppm),^32,33^ in which Li is close to F, in the lithium SEI formed with TFMP and TFPP, whereas a higher concentration of compounds in which Li is close to O, such as Li_2_O (2.8 ppm) and LiOH (0.4 ppm),^32^ appears to be present in the baseline electrolyte and TTE. The ^19^F NMR spectra (Figure 10A) confirm the presence of fluorine-containing salts, specifically LiTFSI (−79 ppm) and LiF (−204 ppm),^34^ although part of the LiTFSI signal must be due to salt precipitates (since the samples were not rinsed to avoid disruption of the SEI^35^). The presence of LiF in all spectra suggests that, in all cases, LiF is mainly formed from the decomposition of the LiTFSI salt, as suggested previously,^34^ since LiTFSI is present in all of the studied electrolytes.

*Operando* ^7^Li NMR measurements were performed to monitor the evolution of the morphology of lithium metal anodes induced during cycling in different electrolytes. Experiments were done via unidirectional lithium plating in Li–Li symmetrical cells, with brief interruptions in the application of the current for measuring the cell impedance every hour. As shown in Figure 11 (upper panel), in all cases, the formation of lithium microstructures (e.g., mossy lithium and lithium dendrites) is evidenced by the growth of a ^7^Li NMR signal at higher shifts (ca. 265–275 ppm) than the bulk lithium metal (at ca. 250 ppm). The high sensitivity of these measurements to the formation of lithium microstructures is due to the limited penetration depth of the radiofrequency used for the NMR measurements (by an amount called “skin depth”, of ca. 15 μm) inside the lithium metal electrodes, which thus limits the intensity of the signal due to the bulk lithium metal.^36^ The lithium microstructures appear at a different shift than the bulk lithium metal because, due to the bulk magnetic susceptibility of the material, the loosely packed microstructures growing perpendicular to the electrode surface experience a different magnetic field.^36,37^

**Figure 11:**
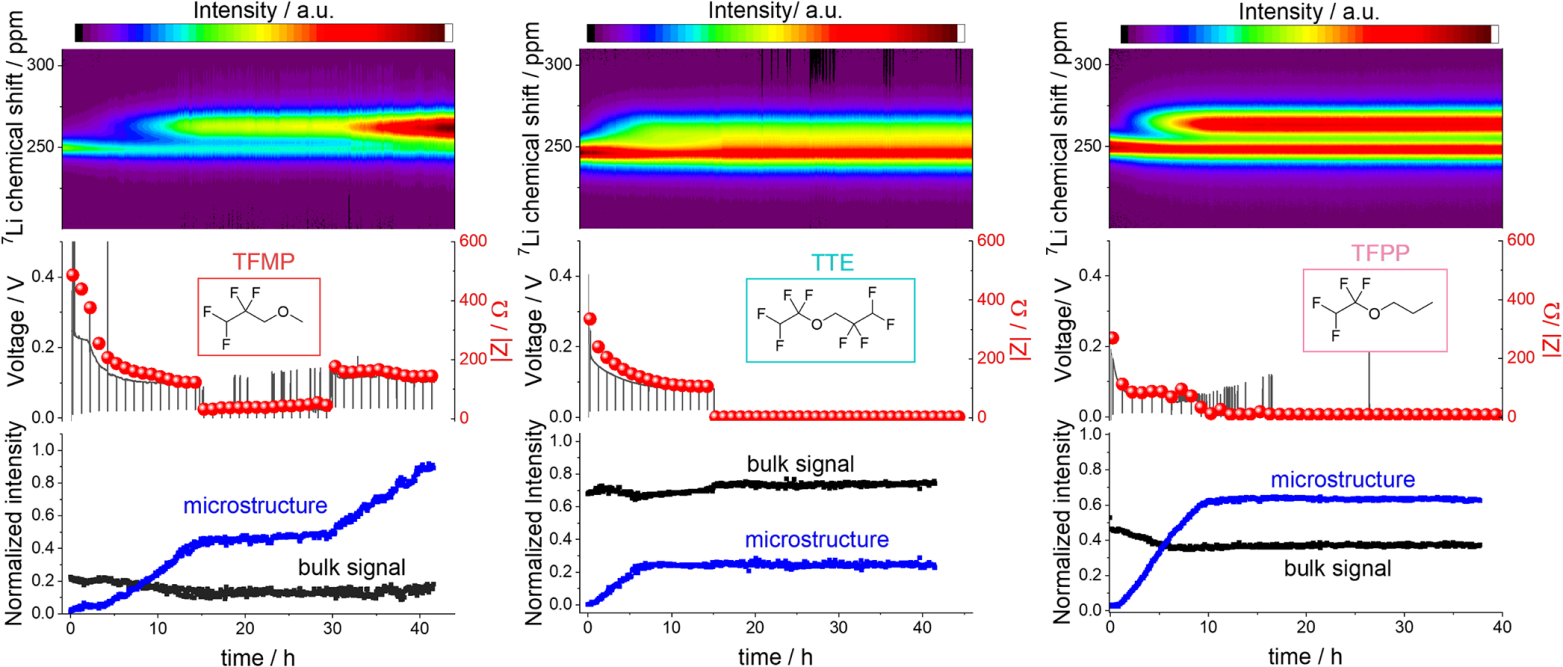
Investigation of the evolution of the morphology of lithium metal anodes by *operando* ^7^Li NMR measurements of symmetrical lithium cells during unidirectional lithium plating experiments, with electrolytes with the fluorinated ethers with chemical formulas shown in the figure: TFMP (left panel), TTE (middle panel), and TFPP (right panel), using a DME:FE solvent ratio of 3:2, where FE is the fluorinated ether. The top panel shows the evolution of the ^7^Li NMR spectra, the middle panel shows the evolution of the cell voltage and cell impedance, and the lower panel shows the evolution of the intensity of the bulk lithium metal signal (at ca. 250 ppm) and of the signal due to lithium microstructures (at ca. 265–275 ppm), both normalized to the total signal at the end of the experiments.

Integration of the ^7^Li NMR signals associated with the bulk lithium metal and lithium microstructures, and normalization to the sum of the intensities of these signals at the end of the experiment, allow us to analyze the temporal evolution of the growth of lithium microstructures, as shown in Figure 11 (lower panel). A slower growth of lithium microstructures is observed with the best-performing fluorinated ether, TFMP, compared to the other two, TTE and TFPP. Indeed, hardly any signal due to lithium microstructures is seen with TFMP during the first 5 h of the experiments, highlighting that the improvement of lithium transport properties by TFMP, discussed in Section 2.2, indeed leads to flatter lithium deposits.

In all the electrolytes, the growth of the lithium microstructure signal is seen to plateau, and a sudden drop of the cell impedance is also observed (Figure 11, lower and middle panels, respectively). These effects can be ascribed to the formation of cell short-circuits, in which the current is carried partially (for soft short-circuits) or entirely (for hard short-circuits) by electrons through an internal connection between the two electrodes, instead of the lithium plating and stripping reactions in the nonshorted cell.^38,39^ The short-circuits are formed as lithium microstructures (dendrites) bridge the two electrodes. However, in the case of the best-performing fluorinated ether, TFMP, the internal short-circuit is achieved much later, after around 15 h of plating (15 mAh cm^–2^ of plating capacity), whereas in TTE and TFPP, the internal short-circuit is achieved after around 6 and 10 h of plating, respectively. Note that in TTE, the cell impedance drop occurs later, as the short-circuit initially has high resistance, and thus operando ^7^Li NMR is, in this case, essential to detect the early appearance of the short-circuit.

Importantly, the short-circuit in TFMP is soft (associated with impedance >10 Ω cm^2^, thus resulting in a small internal current), whereas in TTE and TFPP, the short-circuit is hard (with negligible resistance, thus triggering a higher internal current). The distinction between soft and hard short-circuits is done based on the impedance data,^40^ and details of the analysis are provided in Section S6. In addition, the short-circuit observed in the TFMP electrolyte is temporal, whereas with TTE and TFPP, the short-circuit is permanent. It is also important to note that recent work has shown that soft short-circuits, as seen in TFMP, are not able to trigger thermal runaway or fires.^41,42^

The higher resistance during the soft short circuit in TFMP evidences the beneficial role of TFMP in promoting a more protective (that is, more passivating) lithium SEI. Consequently, the direct lithium metal–metal contact, when the dendrite touches the counter-electrode, is prevented by the presence of such a stable and electronically insulating lithium SEI. This behavior is in agreement with the higher content of fluorinated compounds (e.g., LiF) revealed by the *ex situ* ^7^Li NMR of the lithium SEI in TFMP. In addition, the temporal nature of the short-circuit in TFMP suggests that lithium-ion transport through the lithium SEI is fast and, consequently, facilitates the dissolution of the lithium dendrite (since the dendrite is thermodynamically less stable than flat deposits) and also minimizes plating hot spots (as not enough dendrites are formed to create a stable short circuit). Indeed, EDX analysis of cycled lithium electrodes indicates that the lithium SEI is thinner in TFMP, as discussed below.

Postmortem SEM and EDX analysis of electrodes from cycled Li–S cells were performed to further characterize the changes in morphology and composition of the electrodes upon cycling. Figure 12 shows that the lithium electrodes cycled in the TFMP electrolyte exhibit significantly larger and more regular grain sizes compared to the baseline formulation. These differences are further exacerbated with longer cycling, where the lithium electrodes cycled with TFMP maintain a similar grain size, whereas the lithium electrodes cycled in the baseline electrolyte show a highly irregular surface (Figure S9). These results are fully consistent with the beneficial effect of TFMP in suppressing lithium microstructure growth, as shown in the *operando* NMR data (Figure 11).

**Figure 12:**
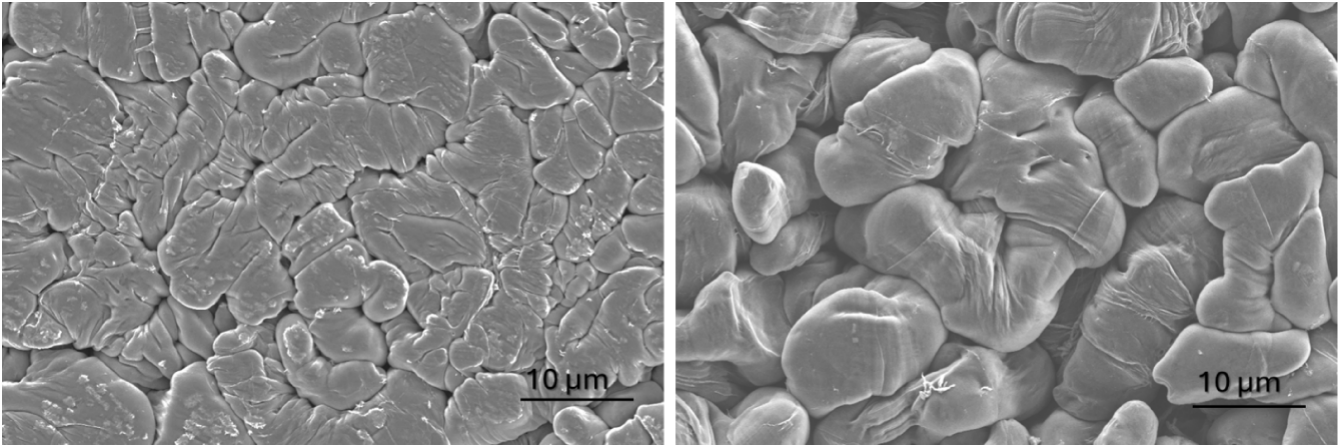
SEM characterization of lithium electrodes after cycling in the baseline electrolyte (left) and TFMP electrolyte (right) for five cycles of discharge and charge in Li–S cells.

Furthermore, the coupled SEM + EDX analysis of cycled lithium electrodes (Figure 13) reveals that the lithium electrodes from the TFMP electrolyte exhibit a much more spatially homogeneous elemental composition compared to the baseline electrolyte, and these differences are again exacerbated with cycling (Figures S10–S11). The improvement in the spatial homogeneity of the reactions by TFMP is thus identified as the key reason behind the improved battery performance, in agreement with the improvement in the lithium-ion transport properties achieved by TFMP, as revealed by the molecular dynamics simulations (Figure 8).

**Figure 13:**
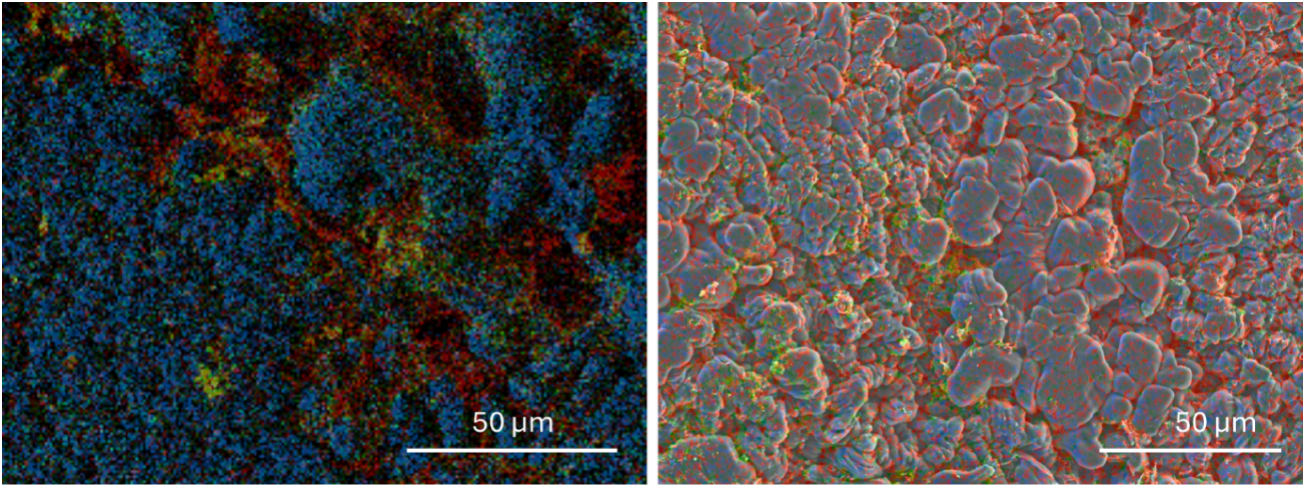
SEM + EDX characterization of the lithium electrodes in Figure 12, cycled in the baseline electrolyte (left) and TFMP electrolyte (right) for five cycles in Li–S cells, where colors represent lithium (blue), fluorine (green), and sulfur (red). Note that the detection of elemental lithium is achieved by using a windowless EDX detector.

The EDX characterization of the cycled lithium electrodes also reveals a change in the average elemental composition of the electrodes after cycling in the baseline and TFMP electrolytes (Table S4). Specifically, lithium electrodes from TFMP electrolytes have a higher content of elemental lithium and a smaller content of sulfur compared to the baseline electrolyte, particularly after cycling for 50 cycles. These differences suggest that TFMP promotes the formation of a thinner lithium SEI (thus enhancing the signal of the underneath lithium electrode during the EDX measurements), as well as a stronger suppression of the parasitic reaction of lithium corrosion by polysulfides producing Li_2_S deposition (e.g., via Li_2_S_*n*_ + (2*n* – 2) Li → *n*Li_2_S). A small increase in the fluorine content is also observed in TFMP-cycled lithium electrodes, consistent with the results from the *ex situ* solid-state NMR characterization that suggest a higher degree of fluorination of the lithium SEI (Figure 10), which, in the literature, has often been seen to be correlated with improved performance.^43,44^

Finally, the coupled SEM + EDX analysis of the cycled sulfur electrodes (Figure 14) reveals that, in the baseline electrolyte, nearly all of the electrode surface is covered by sulfur (presumably Li_2_S, since the electrodes were characterized in the discharged state), whereas the electrode cycled in TFMP shows significant areas of bare carbon. Similar results are obtained with sulfur electrodes that have undergone just one discharge (Figure S12), showing the presence of bare carbon domains on the TFMP-cycled electrode, whereas the baseline-cycled electrode is covered by sulfur-containing grains, presumably of the Li_2_S discharge product. The average compositions of the electrodes also confirm a higher content of bare carbon in the presence of TFMP (Table S5).

**Figure 14:**
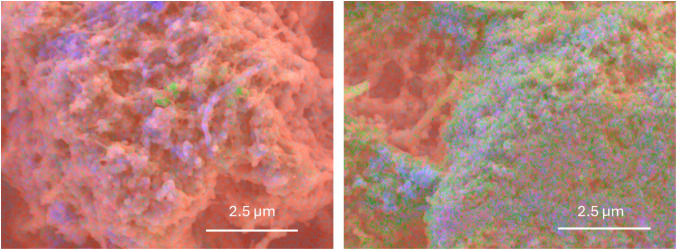
SEM + EDX characterization of cycled sulfur electrodes in the baseline electrolyte (left) and TFMP electrolyte (right), after cycling for five cycles in Li–S cells with the end of cycling in the discharged state, where colors represent carbon (blue), fluorine (green), and sulfur (red).

Prior literature demonstrated that polysulfide chemical reactions, such as those in eq 1, are key to achieve high capacities and high rechargeability of Li–S batteries.^45–47^ Such a chemical pathway for producing Li_2_S as the discharge product was also shown to be key to avoid the passivation of the carbon conductive additive in the sulfur electrode, which would otherwise occur if Li_2_S was only formed electrochemically (e.g., via Li_2_S_2_ + 2e^–^ + 2Li^+^ → 2Li_2_S).^28^ Thus, the absence of carbon passivation in TFMP-containing electrolytes suggests that TFMP enhances the rate of polysulfide chemical reactions, and it is likely that such enhancement stems from the promotion of a more dynamic lithium-ion coordination environment by TFMP. In polysulfide chemical reactions, lithium ions move from coordinating one polysulfide to coordinating another polysulfide (Figure S8), which also necessarily requires adjustments in the lithium-ion solvation, and the latter will be more facile in systems in which all solvents have similar lithium binding energies.

To summarize, Figure 15A illustrates the various beneficial mechanistic routes promoted in Li–S batteries with electrolyte systems in which all solvents have similar lithium binding energies, which are associated with a high lithium transference number and more dynamic lithium-ion solvation, while Figure 15B highlights the various detrimental reactions promoted in electrolyte systems with components with dissimilar lithium binding energies, which are associated with a high activation energy for lithium-ion motion and solvation reorganization.

**Figure 15:**
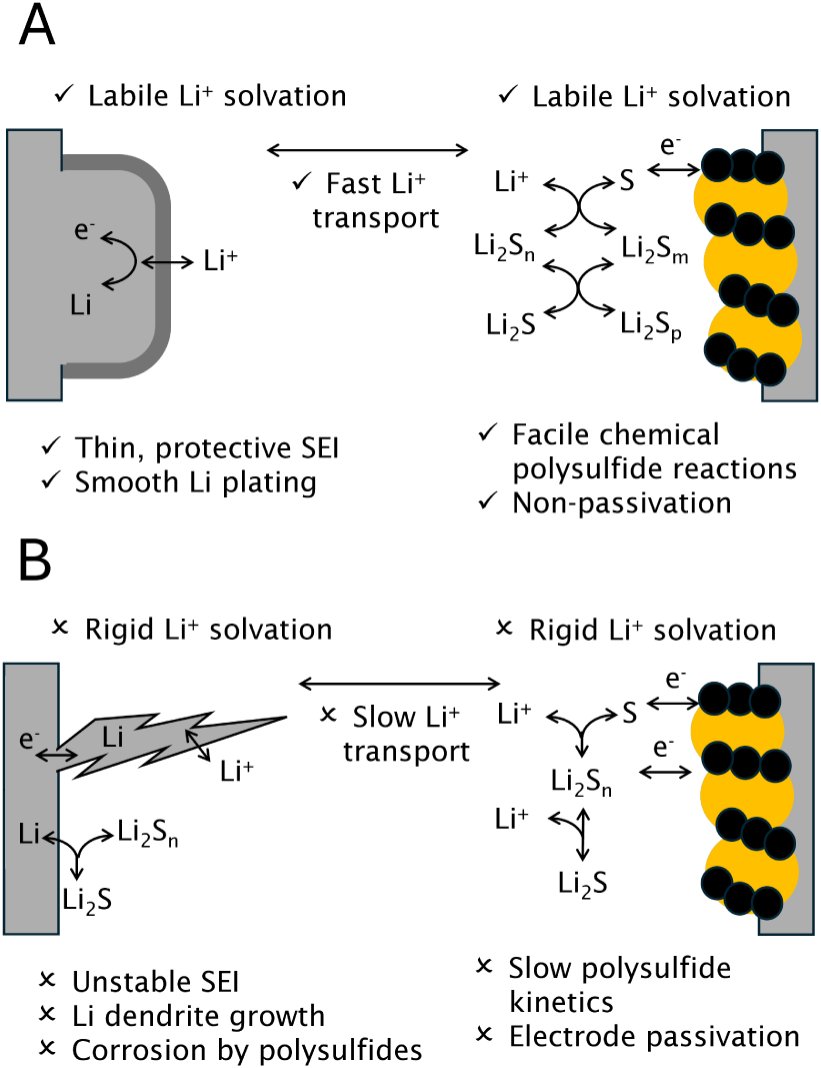
Illustration of the beneficial (A) and detrimental (B) mechanistic routes promoted in Li–S batteries with electrolytes with low (A) or high (B) energy barriers toward lithium-ion motion and solvent exchange reactions.

## 3 Conclusions

The improvement of Li–S battery performance using fluorinated ethers has been studied in many articles, but few have studied more than one fluorinated ether. Since fluorinated ethers exhibit weak intermolecular interactions (which lead to advantageous properties such as low viscosity), it has been largely overlooked that tuning the extent of such interactions, via tuning the chemical formula of the fluorinated ether, can lead to substantial performance improvements.

Here we study Li–S batteries that combine fluorinated and nonfluorinated ethers, which are of commercial interest due to their promising electrochemical performance, and we find that a stronger bonding of the fluorinated ether to lithium-containing species leads to a higher lithium transference number that is correlated with improved Li–S battery longevity and rate capability. The high lithium transference number is achieved thanks to the similarities in the coordination strengths of lithium ions to the different solvents in the electrolyte, which promote fast solvent exchange reactions and, consequently, more beneficial mechanisms for both the lithium and the sulfur electrode reactions. The asymmetry in the chemical formula and the absence of fluorination on the carbon next to the oxygen ether of the fluorinated ether were found to be key properties that facilitated the interaction with lithium ions, while the overall degree of fluorination had a small effect. The best battery performance was obtained with TFMP (1,1,2,2-tetrafluoro-3-methoxypropane; Figure 16), which promoted homogeneous lithium reactions leading to smoother lithium deposits with a thinner and more passivating lithium SEI, and it also prevented the passivation of the carbon in the sulfur electrode, likely by promoting lithium polysulfide chemical reactions.

**Figure 16:**
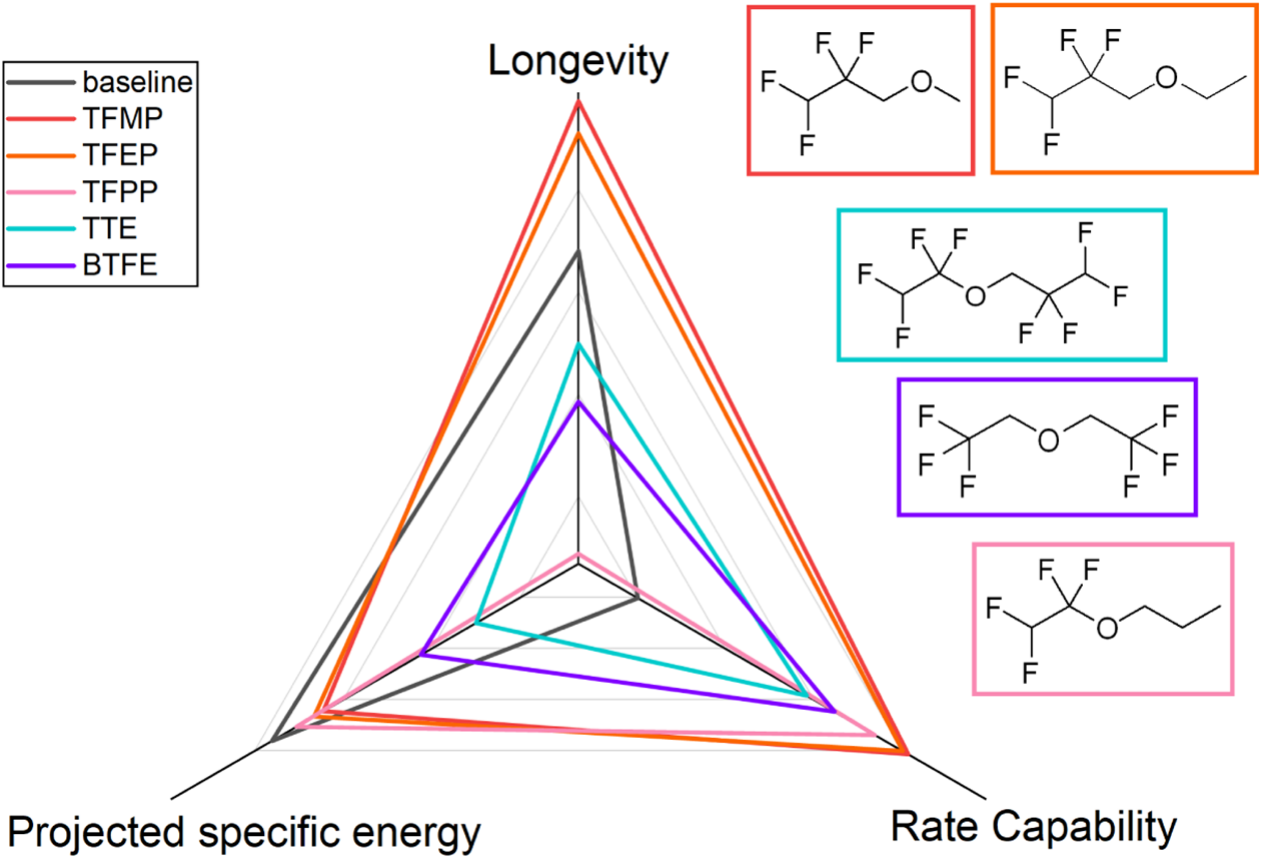
Radar chart comparing the effect of selected fluorinated ethers on Li–S battery longevity (quantified as the capacity at the 100^th^ C/5 cycle, see section S7), rate capability (quantified as the capacity at 1C cycling, see section S7), and projected specific energy (calculated taking into account the density of the fluorinated ether, see section S8).

Although the requirements for the properties, and thus the chemical structure, of the fluorinated ether may change depending on the battery formulation and the commercial application, the need to study a range of fluorinated ethers to optimize battery performance is undeniable. This work shows that the lithium transference number is the bulk electrolyte property that most critically affects battery performance, and it demonstrates that designing electrolyte formulations in which all of the electrolyte components have similar lithium binding energies, so that there is no lithium trapping in one strongly bound configuration, is an effective approach to enhance it.

## Supplementary Material



## Notes

Experimental procedures and computational methods, overview of previous literature using fluorinated ethers in Li–S batteries, additional experimental and computational results (with additional electrochemical data of Li–S batteries, values of diffusion coefficients and bond distances from MD simulations and additional SEM + EDX characterization data of cycled electrodes), discussion about determination of transference numbers, calculations of Sand’s time and diffusion limited current, discussion of the combined impedance and *operando* ^7^Li NMR characterization of lithium metal anode reactions, analysis of effect of cell-to-cell reproducibility on performance metrics, calculations of projected specific energies and energy densities of Li–S batteries, and repeat experiments demonstrating reproducibility (PDF)

## Abbreviations

BTFE: bis­(2,2,2-trifluoroethyl) ether
DME: 1,2-dimethoxyethane
DOL: 1,3-dioxolane
EDX: energy-dispersive X-ray spectroscopy
ETFE: 1,1,2,2-tetrafluoro-1-ethoxy-ethane
FDMB: fluorinated 1,4-dimethoxylbutane
MAS: magic angle spinning
NMR: nuclear magnetic resonance
TFEE: 1,2-(1,1,2,2-tetrafluoroethoxy)­ethane
SEM: scanning electron microscopy
TFEP: 1,1,2,2-tetrafluoro-3-ethoxy-propane
TFFMP: 1,1,2,2-tetrafluoro-3-(fluoromethoxy)­propane
TFMP: 1,1,2,2-tetrafluoro-3-methoxypropane
TFPP: 1-(1,1,2,2-tetrafluoroethoxy)­propane
TTE: 1,1,2,2-tetrafluoroethyl 2,2,3,3-tetrafluoropropyl ether
