# Supplementary material for: Structure–Property Relationships to Guide the Selection of Fluorinated Ethers for Li–S Batteries
Source: J Am Chem Soc. 2026 Jul 26;148(30):31896–908. doi: 10.1021/jacs.6c04480 (PMC13449949; doi:10.1021/jacs.6c04480)
Supplement: Supplementary file 1 [file ja6c04480_si_001.pdf]

Supporting information

Structure-property relationships to guide the selection of fluorinated ethers for Li-S batteries

Mark Stockham[a,d†], Neubi F. Xavier Jr.[b†], Jana B. Fritzke[c,d,e†], Samuel D.S. Fitch[a,d], Liam Furness[a,d], Vikram K. Bharti[a,d], Nikolay Zhelev[a], Qiong Cai[b,d], Clare P. Grey[c,d,*], Nuria Garcia-Araez[a,d,*]

a: School of Chemistry and Chemical Engineering, University of Southampton, SO17 1BJ, Southampton, United Kingdom

b: School of Chemistry and Chemical Engineering, University of Surrey, GU2 7XH, Guildford, United Kingdom

c: Yusuf Hamied Department of Chemistry, University of Cambridge, CB2 1EW, Cambridge, United Kingdom

d: The Faraday Institution, Harwell Campus, OX11 0RA, Didcot, United Kingdom

e: Chemistry of Interfaces, Department of Civil Environmental and Natural Resources Engineering, Luleå University of Technology, SE-97187, Luleå, Sweden

†: M.S., N.F.X and J.B.F contributed equally

Content:

S1. Experimental procedures and computational methods ……………………….…page 2

S2. Overview of previous literature using fluorinated ethers in Li-S batteries…..........page 6

S3. Additional experimental and computational results....……………………………..page 15

S4. Discussion about the determination of transference numbers …..………………...page 24

S5. Calculations of Sand's time and diffusion limited current…………………………page 27

S6. Discussion of the combined impedance and *operando* ^{7}Li NMR characterization of lithium metal anode reactions……………………………………………………......page 28

S7. Analysis of the effect of cell-to-cell reproducibility on performance metrics....…...page 33

S8. Calculations of projected specific energies and energy densities of Li-S batteries...page 39

S9. Repeat experiments demonstrating reproducibility…………………….……..page 42

References…………………………………………………………………...…page 75

S1. Experimental procedures and computational methods

S1.1 Materials

The fluorinated ethers were obtained as follows:

-TFMP: 1,1,2,2-tetrafluoro-3-methoxypropane, also known as methyl 2,2,3,3-tetrafluoropropyl ether, Apollo Scientific or Manchester Organics, 99% or 97%.

-FDMB: fluorinated 1,4-dimethoxylbutane, also known as 2,2,3,3-tetrafluoro-1-4-dimethoxybutane, Tokyo Chemical Industry UK, 98%.

-TFEP: 1,1,2,2-tetrafluoro-3-ethoxy-propane, also known as ethyl 2,2,3,3-tetrafluoropropyl ether, Synquest, 97%.

-ETFE: 1,1,2,2-tetrafluoro1-ethoxy-ethane, also known as ethyl 1,1,2,2-tetrafluoroethyl ether, Apollo Scientific, 99%.

-TFPP: 1-(1,1,2,2-tetrafluoroethoxy)propane, also known as propyl 1,1,2,2-tetrafluoroethyl ether, Apollo Scientific, 99 %.

-BTFE: bis(2,2,2-trifluoroethyl) ether, Apollo Scientific, 99 %.

-TFEE: 1,2-(1,1,2,2-tetrafluoroethoxy)ethane, Apollo Scientific, 99 %.

- TTE: 1,1,2,2-tetrafluoroethyl 2,2,3,3-tetrafluoropropyl ether, Manchester Organics, 97 %.

- TFFMP: 1,1,2,2-tetrafluoro-3-(fluoromethoxy)propane, also known as fluoromethyl 2,2,3,3-tetrafluoropropyl ether, Manchester Organics, 97%.

For the preparation of the electrolytes, LiTFSI (lithium bis(trifluoromethanesulfonyl)imide, Sigma-Aldrich, anhydrous, 99.99%) and $LiNO_3$ (lithium nitrate, Sigma-Aldrich, 99.99%) were dried at 120 °C under vacuum overnight. DME (1,2-dimethoxyethane, Sigma-Aldrich, anhydrous, 99.5%), DOL (1,3-dioxolane, Sigma-Aldrich, anhydrous, 99.8%) and the fluorinated eithers were dried with 4 Å molecular sieves for three days (8–12 mesh bead size, Aldrich). The sieves were first dried under vacuum in a Büchi tube at 220 °C for 72 h. The tube was then sealed and transferred to a glovebox, where the sealed tube was left to cool overnight before the sieves were used. The salt concentrations in the electrolytes are reported as moles of salt per liter of solvent mixture.

The preparation of the sulfur electrodes was done by following the procedure described previously.[1] The electrodes were made of 65% S (sulfur, Sigma-Aldrich, 99.98%), 21% KB (Ketjenblack, EC-600JD, NanoGrafi), 3.5% C65 (Super C65, Imerys), 3.5% CNF (carbon nanofibers, 20–200 nm × 100 µm, Sigma-Aldrich), 5.6% PEO (poly(ethylene oxide, Mw ~4000,000, Sigma-Aldrich) and 1.4% PVP (polyvinylpyrrolidone, Mw ~360,000, Sigma-Aldrich), where the percentages are given by weight. The sulfur and carbon additives were first manually mixed with pestle and mortar and then heated in a porcelain crucible with a lid, wrapped in foil, to 155 °C for 1-2 h to melt infiltrate the sulfur into the carbon. The resulting sulfur-carbon composite was then mixed with the binders, and a viscous ink was made using ultrapure water (18.2 MΩ) with 7.5 wt.% ethanol (Fisher Scientific, 99.7%) as the solvent, using ~3.3 mL of solvent per gram of solids. The inks were mixed with a Thinky mixer (ARE-250) at 1300 rpm for 5 min, then at 1800 rpm for 5 min and then at 2000 rpm for 5 min, with

5 min breaks in between the mixing steps for cooling. The inks were cast using a doctor blade onto carbon-coated aluminum foil (MTI), thus obtaining sulfur electrodes with sulfur loadings close to 3 mg/cm^2. The sulfur electrodes were cut into 14-mm diameter discs using a precision puncher (Nogami) and dried at room temperature under vacuum in a Büchi tube for at least 2 days. Celgard 2400 was used as the separator, these were cut into 16-mm diameter discs and dried under vacuum in a Büchi tube at 60°C for 72 hours.

S1.2. Electrochemical testing and electrode characterization

The assembly of the electrochemical cells and preparation of the electrolytes were done inside an argon-filled glovebox (Unilab, MBraun, H_2O and O_2 content <0.1 ppm).

The electrochemical testing was done in CR2032 coin cells (Seika Sangyo GmbH Germany) using a HS-HCR2 hand-operated coin crimper (Seika Sangyo GmbH Germany). First, a 15 mm-diameter lithium disc (battery grade, China Energy Lithium Co., 100 microns thick) was pressed into a 0.5mm coin cell spacer and placed inside the coin cell case with a coin cell gasket attached. A 16 mm- diameter Celgard 2400 separator was placed on top of the lithium disc, and then the separator was wet with an electrolyte volume equivalent to an electrolyte-to-sulfur ratio of 7 µL/mg. A 14-mm diameter sulfur electrode was then placed centrally on top of the separator followed by an additional 0.5 mm spacer and a square wave spring. The cell was then sealed with the coin cell cap and crimped. After assembly, cells were introduced into a climatic chamber (Memmert) set at 25 °C and rested for 2 hours at OCV, followed by C/5 cycling between 1.8 and 2.6 V. The specific capacities were normalized by the mass of sulfur in the sulfur electrode.

Note that, due to the significant empty space volume inside coin cells (known as 'dead volume'), it is not possible to achieve the low electrolyte loading achievable with pouch cells.[2] This is because, during coin cell assembly, the electrolyte fills the whole cell 'dead volume', so the actual volume of electrolyte inside the cell stack (comprising the electrodes and separator) is much lower than the total volume of electrolyte added to the cell. Here we employ a low volume of electrolyte (30-35 µL) that provides high cell-to-cell reproducibility, and it corresponds to an electrolyte-to-sulfur ratio of 7 µL/mg. that is among the smallest used in coin cells in the literature (see Tables S1-2).

For the SEM characterization, the cycled cells were disassembled in the glovebox and the electrodes were rinsed with ~3 mL of DME to remove salt and polysulfide residues. The solvent was then allowed to dry off in the glovebox and the electrodes were stored face up in glass vials. The cycled electrodes were loaded into a SemiLab sample transfer suitcase and reopened under vacuum inside the transfer chamber of the SEM (Zeiss Sigma 500 VP FESEM). EDX measurements were done using an Oxford Instruments Ultim Extreme 100 mm^2 windowless detector that enabled the detection of elemental lithium kα X-rays, SEM images show secondary electron micrographs and EDX data was obtained at a 5 kV acceleration voltage unless stated otherwise.

For the *ex-situ* NMR characterization, thin lithium deposits were prepared by galvanostatic plating of lithium on copper current collectors in Li//Cu coin cells (304, Cambridge Energy Solutions) with Celgard 2325 separators at 1 mA cm^{-2} for 1 h, followed by 48 h resting at OCV. Using Faraday's law, the bulk thickness of the deposits is estimated to be of ca. 5 µm. Then,

the cells were disassembled inside an argon glovebox and the electrode was dried under vacuum for 10 min and afterwards the lithium deposits were removed from the copper surface using a spatula. The lithium deposits were then mixed thoroughly with dried (40 h at 200 C) KBr (5:1 or 10:1 w/w) using a mortar and pestle inside the glovebox; this was done to limit electrical conductivity of the samples and, with this, reduce eddy currents.[3]

The solid-state NMR experiments were performed on a Bruker Avance III 500 MHz spectrometer equipped with a 1.3 mm HFXY MAS Bruker NMR probehead. All spectra were collected at room temperature with 25 kHz spinning speed with a 1.3 mm ZrO_2 rotor. ^{7}Li solid-state NMR experiments were performed using a hahn-echo pulse sequence (90°–τ–180°–τ–acquire, with τ = 36.9 µs), a recycle delay of 5 s, a power of 100 W with a pulse length of 1.06 µs and 256 scans. ^{19}F solid-state NMR experiments were performed using a hahn-echo pulse sequence (90°–τ–180°–τ–acquire, with τ = 38.4 µs), a recycle delay of 5 s, a power of 50 W with a pulse length of 1.3 µs and 16220 scans.

The *operando* NMR experiments were conducted on a Bruker Avance 300 MHz spectrometer (the Larmor frequency for ^{7}Li being 116.6 MHz) using a solenoidal Ag-coated Cu coil. The spectra were recorded using an *in-situ* automatic-tuning-and-matching probe (ATM VT X *in-situ* WB NMR probe, NMR Service) that allows for an automatic recalibration of the NMR rf-circuit during an *in-situ* electrochemistry experiment. The retuning of the rf-circuit becomes essential in order to quantify the NMR signals when the sample conditions are changing during the electrochemistry.[4] The probe has highly shielded wire connections to the electrochemistry with low-pass filters (5 MHz) attached to the probe, minimizing the interferences between the NMR- and the electrochemistry circuit. Overall, the *in-situ* setup allows for highly reproducible NMR measurements. Single-pulse experiments were used to collect the NMR data, with a recycle delay of 1 s (>5 × T1) and 256 transients recorded. This resulted in an experimental time of about 4.5 min. The shift of ^{7}Li was referenced to 1 M LiCl in water at 0 ppm. The spectra were processed in the Bruker Topspin software using the automatic phase and baseline correction. Further data processing was done in R. The total intensity of the Li metal peak was integrated over the ^{7}Li shift range of 310–220 ppm and normalized to the intensity measured at the end of plating.

For the *in-situ* NMR experiments, a current density of 1 mA cm^{-2} was applied for 40 h. A capsule cell (NMR Service) made out of PEEK was used for all *in-situ* NMR experiments and has been described before.[4–6] The cell was assembled with lithium metal foil electrodes, an Advantec glass fiber separator and a 0.5 cm thick Viton® rubber spacer. The amount of electrolyte added to each cell was 150 µL. Every 1 h, the current was stopped for 9 s to record impedance measurements using a current amplitude of 100 µA amplitude and a frequency range of 500-0.8 Hz, and the magnitude of the impedance was evaluated at 9 Hz. The impedance data are normalized by the geometrical area of the electrodes (0.5 cm^2).

S1.3. Computational methods

Molecular dynamics (MD) calculations were performed with the aid of Battflow,[7] an automated workflow for molecular dynamics simulations of liquid electrolytes. MD simulations were conducted with the GROMACS package, version 2021.5.[8] To prepare for MD simulations, we constructed each simulation box of around 6 nm, with electrolyte molar ratios equal to those

used in the experiments, by randomly displacing the species using the PACKMOL software.[9] The interatomic interactions of molecules and ions were described by the Optimized Potentials for Liquid Simulations All-Atom (OPLS-AA) force field,[10] in which the topology files were generated using the LigParGen server.[11–13] Bonded and non-bonded parameters for the anions adopted here ($TFSI^-$ and NO_3^-), as well as partial charges, were obtained from Doherty et al.[14]

The following steps were employed for MD simulations: (i) configurations were relaxed through 5000 steps of the steepest descent method; (ii) equilibration was done through NVT simulations at 300 K for 2 ns, followed by NPT simulations in which reference pressure was set to 1 bar; (iii) production runs of 50 ns, under the NPT ensemble, were performed using the V-rescale temperature coupling thermostat and Parrinello-Rahman pressure coupling barostat at the same conditions of the equilibration steps. Long-range electrostatic interactions were calculated through the particle mesh Ewald method, assuming a cut-off of 1.2 nm and a Fourier spacing of 0.10 nm. Periodic boundary conditions were applied in all directions. Bonds with hydrogen atoms were constrained using the LINCS algorithm. The leap-frog algorithm was used to integrate the equations of motion with a 2.0 fs time step. Steps were saved in the trajectory files every 20 ps. The final 25 ns of the production run were used for further analysis.

Solvation sheath statistics were computed through scripts based on the MDAnalysis[15] and SolvationStructure[16] Python modules. We calculated the self-diffusion coefficients for the ions present in the electrolyte composition (D^{ion}) by performing the linear regression of the mean square displacement (MSD) as a function of time. The MSD was calculated following the Einstein relation, described as follows:[17]

$$D^{ion} = \frac{1}{6} \lim_{t \to \infty} \frac{d}{dt} MSD(t) \tag{1}$$

$$MSD(t) = \langle \frac{1}{N} \sum_{i=1}^{N} |r_i(t) - r_i(0)|^2 \rangle \tag{2}$$

where N is the number of particles present in the MSD calculations, and r_i are the coordinates for a given ion i at a time t. We used the self-diffusion coefficients to calculate the apparent transference number for Li^+ (t_{Li^+}) in all simulated systems, as defined in Equation 3.

$$t_{Li^+} = \frac{D^{Li^+} N^{Li^+}}{D^{Li^+} N^{Li^+} + D^{NO_3^-} N^{NO_3^-} + D^{TFSI^-} N^{TFSI^-}} \tag{3}$$

where N^{ion} is the number of each ion present in the simulated electrolyte composition.[18,19] A further discussion about how the apparent lithium transference numbers determined here by MD simulations are related to other methods of determination of transference numbers is presented in section S4.

Density Functional Theory (DFT) was used to perform geometry optimizations of the geometries of Li^+ ion and Li_2S_4 cluster interacting with the fluorinated ethers studied here. The most stable configuration of the Li_2S_4 cluster was extracted from the literature.[20] Calculations were conducted at the B3LYP/6-311G++(d, p) level of theory. All simulations were carried out with the ORCA 5.0.4 package.[21] Binding energy (BE) values were calculated as described in Equation 4:

$$BE = E_{X-FE} - E_X - E_{FE} \tag{4}$$

where X is either Li^+ or Li_2S_4, and FE is the fluorinated ether.

S2. Overview of previous literature using fluorinated ethers in Li-S batteries

Table S1. Overview of fluorinated ethers used as co-solvents with non-fluorinated ethers in Li-S batteries.[a]

Fluorinated ether	Testing conditions	Electrochemical results	Reference
TTE 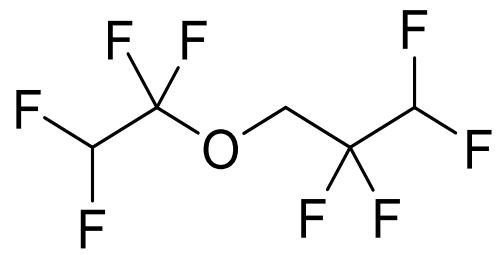	Sulfur content in sulfur electrode: N/A<br>Sulfur loading: 3.9-4.2 mg cm^{-2}<br>Electrolyte: 1 M LiTFSI in DOL:TTE (1:1)<br>Electrolyte to sulfur ratio: N/A	Initial capacity: ca. 1400 mAh/g at C/10<br>Capacity retention: ca.1000 mAh/g after 50 cycles at C/10	[22]
TTE 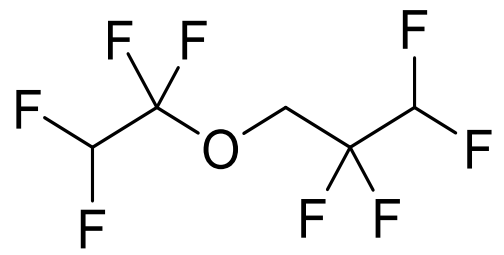	Sulfur content in sulfur electrode: 56 wt%<br>Sulfur loading: 5 mg cm^{-2}<br>Electrolyte: 1 M LiTFSI + 0.2M $LiNO_3$ in DOL:TTE (1:1)<br>Electrolyte to sulfur ratio: 6.3 µl/mg in coin cells	Initial capacity: ca. 1050 mAh/g at C/10<br>Negligible self-discharge after 10 h at 55 °C	[23]
TTE 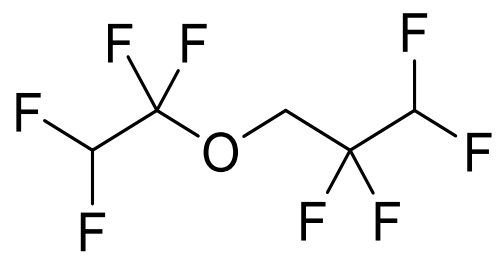	Sulfur content in sulfur electrode: 41 wt%<br>Sulfur loading: 1-1.5 mg cm^{-2}<br>Electrolyte: 1 M LiTFSI in DOL:TTE (1:1)<br>Electrolyte to sulfur ratio: N/A	Initial capacity: ca 1200 mAh/g at C/10<br>Capacity retention: ca 600 mAh/g after 150 cycles at C/10	[24]
TTE 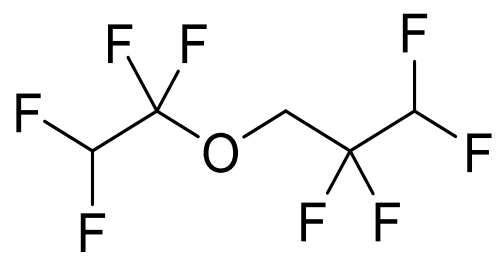	Sulfur content in sulfur electrode: 60 wt%<br>Sulfur loading: 5 mg cm^{-2}<br>Electrolyte: 1 M LiTFSI in DOL/TTE (1:2)<br>Electrolyte to sulfur ratio: N/A	Initial capacity: ca 1600 mAh/g at C/10<br>Capacity retention: ca 1100 mAh/g after 50 cycles at C/10	[25]
TTE 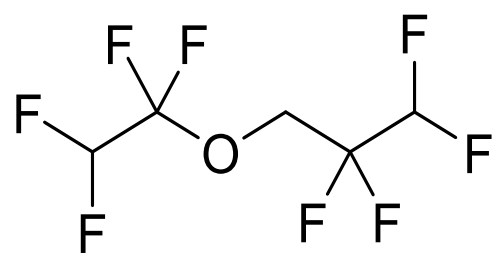	Sulfur content in sulfur electrode: 46 wt%<br>Sulfur loading: N/A<br>Electrolyte: 1 M LiTFSI in DOL/TTE (1:1)<br>Electrolyte to sulfur ratio: N/A	Initial capacity: ca 1050 mAh/g at C/10 and ca 900 mAh/g at C/3.3 (3rd cycle)<br>Capacity retention: ca. 800 mAh/g after 100 cycles at C/10 and ca. 600 mAh/g after 300 cycles at C/3.3	[26]
TTE 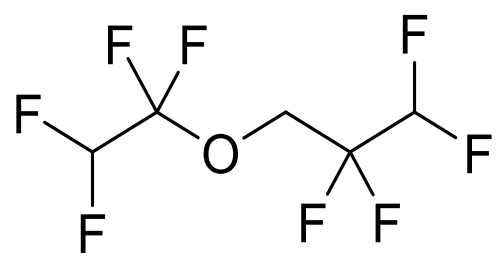	Sulfur content in sulfur electrode: 55 wt%<br>Sulfur loading: N/A<br>Electrolyte: 1 M LiFSI in DME/TTE (4:96)<br>Electrolyte to sulfur ratio: ca. 5.5 µl/mg in coin cells	Initial capacity: ca 1000 mAh/g at C/17 in coin cells (2nd cycle)<br>Capacity retention: ca 800 mAh/g after 50 cycles at C/17	[27]

TTE<br>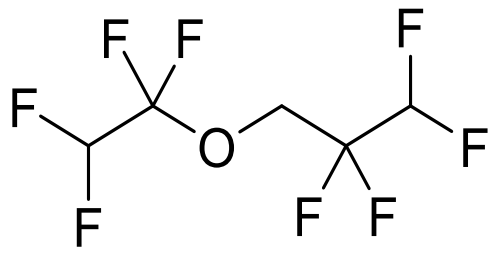	Sulfur content in sulfur electrode: 44 wt%<br>Sulfur loading: 2 mg cm^{-2}<br>Electrolyte: 1 M LiTFSI in DME/TTE (1:1)<br>Electrolyte to sulfur ratio: N/A	Initial capacity: 1100 mAh/g at C/10<br>Capacity retention: 800 mAh/g after 100 cycles at C/10	[28]
TTE<br>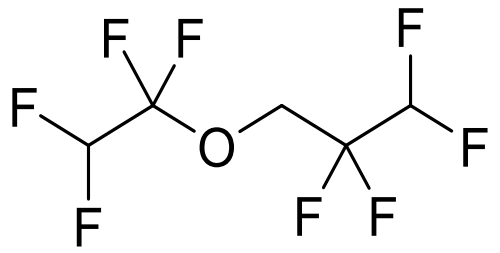	Sulfur content in sulfur electrode: 50 wt%<br>Sulfur loading: 1.8 mg cm^{-2}<br>Electrolyte: 1 M LiTFSI in DOL:TTE (1:1)<br>Electrolyte to sulfur ratio: 10 µl/mg in coin cells	Initial capacity: ca 750 mAh/g at C/10 (2nd cycle)<br>Capacity retention: ca 450 mAh/g after 200 cycles at C/10	[29]
TTE<br>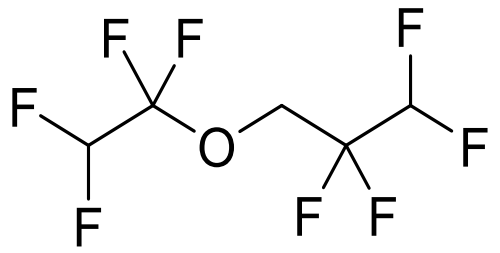	Sulfur content in sulfur electrode: 50 wt%<br>Sulfur loading: 2 mg cm^{-2}<br>Electrolyte: 1 M LiTFSI in DOL:TTE (1:1)<br>Electrolyte to sulfur ratio:16 µl/mg in coin cells	Initial capacity: ca 1500 mAh/g at C/10 (2nd cycle)<br>Capacity retention: ca 800 mAh/g after 80 cycles at C/10	[30]
TTE<br>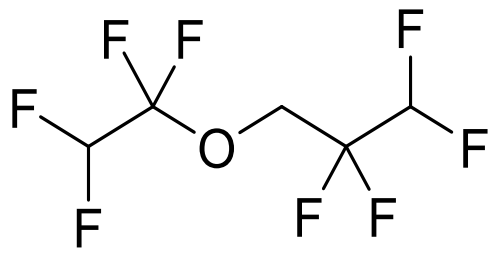	Sulfur content in sulfur electrode: 70 wt%<br>Sulfur loading: 2 mg cm^{-2}<br>Electrolyte: 1 M LiTFSI in DOL:DME:TTE (0.5:0.5:9)<br>Electrolyte to sulfur ratio: 10 µl/mg in coin cells	Initial capacity: ca 900 mAh/g at C/5 (2nd cycle)<br>Capacity retention: ca 900 mAh/g after 100 cycles at C/5	[31]
TTE<br>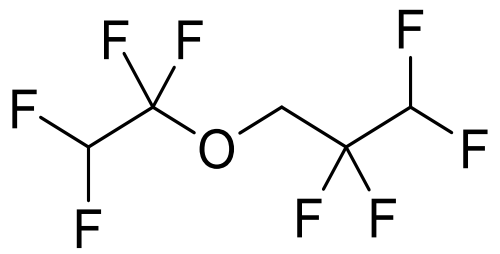	Sulfur content in sulfur electrode: 64 wt% (of which, 10 wt.% was Se)<br>Sulfur loading: 3.33 mg cm^{-2}<br>Electrolyte: 1 M LiTFSI +0.1M LiNO3 in DOL:TTE (1:1)<br>Electrolyte to sulfur ratio: 8 µl/mg in pouch cells	Initial capacity: ca 1300 mAh/g at C/8<br>Capacity retention: ca 1100 mAh/g after 30 cycles at C/8	[32]
TTE<br>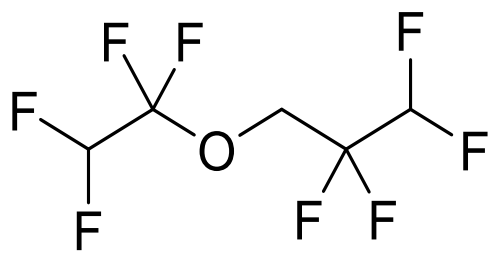	Sulfur content in sulfur electrode: 63 wt%<br>Sulfur loading: 6.6 mg cm^{-2}<br>Electrolyte: 1 M LiTFSI +0.3M $LiNO_3$ DME:DOL:TTE (2:2:1)<br>Electrolyte to sulfur ratio: 6.9 µl/mg in coin cells<br>Li electrode 50 µm thick	Initial capacity: ca 1100 mAh/g at C/10<br>Capacity retention: ca 800 mAh/g after 40 cycles at C/10	[33]

TTE 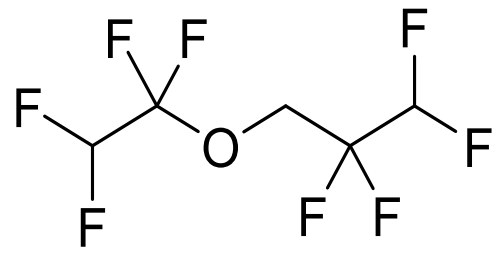	Sulfur content in sulfur electrode: 63 wt%<br>Sulfur loading: 4.3 mg cm^{-2}<br>Electrolyte: 1 M LiTFSI +0.3M $LiNO_3$ DME:DOL:TTE (2:2:1)<br>Electrolyte to sulfur ratio: 6.1 µl/mg in coin cells<br>Li electrode 50 µm thick	Initial capacity: ca 900 mAh/g at C/10<br>Capacity retention: ca 700 mAh/g after 146 cycles at C/10	[34]
TTE 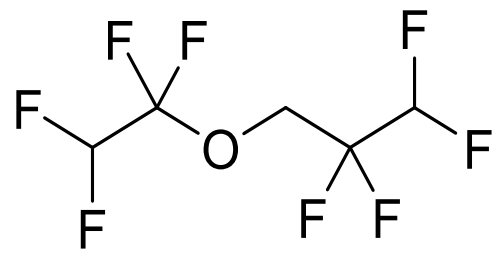	Sulfur content in sulfur electrode: 53 wt% (of which, 12.5 atomic % was Se)<br>Sulfur loading: 5.5 mg cm^{-2}<br>Electrolyte: 1 M LiTFSI DME:DOL:TTE (1:2:1)<br>Electrolyte to sulfur ratio: 9 µl/mg in coin cells	Initial capacity: ca 900 mAh/g at C/5<br>Capacity retention: ca 550 mAh/g after 50 cycles at C/5	[35]
BTFE 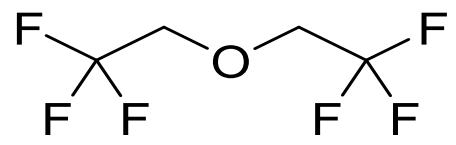	Sulfur content in sulfur electrode:56 wt%<br>Sulfur loading: 5 mg cm^{-2}<br>Electrolyte: 1 M LiTFSI in DOL/DME/ BTFE (1:1:2)<br>Electrolyte to sulfur ratio: 13 µl/mg in coin cells	Initial capacity: ca. 1200 mAh/g at C/10 at 45 °C<br>Capacity retention: ca 800 mAh/g after 10 cycles at C/10 at 45 °C	[36]
TFTFE 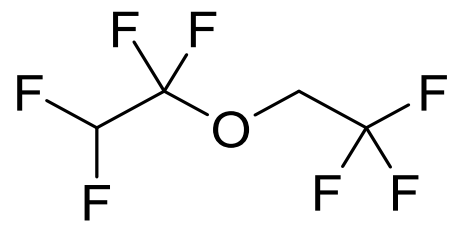	Sulfur content in sulfur electrode: 65 wt%<br>Sulfur loading: 1-1.2 mg cm^{-2}<br>Electrolyte: 1 M LiTFSI in DME/DOL/TFTFE (1:1:1)<br>Electrolyte to sulfur ratio: N/A	Initial capacity: ca 900 mAh/g at C/5 (3rd cycle)<br>Capacity retention: ca 700 mAh/g after 100 cycles at C/5	[37]
TFTFE 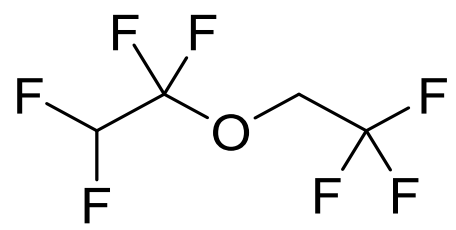	Sulfur content in sulfur electrode: 65 wt%<br>Sulfur loading: 2.5 mg cm^{-2}<br>Electrolyte: LiFSI:$LiNO_3$:DME:TFTFE in molar ratio of 1.9:1.0:18.5:3.8<br>Electrolyte to sulfur ratio: 2 µl/mg in pouch cells	Initial capacity: ca 900 mAh/g at C/20 (4th cycle)<br>Capacity retention: 600 mAh/g after 154 cycles at C/20	[38]
ETFE 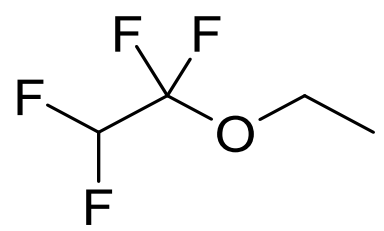	Sulfur content in sulfur electrode: 65 wt%<br>Sulfur loading: 1-1.2 mg cm^{-2}<br>Electrolyte: 1 M LiTFSI in DOL:ETFE (1:1)<br>Electrolyte to sulfur ratio: N/A	Initial capacity: ca 1000 mAh/g at C/5<br>Capacity retention: ca 700 mAh/g after 50 cycles at C/5	[39]

FDE<br>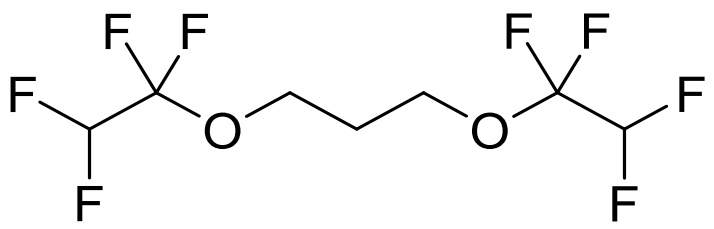	Sulfur content in sulfur electrode: 48 wt%<br>Sulfur loading: 1 mg cm^{-2}<br>Electrolyte: 1 M LiTFSI in DME/DOL/FDE (1:1:8)<br>Electrolyte to sulfur ratio: N/A	Initial capacity: ca 1200 mAh/g at C/2 (2nd cycle)<br>Capacity retention: ca 700 mAh/g after 200 cycles at C/2	40
TFEE<br>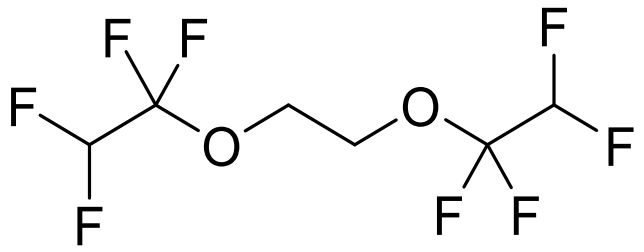	Sulfur content in sulfur electrode: 42 wt%<br>Sulfur loading: 2 mg cm^{-2}<br>Electrolyte: 1 M LiTFSI in DOL/TFEE (1:1)<br>Electrolyte to sulfur ratio: 12 µl/mg in coin cells	Initial capacity: ca 1200 mAh/g at C/10<br>Capacity retention: ca. 800 mAh/g after 100 cycles at C/10	41
TFEE<br>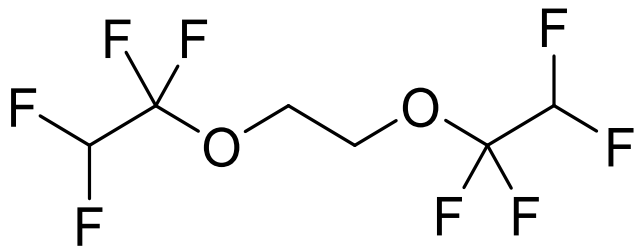	Sulfur content in sulfur electrode: 53wt%<br>Sulfur loading: 4 mg cm^{-2}<br>Electrolyte: 1 M LiTFSI in DOL/TFEE (1:1)<br>Electrolyte to sulfur ratio: 6.5 µl/mg in pouch cells	Initial capacity: ca. 1250 mAh/g at C/10<br>Capacity retention: ca. 800 mAh/g after 32 cycles at C/10	42
TPE<br>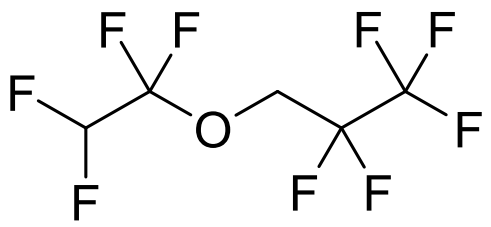	Sulfur content in sulfur electrode: 64 wt%<br>Sulfur loading: 1.2 mg cm^{-2}<br>Electrolyte: 0.5 M LiTFSI in DOL/TPE (1:2)<br>Electrolyte to sulfur ratio: 20 µl/mg in coin cells	Initial capacity: ca. 1000 mAh/g at C/10 (3rd cycle)<br>Capacity retention: ca 800 mAh/g after 50 cycles at C/10	43
F_3EO_1<br>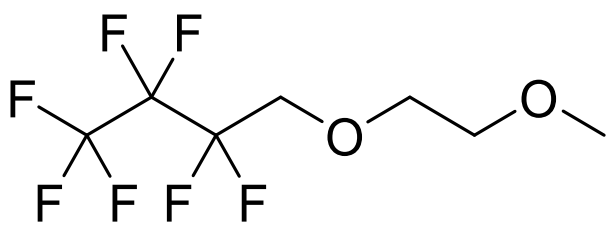	Sulfur content in sulfur electrode: 42 wt%<br>Sulfur loading: 1 mg cm^{-2}<br>Electrolyte: 0.5 M LiTFSI in F_3EO_1/TTE (1:5)<br>Electrolyte to sulfur ratio: N/A	Initial capacity: ca 1200 mAh/g at C/10 at 30°C (2nd cycle)<br>Capacity retention: ca 900 mAh/g after 100 cycles at C/10 at 30°C	44
TFEG<br>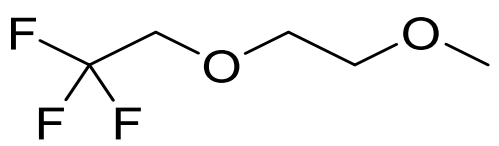	Sulfur content in sulfur electrode: 49 wt%<br>Sulfur loading: N/A<br>Electrolyte: 1 M LiTFSI in DME:TFEG (1:2)<br>Electrolyte to sulfur ratio: N/A	Initial capacity: ca 700 mAh/g at C/5 (3rd cycle)<br>Capacity retention: ca 400 mAh/g after 50 cycles at C/5	45
OFE<br>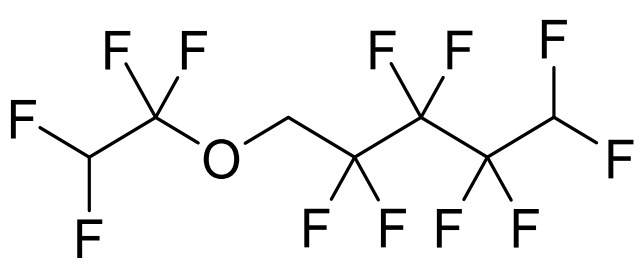	Sulfur content in sulfur electrode: 40 wt%<br>Sulfur loading: 1-2 mg cm^{-2}<br>Electrolyte: 1 M LiFSI in DME:OFE (95:5)<br>Electrolyte to sulfur ratio: N/A	Initial capacity: ca 1200 mAh/g at C/17 (3rd cycle)<br>Capacity retention: 800 mAh/g after 150 cycles at C/17	46

OFE<br>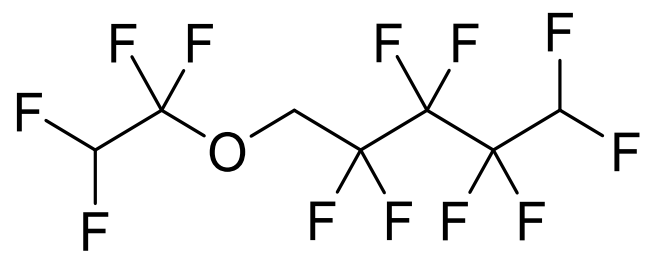	Sulfur content in sulfur electrode: 64 wt%<br>Sulfur loading: 4.5 mg cm^{-2}<br>Electrolyte: 1.5 M LiTFSI in DOL:OFE (1:1)<br>Electrolyte to sulfur ratio: 7 µl/mg in coin cells	Initial capacity: ca 1350 mAh/g at C/20<br>Capacity retention: ca 750 mAh/g after 100 cycles at C/10 (with 5 initial cycles at C/20)	[47]
F4DEE<br>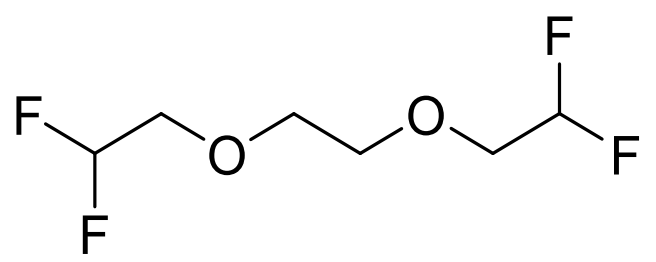	Sulfur content in sulfur electrode: 63% wt%<br>Sulfur loading: 1-2 mg cm^{-2}<br>Electrolyte: 1.2 M LiTFSI in F4DEE<br>Electrolyte to sulfur ratio: 15 µl/mg in coin cells	Initial capacity: ca 1000 mAh/g at C/10 (after formation at C/20)<br>Capacity retention: ca 600 mAh/g after ca. 50 cycles at C/5	[48]
FDMB<br>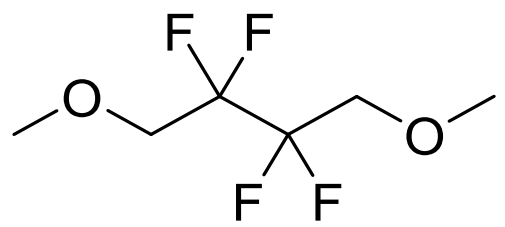	Sulfur content in sulfur electrode: 63 wt%<br>Sulfur loading: 5.3 mg cm^{-2}<br>Electrolyte: 1.2 M LiTFSI in F4DMB<br>Electrolyte to sulfur ratio: 7 µl/mg in coin cells<br>Li electrode 40 µm thick	Initial capacity: ca 1000 mAh/g at C/10 at 60 °C (after formation at C/20)<br>Capacity retention: ca 600 mAh/g after 20 cycles at C/10 at 60 °C	[48]
TFMP<br>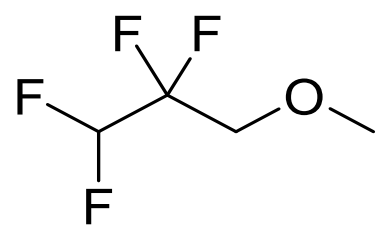	Sulfur content in sulfur electrode: **65 wt%**<br>Sulfur loading: **3 mg cm^{-2}**<br>Electrolyte: 1 M LiTFSI + 0.25 M $LiNO_3$ in DME:TFMP (3:2)<br>Electrolyte to sulfur ratio: **7 µl/mg in coin cells**	Initial capacity: ca 1000 mAh/g at C/5 (2nd cycle)<br>Capacity retention: **ca 800 mAh/g after 150 cycles at C/5**	**This work**

a: The electrolyte formulation and electrochemical results are provided for the best performing Li-S cell reported in each article. The testing temperature is room temperature or 25 °C, unless otherwise stated. The solvent ratios are in volume, unless otherwise stated. DOL: 1,3-Dioxolane, DME: 1,2-dimethoxyethane, TMS: tetramethylene sulfone, G2: diethylene glycol dimethyl ether; LiFSI: lithium bis(fluorosulfonyl)imide, LiTFSI: lithium bis(trifluoromethanesulfonyl)imide, TFTFE: 1,1,2,2-tetrafluoroethyl 2,2,2-trifluoroethyl ether, FDE: 1,3-(1,1,2,2-tetrafluoroethoxy)propane, TPE: 1,1,2,2-tetrafluoroethyl-2,2,3,3,3-pentafluoropropyl ether, OFE: 1H,1H,5H-octafluoropentyl-1,1,2,2-tetrafluoroethyl ether

Table S2. Overview of fluorinated ethers used as co-solvents to decrease the viscosity of sparingly solvating electrolytes.[a]

Fluorinated ether	Testing conditions	Electrochemical results	Reference
TTE 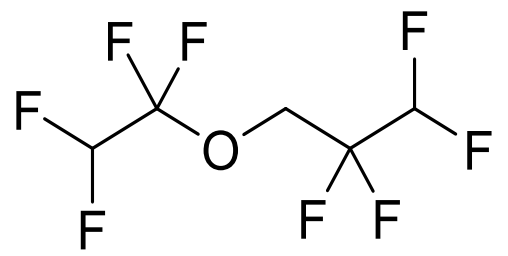	Sulfur content in sulfur electrode: 60 wt%<br>Sulfur loading: 0.6 mg cm^{-2}<br>Electrolyte: LiTFSI:G4:TTE in 1:1:4 molar ratio<br>Electrolyte to sulfur ratio: N/A	Initial capacity: ca 800 mAh/g at C/12<br>Capacity retention: ca 600 mAh/g after 50 cycles at C/12	[49]
TTE 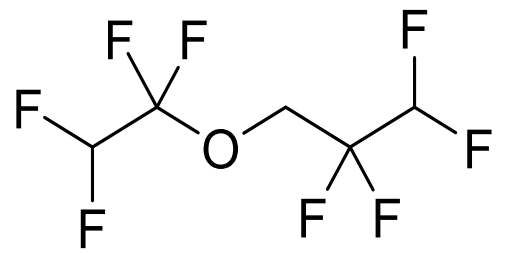	Sulfur content in sulfur electrode: 40 wt%<br>Sulfur loading: 1 mg cm^{-2}<br>Electrolyte: $(ACN)_2$-LiTFSI solvate mixed with TTE in 1:1 volume ratio<br>Electrolyte to sulfur ratio: N/A	Initial capacity: ca 1250 mAh/g at D/5, C/10<br>Capacity retention: ca 1050 mAh/g after 50 cycles at D/5, C/10	[50]
TTE 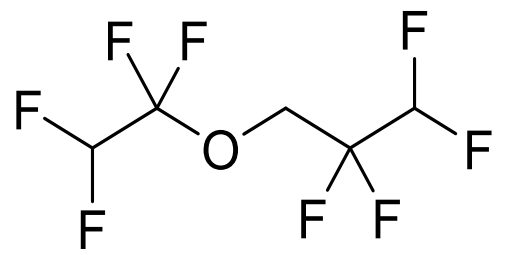	Sulfur content in sulfur electrode: 60 wt%<br>Sulfur loading: 1.9 mg cm^{-2}<br>Electrolyte: 1.5 M LiTFSI in TMS:TTE (1:1)<br>Electrolyte to sulfur ratio: 2.6 µl/mg in pouch cells	Initial capacity: ca 900 mAh/g at C/10 (2nd cycle)<br>Capacity retention: ca 400 mAh/g after 40 cycles at C/10	[51]
TTE 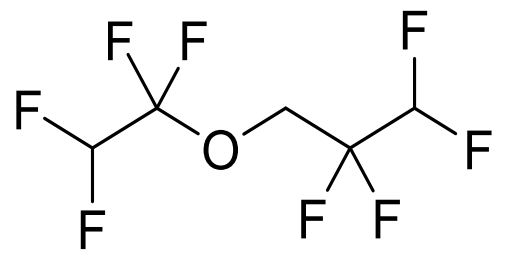	Sulfur content in sulfur electrode: 40 wt%<br>Sulfur loading: 0.2-0.4 mg cm^{-2}<br>Electrolyte: $(ACN)_2$-LiTFSI solvate mixed with TTE in 2:1 volume ratio<br>Electrolyte to sulfur ratio: 20 µl/mg in Swagelok cells	Initial capacity: ca 1100 mAh/g at C/10 (3rd cycle)<br>Capacity retention: ca 700 mAh/g after 100 cycles at C/10	[52]
TTE 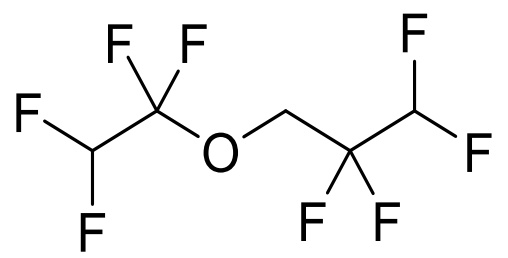	Sulfur content in sulfur electrode: 55 wt%<br>Sulfur loading: 2 mg cm^{-2}<br>Electrolyte: 0.4M LiTFSI-DEMETFSI : DOL:TTE 1:1:1 weight ratio<br>Electrolyte to sulfur ratio: 20 µl/mg in coin cells	Initial capacity: ca 900 mAh/g at C/10 (4th cycle)<br>Capacity retention: ca 750 mAh/g after 100 cycles at C/10	[53]
TTE 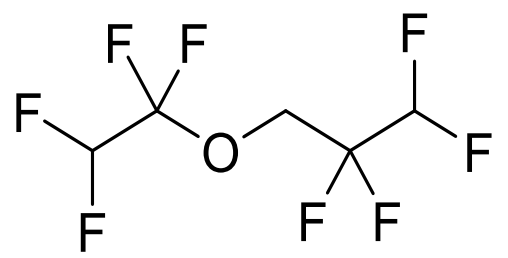	Sulfur content in sulfur electrode: 56 wt%<br>Sulfur loading: 1.8-2 mg cm^{-2}<br>Electrolyte: 0.4M LiTFSI-P1201TFSI : DOL:TTE 1:1:1 weight ratio<br>Electrolyte to sulfur ratio: 20 µl/mg in coin cells	Initial capacity: ca 750 mAh/g at C/5 (2nd cycle)<br>Capacity retention: ca 750 mAh/g after 100 cycles at C/5	[54]

TTE 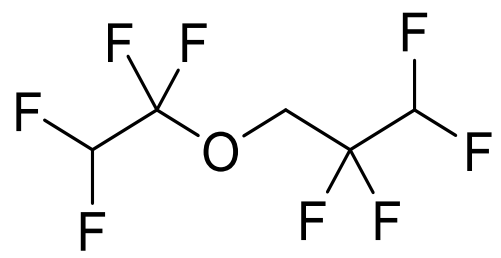	Sulfur content in sulfur electrode: 56 wt%<br>Sulfur loading: 2 mg cm^{-2}<br>Electrolyte: G2$_{0.8}$LiTFSI mixed with TTE in a 1:1 volume ratio.<br>Electrolyte to sulfur ratio: 5 µl/mg in coin cells	Initial capacity: ca 1000 mAh/g at C/5 (after formation cycle at C/20) at 55 °C<br>Capacity retention: ca 700 mAh/g after 100 cycles at C/5 at 55 °C	[55]
TTE 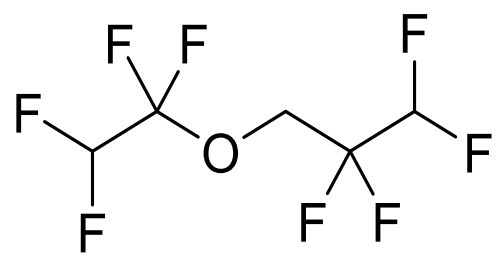	Sulfur content in sulfur electrode: 54 wt%<br>Sulfur loading: 1-1.2 mg cm^{-2}<br>Electrolyte: 5 M LiTFSI in G4 mixed with TTE in a 1:4 volume ratio.	Initial capacity: ca 1200 mAh/g at C/10<br>Capacity retention: ca 750 mAh/g after 200 cycles at C/10	[56]
TTE 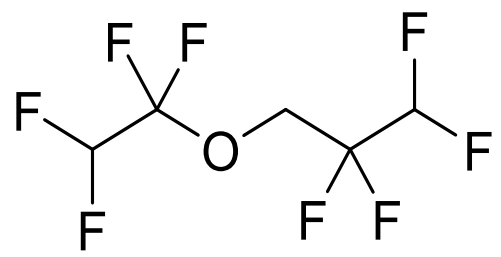	Sulfur content in sulfur electrode: 56 wt%<br>Sulfur loading: 3-3.4 mg cm^{-2}<br>Electrolyte: G2$_{0.8}$LiTFSI mixed with TTE in a 1:2 volume ratio<br>Electrolyte to sulfur ratio: 5 µl/mg in pouch cells<br>Li electrode 50 µm thick	Initial capacity: ca 1400 mAh/g in pouch cell<br>Capacity retention: ca 1400 mAh/g after 10 cycles in pouch cell	[57]
TTE 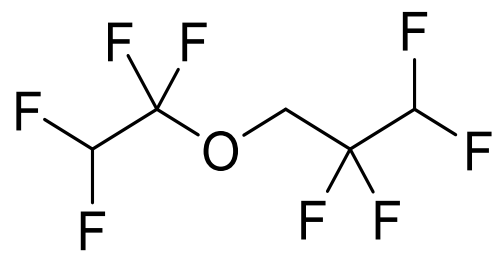	Sulfur content in sulfur electrode: 64 wt%<br>Sulfur loading: 5 mg cm^{-2}<br>Electrolyte: DME$_{1.6}$LiFSI mixed with TTE in a 1:2 volume ratio<br>Electrolyte to sulfur ratio: 2 µl/mg in coin cells	Initial capacity: ca 1500 mAh/g at C/20 at 30 °C<br>Capacity retention: ca 1000 mAh/g after 20 cycles at C/10 at 30 °C	[58]
TTE 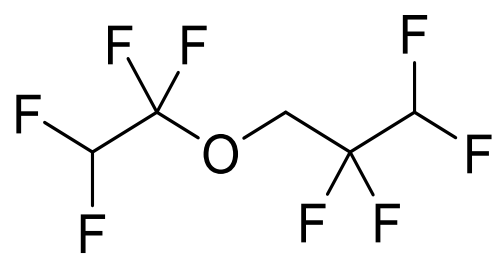	Sulfur content in sulfur electrode: 64 wt%<br>Sulfur loading: 4 mg cm^{-2}<br>Electrolyte: 2 M LiTFSI + 0.5M LiFSI in SL, mixed with TTE in 1:1.5 volume ratio<br>Electrolyte to sulfur ratio: 7 µl/mg in pouch cells	Initial capacity: ca 1250 mAh/g at C/10 (2nd cycle)<br>Capacity retention: ca 700 mAh/g after 100 cycles at C/10	[59]
TTE 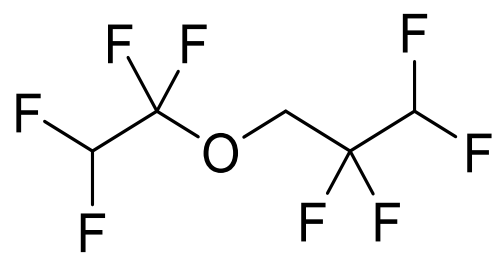	Sulfur content in sulfur electrode: 60 wt%<br>Sulfur loading: 3 mg cm^{-2}<br>Electrolyte: 1M LiTFSI + 10 mM DPDTe in EGDBE:TTE (3:2)<br>Electrolyte to sulfur ratio: 4 µl/mg in coin cells	Initial capacity: ca 800 mAh/g at C/20<br>Capacity retention: ca 500 mAh/g after 50 cycles at C/20	[60]

a: The electrolyte formulation and electrochemical results are provided for the best performing Li-S cell reported in each article. The testing temperature is room temperature or 25 °C, unless otherwise stated. The solvent ratios are in volume, unless otherwise stated. ACN: acetonitrile, TMS: tetramethylene sulfone, EGDBE: ethylene glycol dibutyl ether, SL: sulfolane, DPDTe: diphenyl ditelluride, DBE: dibutyl ether.

Table S3. Overview of rate capability testing of Li-S batteries with fluorinated ethers as co-solvents with non-fluorinated ethers.

Fluorinated ether	Testing conditions	Rate capability testing	Reference
TTE 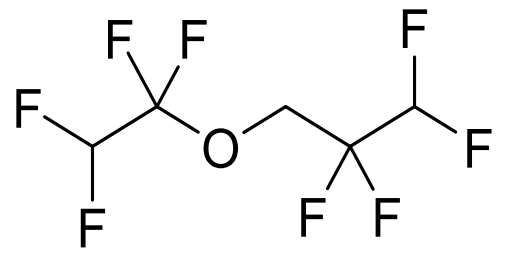	Sulfur content in sulfur electrode: 46 wt%<br>Sulfur loading: N/A<br>Electrolyte: 1 M LiTFSI in DOL/TTE (1:1)<br>Electrolyte to sulfur ratio: N/A	ca 900 mAh/g at C/3.3 (3rd cycle) and ca 600 mAh/g after 300 cycles at C/3.3<br>Benchmark 1M LiTFSI in DOL:DME (1:1): ca 900 mAh/g at C/3.3 (3rd cycle) and ca 200 mAh/g after 300 cycles at C/3.3	[26]
TTE 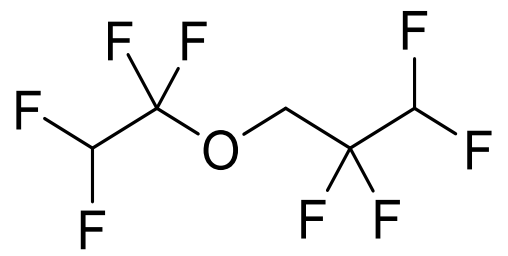	Sulfur content in sulfur electrode: 55 wt%<br>Sulfur loading: N/A<br>Electrolyte: 1 M LiFSI in DME/TTE (4:96)<br>Electrolyte to sulfur ratio: ca. 5.5 µl/mg in coin cells	ca 1200 mAh/g at C/17 (4th cycle), ca 1100 mAh/g at C/8.4, ca 900 mAh/g at C/4.2 ca 700 mAh/g at C/1.7, ca 500 mAh/g at C/0.84, ca 400 mAh/g at C/ 0.42	[27]
TTE 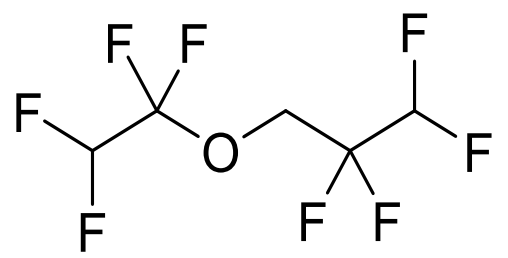	Sulfur content in sulfur electrode: 44 wt%<br>Sulfur loading: 2 mg cm^{-2}<br>Electrolyte: 1 M LiTFSI in DME/TTE (1:1)<br>Electrolyte to sulfur ratio: N/A	ca 1100 mAh/g at C/10, ca 1050 mAh/g at C/5, ca 900 mAh/g at C/2.5, ca 800 mAh/g at C/2, ca 600 mAh/g at 1C, ca 450 mAh/g at 2C	[28]
TFTFE 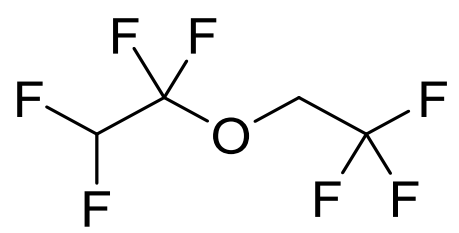	Sulfur content in sulfur electrode: 65 wt%<br>Sulfur loading: 1-1.2 mg cm^{-2}<br>Electrolyte: 1 M LiTFSI in DME/DOL/TFTFE (1:1:1)<br>Electrolyte to sulfur ratio: N/A	ca 1050 mAh/g at C/10 (2nd cycle), 900 mAh/g at C/5 (7th cycle), ca. 800 mAh/g at C/2 (12th cycle), ca. 700 mAh/g at C/1.33 (17th cycle)<br>Benchmark 1M LiTFSI in DOL:DME (1:1): ca 950 mAh/g at C/10 (2nd cycle), 800 mAh/g at C/5 (7th cycle), ca. 750 mAh/g at C/2 (12th cycle), ca. 700 mAh/g at C/1.33 (17th cycle)	[37]
ETFE 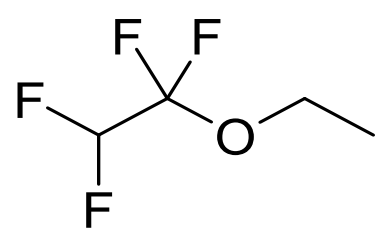	Sulfur content in sulfur electrode: 65 wt%<br>Sulfur loading: 1-1.2 mg cm^{-2}<br>Electrolyte: 1 M LiTFSI in DOL:ETFE (1:1)<br>Electrolyte to sulfur ratio: N/A	ca 1100 mAh/g at C/10 (2nd cycle), ca 800 mAh/g at C/5 (6th cycle), ca 600 mAh/g at C/2 (11th cycle), ca 500 mAh/g at C/1.75 (16th cycle)<br>Benchmark 1M LiTFSI in DOL:TEGDME (1:1):<br>ca 1000 mAh/g at C/10 (2nd cycle), ca 700 mAh/g at C/5 (6th cycle), ca 500 mAh/g at C/2 (11th cycle), ca 500 mAh/g at C/1.75 (16th cycle)	[39]

TFEE 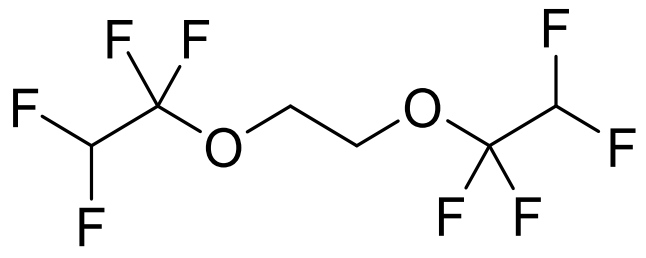	Sulfur content in sulfur electrode: 42 wt%<br>Sulfur loading: 2 mg cm^{-2}<br>Electrolyte: 1 M LiTFSI in DOL/TFEE (1:1)<br>Electrolyte to sulfur ratio: 12 µl/mg in coin cells	ca 1100 mAh/g at C/10 (2nd cycle), ca 900 mAh/g at C/5, ca 550 mAh/g at C/2, ca 400 mAh/g at 1C	[41]
FDMB 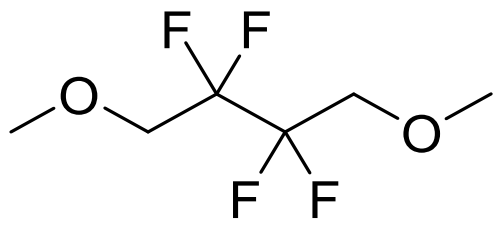	Sulfur content in sulfur electrode: 63% wt%<br>Sulfur loading: 1-2 mg cm^{-2}<br>Electrolyte: 1.2 M LiTFSI in F4DMB<br>Electrolyte to sulfur ratio: 15 µl/mg in coin cells	ca 1350 mAh/g at C/10 at 60 °C, ca 1050 mAh/g at C/5 at 60 °C, ca 700 mAh/g at C/2.5 at 60 °C, ca. 400 mAh/g at C/1.25 at 60 °C, ca 300 mAh/g at 1C at 60 °C	[48]
TFMP 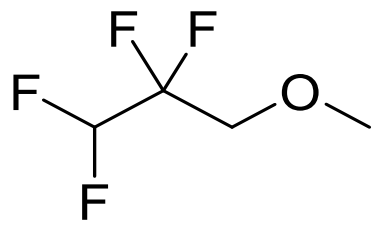<br>TFEP 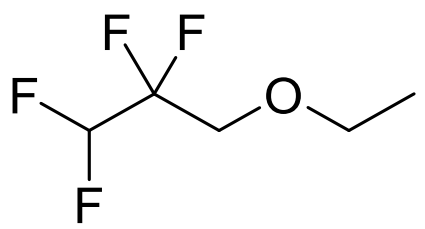	Sulfur content in sulfur electrode: **65% wt%**<br>Sulfur loading: **3 mg cm^{-2}**<br>Electrolyte: 1 M LiTFSI + 0.25M $LiNO_3$ in DOL:DME:FE (1:3:1), where FE is TFMP or TFEP<br>Electrolyte to sulfur ratio: **7 µl/mg in coin cells**	ca 1050 mAh/g at C/10 (2nd cycle), ca 1000 mAh/g at C/5, ca 900 mAh/g at C/2 and **ca. 800 mAh/g at 1C**<br><br>Benchmark (1M LiTFSI + 0.25 M $LiNO_3$ in DOL:DME 1:1): ca 1050 mAh/g at C/10, ca 900 mAh/g at C/5, ca 800 mAh/g at C/2 and ca. 200 mAh/g at 1C	**This work**

a: The electrolyte formulation and electrochemical results are provided for the best performing Li-S cell reported in each article. The testing temperature is room temperature or 25 °C, unless otherwise stated. The solvent ratios are in volume, unless otherwise stated.

S3. Additional experimental and computational results

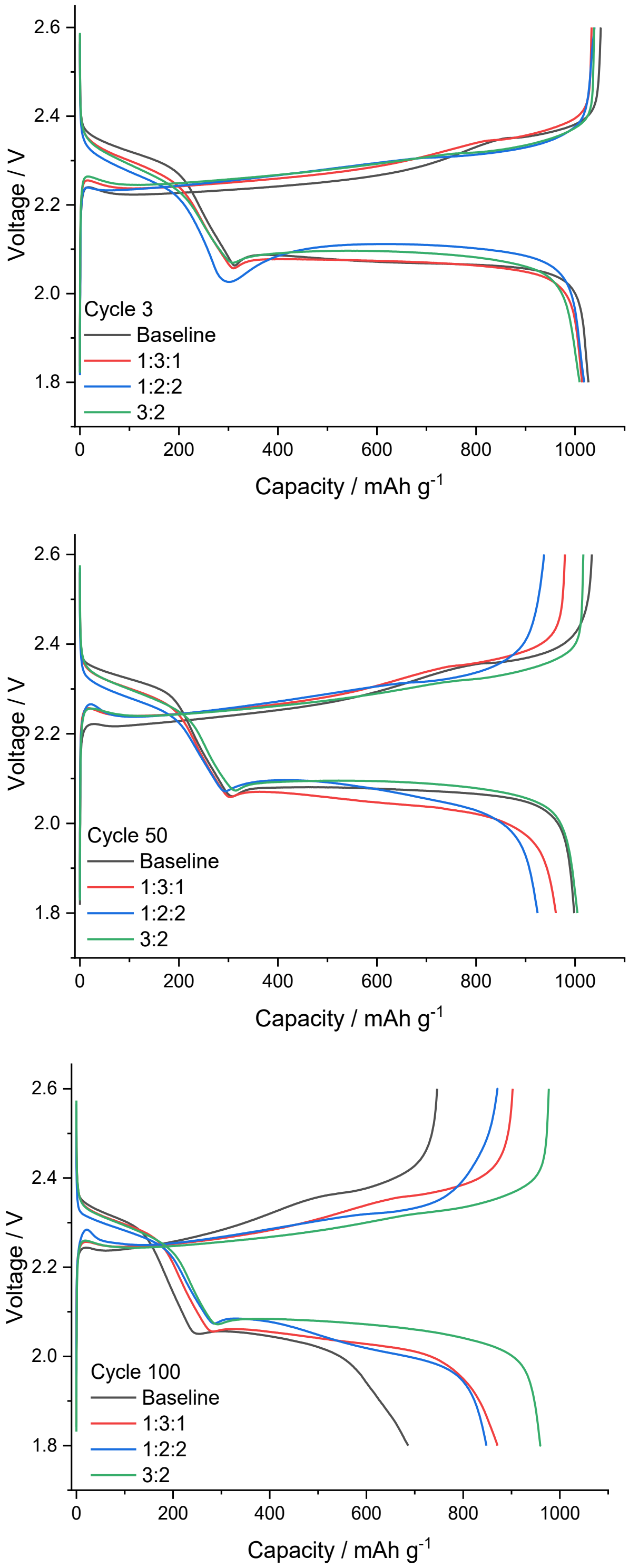

Figure S1. Improvement in the long-term stability of the voltage profiles of Li-S batteries via the incorporation of the fluorinated ether TFMP. The conditions are the same as in Figure 2 in the main article, with 1M LiTFSI + 0.25M $LiNO_3$ in DOL:DME:TFMP with solvent ratios of 0:3:2 (green), 1:3:1 (red) and 1:2:2 (blue), and with the benchmark electrolyte 1M LiTFSI + 0.25M $LiNO_3$ in DOL:DME 1:1 (black).

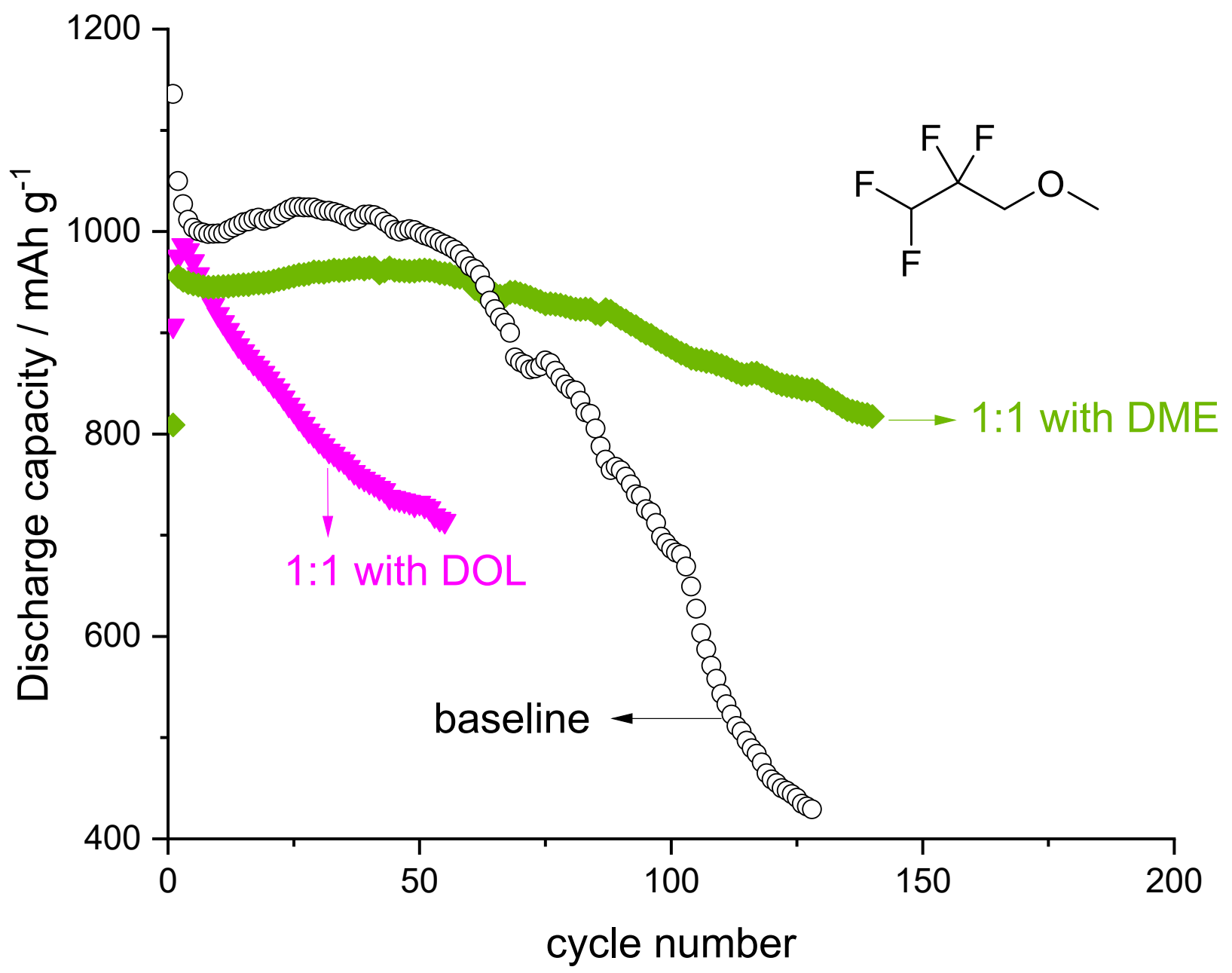

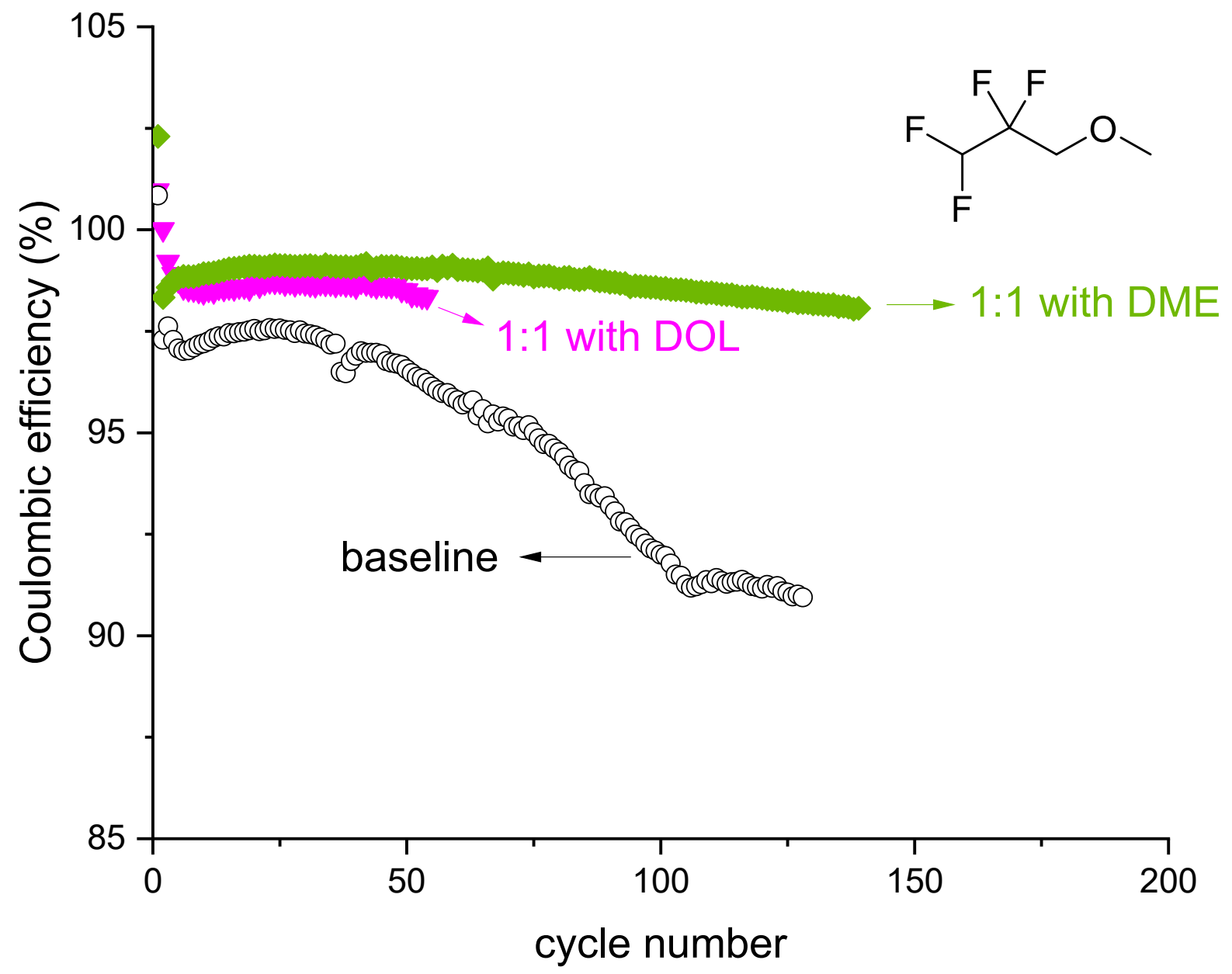

Figure S2. Compromised performance of Li-S battery with electrolyte formulations made of 1M LiTFSI + 0.25M $LiNO_3$ in either DOL:TFMP (magenta) or DME:TFMP (light green) with 1:1 solvent ratio. All other conditions as in Figure 2 in the main article. Note that a small amount of undissolved salt was present in the DOL:TFMP 1:1 formulation, meaning that the actual salt concentrations were slightly lower.

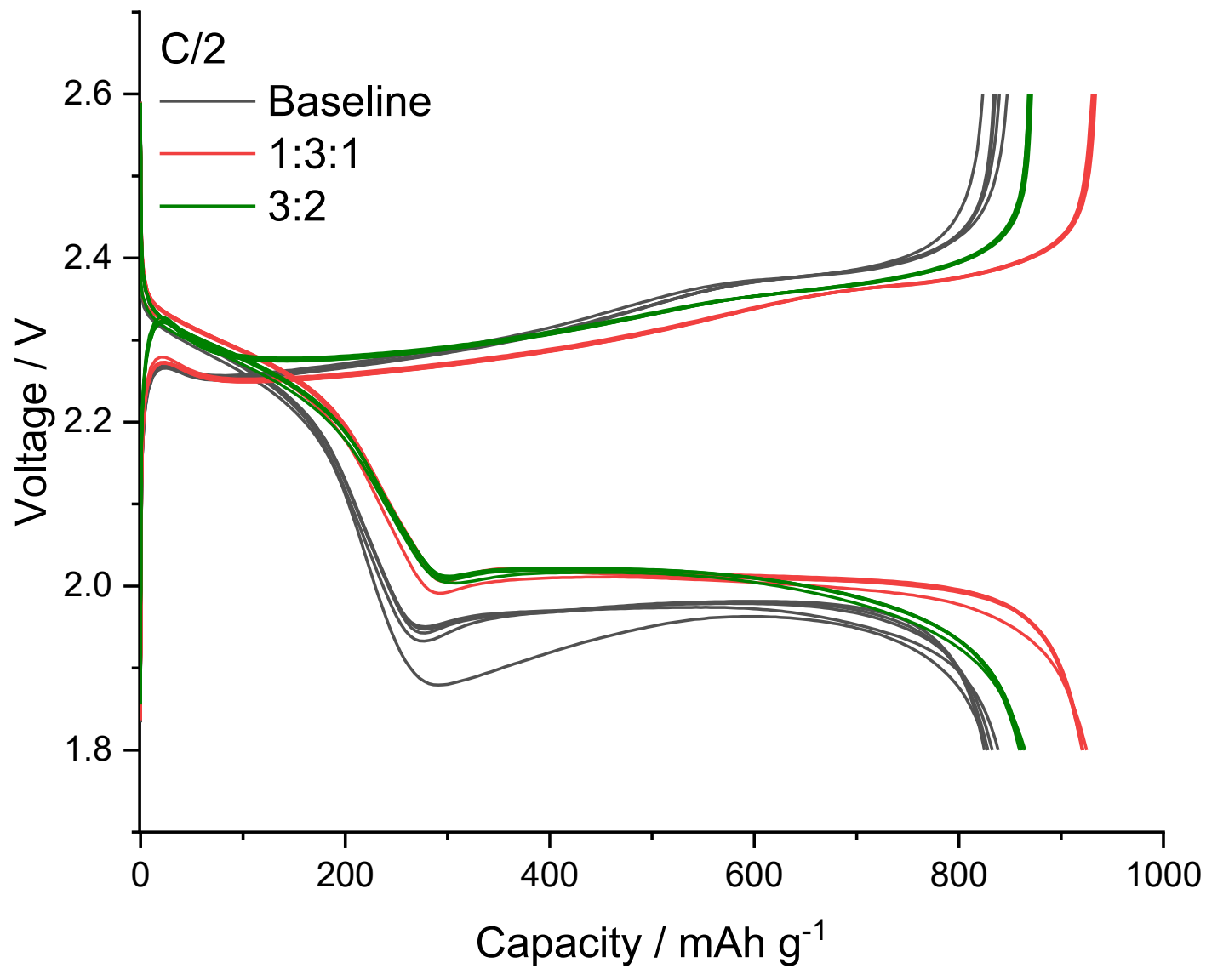

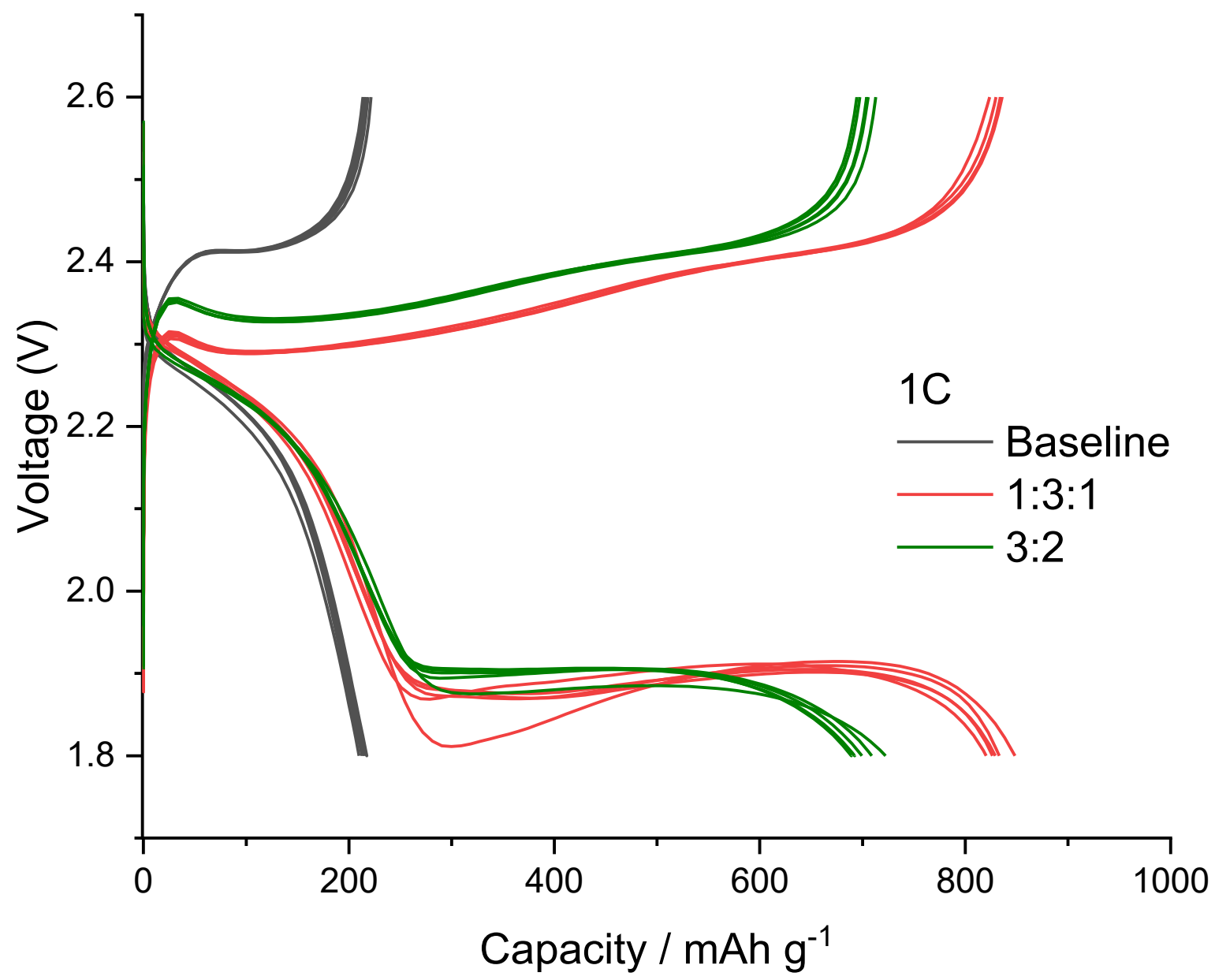

Figure S3. Improvement in the C-rate-dependent stability of the voltage profiles of Li-S batteries via the incorporation of the fluorinated ether TFMP. The conditions are the same as in Figure 3 in the main article, with 1M LiTFSI + 0.25M $LiNO_3$ in DOL:DME:TFMP with solvent ratios of 0:3:2 (green) and 1:3:1 (red), and with the benchmark electrolyte 1M LiTFSI + 0.25M $LiNO_3$ in DOL:DME 1:1 (black).

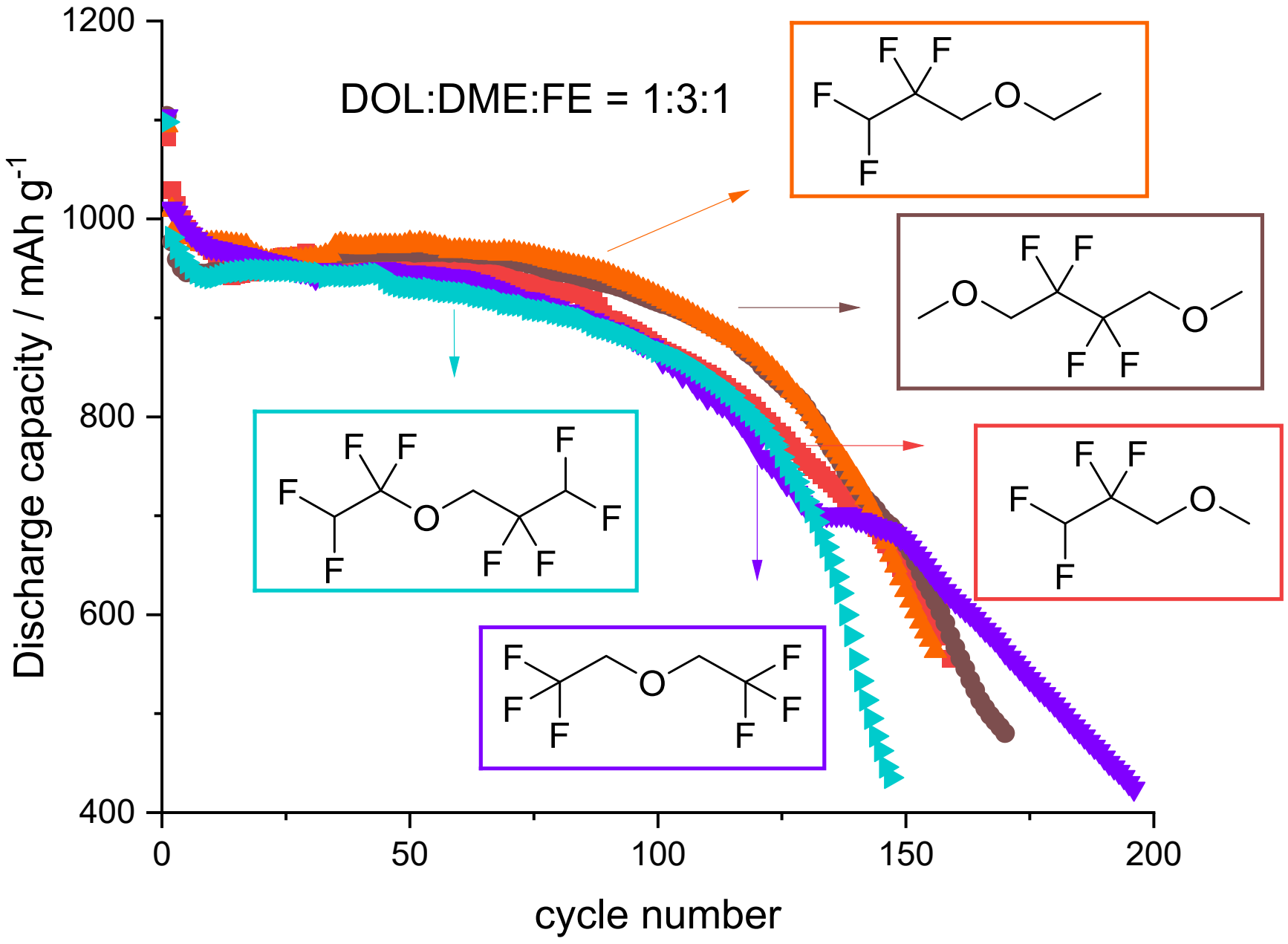

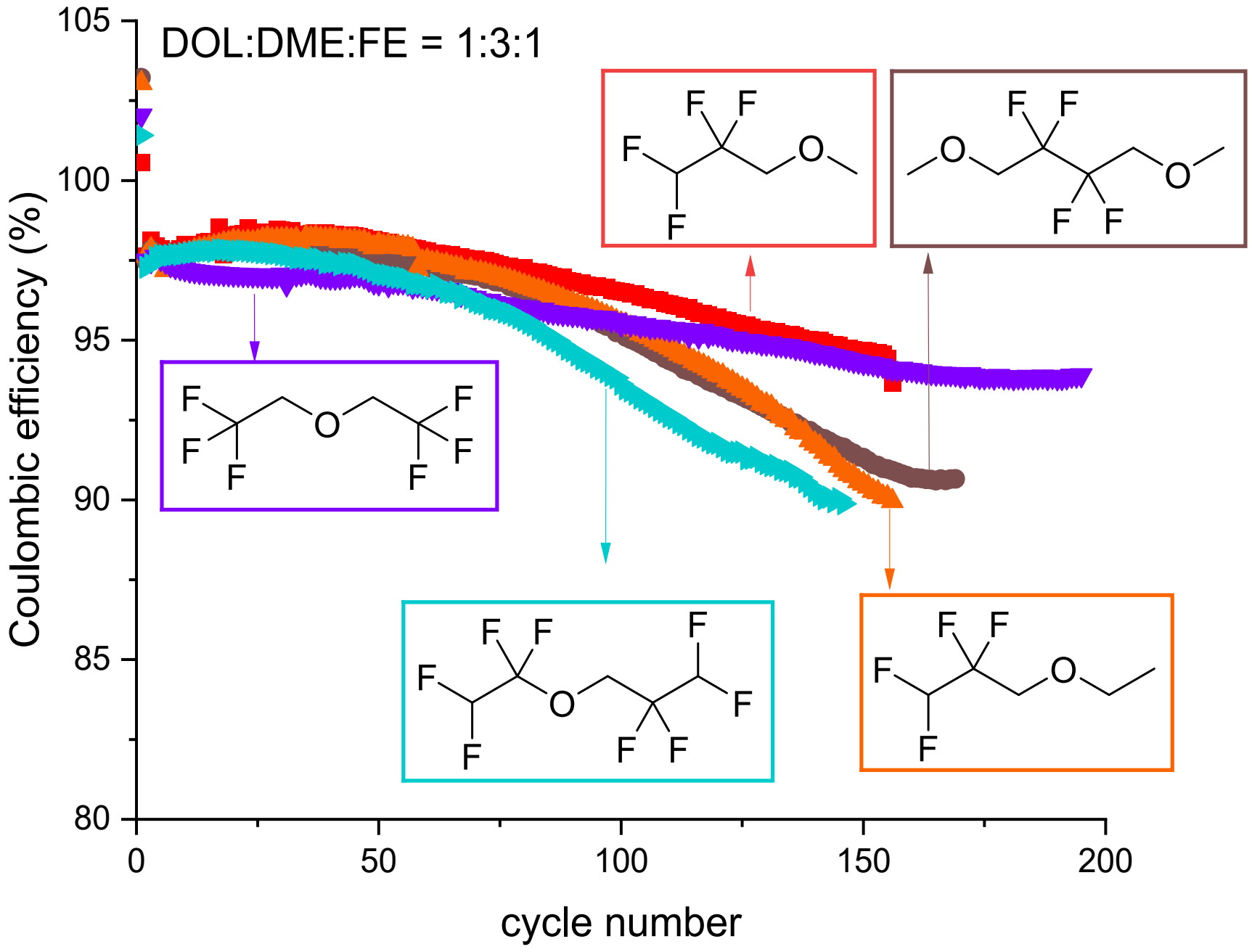

Figure S4. Effect of the fluorinated ether chemical formula on the long-term cycling of Li-S batteries, using electrolyte formulations with a moderate content of the fluorinated ether (20%), made of 1M LiTFSI + 0.25M $LiNO_3$ in DOL:DME:FE with solvent ratios of 1:3:1, where FE is the fluorinated ether, and their chemical structures are shown in the figure: TFMP (red), TFEP (orange), FDMB (brown), BTFE (purple), TTE (light blue).

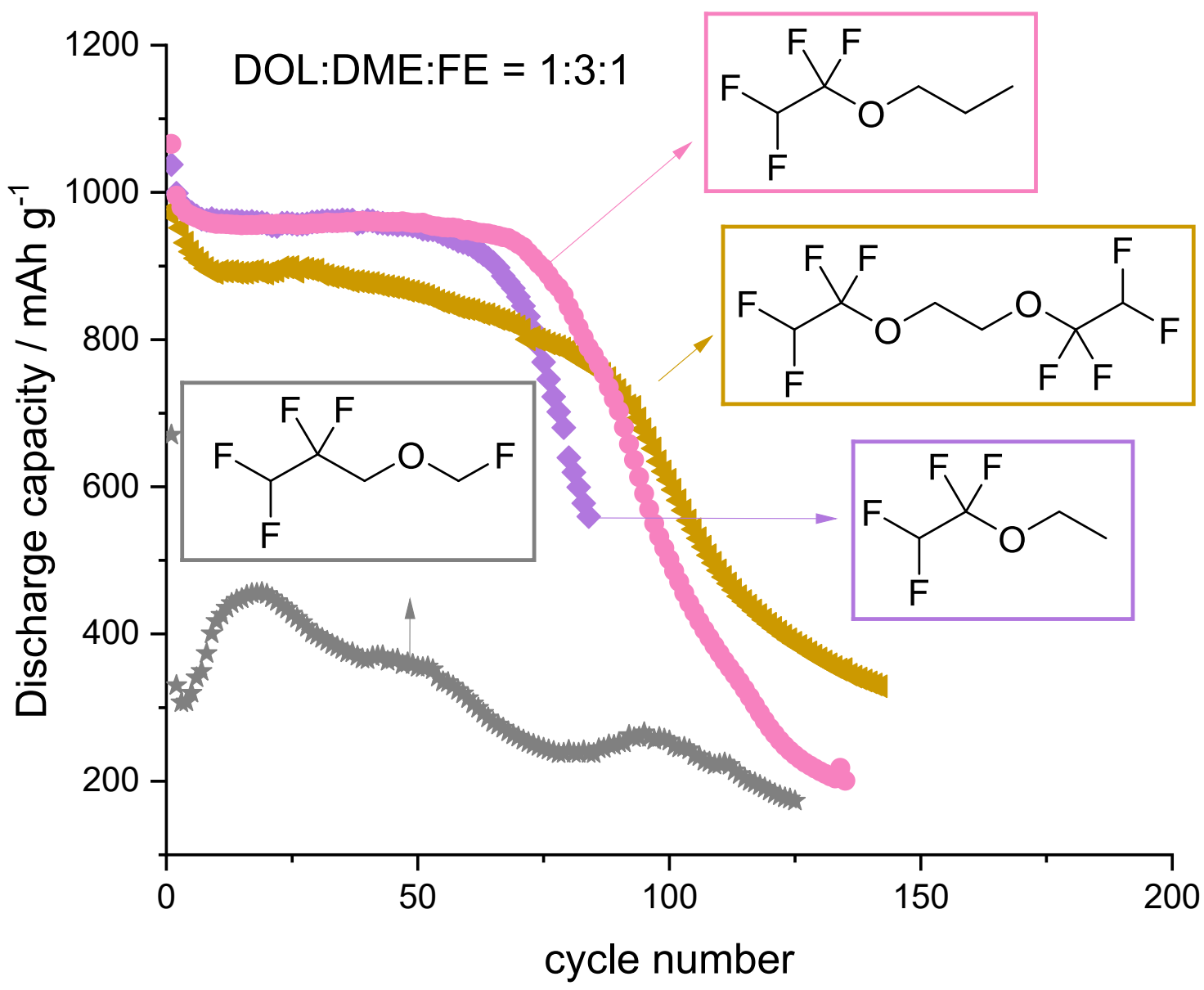

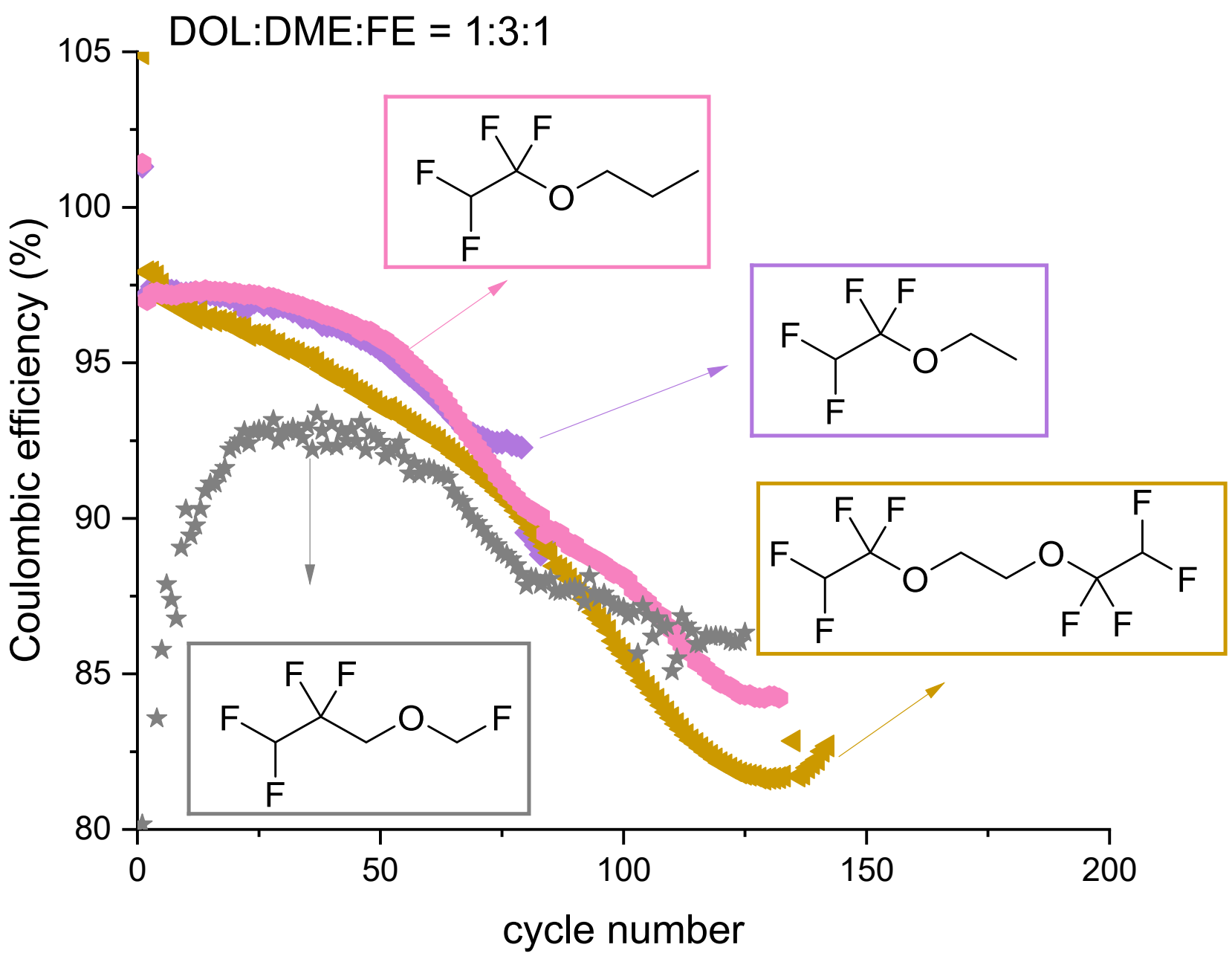

Figure S5. As in Figure S4 but with the following fluorinated ethers, whose chemical structures are shown in the figure: TFPP (pink), ETFE (lilac), TFEE (gold), TFFMP (grey).

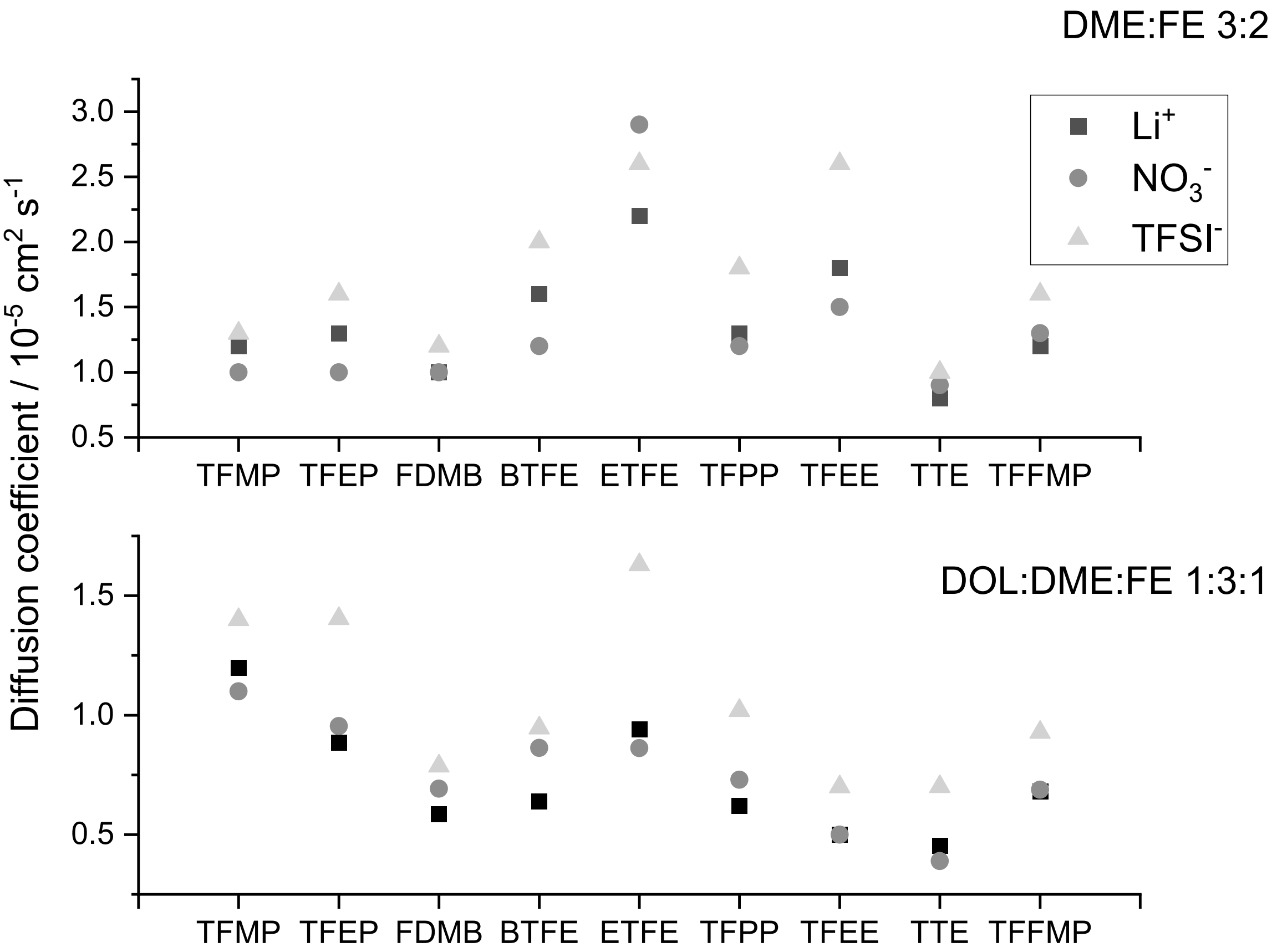

Figure S6. Influence of the chemical structure of fluorinated ethers on the diffusion coefficient of lithium ions (black squares), nitrate ions (dark grey circle) and TFSI ions (light grey triangles), as obtained from molecular dynamic simulations of 1M LiTFSI + 0.25M $LiNO_3$ in DOL:DME:FE with solvent ratios of 0:3:2 (upper panel) and 1:3:1 (lower panel), where FE stands for fluorinated ether, and their chemical structures are shown in Figure 1 in the main article.

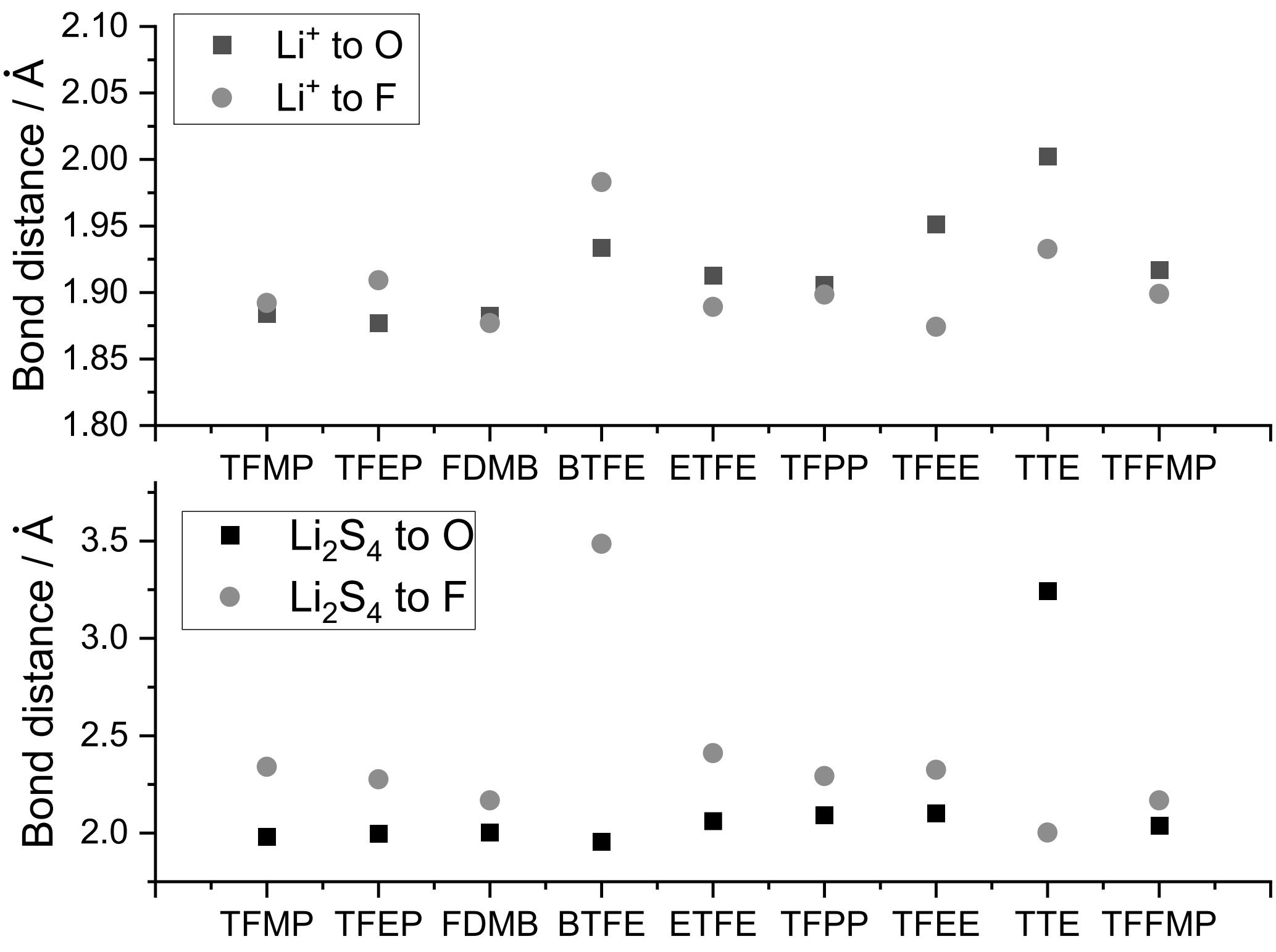

Figure S7. Influence of the chemical structure of fluorinated ethers on the bond distance between the closest F or O atom of the fluorinated ether and lithium ions or Li_2S_4 polysulfides.

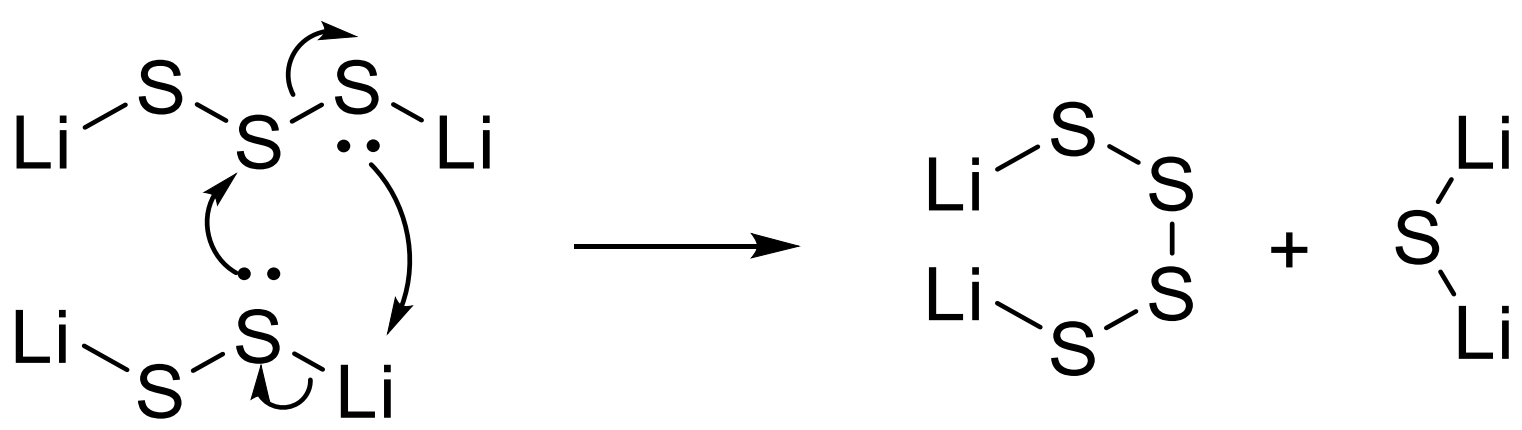

Figure S8. Illustration of the transfer of lithium ions between polysulfides as a result of polysulfide chemical reactions, using the reaction Li_2S_2+ Li_2S_3 → Li_2S_4 + Li_2S as an example.

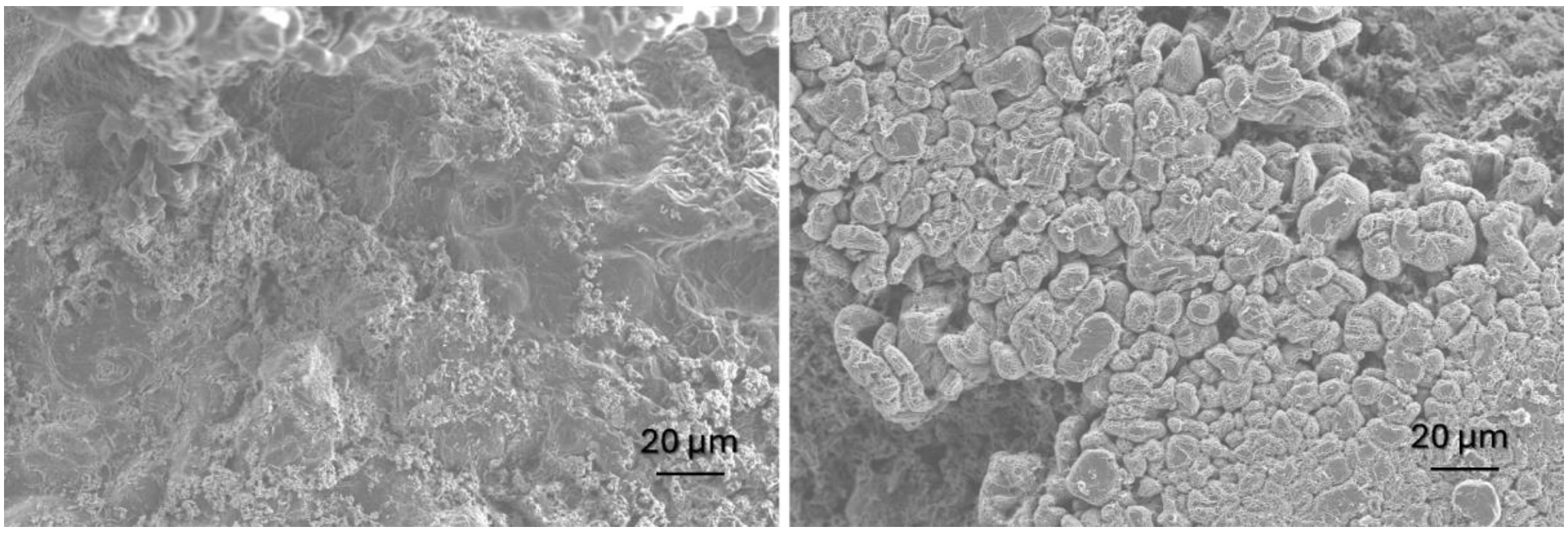

Figure S9. SEM characterization of cycled lithium electrodes in the baseline electrolyte (left) and TFMP electrolyte (right), after 50 cycles in Li-S cells.

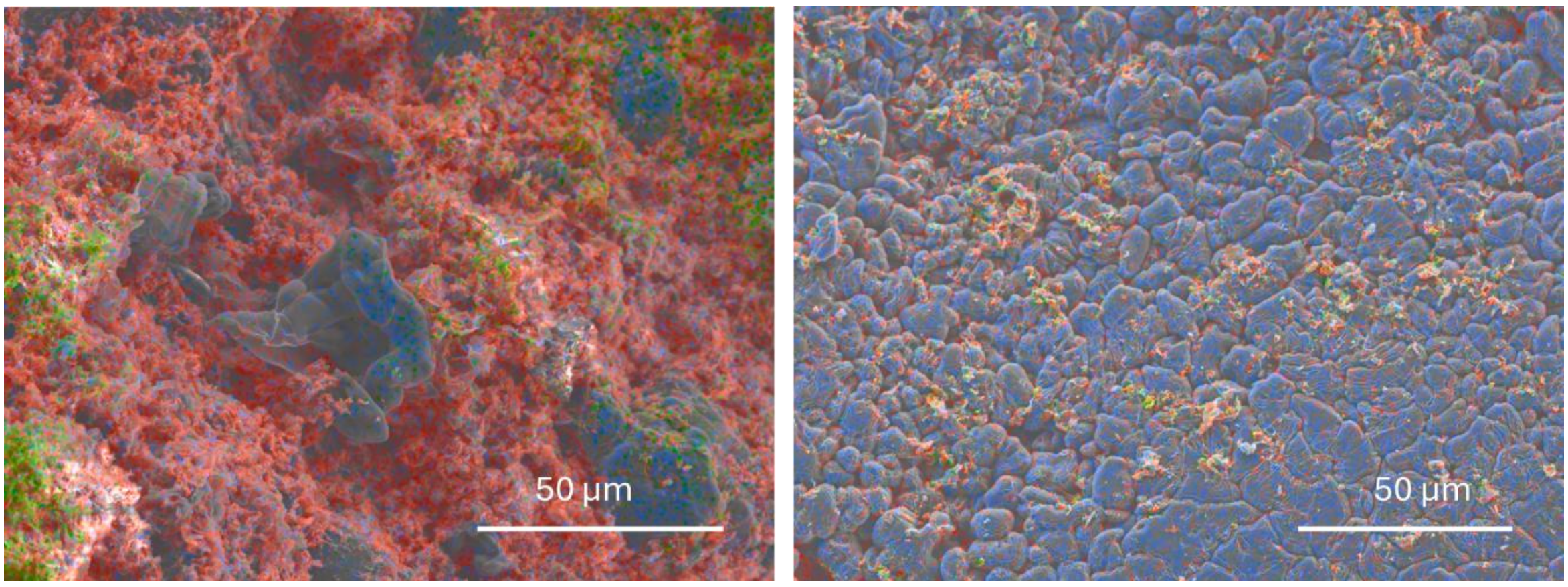

Figure S10. Coupled SEM+ EDX characterization of the lithium electrodes in Figure S9, cycled in the baseline electrolyte (left) and TFMP electrolyte (right) for 50 cycles in Li-S cells, where colors represent lithium (blue), fluorine (green) and sulfur (red).

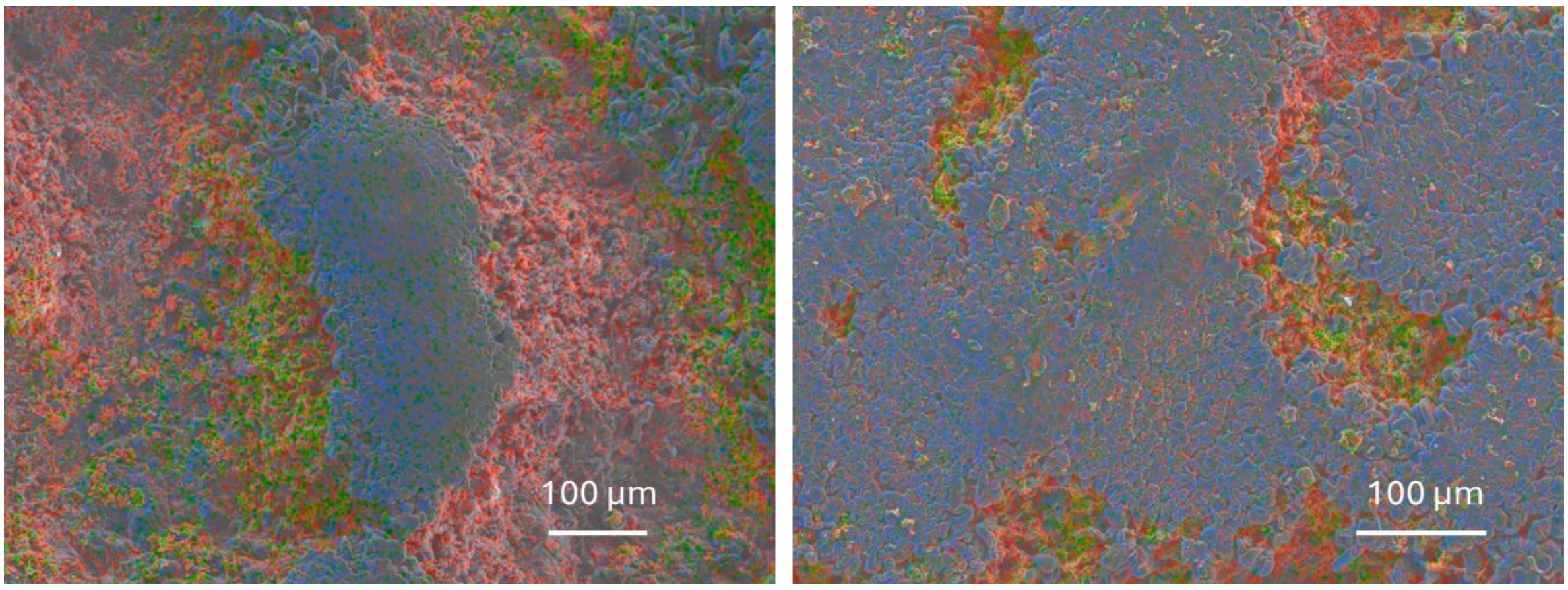

Figure S11. As in Figure S10 but imaging a larger electrode area.

Table S4. Average elemental composition of lithium electrodes, cycled in Li-S cells in the baseline and TFMP electrolytes, as indicated, and as obtained from *ex-situ* EDX characterization.

	5 cycles in baseline electrolyte	5 cycles in TFMP electrolyte	50 cycles in baseline electrolyte	50 cycles in TFMP electrolyte
Li	75.4 wt.%	78.2 wt.%	54.2 wt.%	72.8 wt.%
O	12.5 wt.%	12.5 wt.%	14.3 wt.%	16.5 wt.%
S	7.6 wt.%	6.2 wt.%	24.7 wt.%	7.2 wt.%
C	3.5 wt.%	1.8 wt.%	4.7 wt.%	1.9 wt.%
F	0.8 wt. %	1.2 wt.%	1.4 wt.%	1.3 wt.%
N	0.2 wt/%	0.1 wt. %	0.7 wt. %	0.3 wt.%

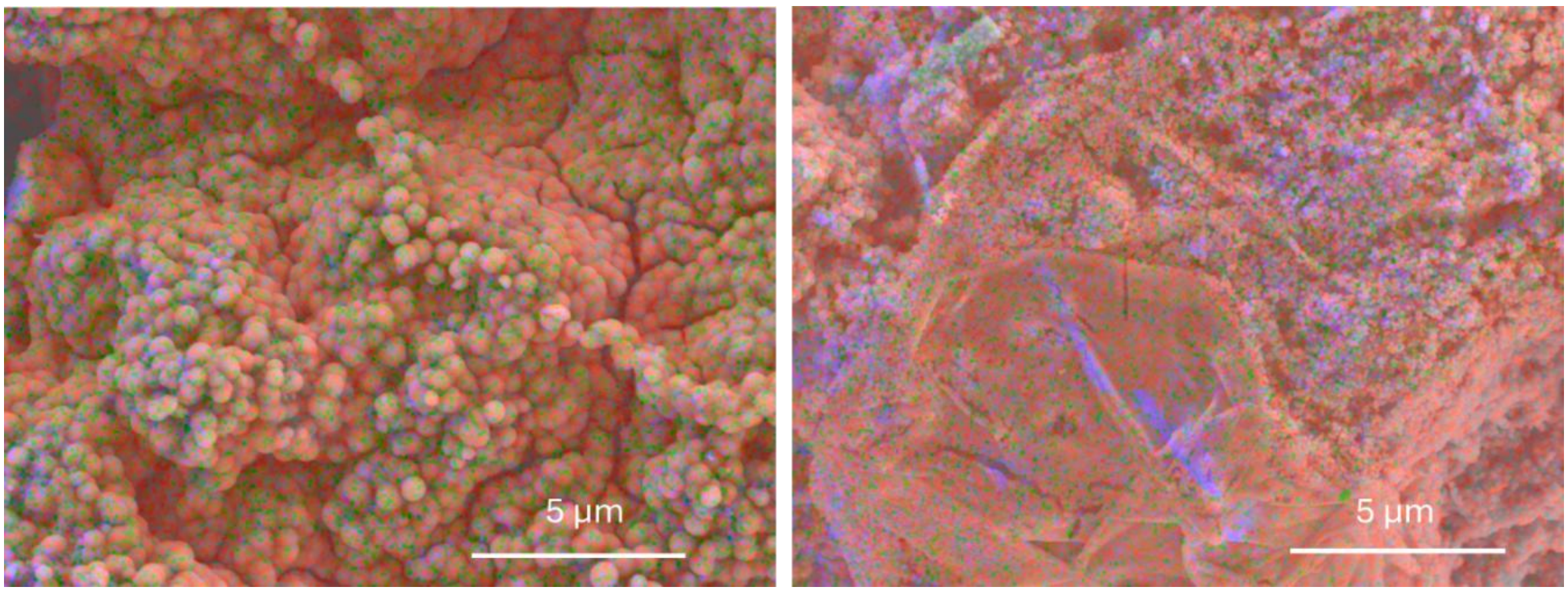

Figure S12. Coupled SEM+EDX characterization of discharged sulfur electrodes in the baseline electrolyte (left) and TFMP electrolyte (right), where electrodes have undergone a single half-cycle of discharge in Li-S cells, and where colors represent carbon (blue), fluorine (green) and sulfur (red).

Table S5. Average elemental composition of discharged sulfur electrodes, cycled in Li-S cells in the baseline and TFMP electrolytes, as indicated, and as obtained from *ex-situ* EDX characterization.

	1 half-cycle in baseline electrolyte	1 half-cycle in TFMP electrolyte	5 cycles in baseline electrolyte	5 cycles in TFMP electrolyte
S	69.8 wt.%	66.3 wt.%	61.0 wt.%	59.3 wt.%
C	16.8 wt.%	22.3 wt.%	24.6 wt.%	27.3 wt.%
O	9.5 wt.%	9.6 wt.%	10.4 wt.%	9.9 wt.%
F	3.1 wt.%	1.1 wt.%	3.2 wt.%	2.5 wt.%
N	0.7 wt. %	0.6 wt.%	0.8 wt.%	0.8 wt.%

S4. Discussion about the determination of transference numbers

The transport number of an ion is the fraction of the total current carried by that ion, but such quantity can only be measured when ions are fully dissociated. On the other hand, the transference number of an ion is the fraction of the total current carried through *any* species containing that ion. Specifically, the transference number measures the ratio between the net current associated with the movement of the ion, regardless of which compound containing the ions is producing the ion flux, with respect to the total current passed through the cell.[61,62]

For example, for a M^+A^- salt undergoing the following ion-association reactions:

$$M^+ + A^- \rightleftharpoons MA$$

$$M^+ + MA \rightleftharpoons M_2A^+$$

$$A^- + MA \rightleftharpoons MA_2^-$$

The transference number of the cation and anion (T_M and T_A, respectively) are related to the transport number of the individual species (t_i) as follows:[61]

$$T_M = t_{M^+} + 2t_{M_2A^+} - T_{MA_2^-} \quad (5)$$

$$T_A = t_{A^-} + 2t_{MA_2^-} - t_{M_2A^+} \quad (5)$$

Transference numbers can be evaluated using different techniques, including the steady-state current analysis method by Bruce and Vincent,[63] the low frequency impedance analysis by Roling and coworkers,[64] and the analysis of concentration and polarization cells by Newman and coworkers.[65] However, the applicability of these methods requires fast and stable interfacial charge-transfer reactions, absence of convection effects and non-linear deviations, and a clear separation of diffusional processes in the bulk electrolyte.[66–68]

An alternative approach is to evaluate the apparent transference number of an ion, which is defined as:[61]

$$t_{i,app} = \frac{z_i^2 D_i c_i}{\sum_j z_j^2 D_j c_j} \quad (7)$$

where z_i, D_i and c_i are the charge, diffusion coefficient and concentration of ion *i*. The diffusion coefficients, D_i, can be determined, for example, from radiotracer or pulsed field gradient NMR experiments. Note that these measurements are sensitive to the motion of charged and neutral compounds containing the ion under consideration. Therefore, for the M^+A^- salt undergoing the ion-association reactions described above, the experimental diffusion coefficients of the cation and anion are given by: [61]

$$D'_M = \alpha_1 D_{M^+} + \alpha_3 D_{MA} + \alpha_4 D_{M_2X^+} + \alpha_5 D_{MX_2^-} \quad (8)$$

$$D'_A = \alpha_2 D_{A^-} + \alpha_3 D_{MA} + \alpha_4 D_{M_2X^+} + \alpha_5 D_{MX_2^-} \quad (9)$$

where α_1 is the fraction of dissociated cations M^+, α_2 is the fraction of dissociated anions A^-, α_3 is the fraction of M^+A^- ion pairs, α_4 is the fraction of M_2A^+ triple ion pairs and α_5 is the fraction of MA_2^- triple ion pairs. The equations above show that when ion coordination is strong, and thus the fraction of dissociated cations and anions without ion pairing is small,

then the experimental diffusion coefficient of the cation and anion, D'_M and D'_A, become very similar and, as a result, the apparent transference numbers become close to 0.5.

Here, we evaluate the apparent transference numbers using MD simulations, where the diffusion coefficients are obtained from the mean square displacements, as described in section S1.3. Although transference numbers and apparent transference numbers are different quantities, as described above, they both reflect the relationship between the rate of transport of the ion under consideration with respect to the rate of transport of all ions present in the electrolyte. Therefore, the trends observed in the apparent transference number are expected to also hold in the values of the transference number.

Table S6 provides compilation of lithium (apparent) transference numbers reported in the literature for related liquid electrolyte systems with (fluorinated) ethers. The variations in the magnitude of the (apparent) transference number as the electrolyte composition was varied were found to be consistent across the different methods of determination. However, the magnitude of the change of the apparent transference number was smaller than that of the transference number, as the former tends towards a value close to 0.5 as ion association increases, for the reasons explained above.

Theoretical considerations have shown that even moderate improvements in lithium transference number can translate into major improvements in the performance of lithium-ion batteries.[69,70] In this work, we show that rather small changes in the apparent lithium transference number are correlated with significant performance improvements in both the Li metal anode and Li-S batteries.

Table S6. Comparison of literature values of lithium (apparent) transference numbers determined by different approaches for related liquid electrolyte systems with (fluorinated) ethers. Note that trends observed when changing the electrolyte formulation hold across the different methods of determination of (apparent) transference numbers.

Electrolyte	MD simulations	Pulse field gradient NMR	Bruce-Vincent method	Impedance analysis	Ref
1M LiTFSI in TFEE:DME 1:1	0.45	0.48	0.29	0.25	[68]
1M LiTFSI in TFEE:DOL 1:1	0.50	0.51	0.74	0.65	
1M LiFSI in DME		0.44	0.397		[71]
1M LiFSI in EME		0.42	0.495		
1M LiFSI in MPE		0.39	0.470		
1M LiFSI in DEE		0.47	0.525		
4.8M LiFSI in DME	0.49	0.47			[72]
3.5 M LiFSI in DME:TTE 2.8:1	0.46	0.48			
2.8 M LiFSI in DME:TTE 1.4:1	0.43	0.47			
2 M LiFSI in DME:TTE 0.7:1	0.47	0.45			
1.2 M LiFSI in DME:TTE 0.3:1	0.53	0.49			
1 M LiFSI in MME-FTriEG	0.506	0.54			[73]
1M LiFSI in DME-FTriEG	0.497	0.44			
1M LiFSI in tetraglyme	0.421	0.4			
4.2M LiTFSI in SL		0.59	0.53		[74]
3.2M LiTFSI in SL:TTE 3:1		0.47	0.43		
3.2M LiTFSI in SL:TTE 1.6:1		0.47	0.39		
3.2M LiTFSI in SL:TTE 0.8:1		0.44	0.38		
1 M LiFSI DOL/DME	0.333		0.314		[75]
1 M LiFSI DEE	0.511		0.512		

S5. Calculations of Sand's time and diffusion limited current

In constant current experiments, the time at which the concentration of the reactant drops to zero at the electrode surface (at which, then, the reaction would stop) is called Sand's time:[76,77]

$$t_{Sand} = \pi D_{app} \left(\frac{Fc_0}{2 j t_a} \right)^2 \tag{10}$$

where D_{app} is the apparent diffusion coefficient (known also as ambipolar diffusion coefficient), F is Faraday's constant, c_0 is the bulk concentration of the reactant, j is the current density and t_a is the anion transference number (where the equation above is for a reaction that consumes a monovalent cation, such as $Li^+ + e^- \rightarrow Li$, and an electrolyte that contains a monovalent anion, such $TFSI^-$).

Using typical values of D_{app} ~10^{-6} cm^2/s, and t_a~0.6 for Li-ion electrolytes,[77] for c_0=1.25 M and a current density of 1 mA/cm^2 (corresponding to a C-rate of C/5 in the Li-S batteries with 3 mg/cm^2 sulfur loading used here) the Sand time is of ca. 9 hours.

In addition, the depletion of the reactant concentration at Sand's time will only happen if the applied current is higher than the limiting current given by: [76,77]

$$j_{lim} = \frac{2c_0 F D_{app}}{t_a L} \tag{11}$$

where L is the distance between the two electrodes. This is because the monovalent cations (Li^+), that are consumed at one electrode ($Li^+ + e^- \rightarrow Li$) are also generated at the other electrode (e.g. $\frac{1}{2} Li_2S_x \rightarrow \frac{1}{2} S_x + Li^+ + e^-$ for Li-S cells and $Li \rightarrow Li^+ + e^-$ for Li-Li symmetrical cells) and thus the maximum concentration gradient that can be built inside the electrolyte is $2c_0/L$.

For Li-S cells, due to the short distance between the electrodes (given by the thickness of two Celgard separators, ca. 40 µm), the limiting current is very large, ca. 100 mA cm^{-2}. Smaller values of limiting current are obtained for the *operando* NMR measurements in Li-Li symmetrical cells, in which the separation between electrodes is bigger (ca. 400 µm), but still the values are large (ca 10 mA cm^{-2}), compared to the applied current (1 mA cm^{-2}).

In conclusion, the above evaluation of the limiting current values demonstrates that the experimental conditions used here will not trigger the depletion of the lithium concentration at the electrode surface due to the build-up of concentration gradients across the bulk electrolyte contained within the separator. However, the process of lithium plating/stripping induces significant changes in the electrode's morphologies that form a tortuous path for lithium-ion transport, thus resulting in additional diffusion limitations.[78] Phase field simulations, among others, can be used to understand the interplay between kinetic and ion transport limitations affecting lithium electrodeposition, although the determination of some of the input parameters for the model is also complex and could be the subject of further work.[79–81]

S6. Discussion of the combined impedance and *operando* ^{7}Li NMR characterization of lithium metal anode reactions

The combination of impedance and *operando* ^{7}Li NMR measurements of the lithium metal anode reactions in lithium symmetrical cells (Figure S13, which is Figure 11 in the main article) provide a wealth of information,[5,6] and thus, they are discussed in this section in more detail.

In the electrolyte containing the best performing fluorinated ether (TFMP, Figure S13, left panel) a nearly continuous increase in the intensity of the NMR lithium metal microstructure signal is observed up to a lithium plating capacity of ca. 15 mA h cm^{-2}, after which, a reversible short-circuit is observed. In contrast, in the electrolytes containing the other fluorinated ethers, the plating capacities before the cell shorting were much smaller, of ca. 6 and 10 mA h cm^{-2} for TTE and TFPP, respectively (Figure S13, middle and right panels, respectively), and the short-circuits lead to permanent cell failure.

Close inspection of the evolution of the magnitude of the cell impedance at a low frequency of 9 Hz (Figure S14), as obtained from impedance measurements performed every hour during the *operando* ^{7}Li NMR measurements, shows that the cell short-circuit with TFMP is soft, since the associated magnitude of the cell impedance (ca. 20 Ω cm^2) is not small, as would be expected for a hard short (note that an upper limit of 10 Ω cm^2 has been proposed to define hard shorts[82]). In contrast, with TTE, although a soft short-circuit happens after 6 hours of plating (as identified from the plateauing in the growth of lithium microstructures detected by NMR), it is followed by a hard short-circuit (evidenced by a marked impedance drop, where the magnitude of the cell impedance becomes very small, ca. 1 Ω cm^2). Finally, with TFPP, there is a period (between 6 and 10 hours of plating) in which the cell is intermittently soft short-circuited (evidenced by the very frequent voltage drops, and the deceleration of the growth of the lithium microstructure NMR signal), after which the cell short circuit becomes hard and the cell impedance drops (to ca. 5 Ω cm^2). The occurrence of hard and irrecoverable short-circuits in the presence of TTE and TFPP suggests a much higher spatial inhomogeneity in the electrode reactions, compared to TFMP, in agreement with the discussion in Section 2.2 in the main article. Note that the repetitive voltage drops, seen in all electrolytes every hour, are due to the interruption of the current to perform the impedance measurements, in which the cell voltage relaxes to close to zero, as expected, since the two electrodes in the cell are lithium electrodes and hence their potentials are the same in the absence of an applied current.

Close inspection of the evolution of the cell voltage at the beginning of the plating experiments (Figure S15) shows that, with TFPP, the value of the cell voltage decreases rapidly as the plating experiment progresses, whereas the decay is slower with TTE, and TFMP shows the slowest decay. Such decrease in cell voltage can be ascribed to an increase in electrode area resulting from the growth of lithium microstructures (e.g. dendrites/mossy lithium), as the lithium plating overpotential decreases when the current is distributed over a larger electrode area.[83] Therefore, the faster voltage decay with TFPP, and to a lesser extent with TTE, can be ascribed to a faster growth of lithium microstructures, in agreement with the earlier appearance of a cell short-circuit with these fluorinated ethers. Indeed, the impedance results reveal that the magnitude of the imaginary component of the impedance in TFPP and TTE electrolytes is smaller than in TFMP, and significantly decreases as plating

progresses (see Figure S16), which can also be ascribed to a growth of the lithium surface area (as the impedance of a capacitor is given by $Z=-i/\omega C$,[84] where i is the imaginary number, ω is the angular frequency and C is the capacitance, and the capacitance of a capacitor increases with the surface area[85]). On the other hand, with the short-circuited cells (for example, after 20 hours of plating, see Figure S16) the impedance, in the 500 Hz-0.8 Hz frequency range used here, is dominated by the resistance of the short, and any capacitance contributions becomes difficult to discern.

It is also worth mentioning that the frequent voltage jumps seen with TFPP indicate poor lithium SEI stability.[5] The voltage fluctuations are likely a consequence of the complex reaction dynamics that occur in the presence of an unstable SEI; for example, fast dissolution of lithium microstructures can occur due to corrosion reactions, promoted by electrolyte reduction, and exacerbated by a non-protective SEI,[86] thus resulting in short-lived cell shorts.

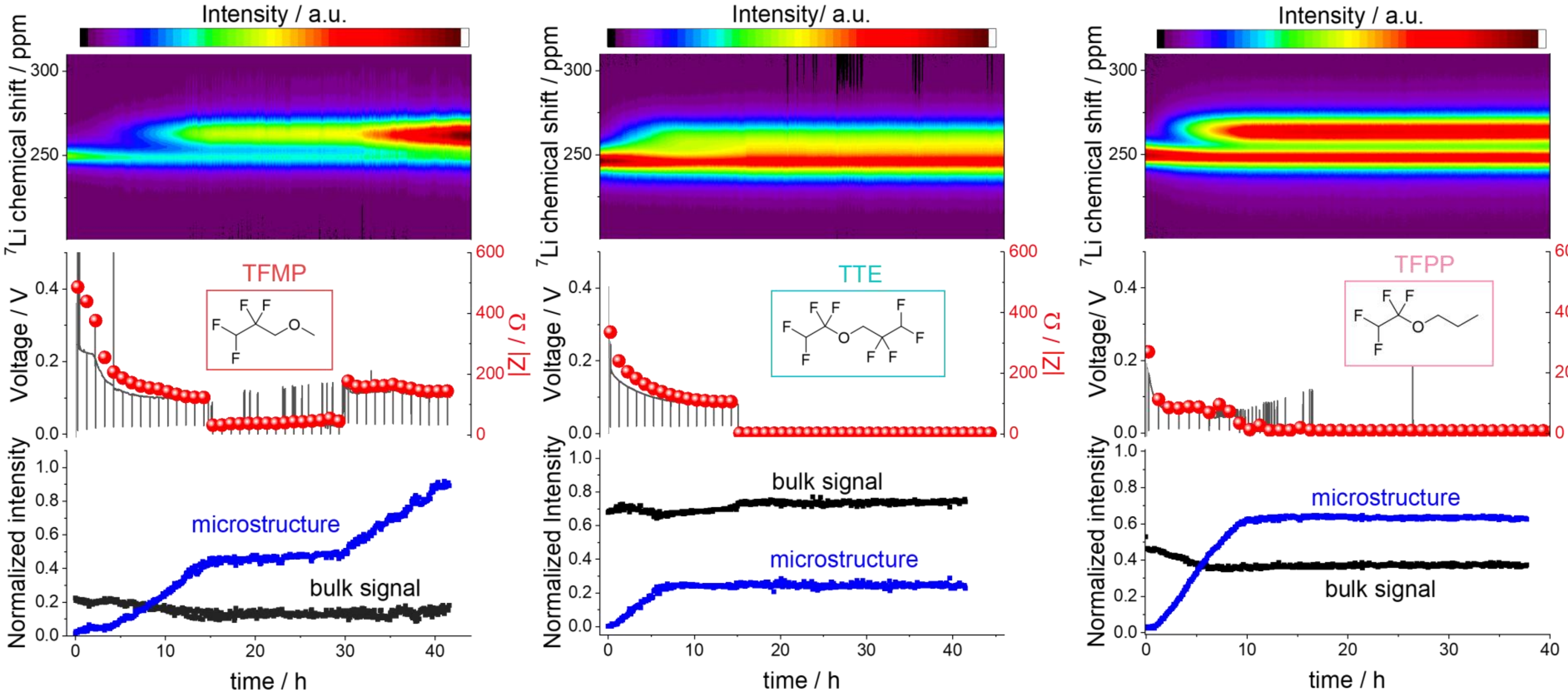

Figure S13. Investigation of the evolution of the morphology of lithium metal anodes by *operando* ^{7}Li NMR measurements of symmetrical lithium cells during unidirectional lithium plating experiments, with electrolytes with the fluorinated ethers with chemical formula shown in the figure: TFMP (left panel), TTE (middle panel) and TFPP (right panel), using a DME:FE solvent ratio of 3:2, where FE is the fluorinated ether. The top panel shows the evolution of the ^{7}Li NMR spectra, the middle panel shows the evolution of the cell voltage and cell impedance, and the lower panel shows the evolution of the intensity of the bulk lithium metal signal (at ca. 250 ppm) and of the signal due to lithium microstructures (at ca. 265-275 ppm), both normalized to the total signal at the end of the experiments.

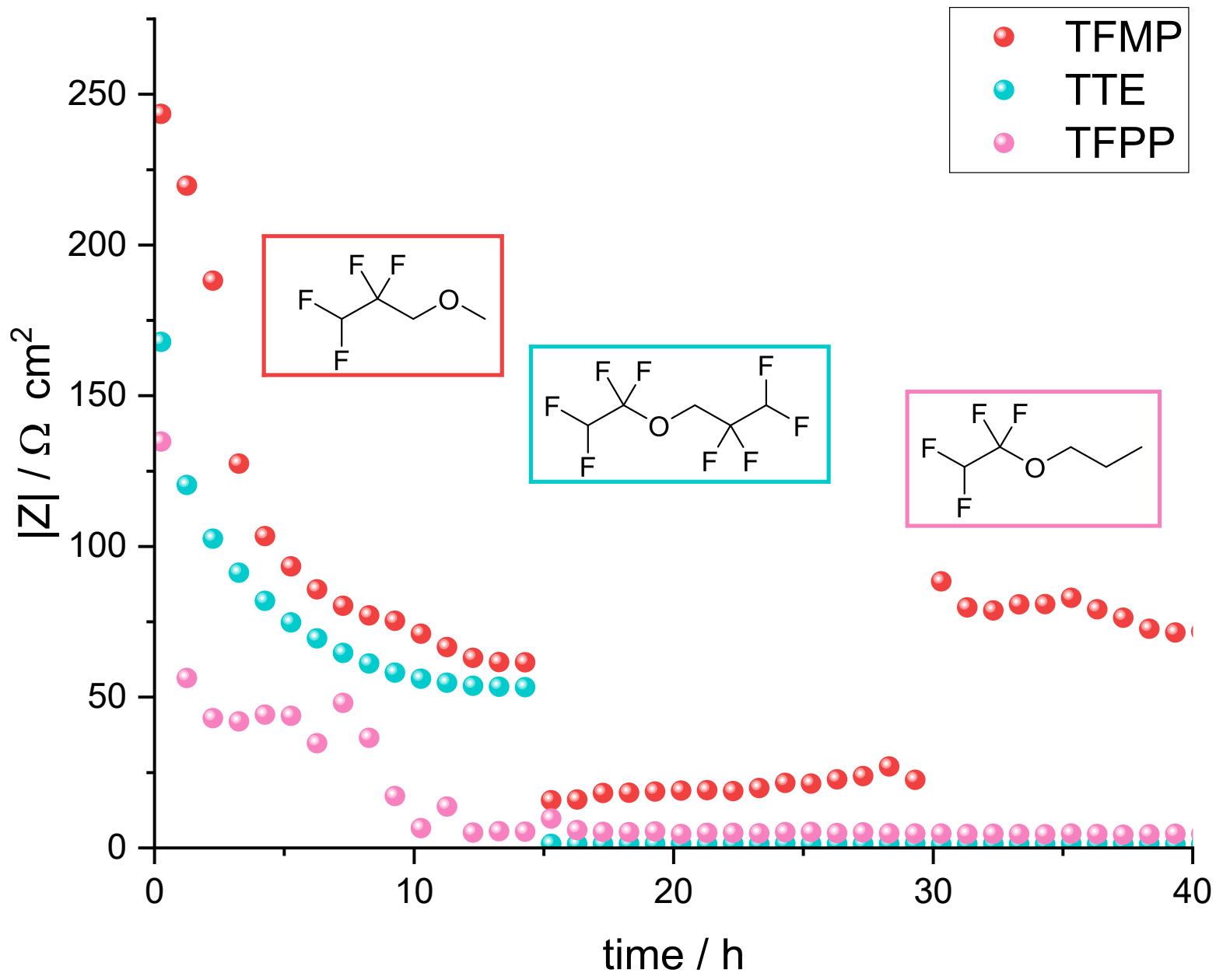

Figure S14. Evolution of the magnitude of the impedance at 9 Hz, as recorded with the *operando* ^{7}Li NMR lithium plating experiments shown in Figure S13.

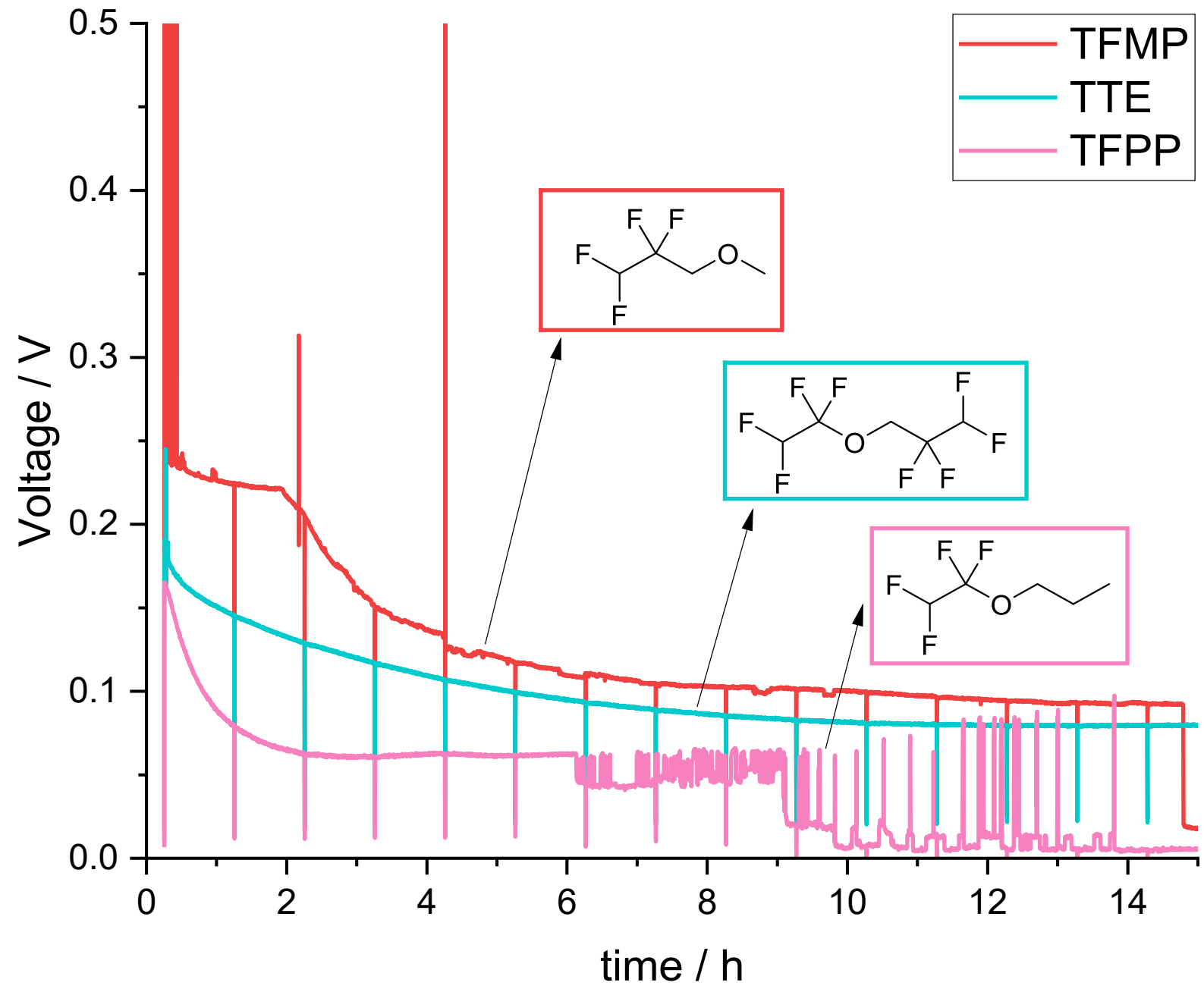

Figure S15. Evolution of the cell voltage at the beginning of the *operando* ^{7}Li NMR lithium plating experiments shown in Figure S13.

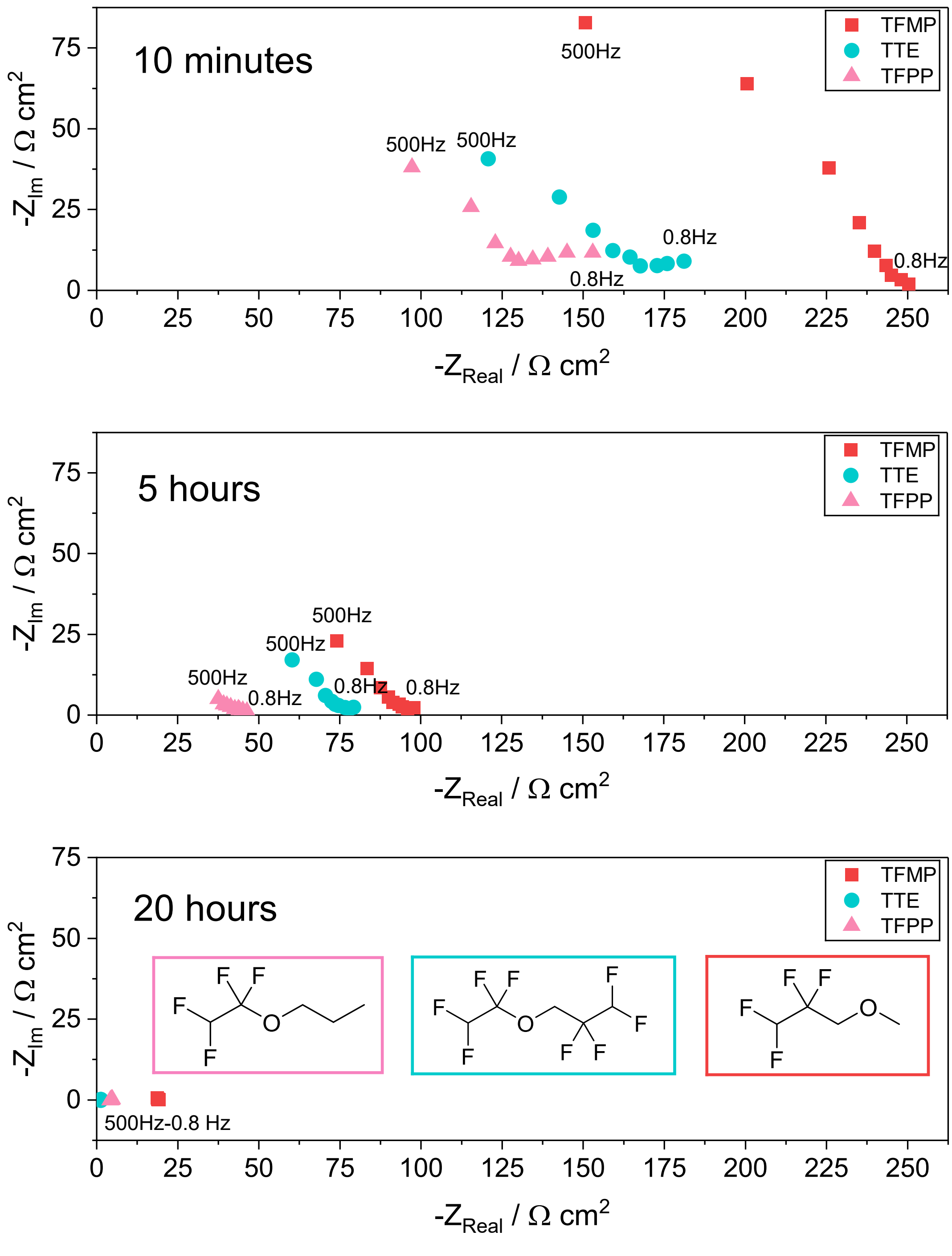

Figure S16. Nyquist plot of the impedance results obtained after 10 minutes, 5 hours or 20 hours of lithium plating, as recorded with the *operando* ^{7}Li NMR measurements shown in Figure S13.

S7. Analysis of the effect of cell-to-cell reproducibility on performance metrics

The graphs below show that the discussed effects of fluorinated ethers on longevity (shown in Figures 2, 5-6, S4-5) and on rate capability (shown in Figures 3-4) are larger than the cell-to-cell reproducibility, where the latter is shown as the error bars in chart graphs plotting performance metrics at selected cycle numbers or C-rate.

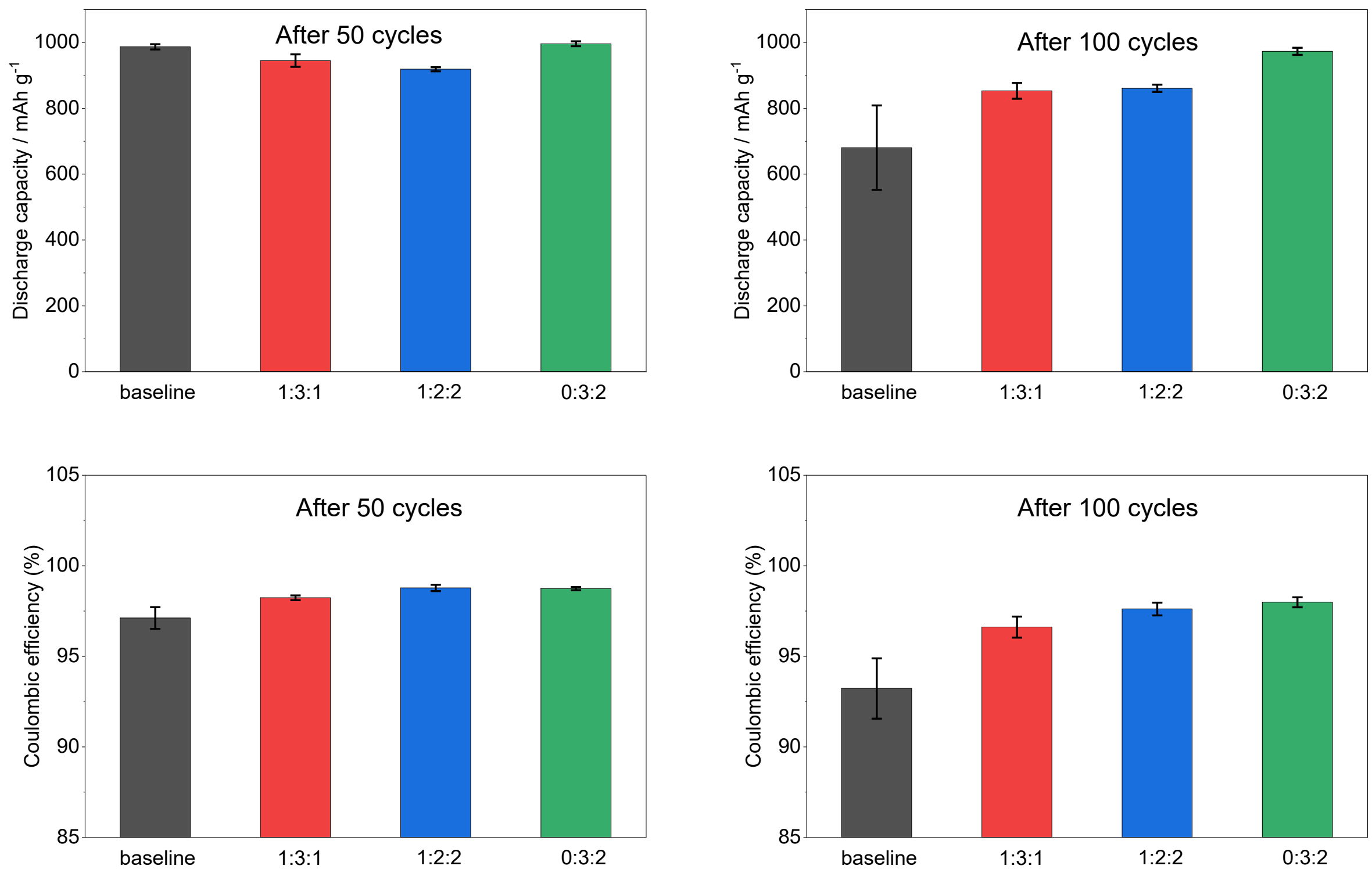

Figure S17. Discharge capacity and coulombic efficiency at cycles 50 and 100 for the conditions in Figure 2, where the electrolyte formulations are 1M LiTFSI + 0.25M $LiNO_3$ in DOL:DME 1:1 (baseline, black), and DOL:DME:TFMP with solvent ratios of 1:3:1 (red) and 1:2:2 (blue) and 0:3:2 (green). The error bars show the standard deviation from repeat experiments.

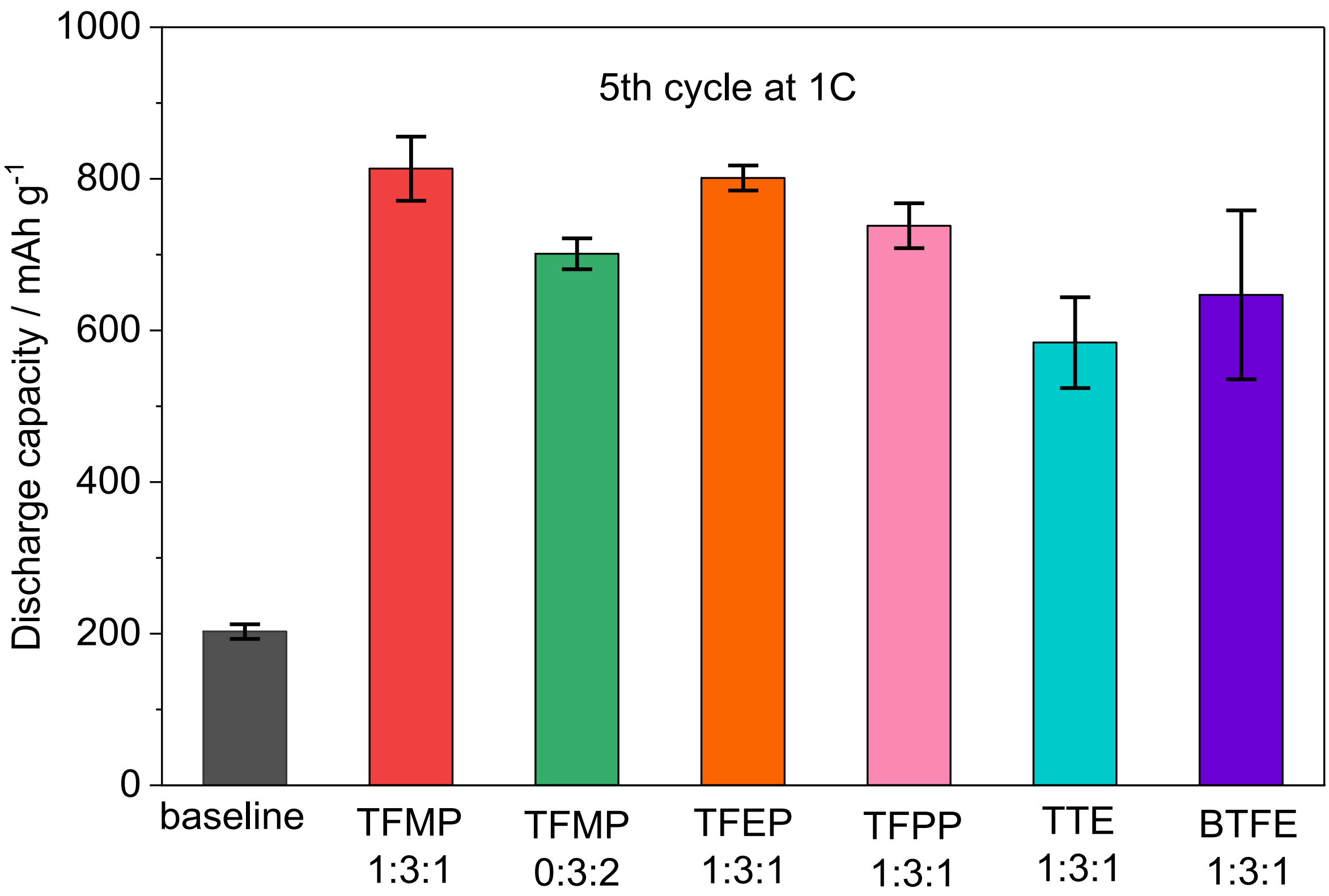

Figure S18. Discharge capacity obtained at 1C cycling for the conditions in Figures 3 and 4, where the electrolytes are 1M LiTFSI + 0.25M $LiNO_3$ in: DOL:DME 1:1 (baseline, black), DOL:DME:TFMP with solvent ratios of 1:3:1 (red) and 0:3:2 (green), and DOL:DME:TFEP 1:3:1 (orange), DOL:DME:TFPP 1:3:1 (pink), DOL:DME:TTE 1:3:1 (light blue) and DOL:DME:BTFE 1:3:1 (purple). The error bars show the standard deviation from repeat experiments.

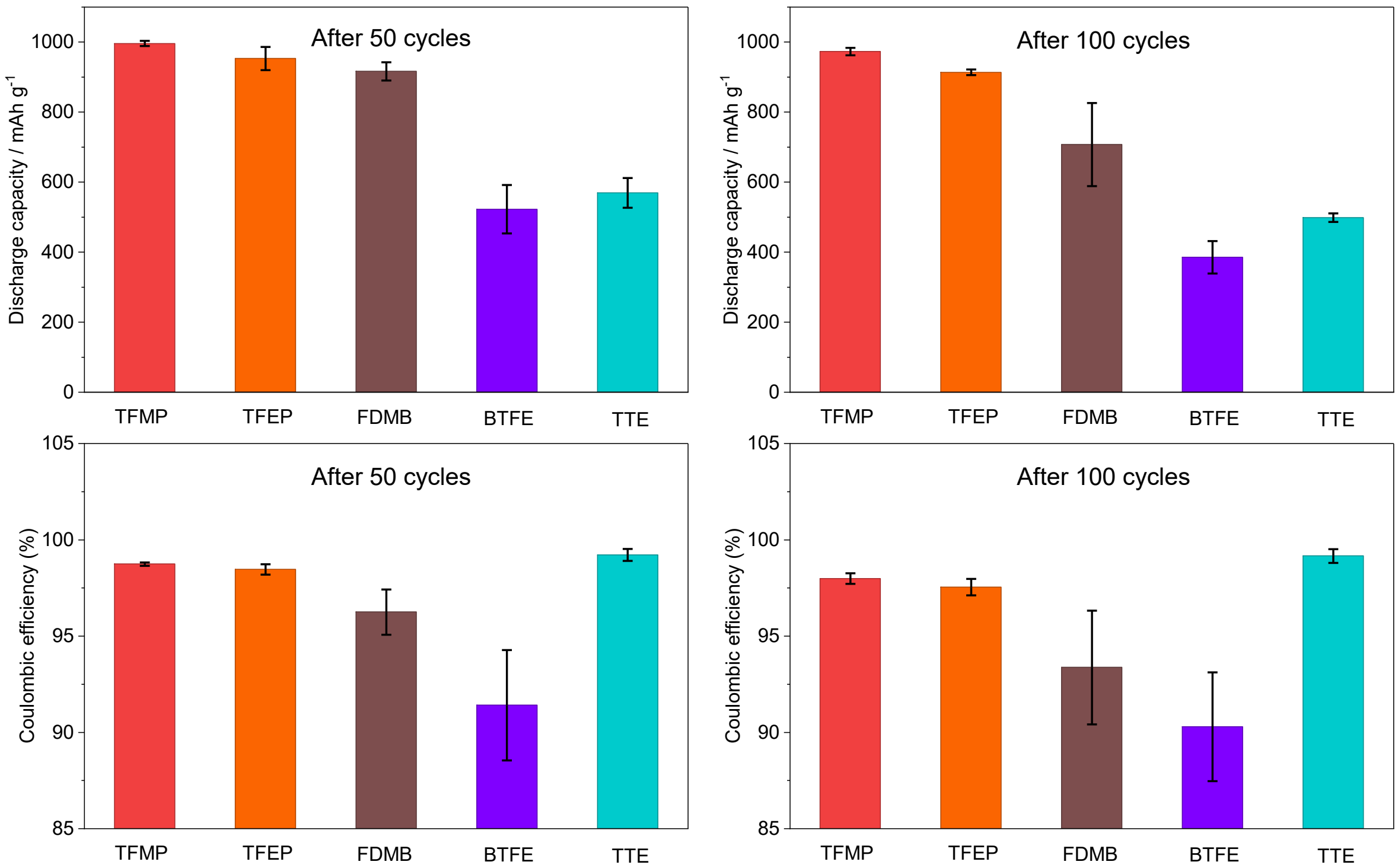

Figure S19. As in Figure S17 but for the conditions in Figures 5, where the electrolyte formulations are 1M LiTFSI + 0.25M $LiNO_3$ in DME:FE equal to 3:2, where FE is the fluorinated ether TFMP (red), TFEP (orange), FDMB (brown), BTFE (purple), and TTE (light blue). Note that the high coulombic efficiency of Li-S cells with TTE is a result of the low capacity values obtained in this electrolyte, as reaction reversibility improves when the extent of the completion of the electrochemical reactions is low.

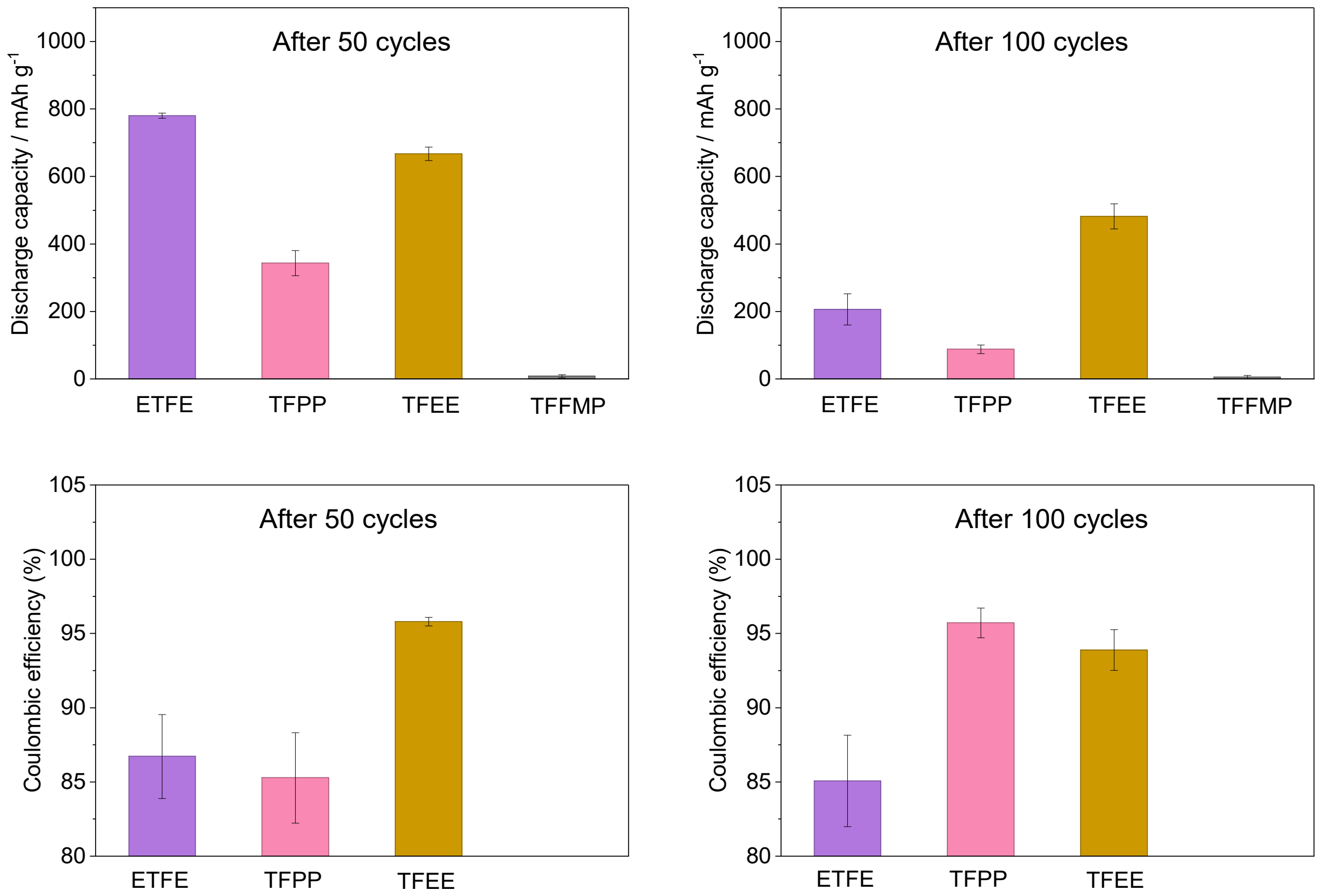

Figure S20. As in Figure S19 but for the conditions in Figure 6, with fluorinated ethers TFPP (pink), ETFE (lilac), TFEE (gold), and TFFMP (grey). Note that the higher capacity retention of TFEE is related to the fact that this electrolyte delivers smaller initial capacity, thus resulting in mitigated degradation. The coulombic efficiency values with TFFMP are not shown as their associated error is large, as a result of the very low capacities obtained with this electrolyte.

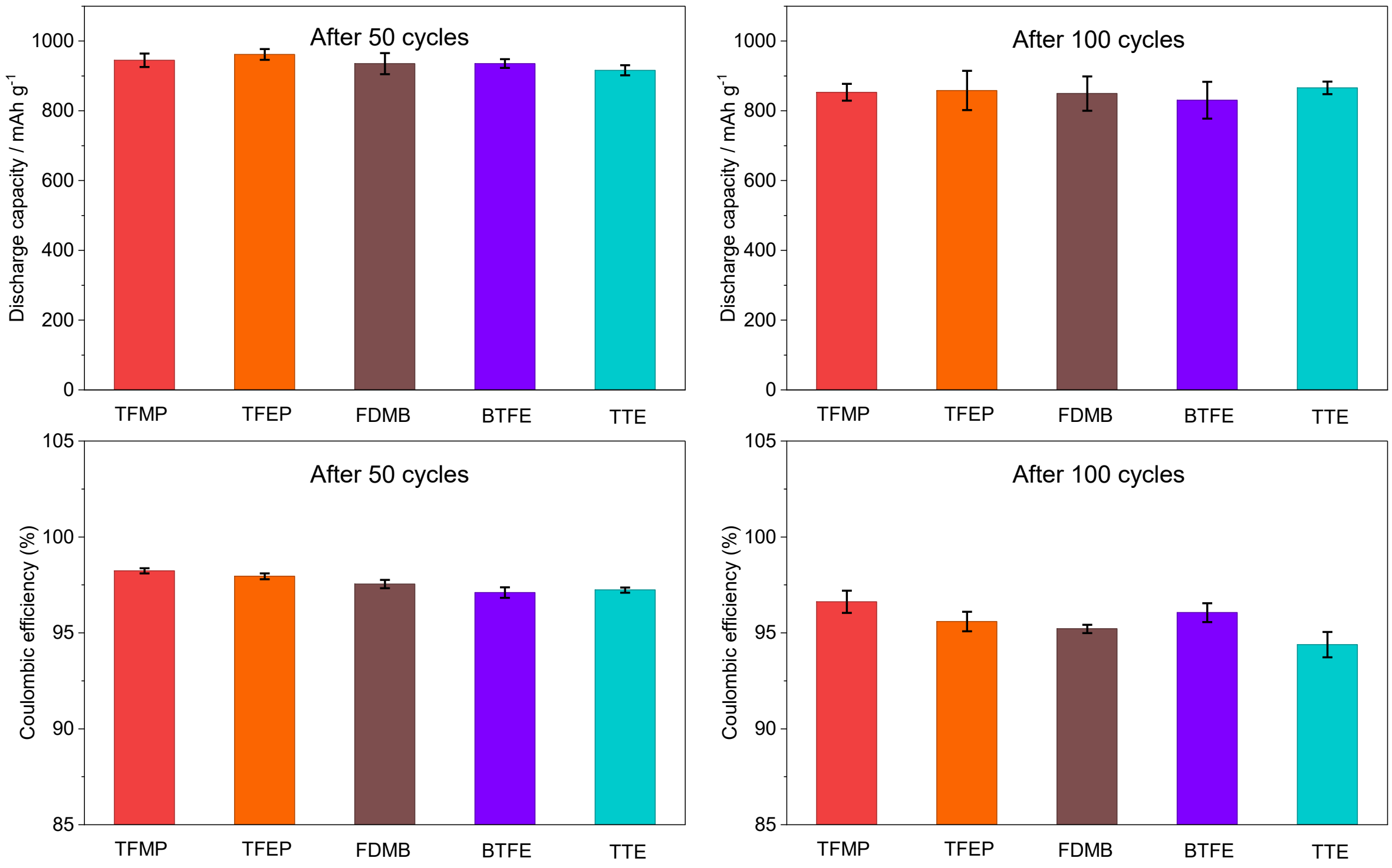

Figure S21. As in Figure S17 but for the conditions in Figures S4, where the electrolyte formulations are 1M LiTFSI + 0.25M $LiNO_3$ in DOL:DME:FE equal to 1:3:1, where FE is the fluorinated ether TFMP (red), TFEP (orange), FDMB (brown), BTFE (purple), and TTE (light blue).

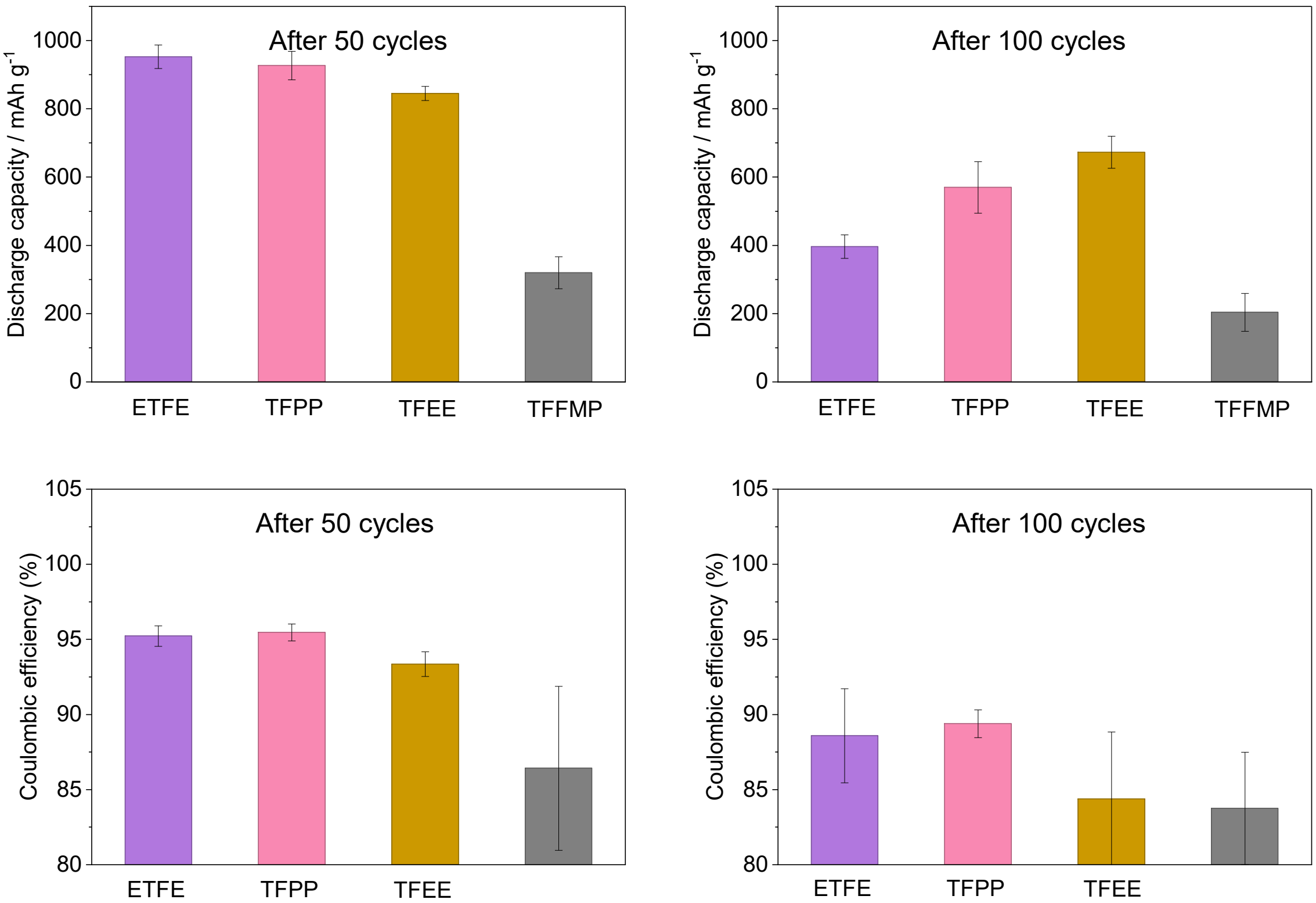

Figure S22. As in Figure S21 but for the conditions in Figure S5, with fluorinated ethers TFPP (pink), ETFE (lilac), TFEE (gold), and TFFMP (grey), shown in Figure S5. Note that the higher capacity retention of TFEE is related to the fact that this electrolyte delivers smaller initial capacity, thus resulting in mitigated degradation.

S8. Calculations of projected specific energies and energy densities of Li-S batteries

The experimental conditions of this work were selected to be representative of commercial Li-S battery applications, which require achieving a high specific energy and energy density. Figure S23 shows the results of the projected specific energy and energy density of a Li-S battery made with the sulfur electrodes formulation used here (with 65 wt% sulfur content and 3 mg/cm^2 sulfur loading) and using the experimental discharge capacities at C/10 (1100 mAh/g) and experimental discharge voltage (2.1 V). The calculations are made as a function of the electrolyte-to-sulfur ratio, since pouch cells enable a much smaller electrolyte loading than the coin cells here employed, as in the latter a significant volume of electrolyte is lost in the empty cell volume inside the cell (known as 'dead volume'), and consequently, the actual electrolyte-to-sulfur ratio inside the cell stack of the coin cell (comprising the electrodes and the separator) is much lower than the nominally quoted electrolyte-to-sulfur ratio that is calculated using the whole volume of electrolyte added to the cell. The calculations are done using the thicknesses and densities of battery grade Al and Cu current collectors and separators, as detailed in Table S7.

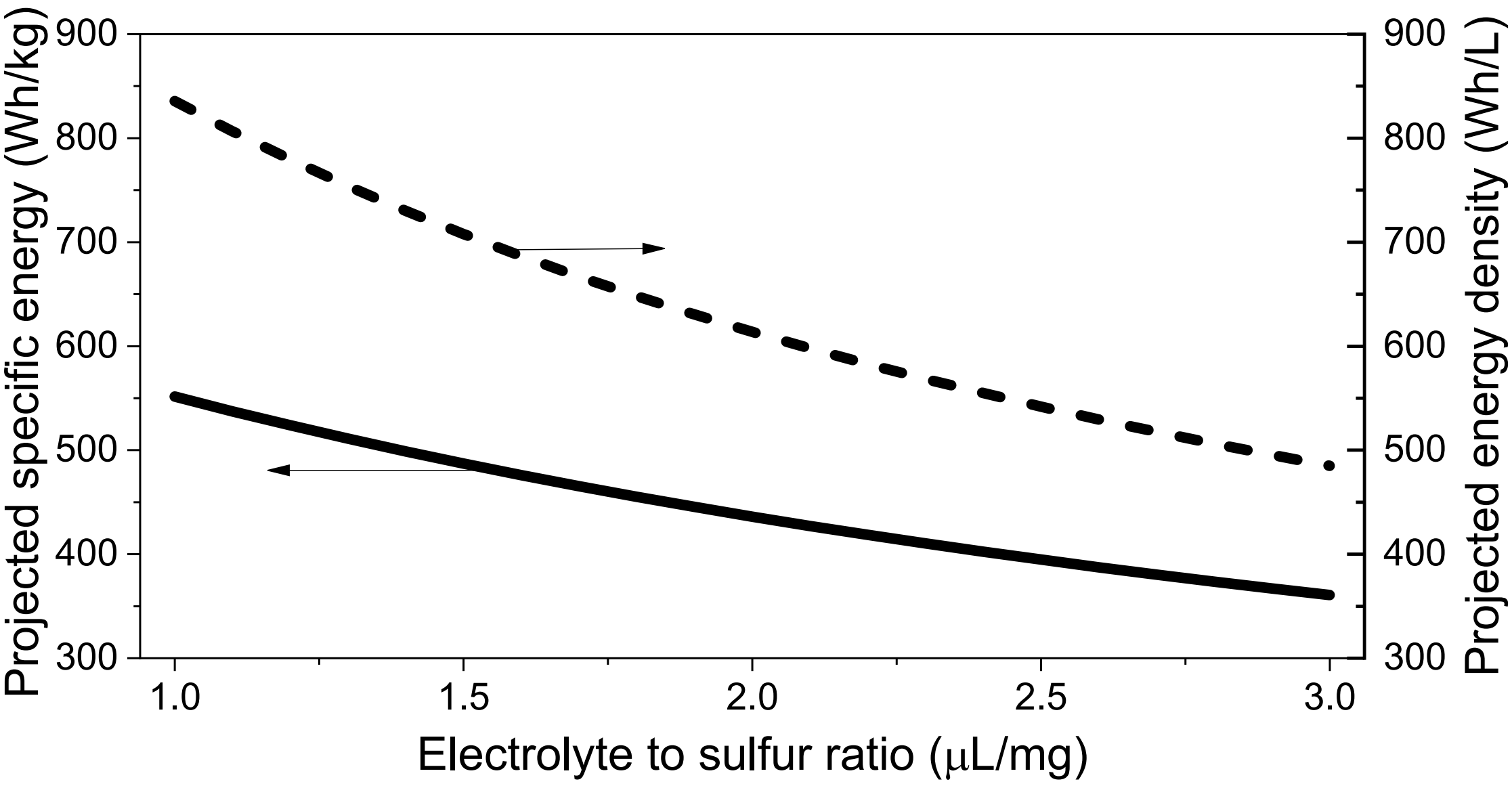

Figure S23. Projected specific energy and energy density of a Li-S battery made with the sulfur electrodes here employed, using the experimental results of discharge capacity at C/10 (1100 mAh/g) and discharge voltage (2.1 V) with the baseline electrolyte (1M LiTFSI + 0.25M $LiNO_3$ in DOL:DME 1:1) that has a density of 1.11 g/mL. Thin separators (12 μm) and Al (8 μm) and Cu (4.5 μm) current collectors are considered for the calculations, as well as a Li metal anode with 50% capacity excess, as detailed in Table S7.

Figure S23 shows that the Li-S batteries here considered can deliver specific energies exceeding 400 Wh/kg with an electrolyte-to-sulfur ratio lower than 2.5 μL/mg. However, these calculations are done for the baseline electrolyte, which leads to limited longevity and rate capability of the batteries. Here we show that using electrolytes with fluorinated ethers can improve significantly these performance metrics, but fluorinated ethers have higher density than the DOL and DME solvents that made the baseline electrolyte. To evaluate the effect of the increased density of the fluorinated ethers, the specific energy calculations were extended to electrolytes with densities calculated from the experimental densities of the

fluorinated ethers, as listed in Table S8, and the results are shown in Figure S24 for an electrolyte-to-sulfur ratio of 2 µL/mg. It is clear that the best performing fluorinated ether, TFMP, produces a projected specific energy that is close to that of the baseline electrolyte. On the other hand, other fluorinated ethers used most commonly in the literature, such as TTE and BTFE, produce a more significant decrease in the projected specific energy as a result of their higher density.

Table S7. Values of thickness, bulk density and porosity used for the calculations of the specific energy and energy density of Li-S cells in Figures S23-24.

Component	Thickness	Bulk density	Porosity	Reference
Sulfur electrode with 65 wt% sulfur and 3 mg/cm^2 sulfur loading	83 µm	1.99 g/mL	0.72	[87]
Celgard separator	12 µm	0.9 g/mL[88]	0.38	[89]
Li metal electrode providing 50% capacity excess	16 µm	0.534 g/mL		
Al current collector	8 µm[a]	2.7 g/mL		[90]
Cu current collector	4.5 µm[a]	8.96 g/mL		[90]

a: calculations are done using half of the total thickness of the current collectors to reflect the projected energy of a multilayer Li-S pouch cell with double-side coated electrodes.

Table S8. Density values of fluorinated ethers used in this work.

Fluorinated ether	Density	Reference
TFMP	1.186 g/mL	[91]
TFEP	1.166 g/mL	[29]
FDMB	1.21 g/mL	[92]
BTFE	1.404 g/mL	[91]
TTE	1.533 g/mL	[91]
ETFE	1.198 g/mL	[91]
TFPP	1.128 g/mL	[91]
TFEE	1.418 g/mL	[91]
TFFMP	1.275 g/mL	[91]

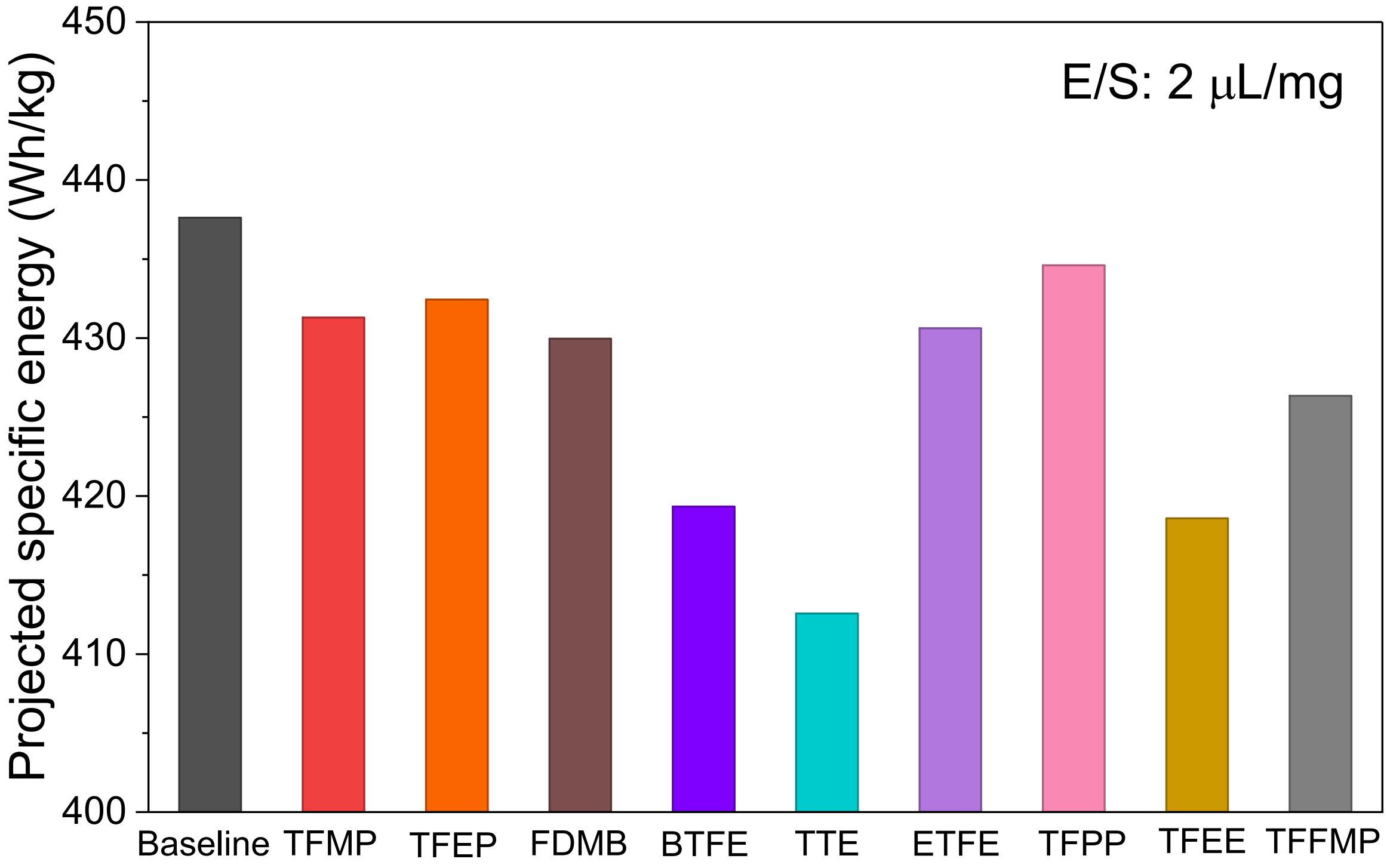

Figure S24. As in Figure S23 but using the calculated densities of the electrolytes 1M LiTFSI + 0.25M $LiNO_3$ in DME:FE 3:2, where FE is the fluorinated ether whose name is indicated in the figure. The calculations employ an electrolyte-to-sulfur ratio of 2 μL/mg, a discharge capacity of 1100 mAh/g, a discharge voltage of 2.1 V and electrolyte densities calculated considering additive volumes of all electrolyte components, using 0.867 g/mL for DME,[88] 2.15 g/mL for LiTFSI,[88] 2.38 g/mL for $LiNO_3$,[88] and the fluorinated ether densities in Table S8.

S9. Repeat experiments demonstrating reproducibility

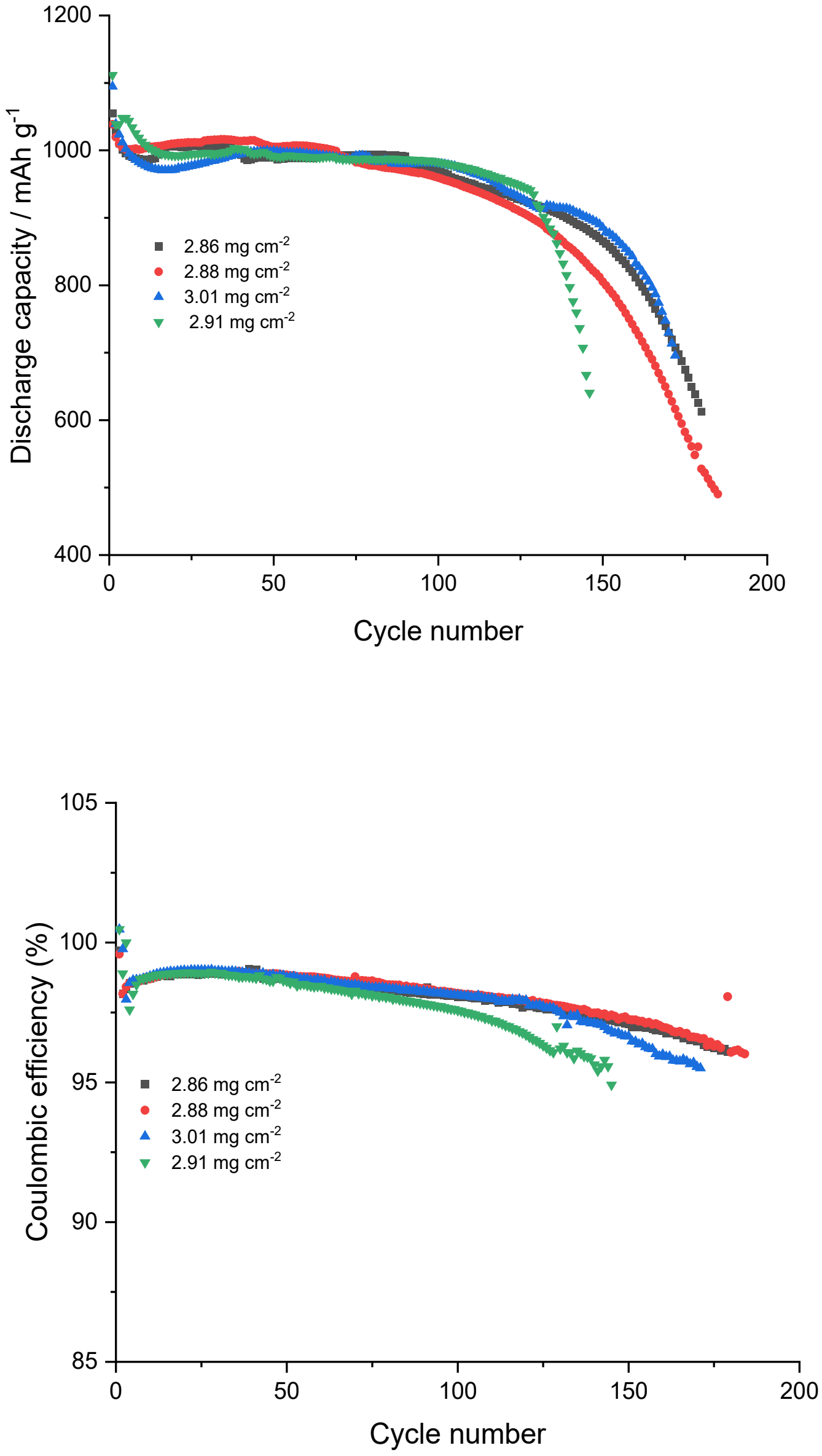

Figure S25. Repeats of Li-S cell cycling in 1M LiTFSI + 0.25M $LiNO_3$ in DME:TFMP 3:2 at C/5. The loadings of the different cells are indicated in the legend.

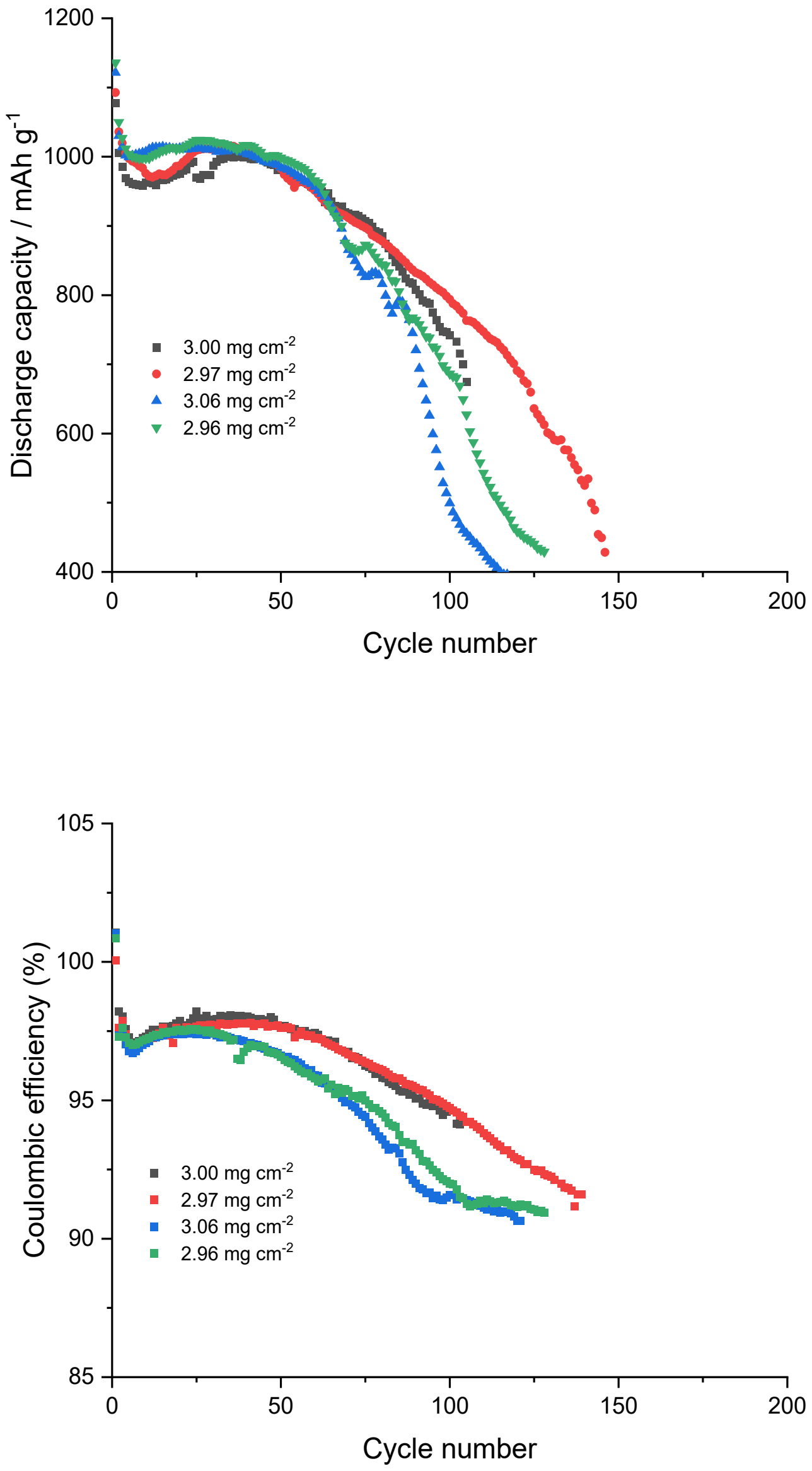

Figure S26. As in Figure S25 but in DOL:DME 1:1.

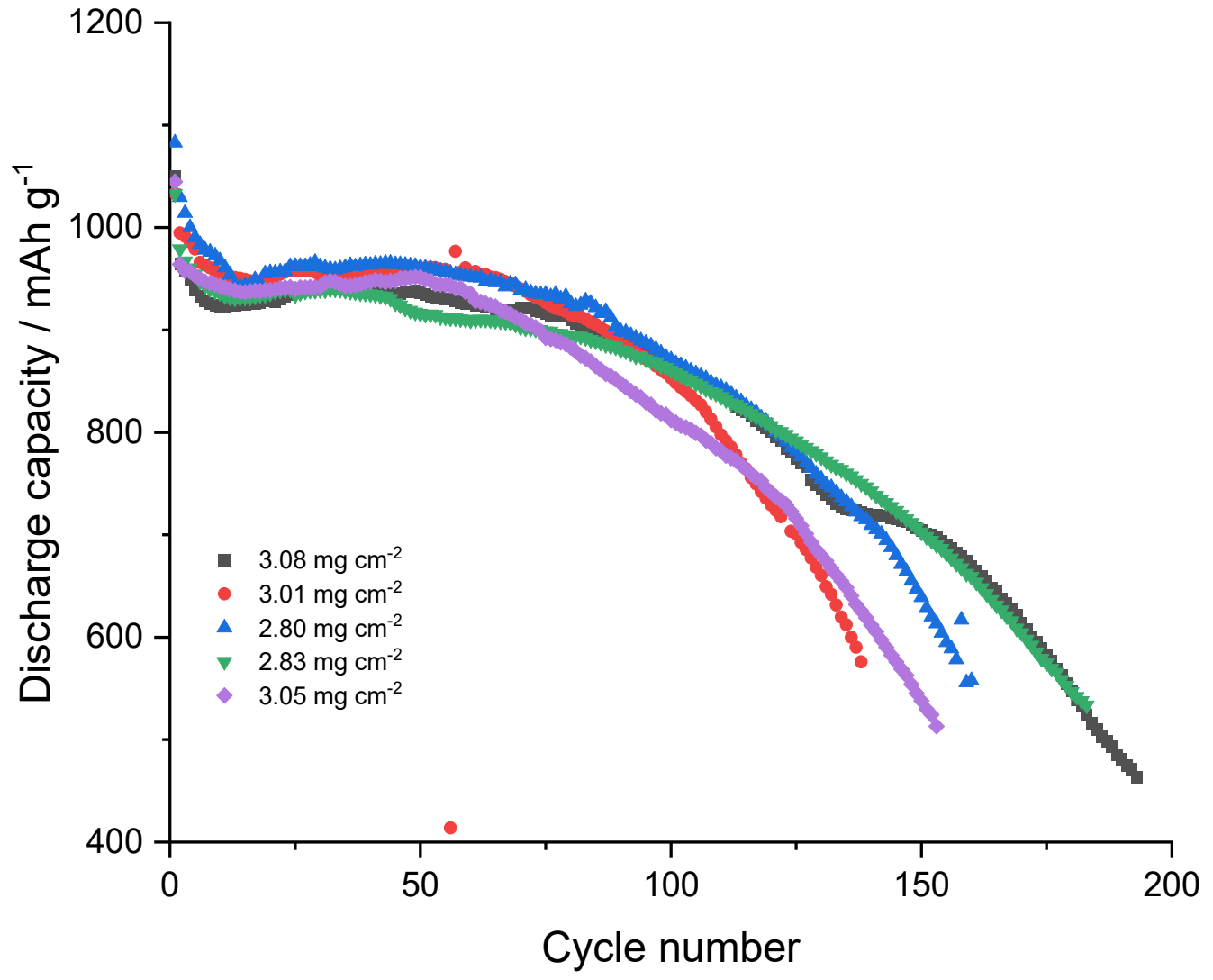

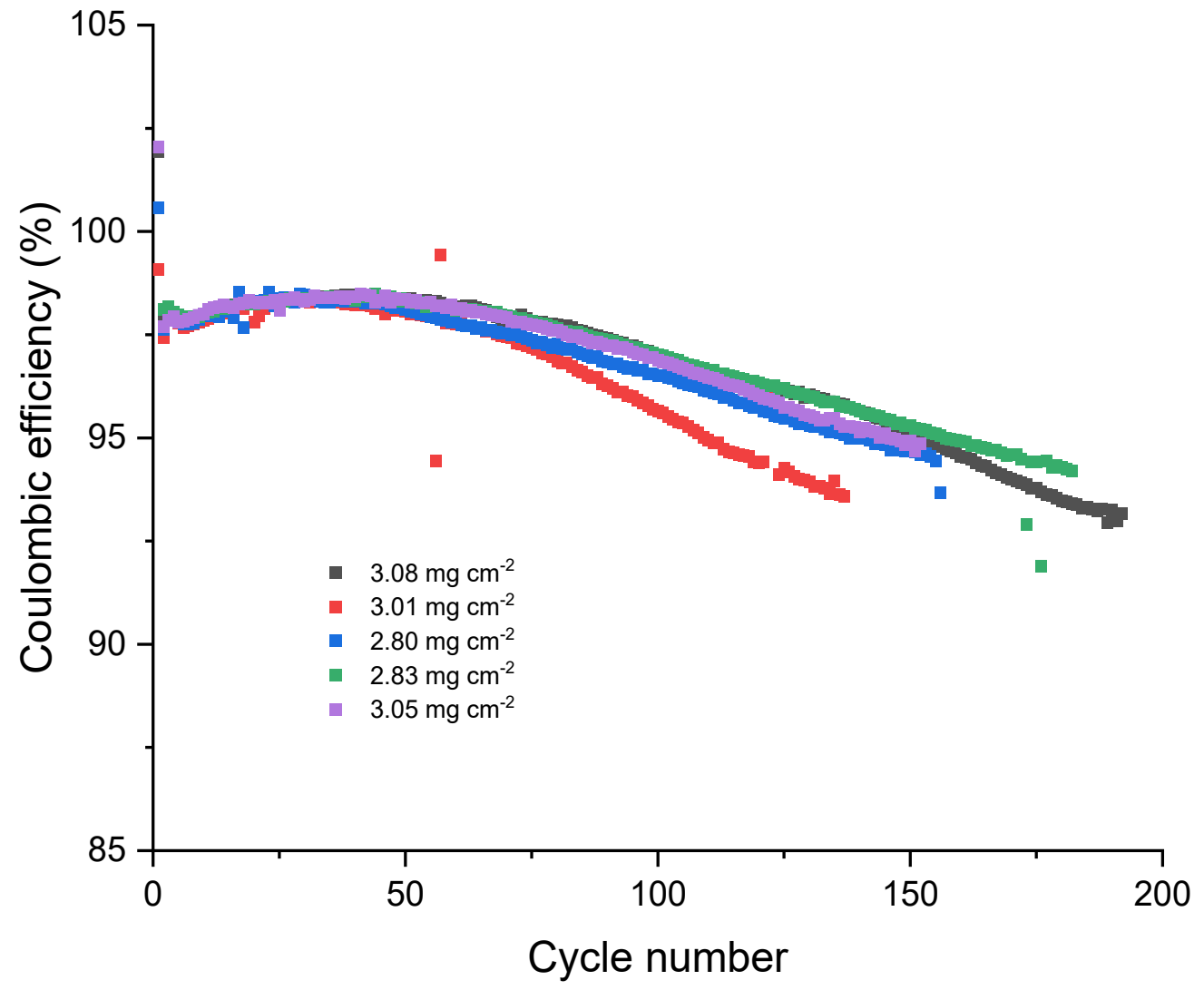

Figure S27. As in Figure S25 but in DOL:DME:TFMP 1:3:1.

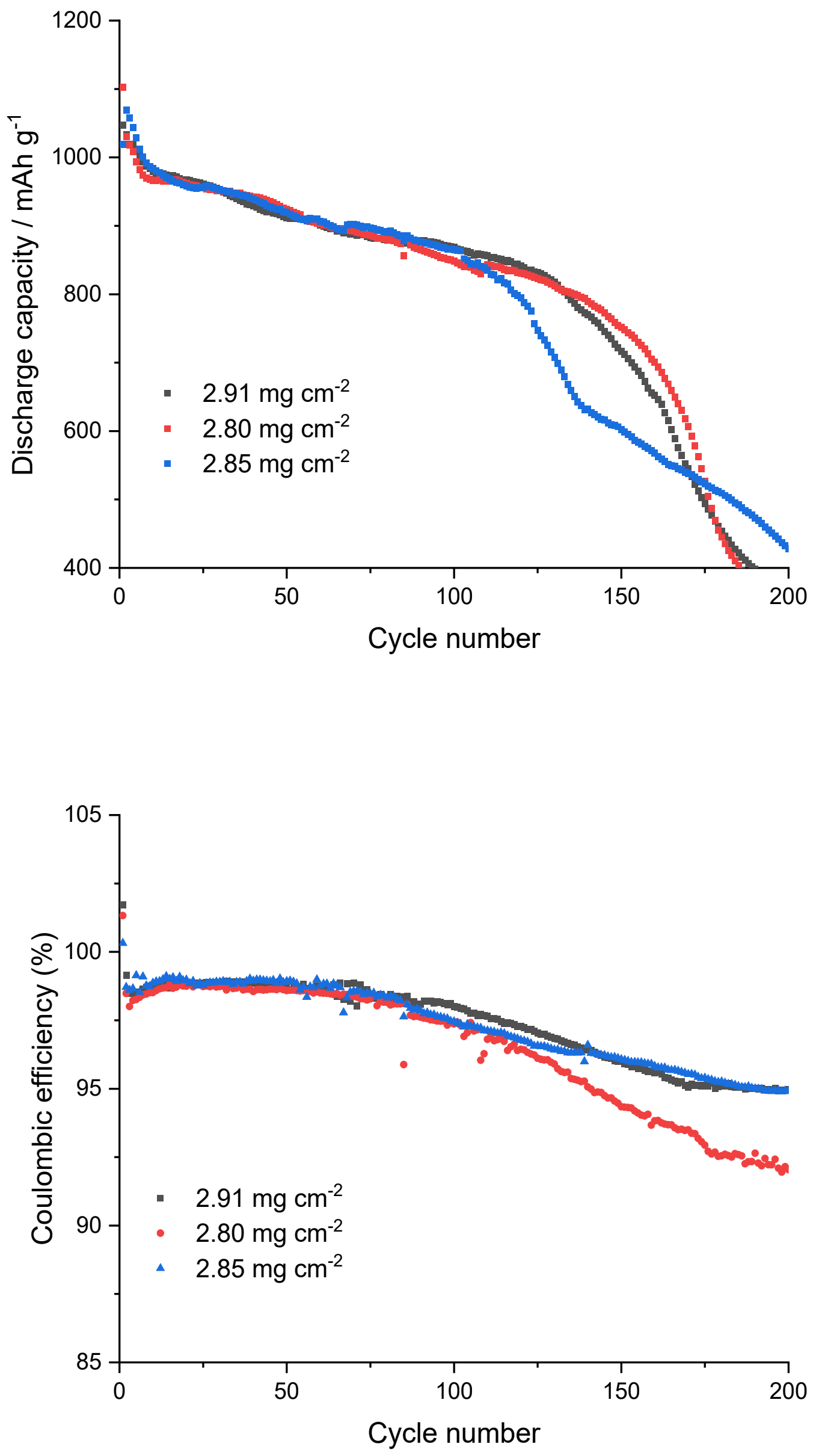

Figure S28. As in Figure S25 but in DOL:DME:TFMP 1:2:2.

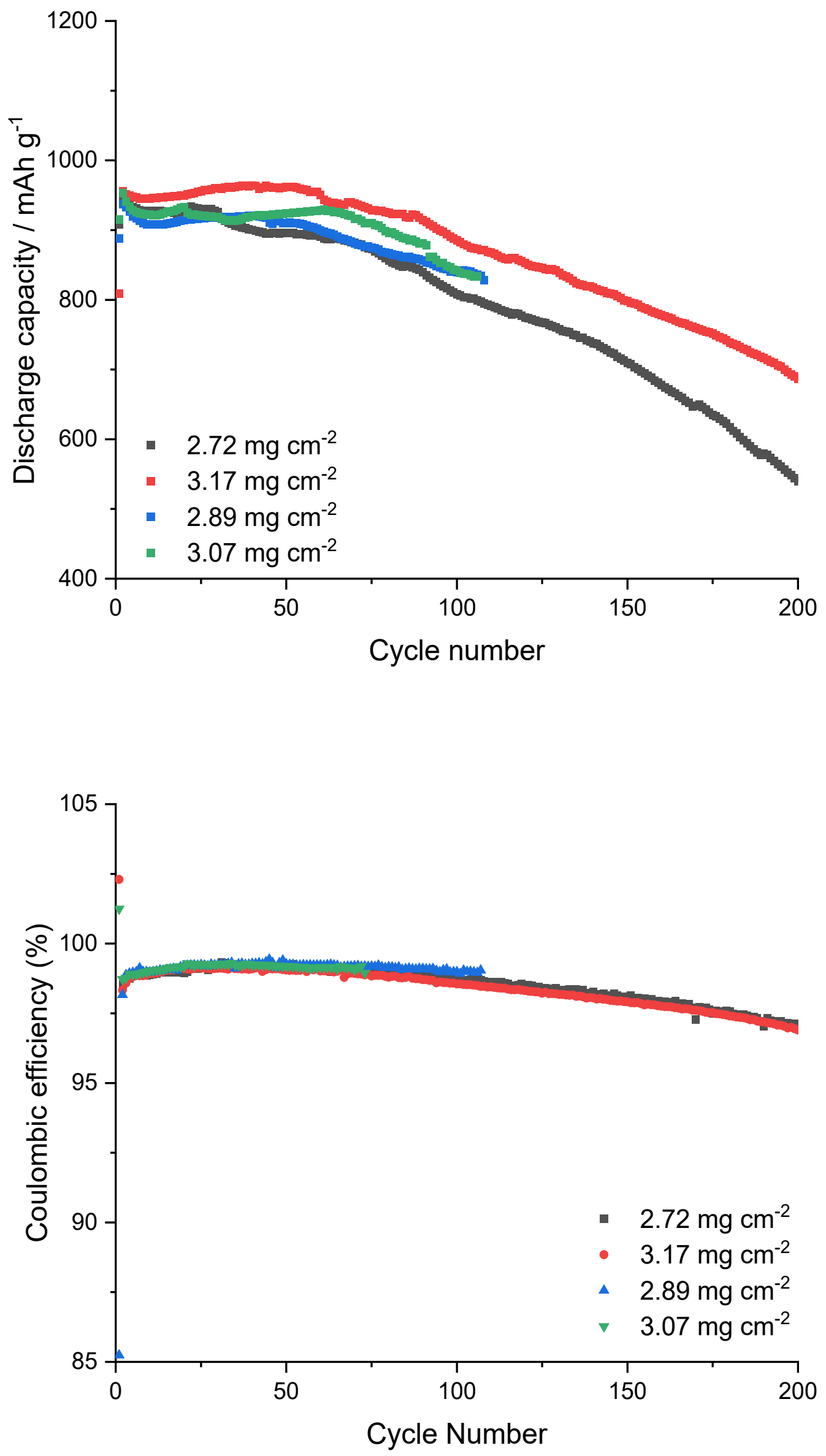

Figure S29. As in Figure S25 but in DME:TFMP 1:1.

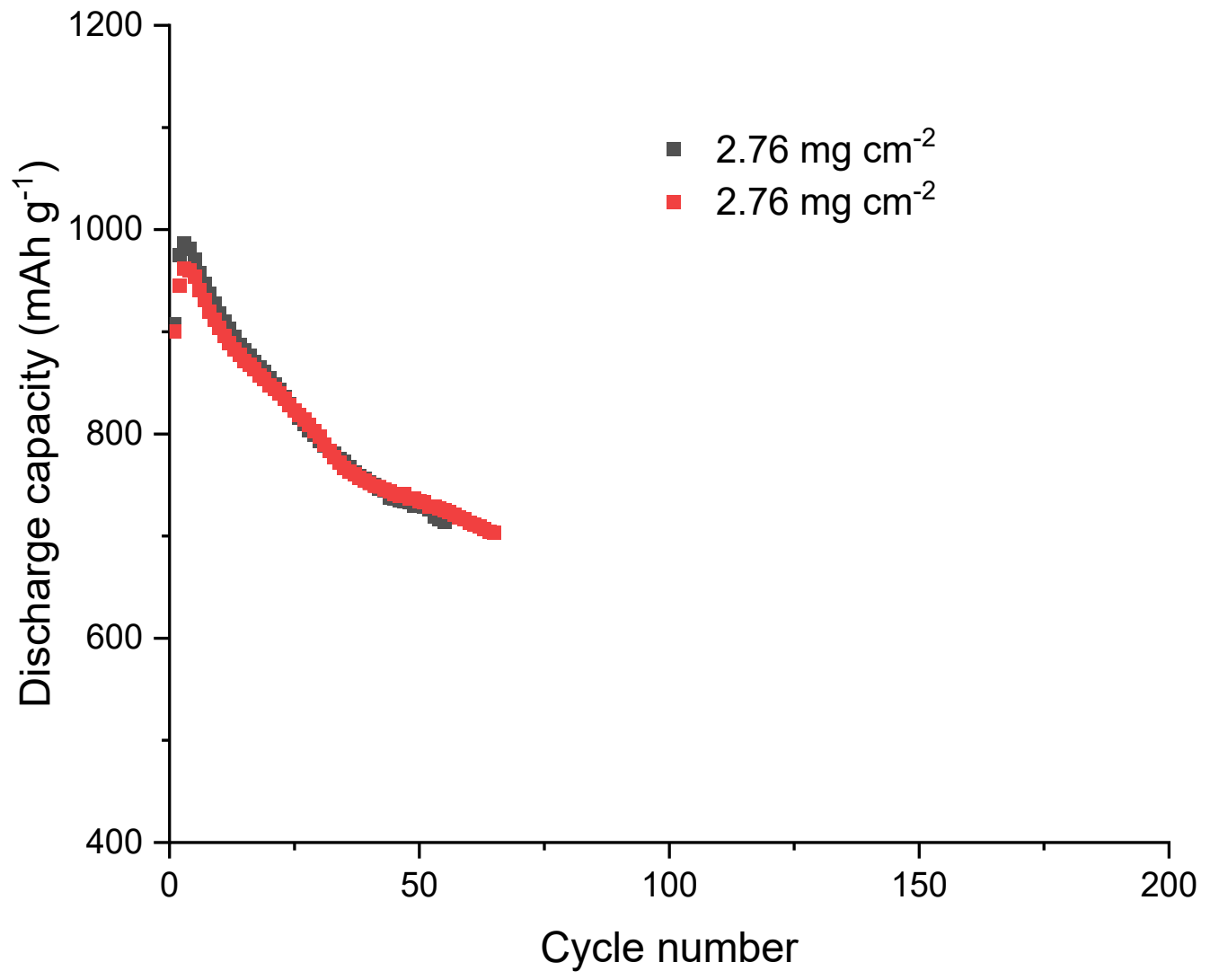

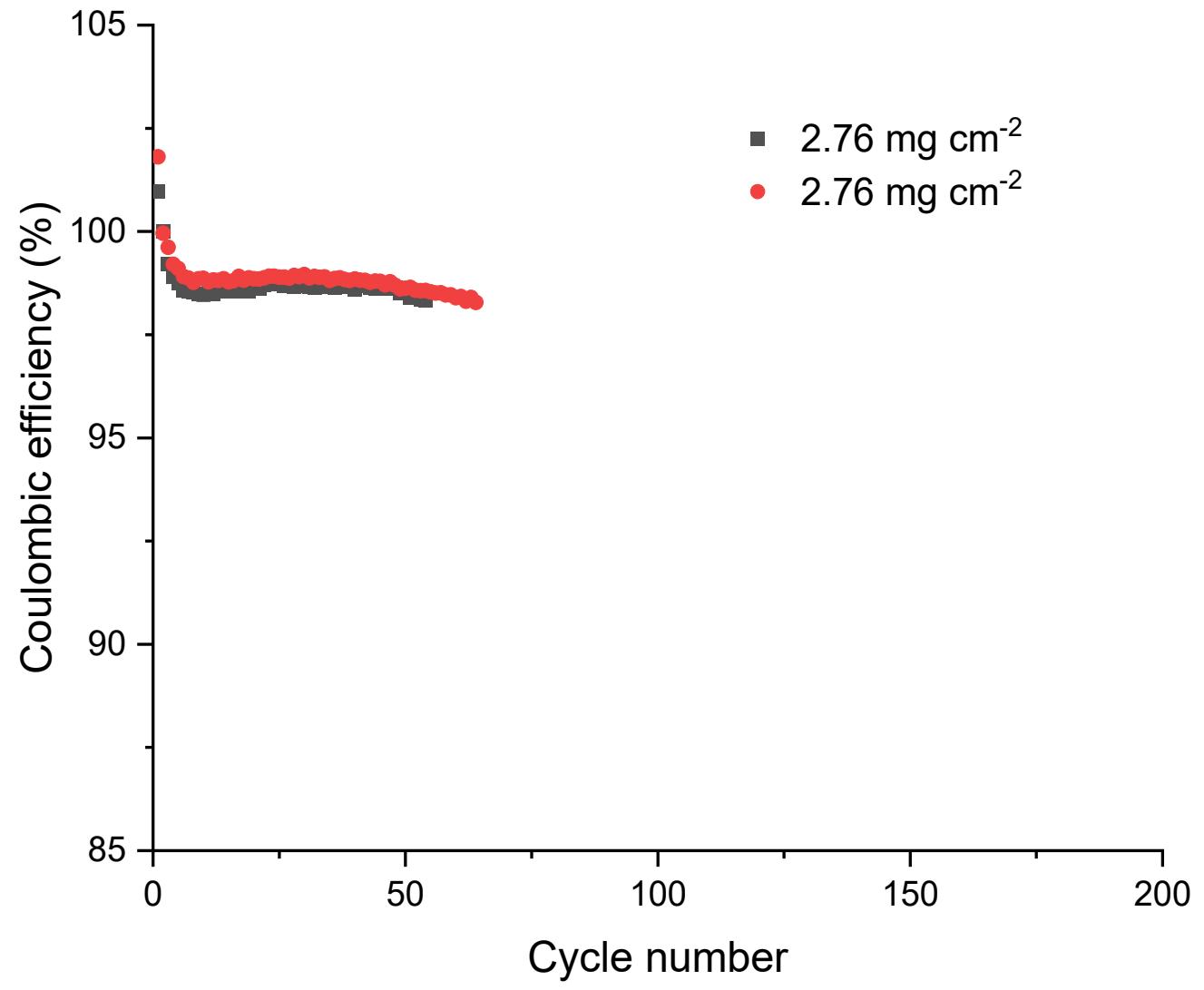

Figure S30. As in Figure S25 but in DOL:TFMP 1:1. Note that the salts did not fully dissolve in this formulation, and thus, the quoted concentrations (1M LiTFSI + 0.25M $LiNO_3$, used to prepare the solutions) are higher than the actual concentrations in solution.

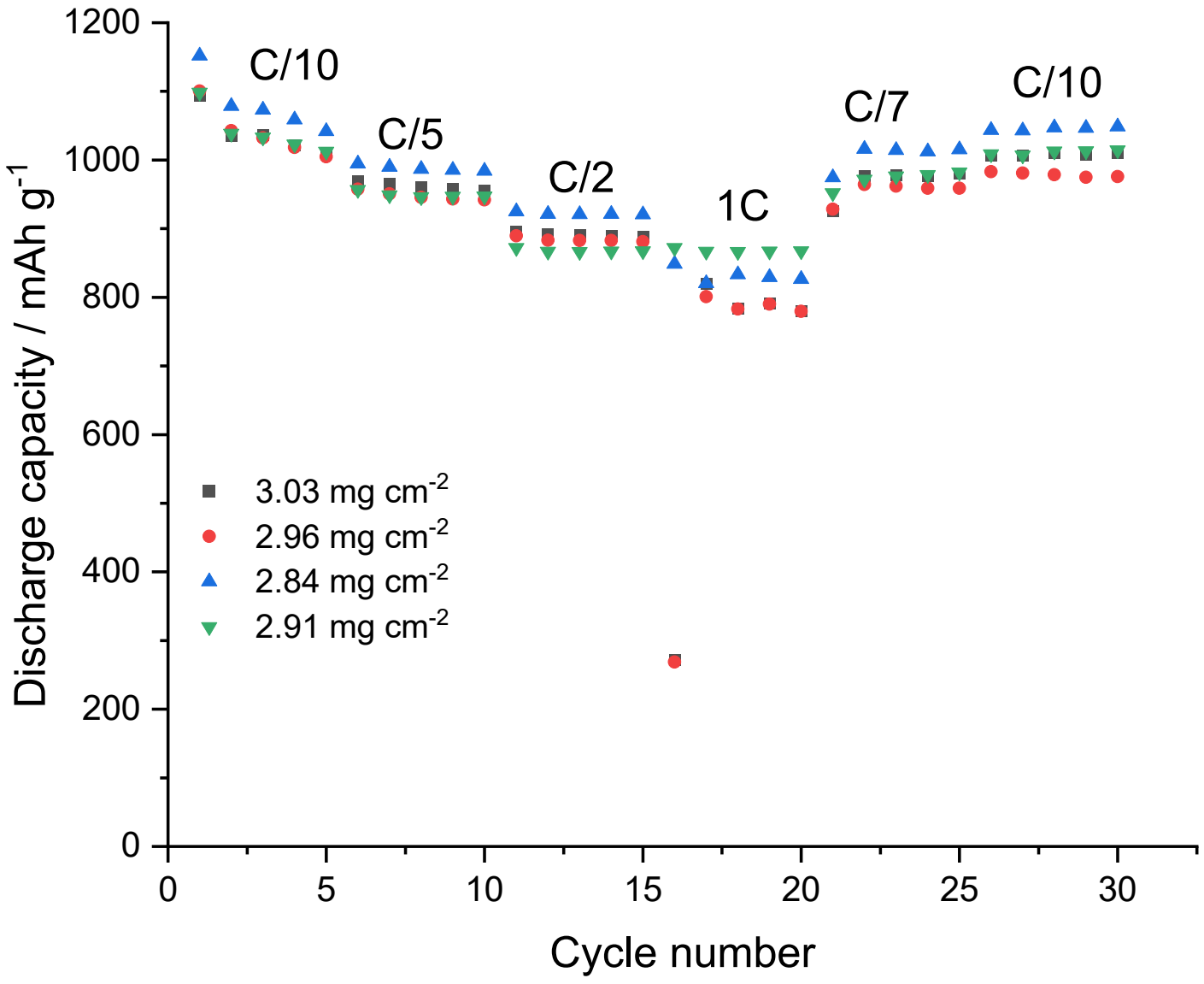

Figure S31. Repeats of rate capability testing of Li-S cells in 1M LiTFSI + 0.25M $LiNO_3$ in DOL:DME:TFMP 1:3:1 at the C-rates indicted in the Figures. The loadings of the different cells are indicated in the legend.

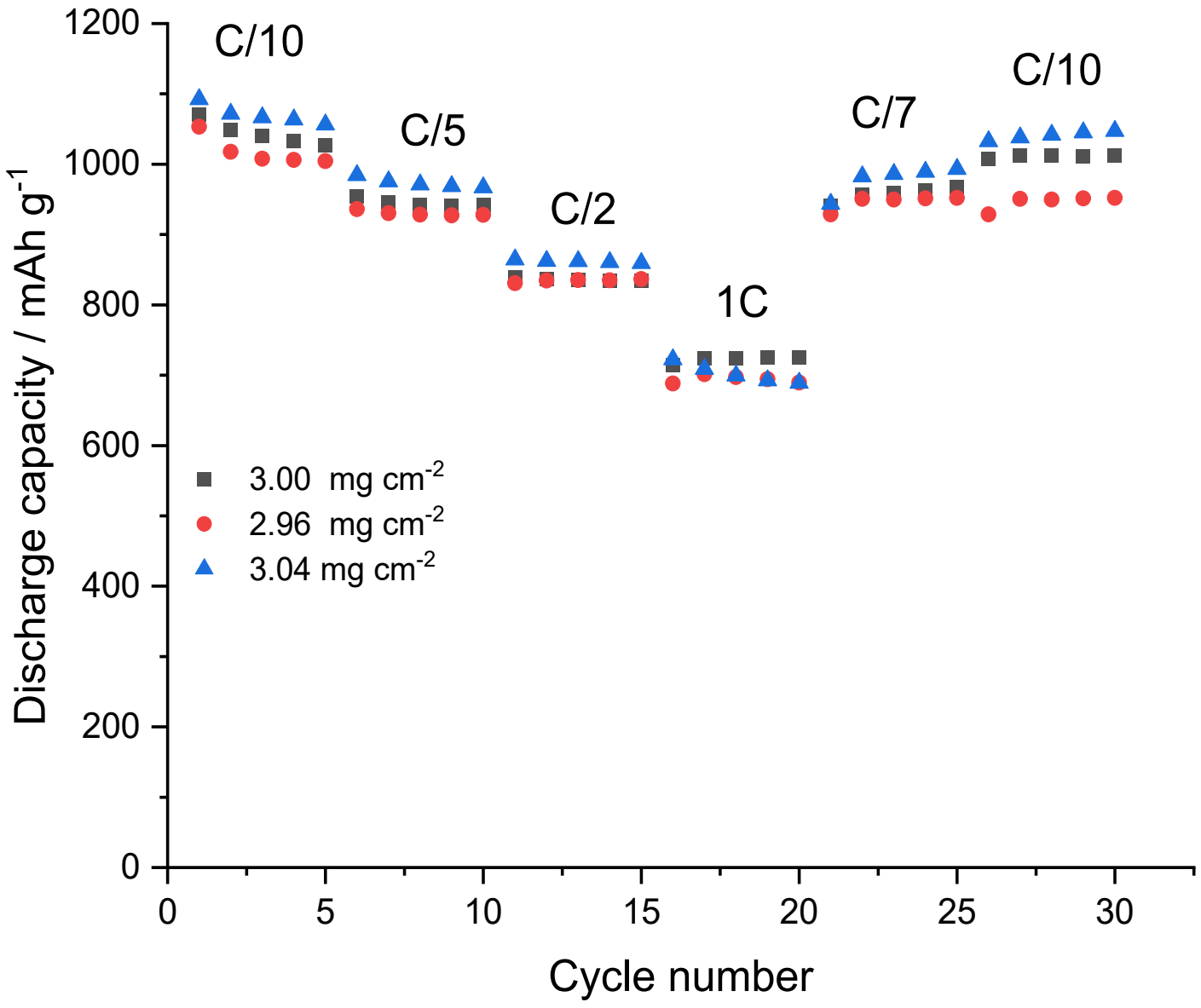

Figure S32. As in Figure S31 but in DME:TFMP 3:2. Note that additional repeat experiments produced smaller capacities (not shown), which can be tentatively ascribed to the high sensitivity of these measurements to the details in cell fabrication, e.g. the optimal location of the electrolyte.

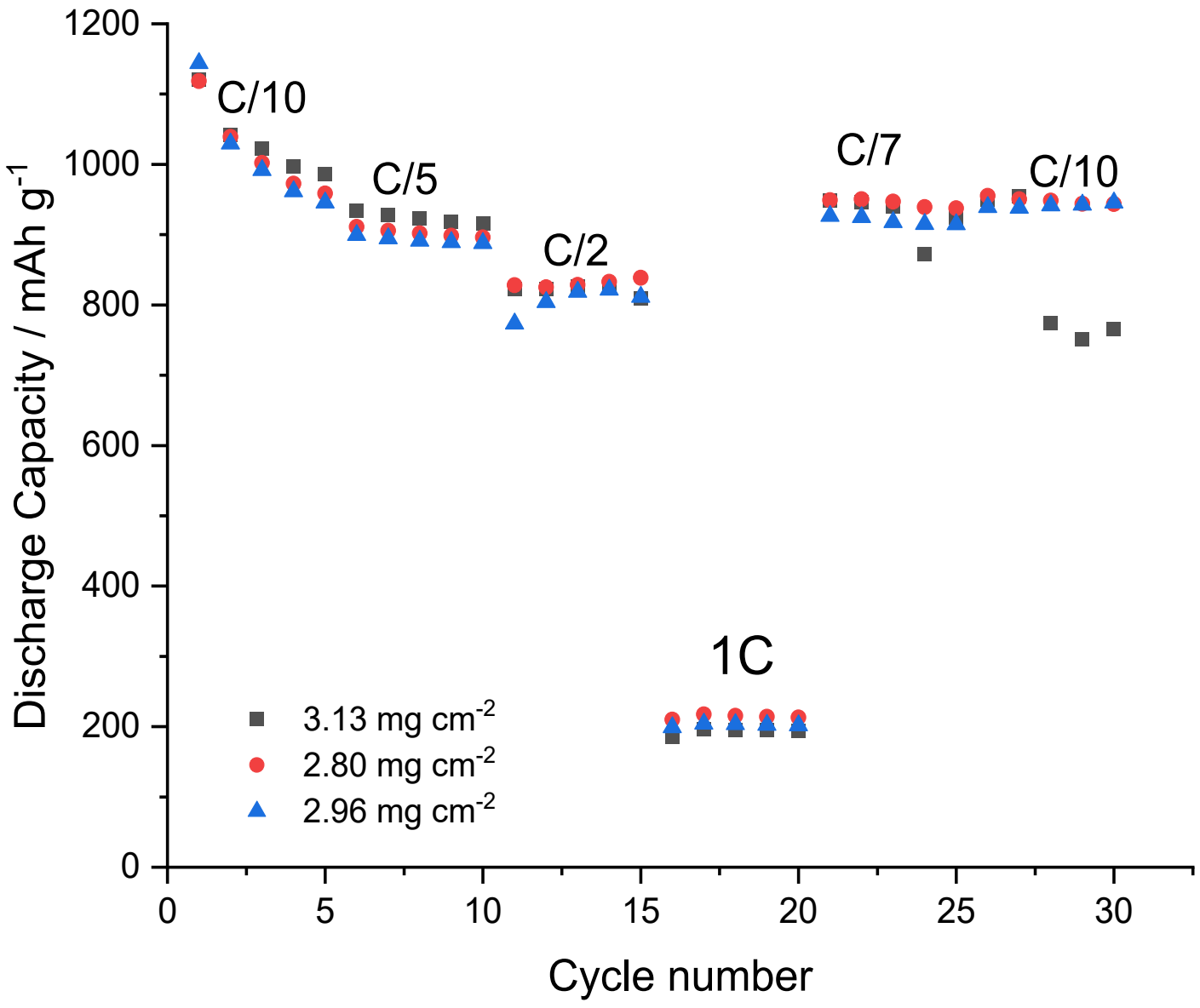

Figure S33. As in Figure S31 but in DOL:DME 1:1.

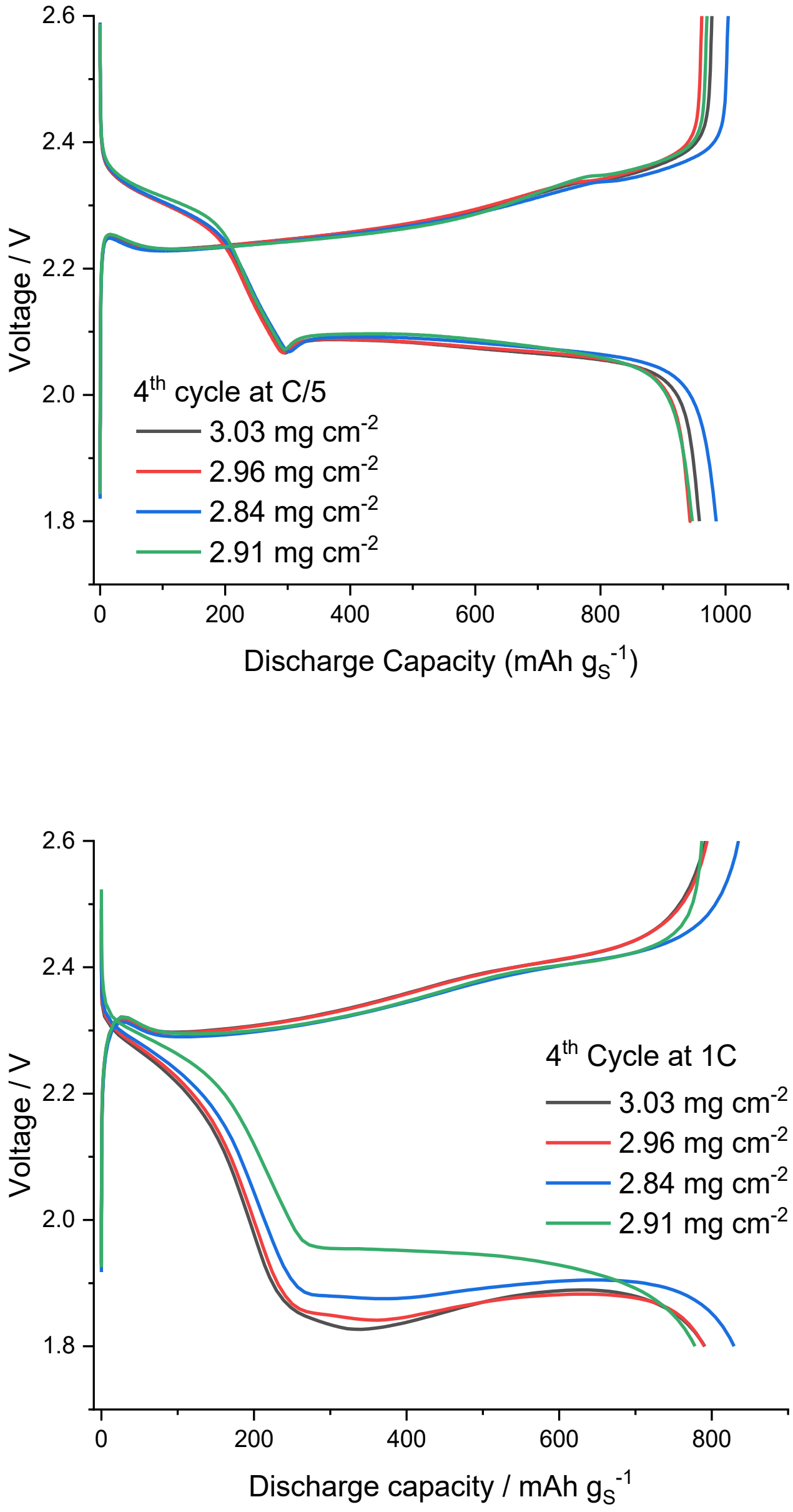

Figure S34. Voltage profiles during C/5 and 1C cycling of Li-S cells in 1M LiTFSI + 0.25M $LiNO_3$ in DOL:DME:TFMP 1:3:1. The loadings of the different cells are indicated in the legend.

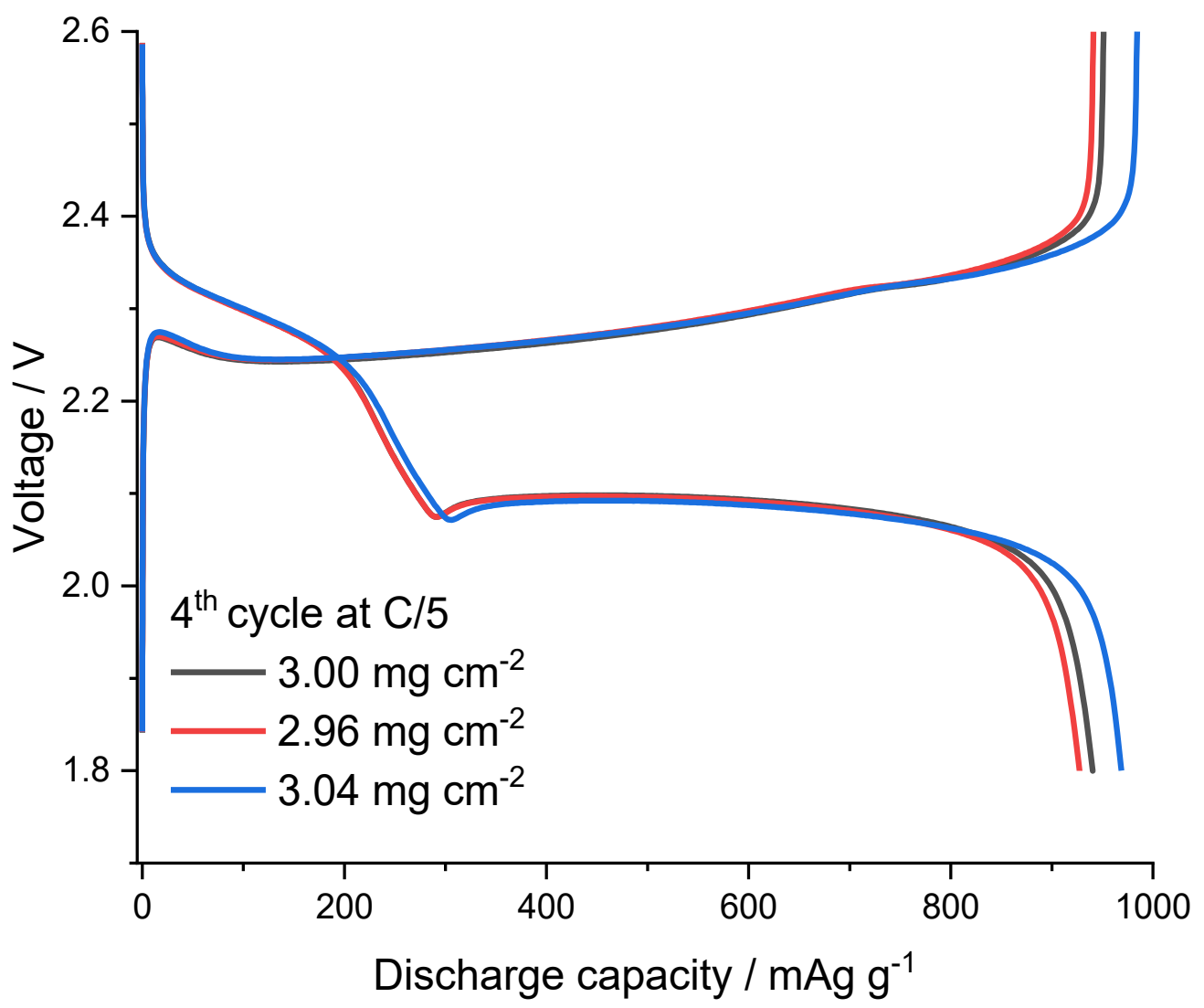

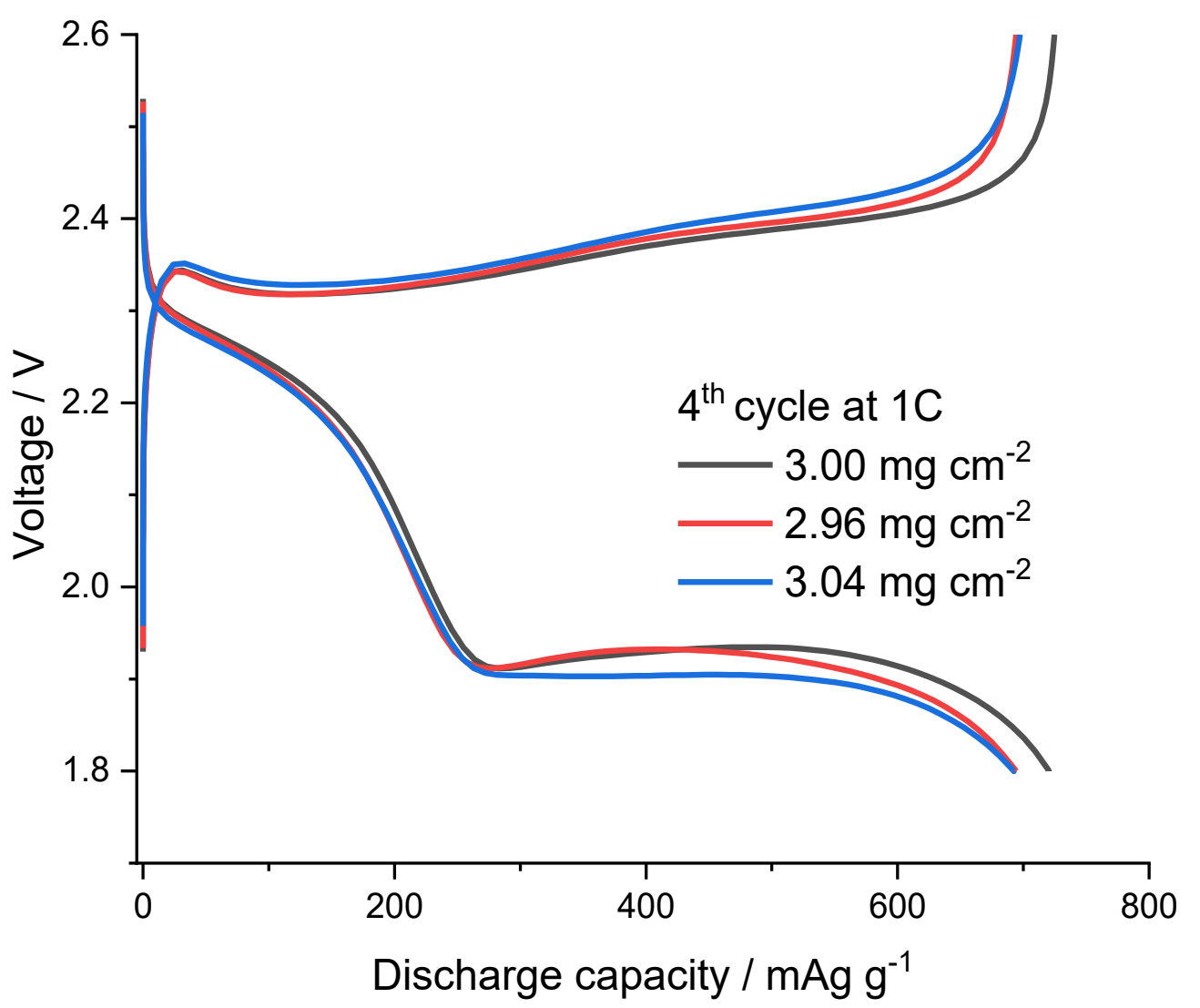

Figure S35. As in Figure S34 but in DME:TFMP 3:2.

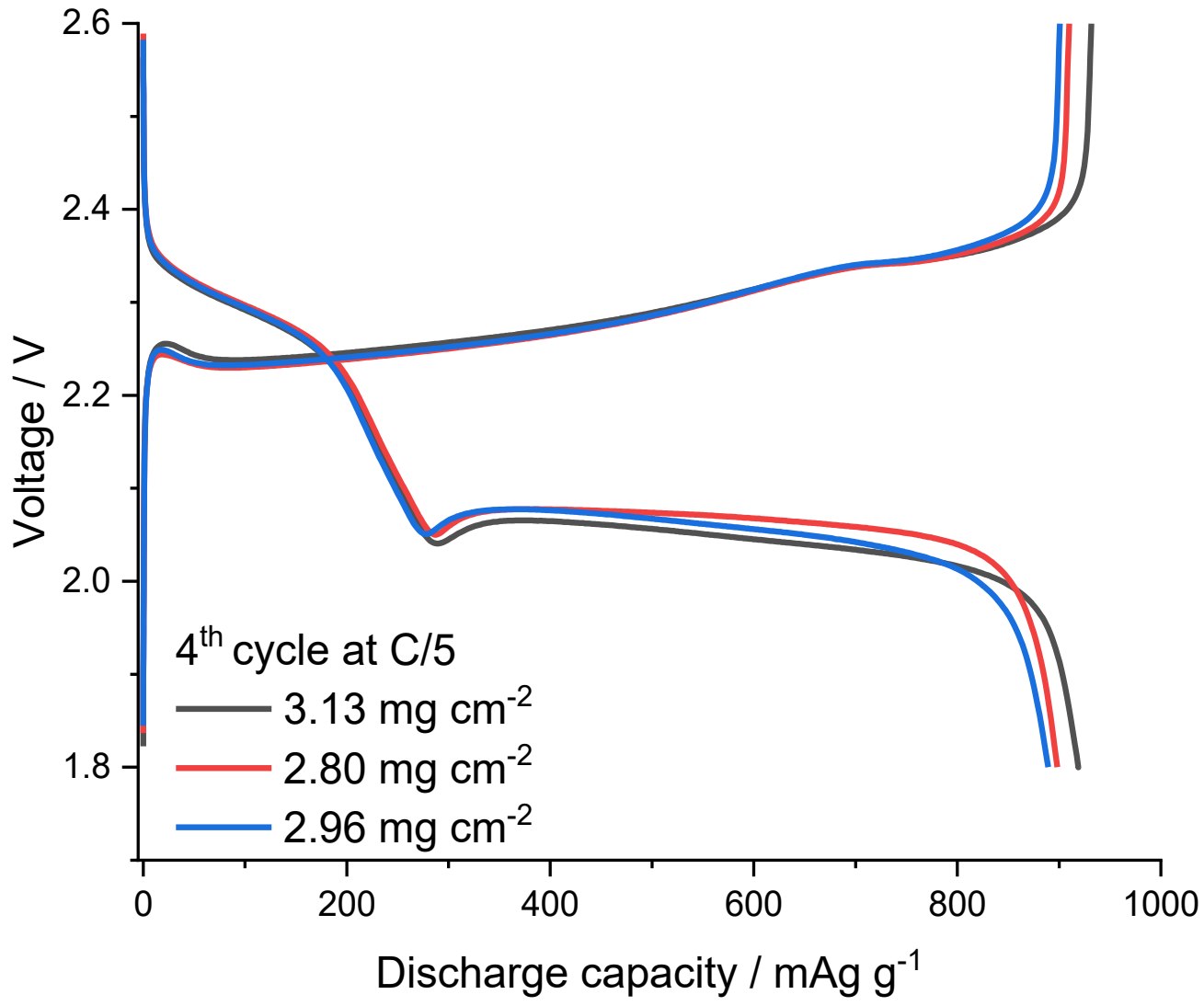

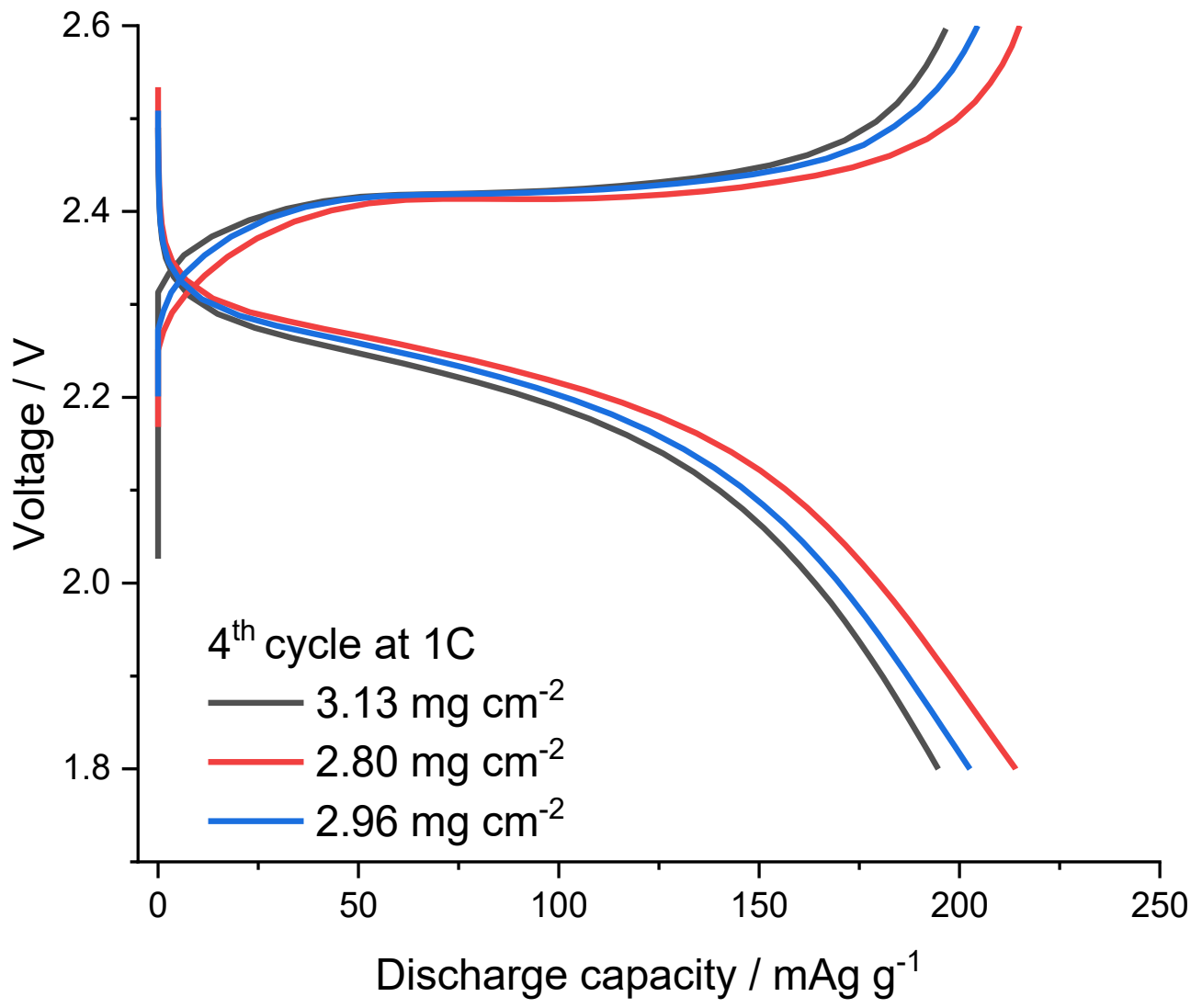

Figure S36. As in Figure S34 but in DOL:DME 1:1.

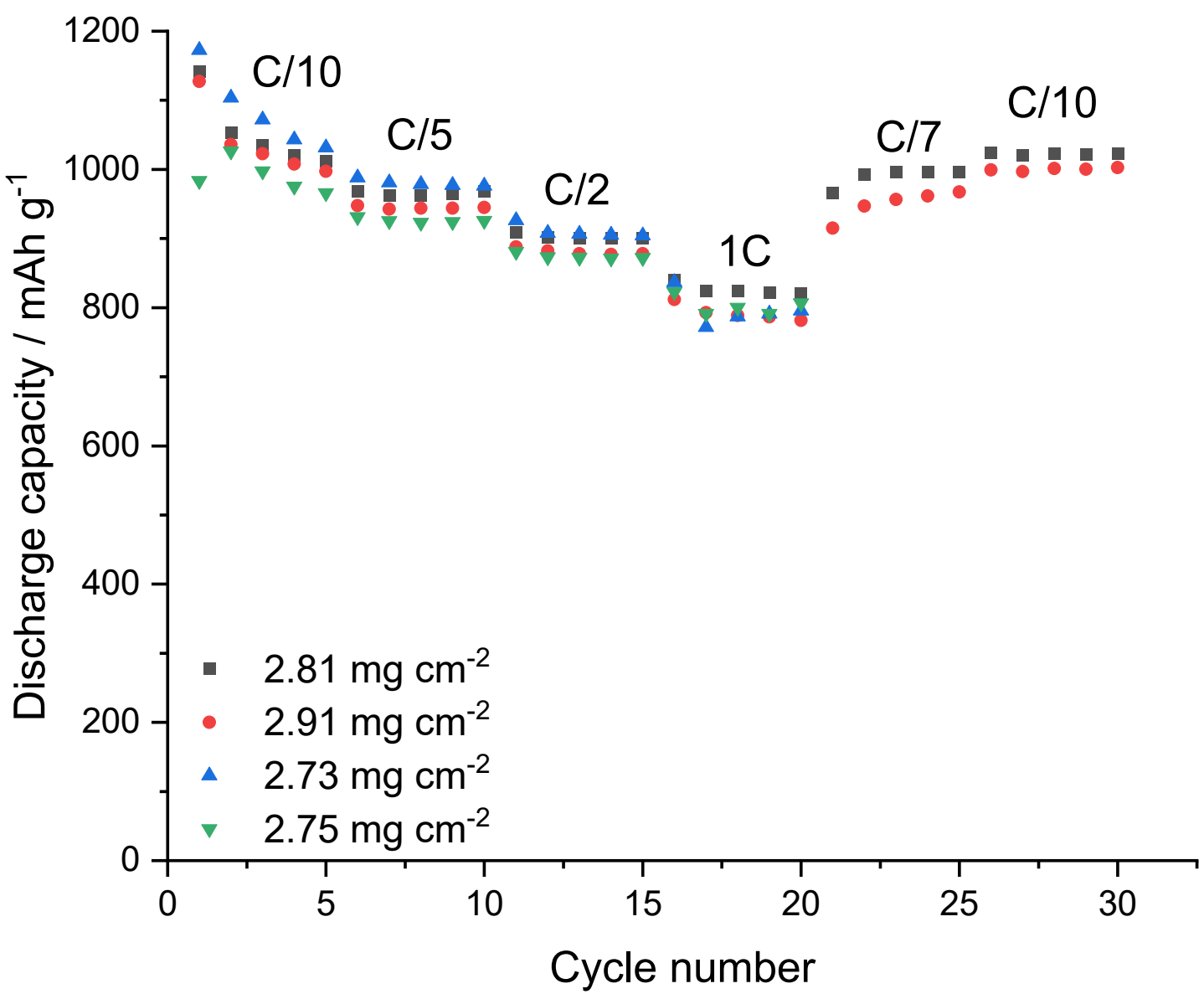

Figure S37. As in Figure S31 but in DOL:DME:TFEP 1:3:1.

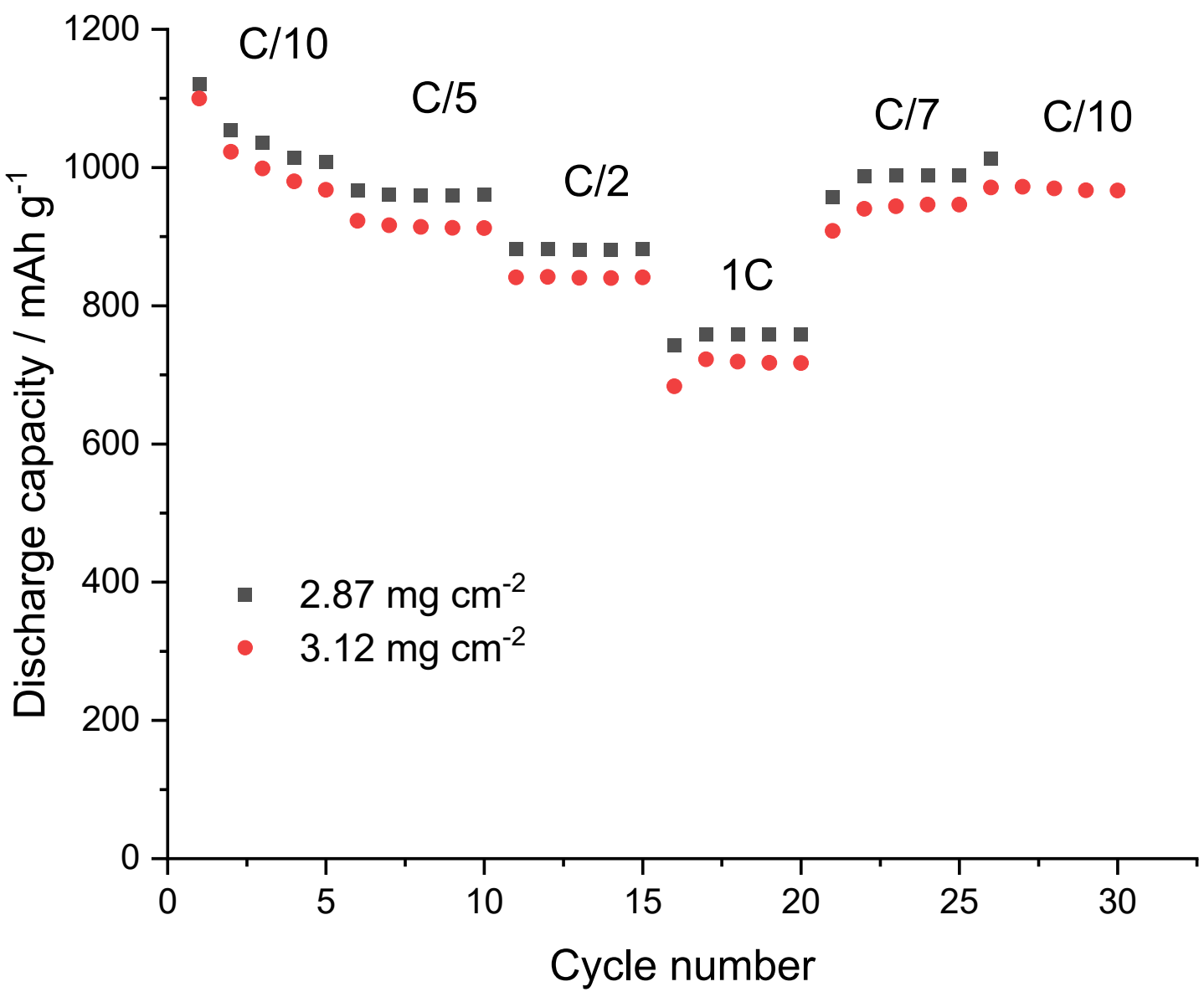

Figure S38. As in Figure S31 but in DOL:DME:TFPP in 1:3:1.

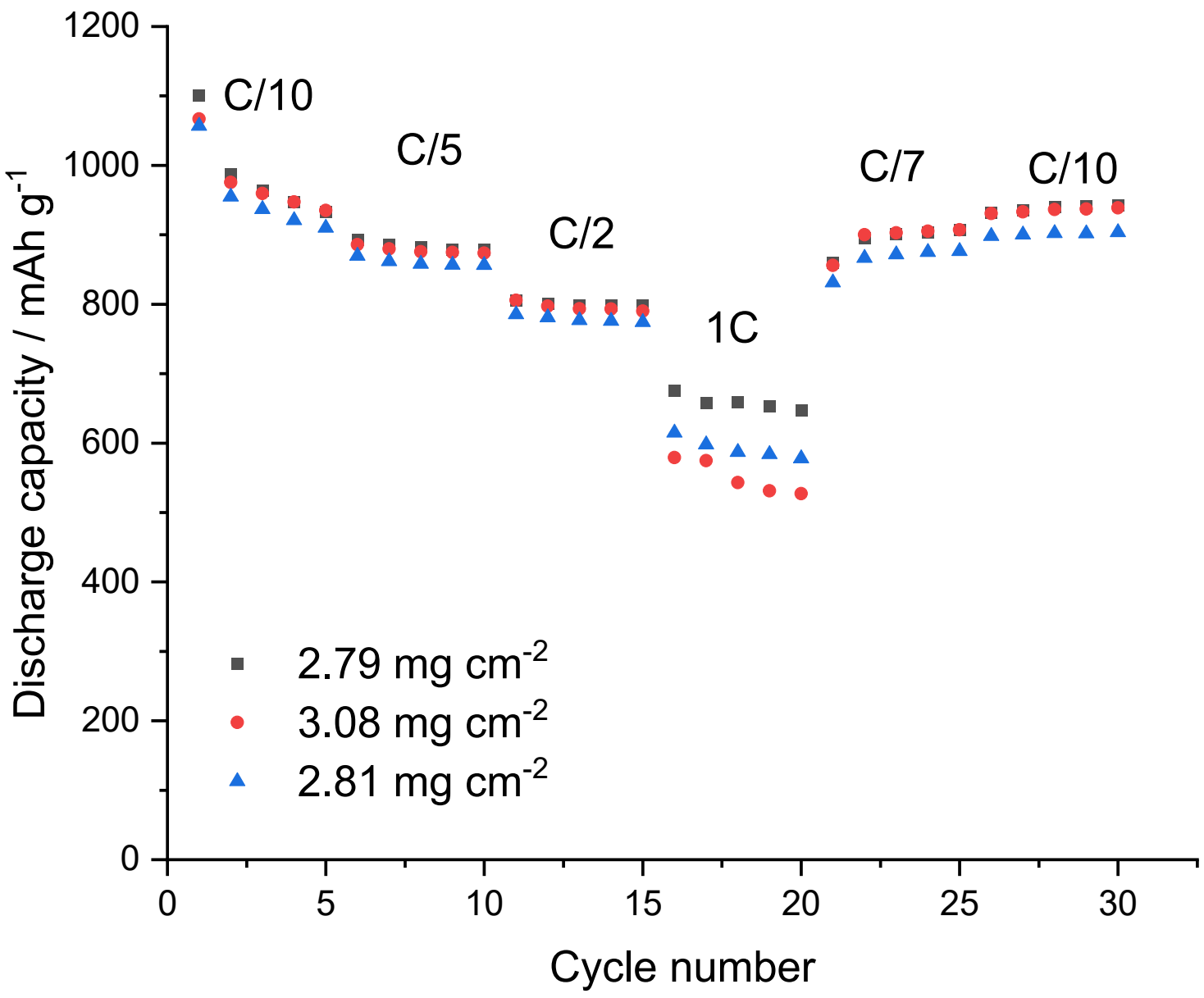

Figure 39. As in Figure S31 but in DOL:DME:TTE in 1:3:1.

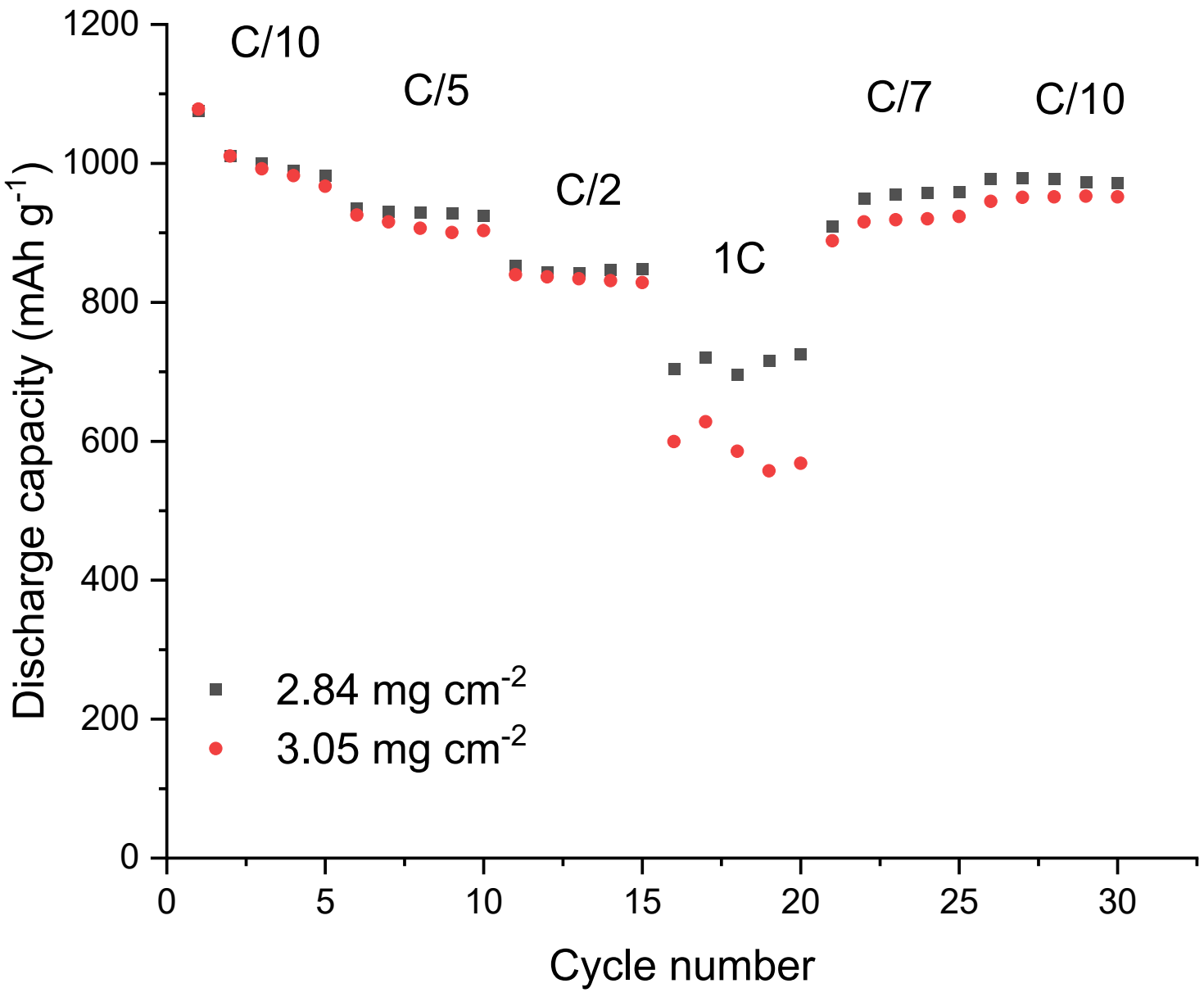

Figure 40. As in Figure S31 but in DOL:DME:BTFE in 1:3:1.

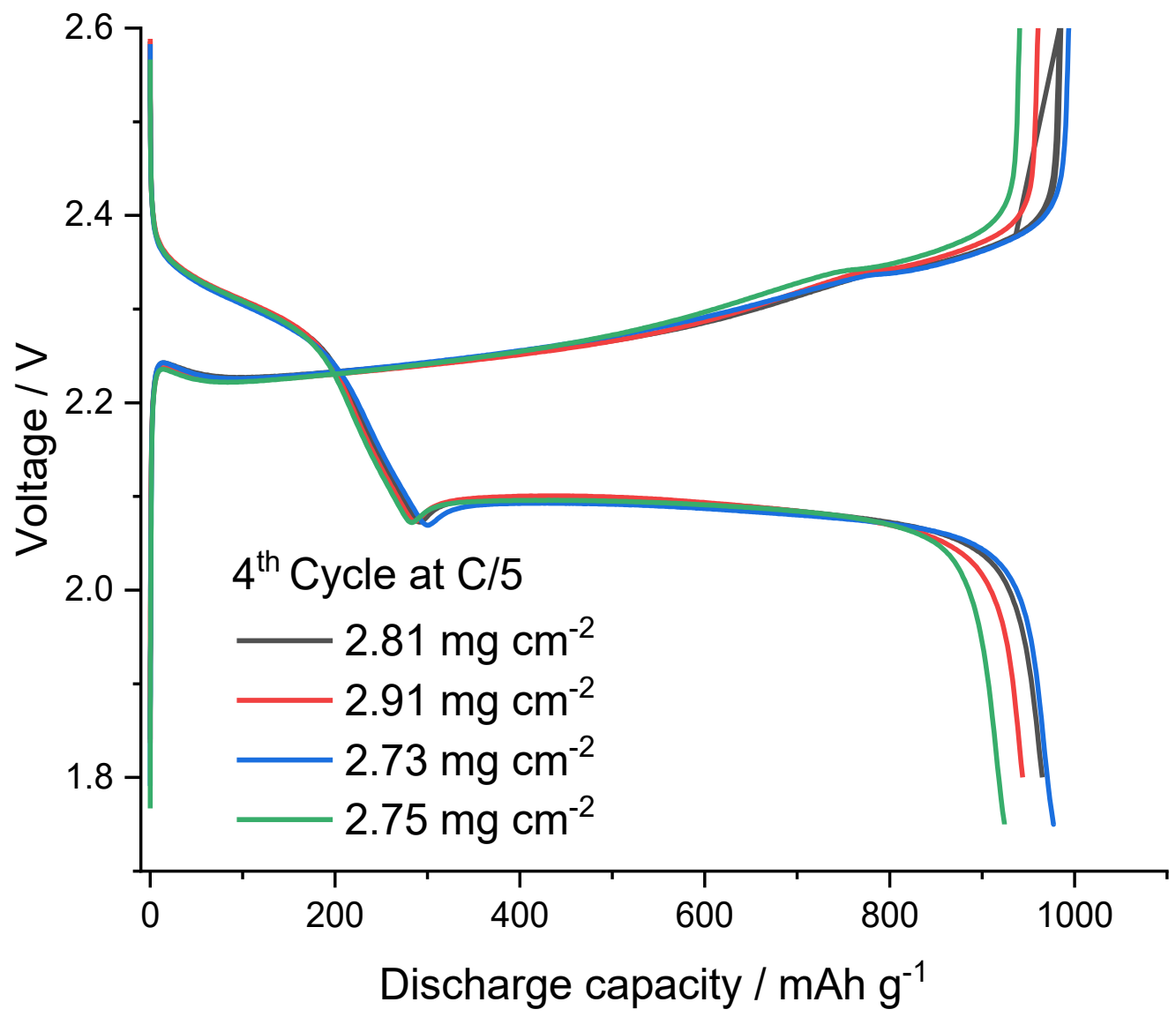

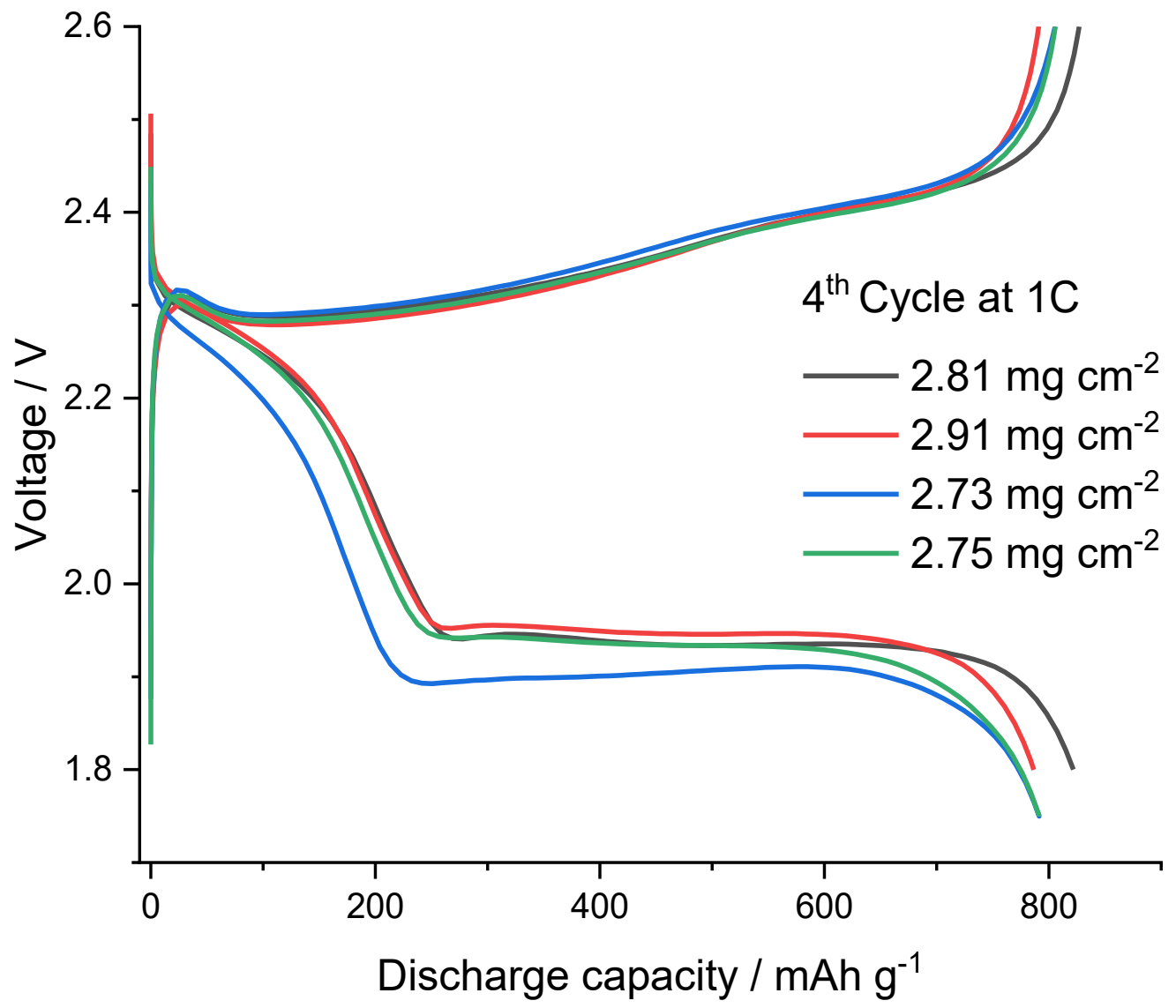

Figure S41. As in Figure S34 but in DOL:DME:TFEP 1:3:1.

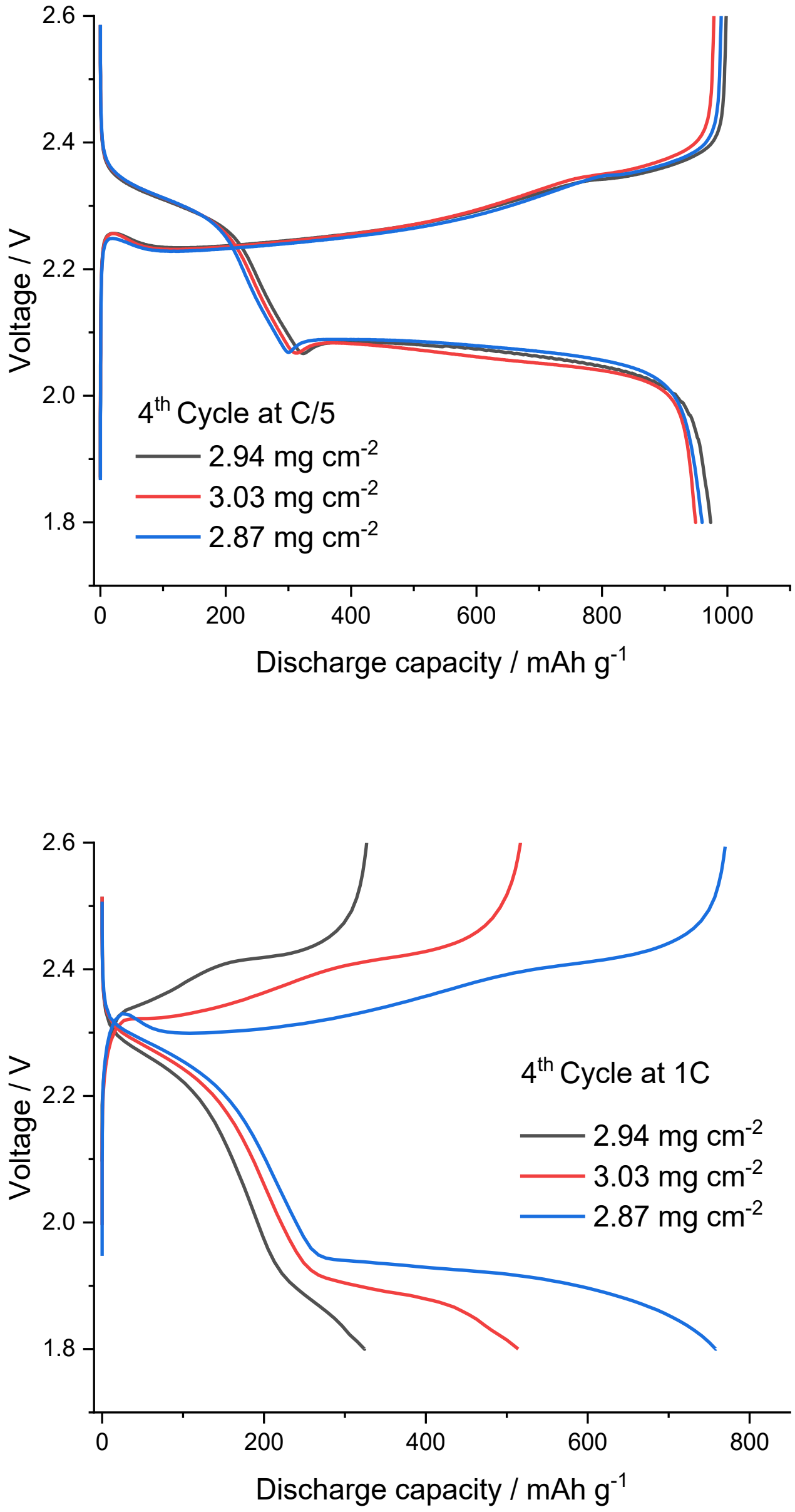

Figure S42. As in Figure S34 but in DOL:DME:TFPP in 1:3:1.

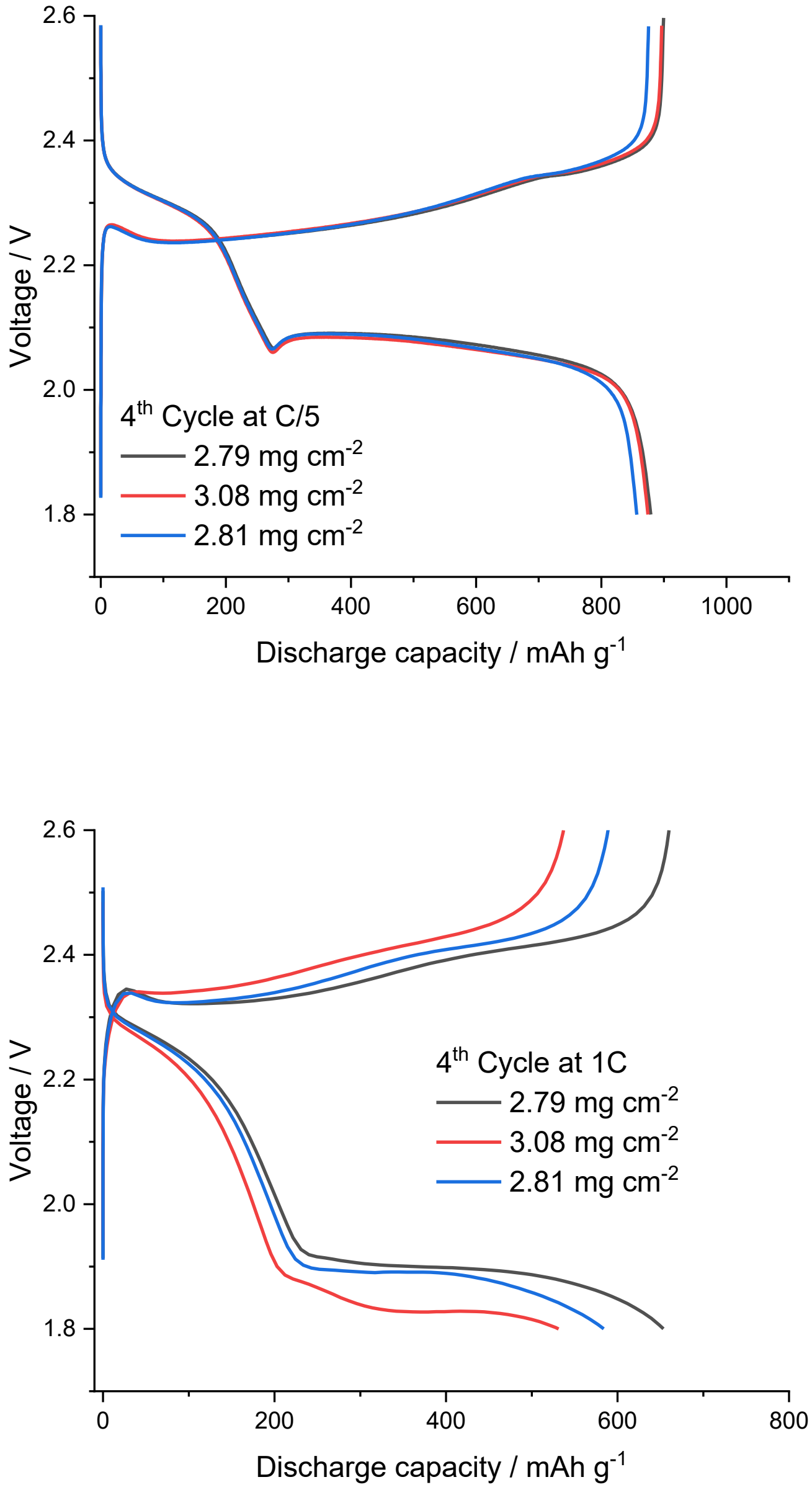

Figure S43. As in Figure S34 but in DOL:DME:TTE in 1:3:1.

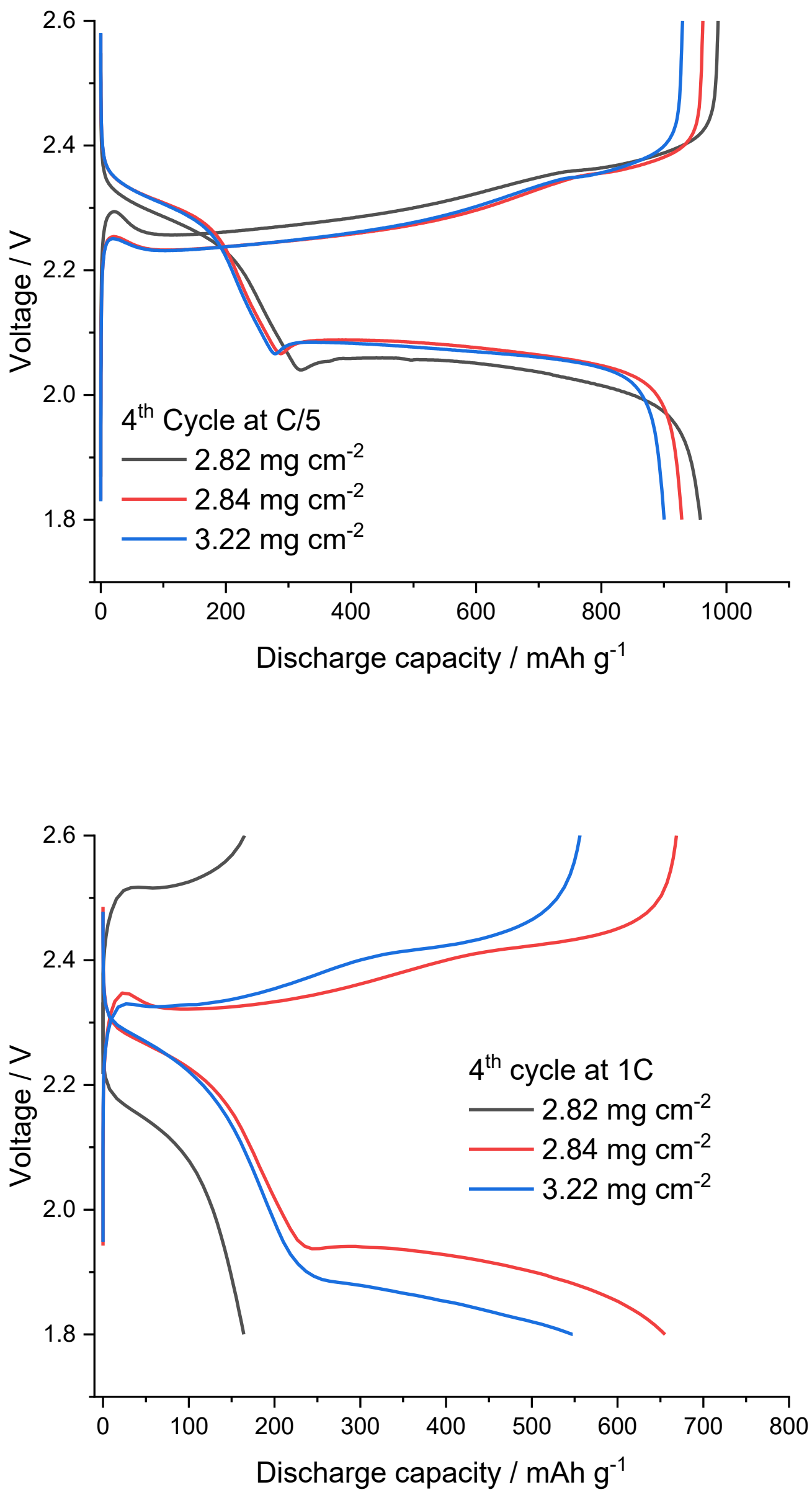

Figure S44. As in Figure S34 but in DOL:DME:BTFE in 1:3:1.

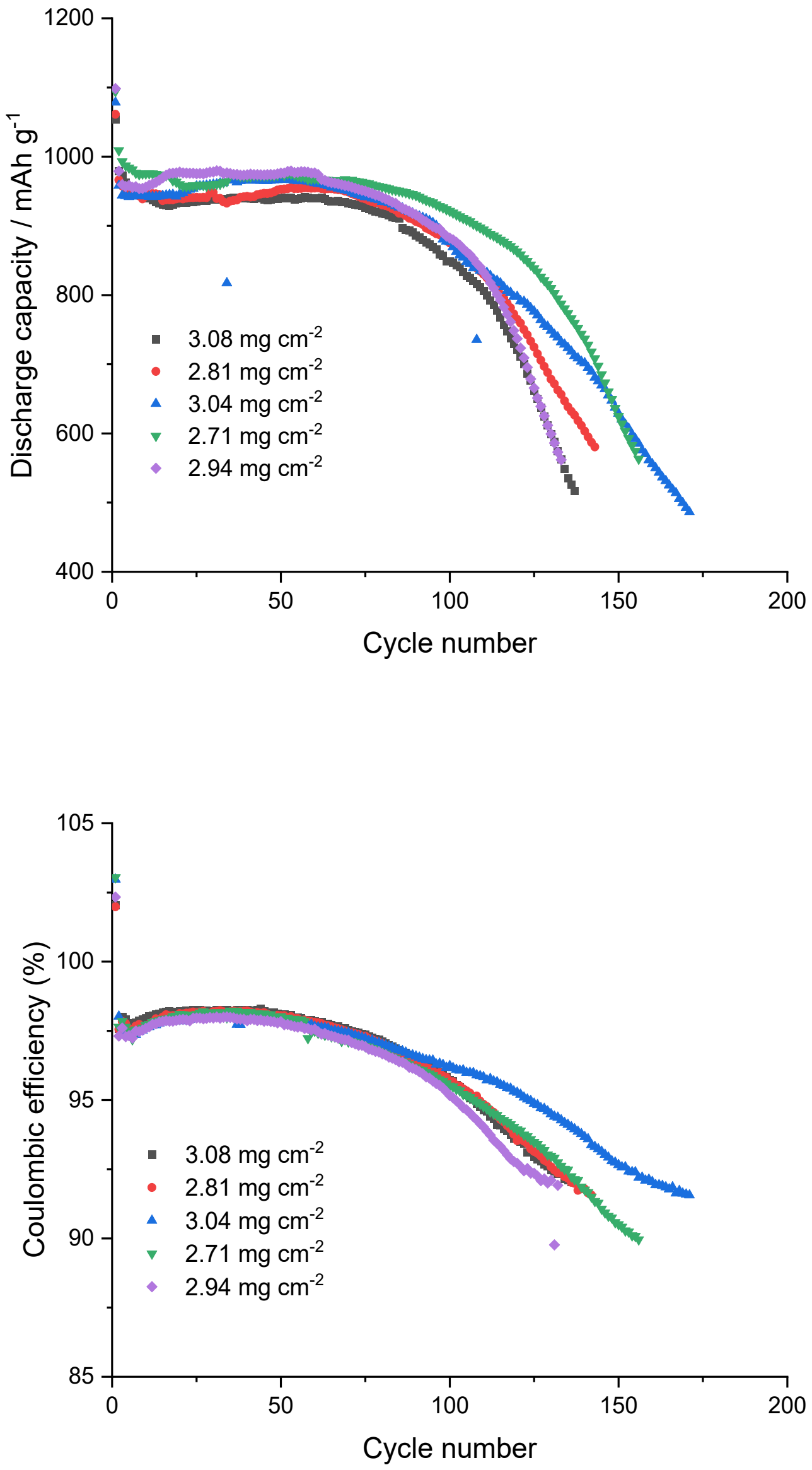

Figure S45. As in Figure S25 but DOL:DME:TFEP 1:3:1.

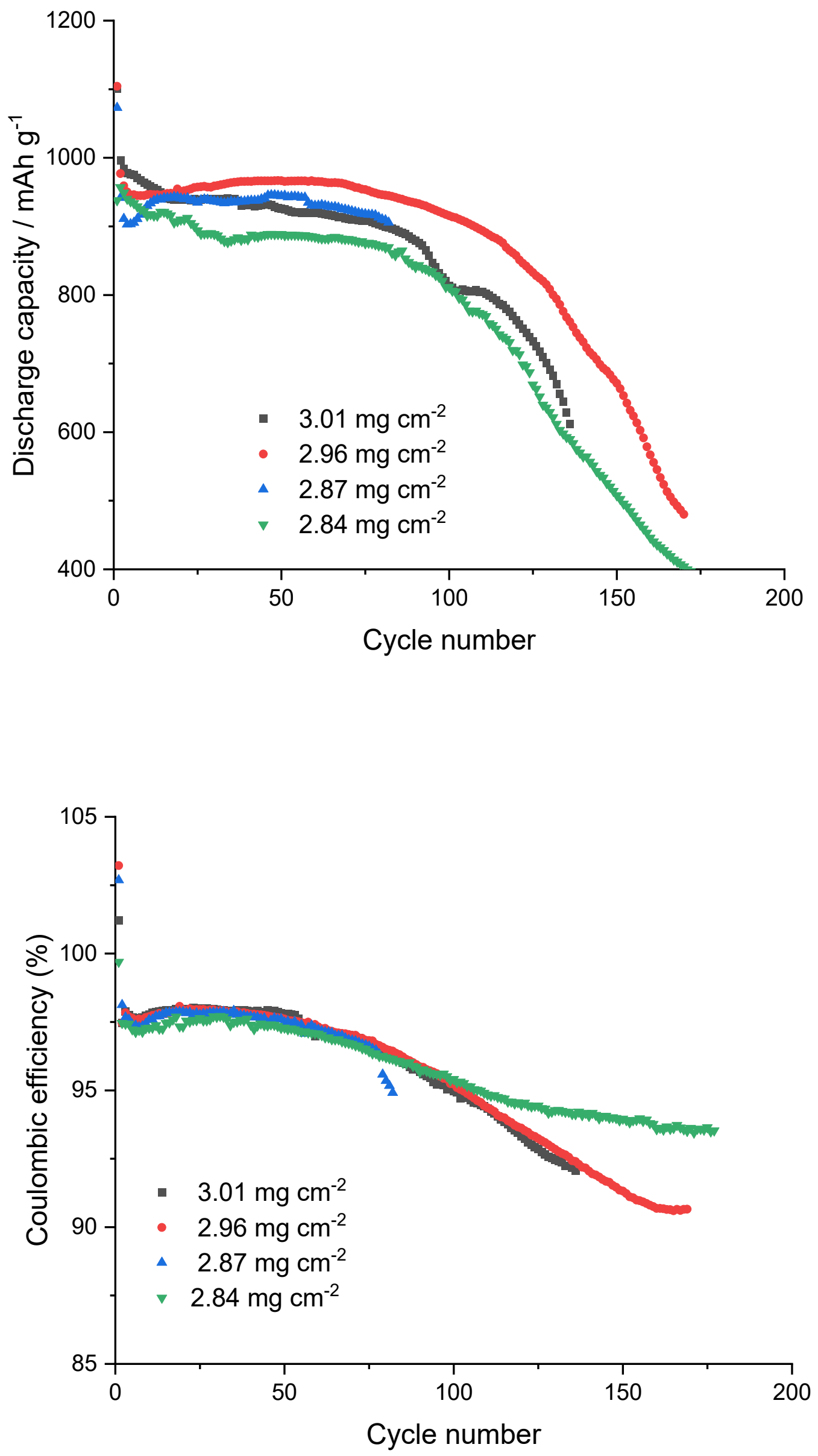

Figure S46. As in Figure S25 but in DOL:DME:FDMB 1:3:1.

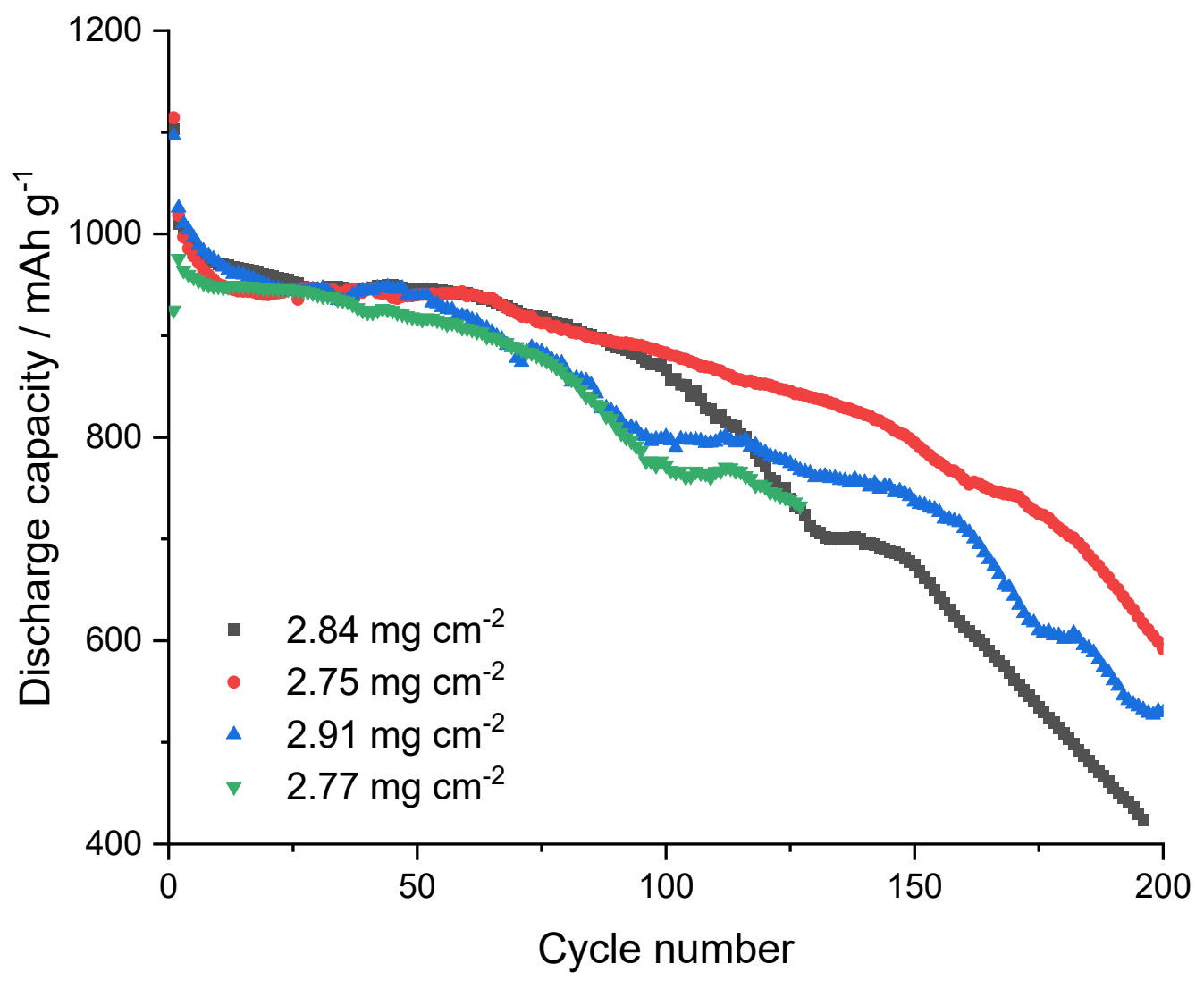

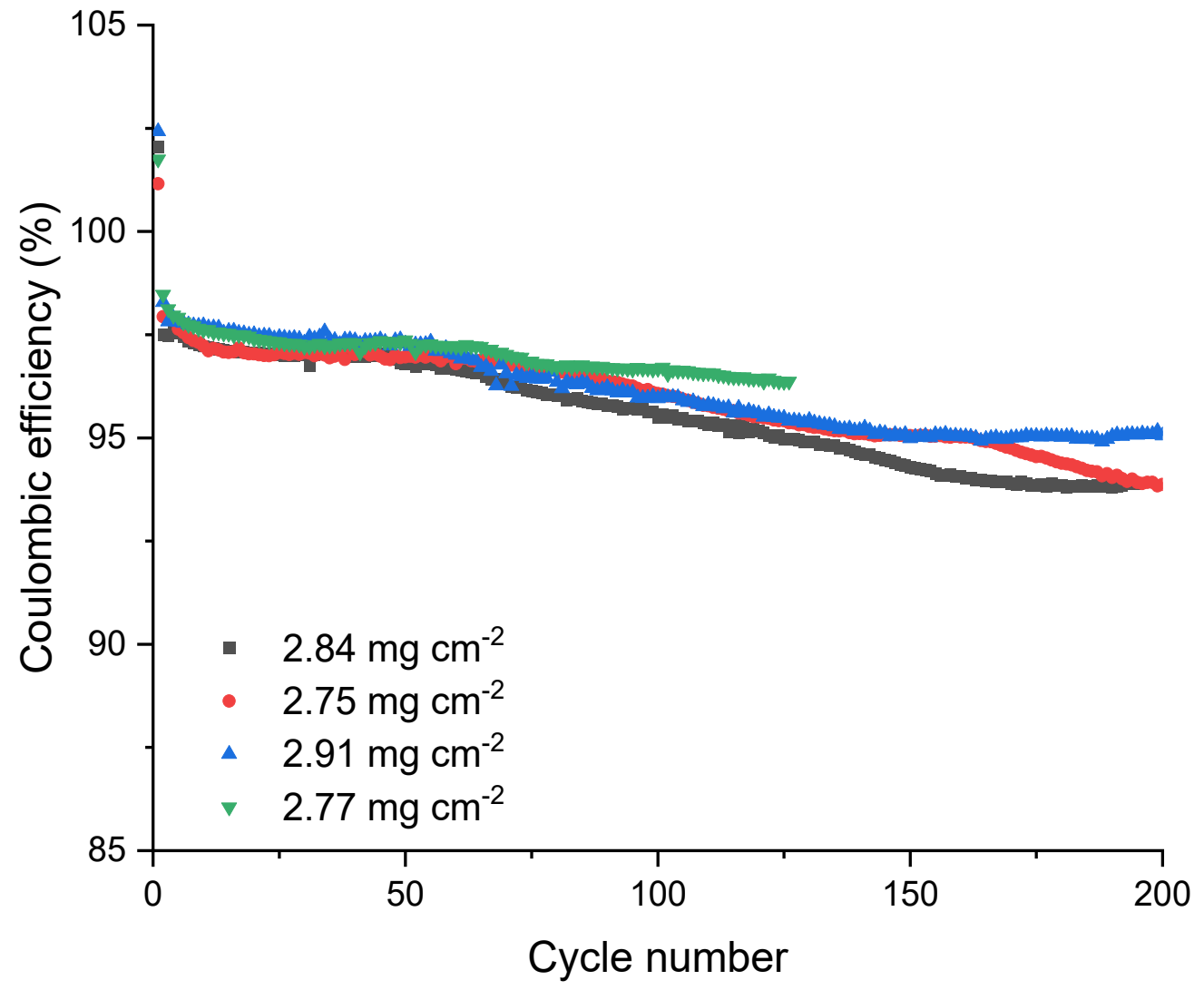

Figure S47. As in Figure S25 but in DOL:DME:BTFE 1:3:1.

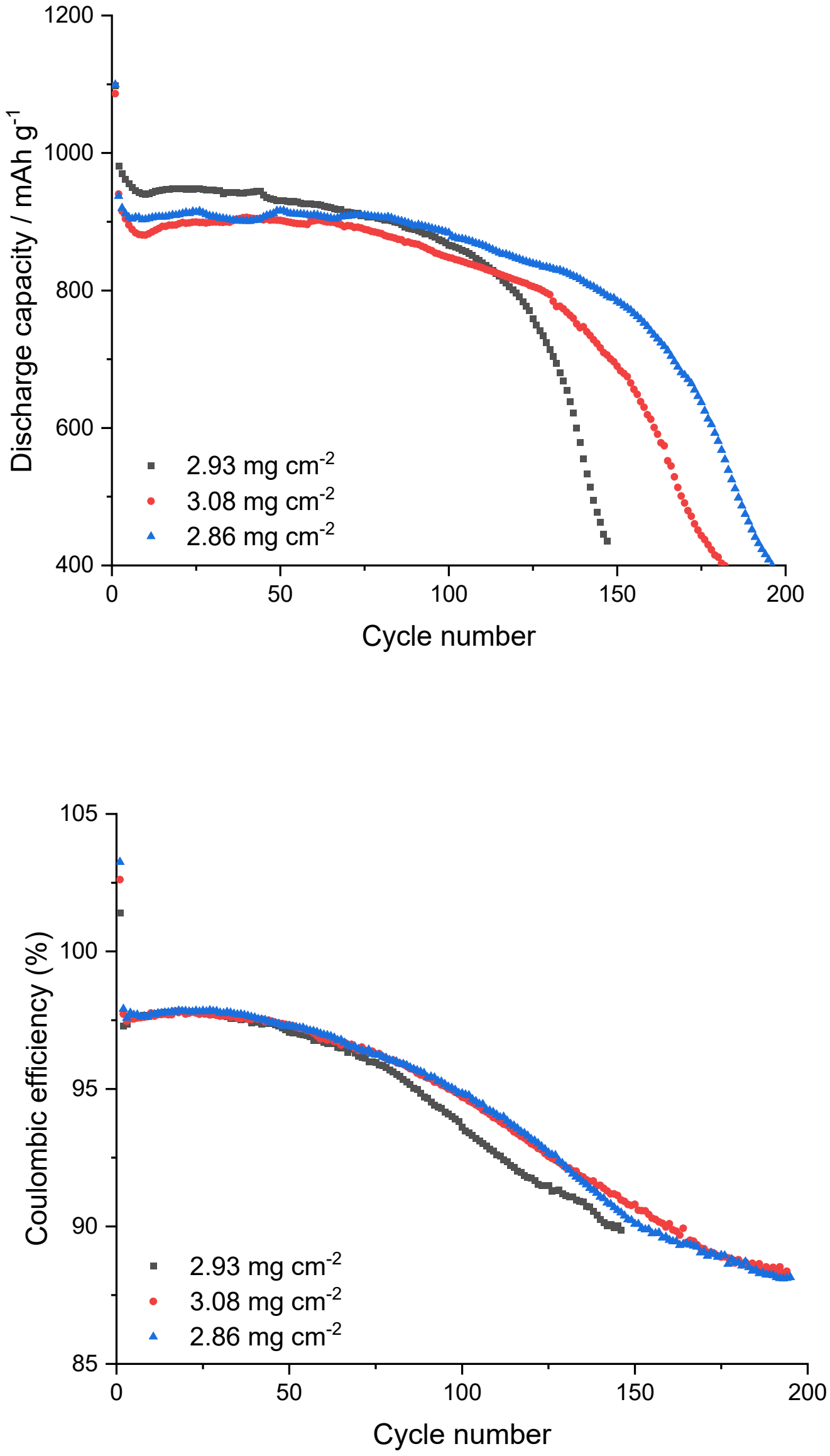

Figure S48. As in Figure S25 but in DOL:DME:TTE in 1:3:1.

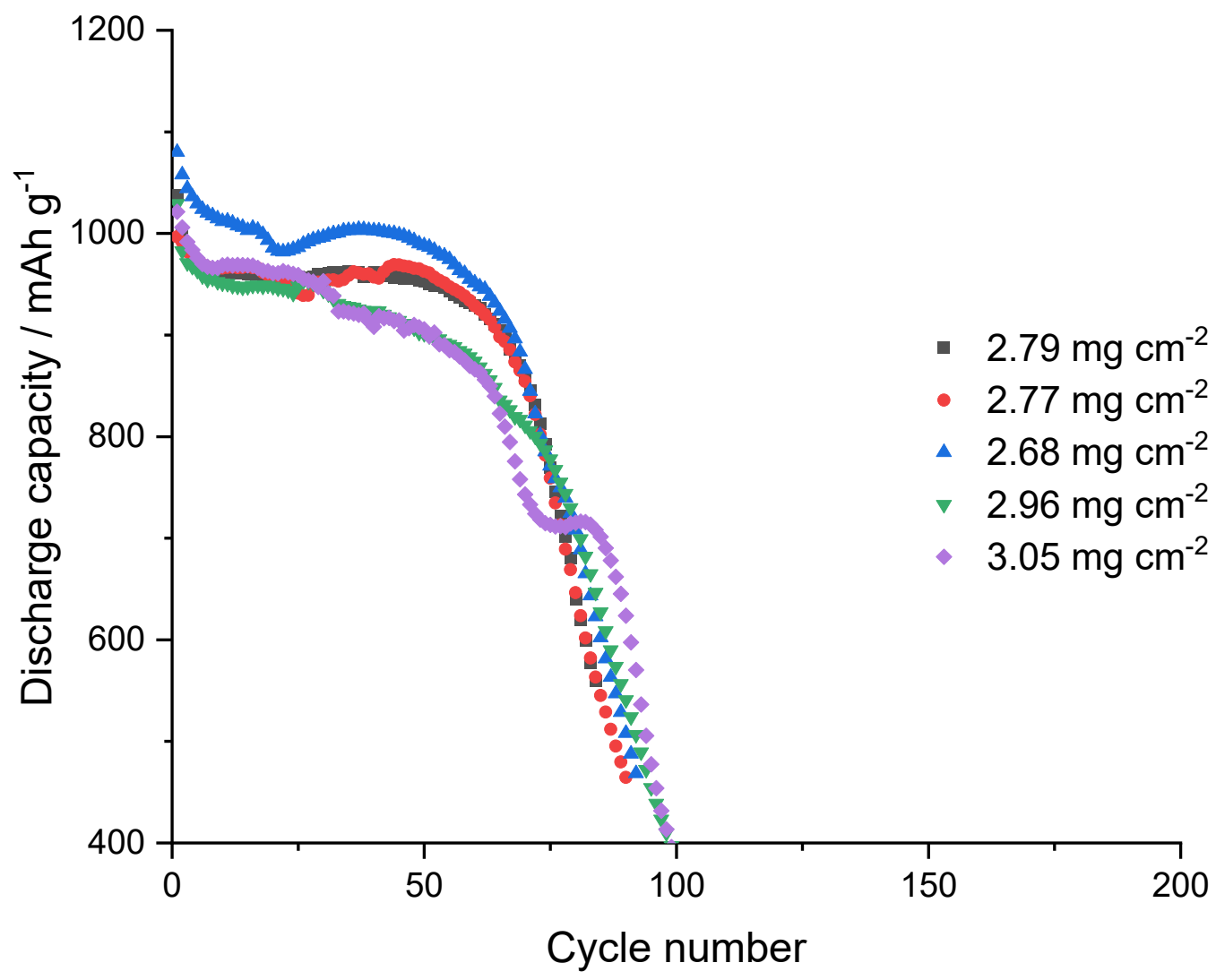

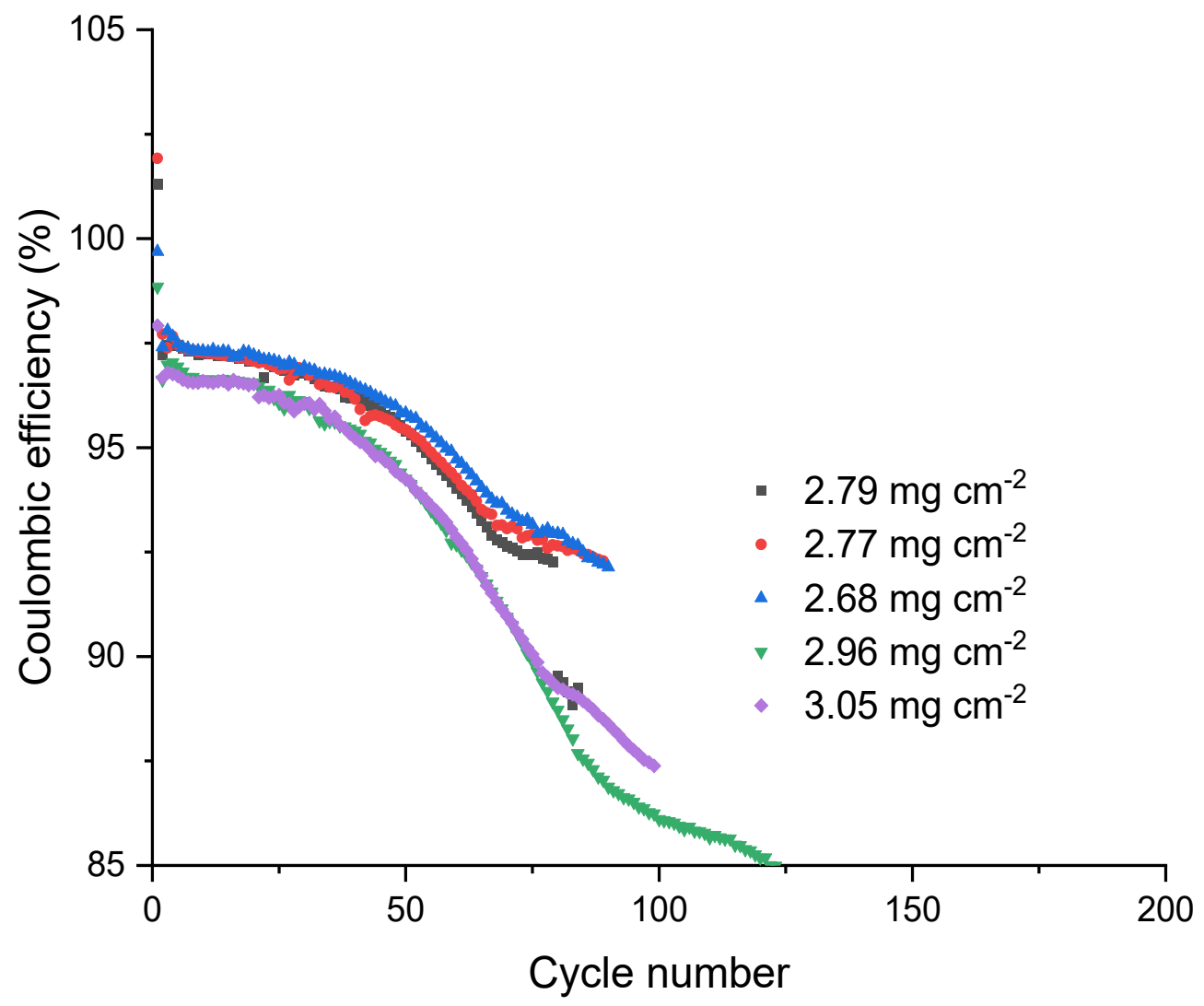

Figure S49. As in Figure S25 but in DOL:DME:ETFE 1:3:1.

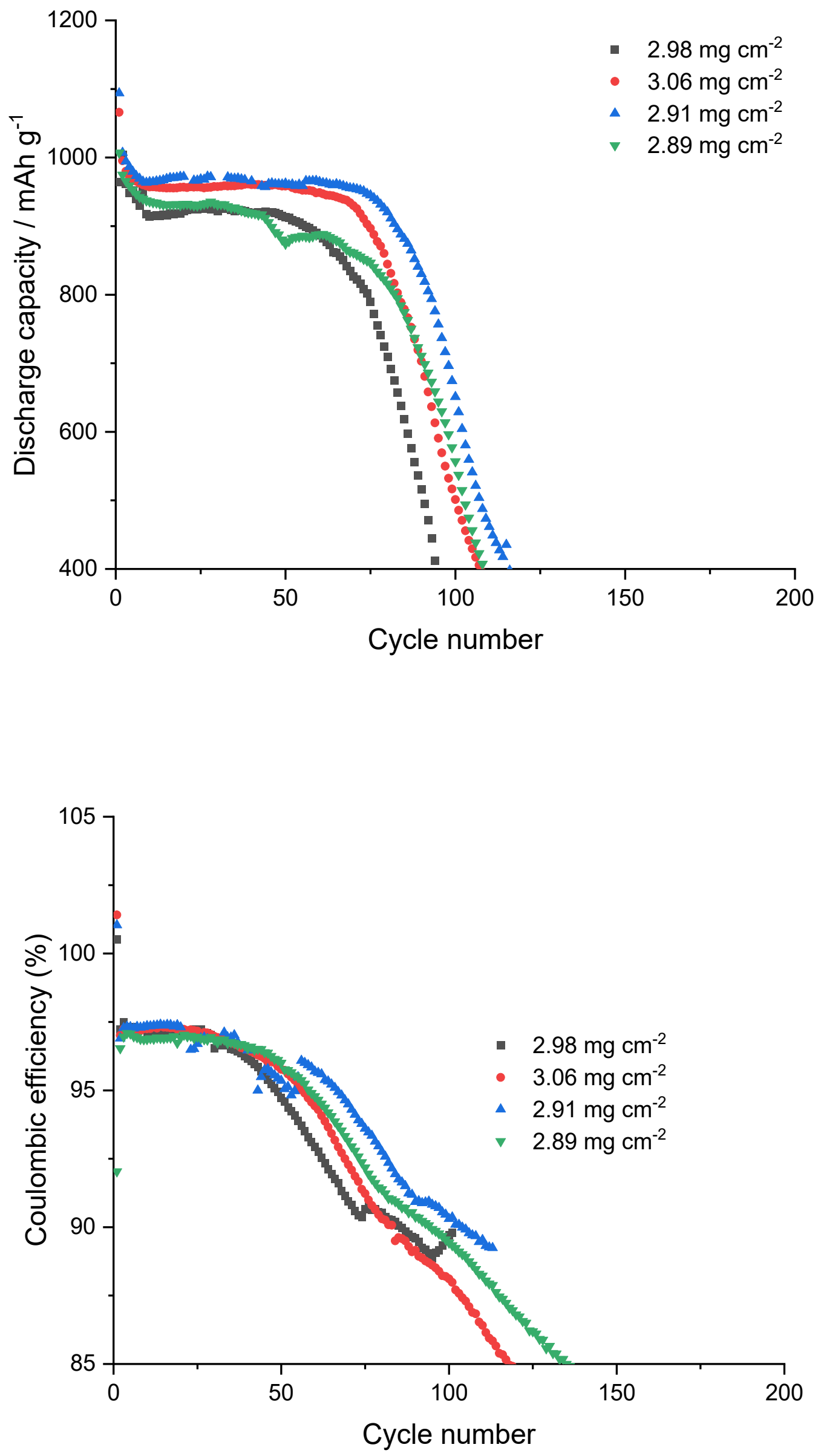

Figure S50. As in Figure S25 but in DOL:DME:TFPP in 1:3:1.

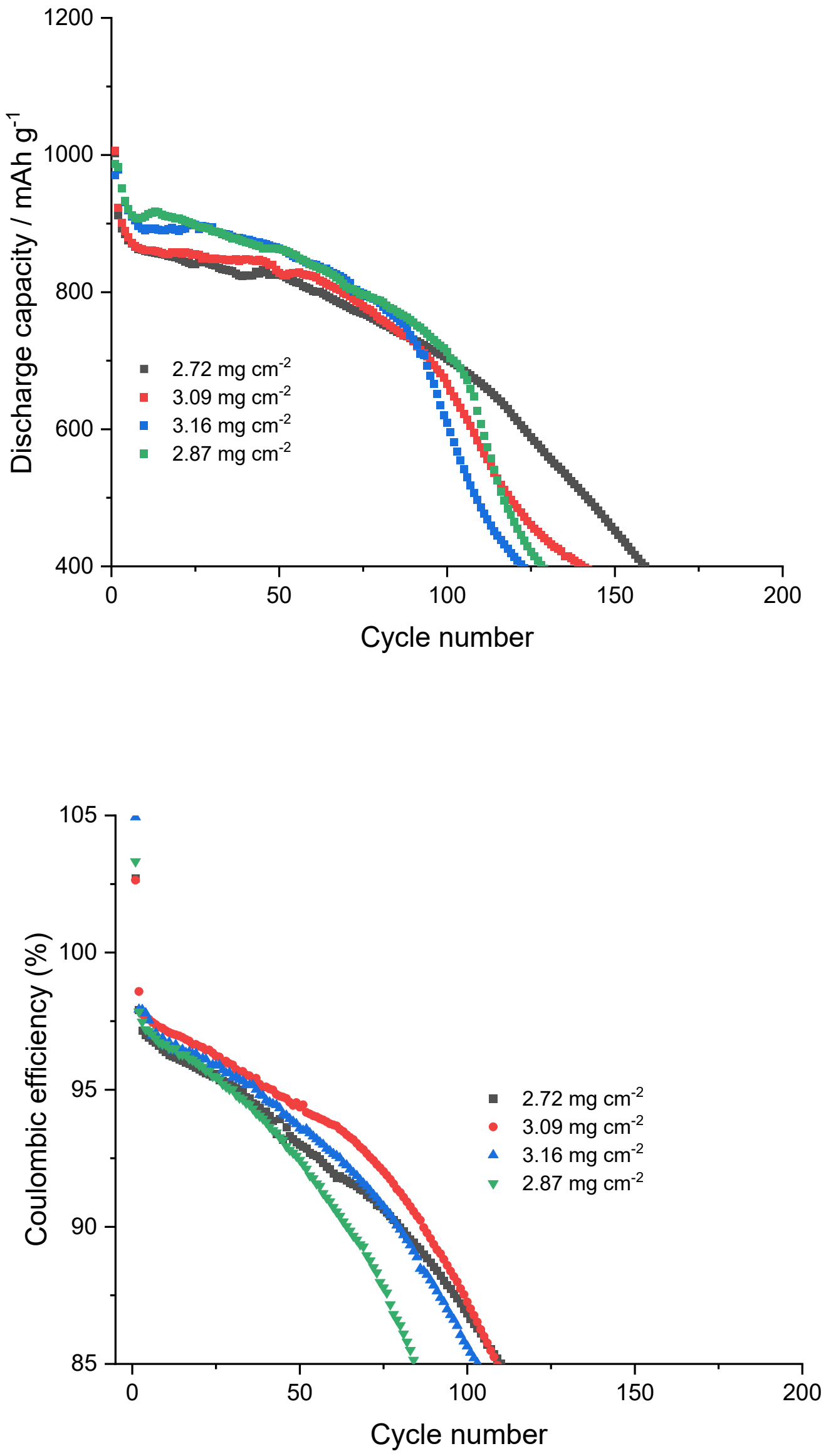

Figure S51. As in Figure S25 but in DOL:DME:TFEE 1:3:1.

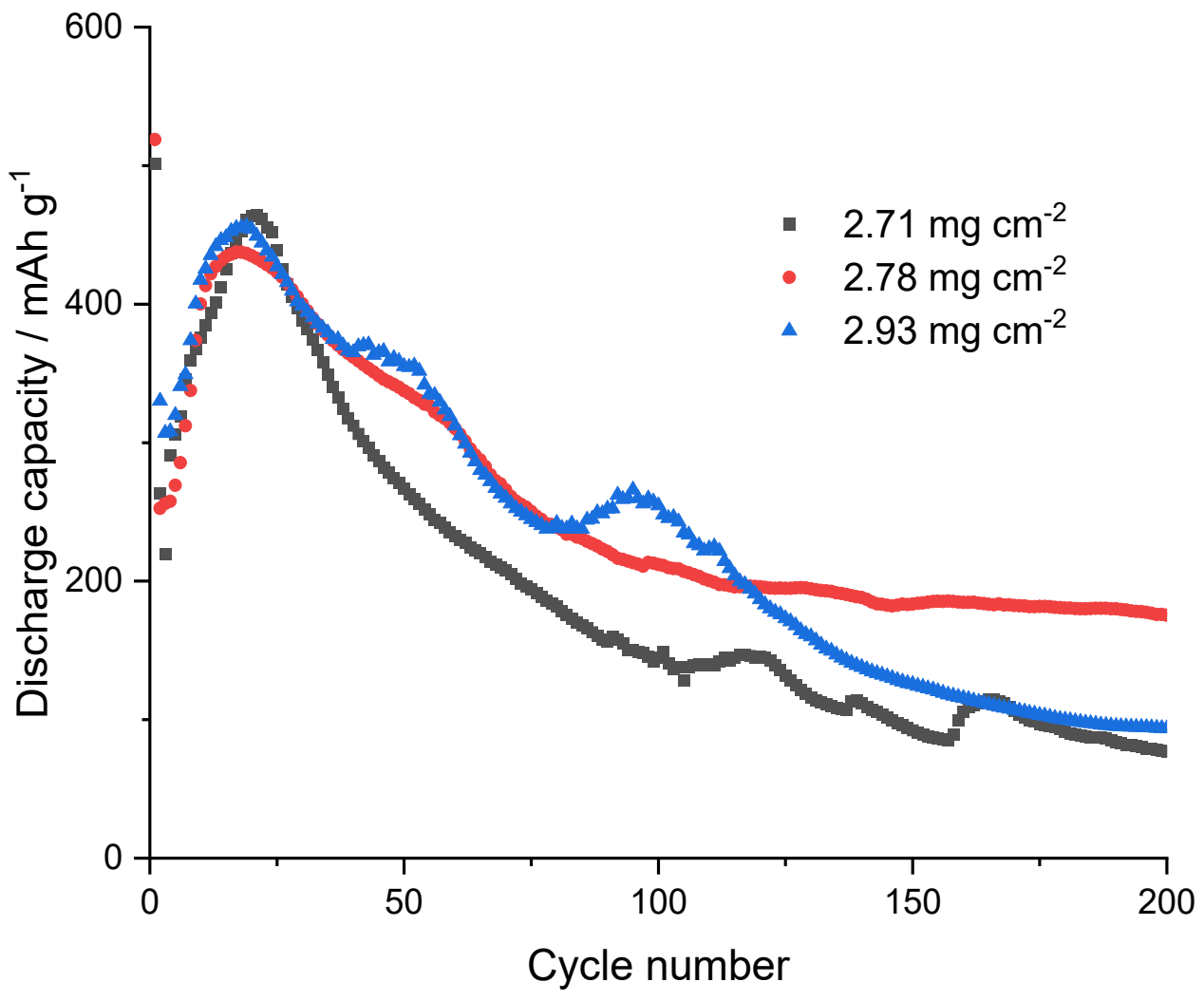

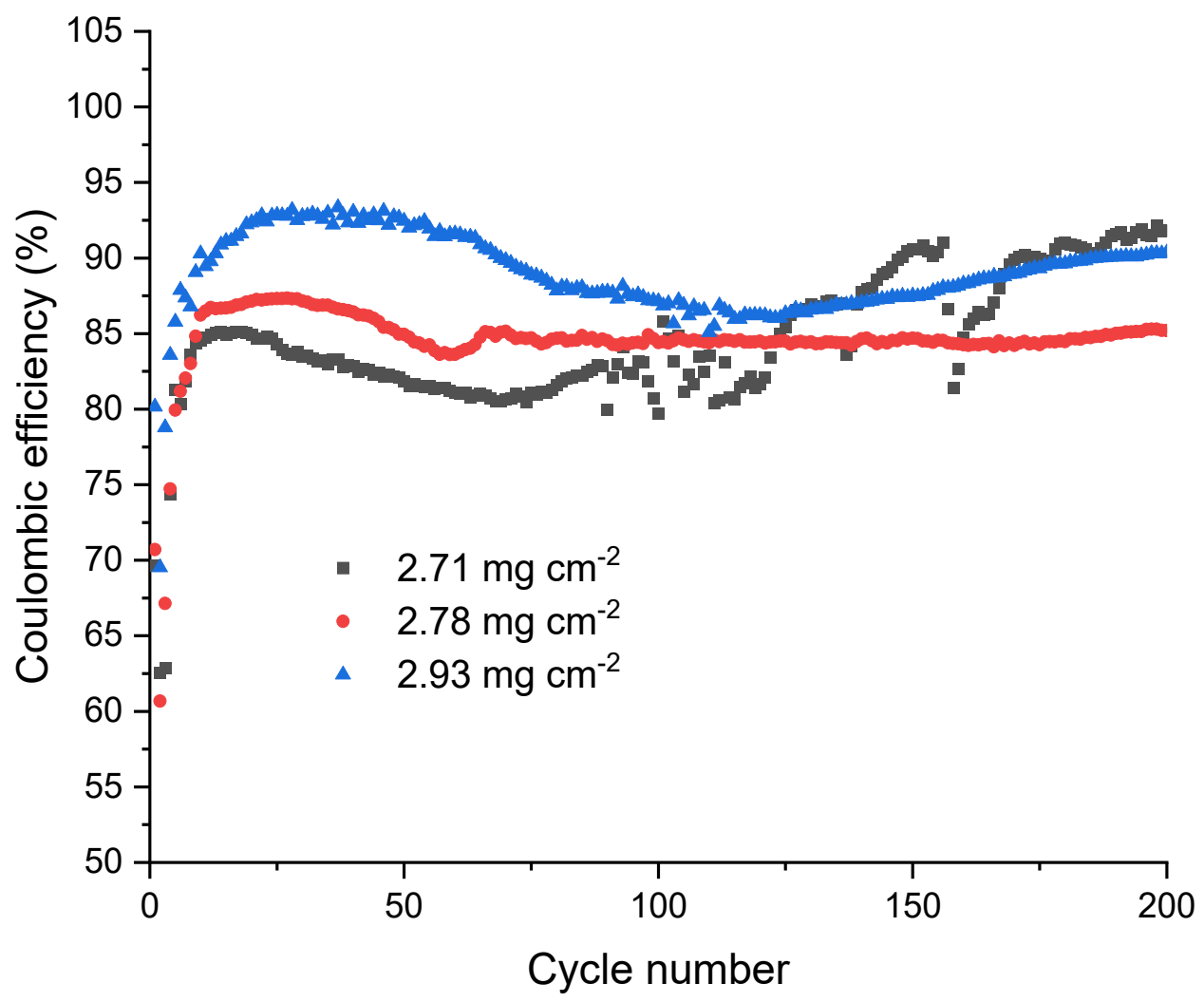

Figure S52. As in Figure S25 but in DOL:DME:TFFMP 1:3:1. Note the use of a different y-scale, compared to other Figures.

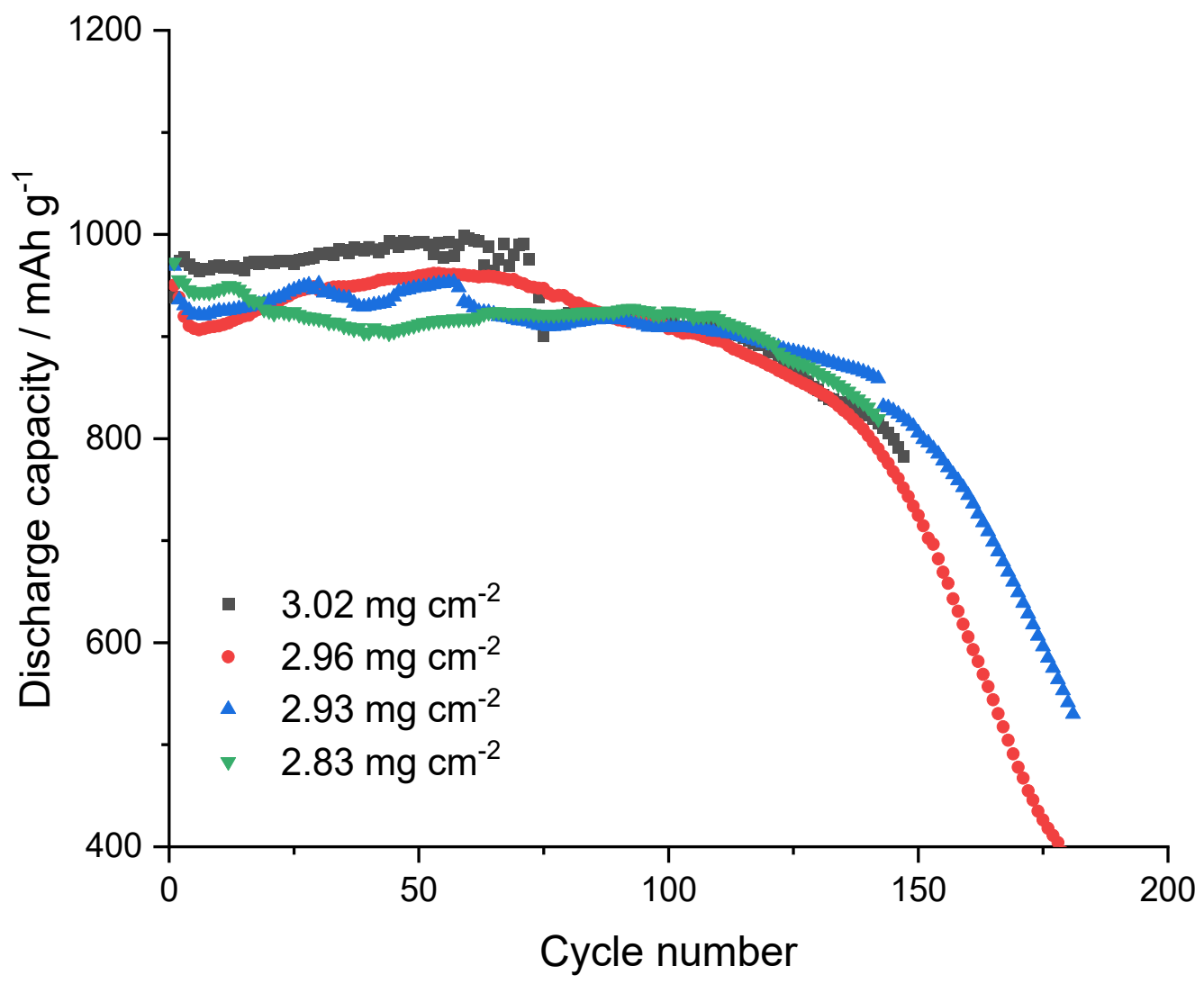

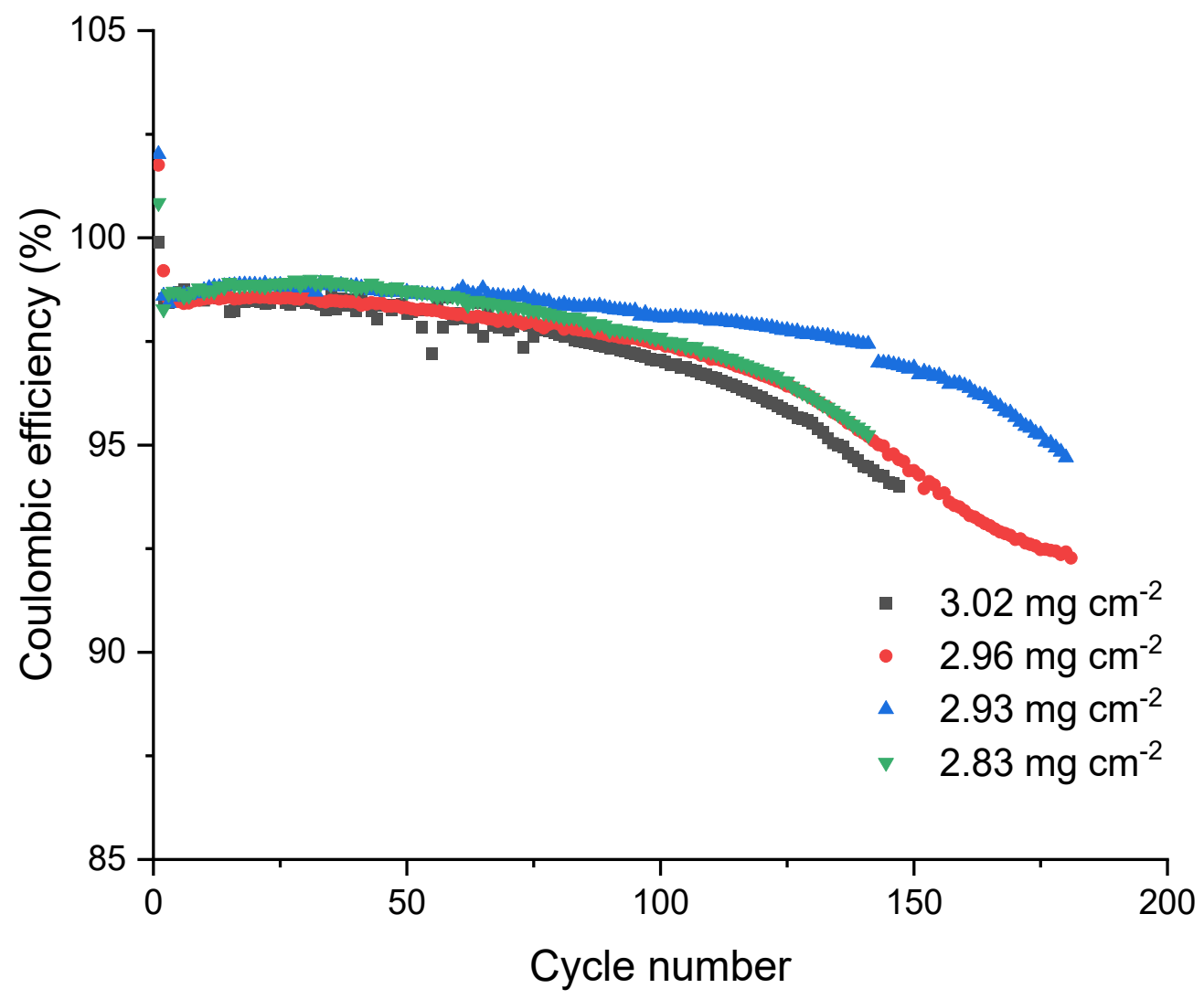

Figure S53. As in Figure S25 but in DME:TFEP 3:2.

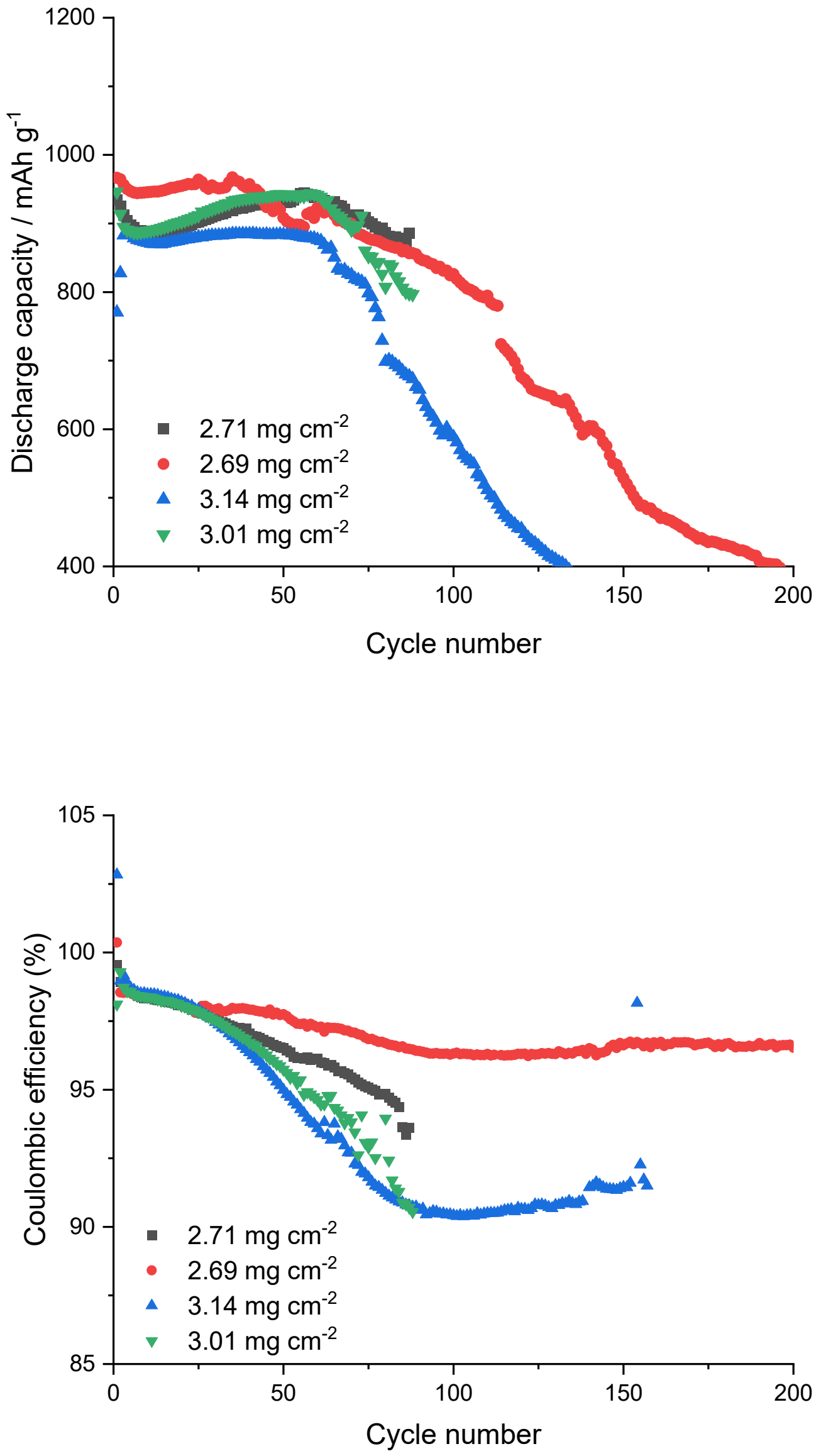

Figure S54. As in Figure S25 but in DME:FDMB 3:2.

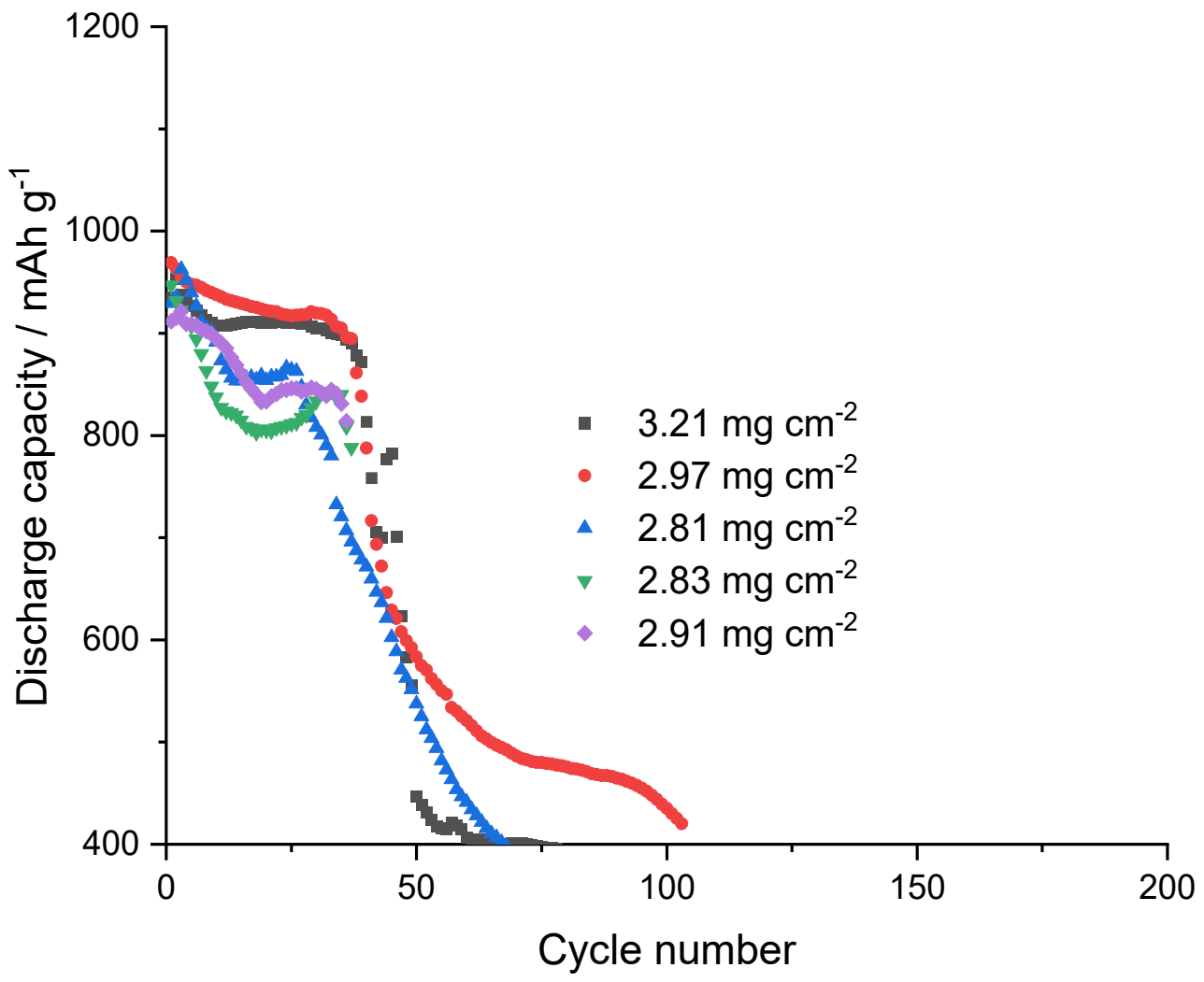

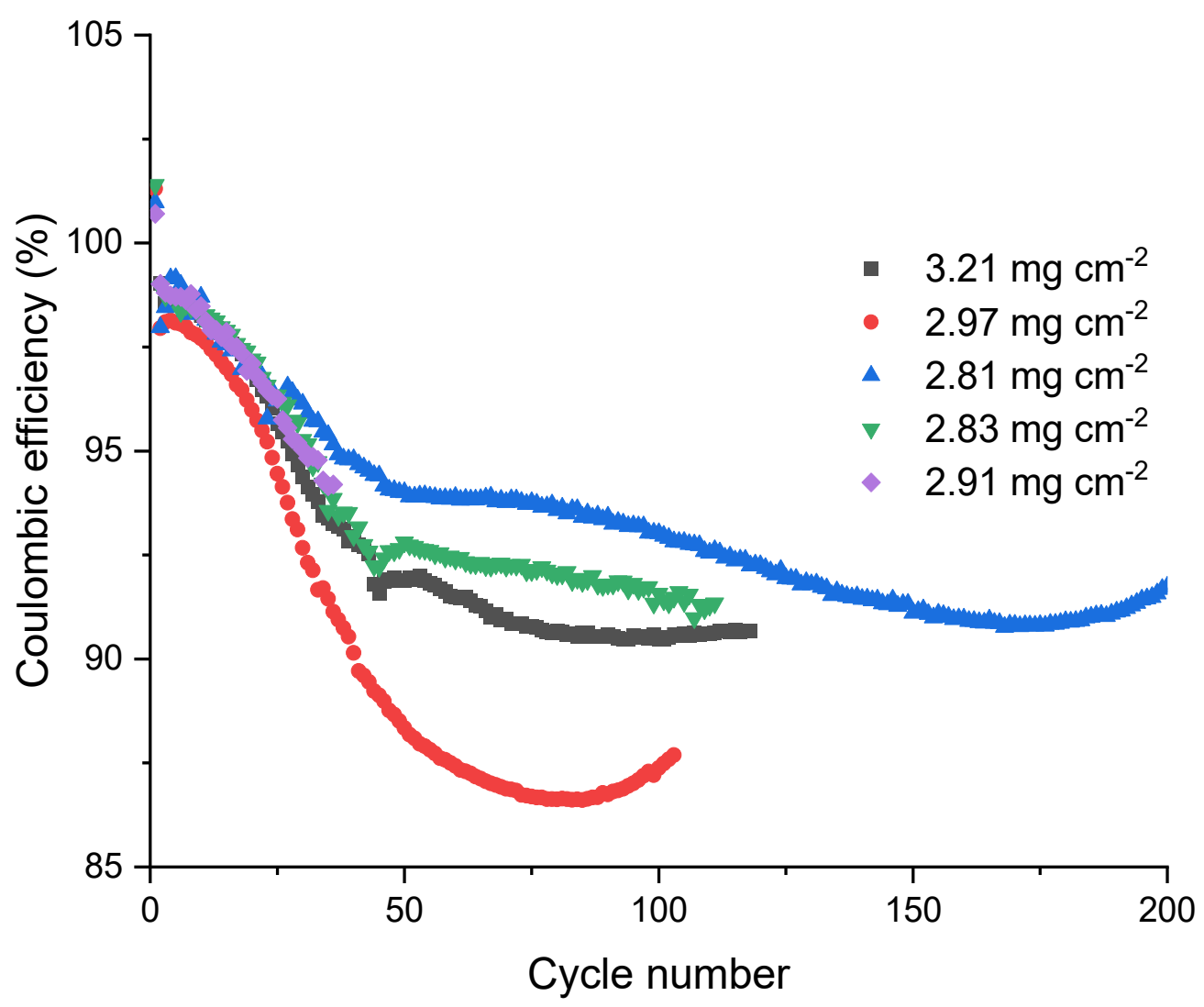

Figure S55. As in Figure S25 but in DME:BTFE 3:2.

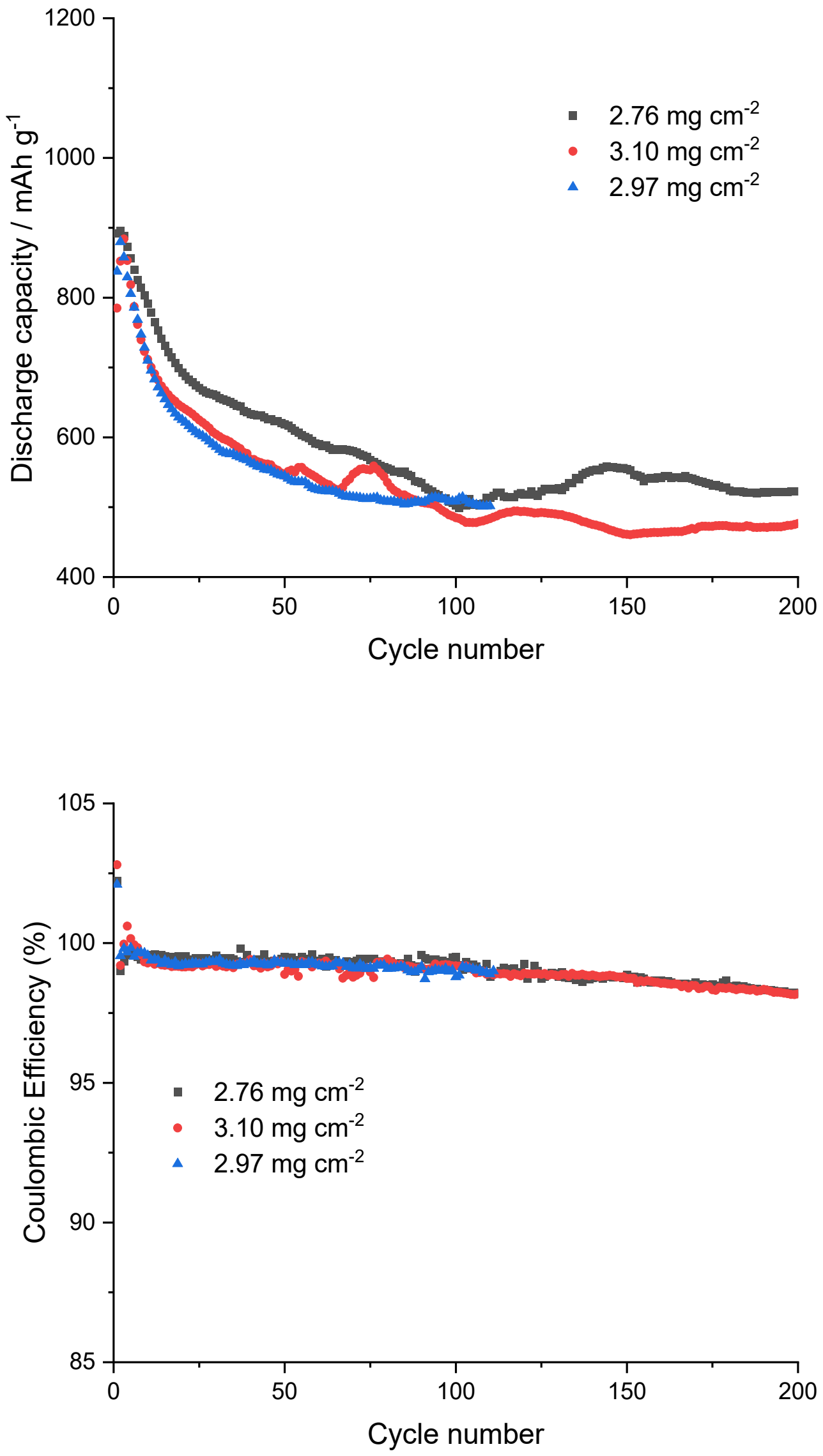

Figure S56. As in Figure S25 but in DME:TTE 3:2.

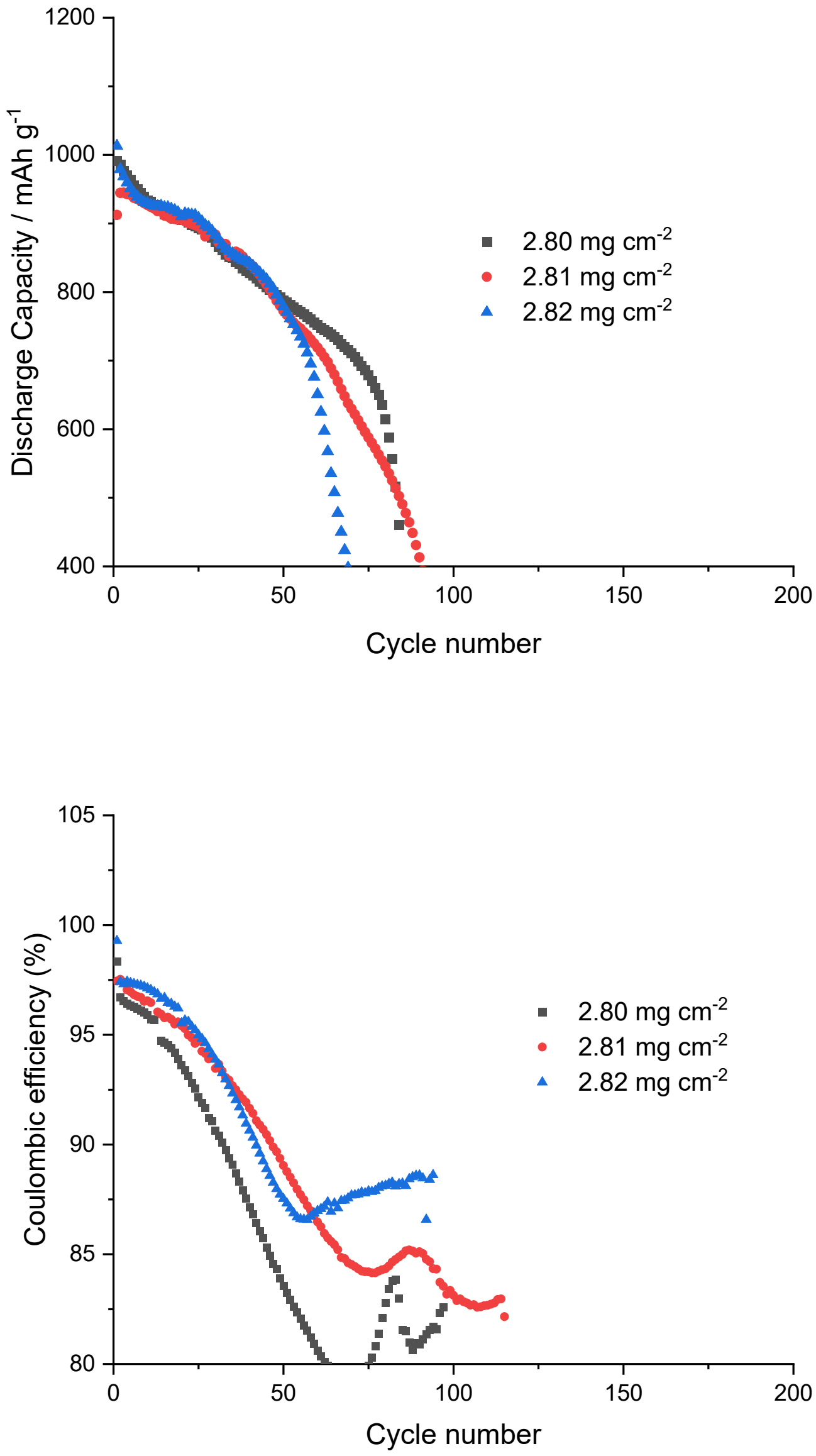

Figure S57. As in Figure S25 but in DME:ETFE 3:2.

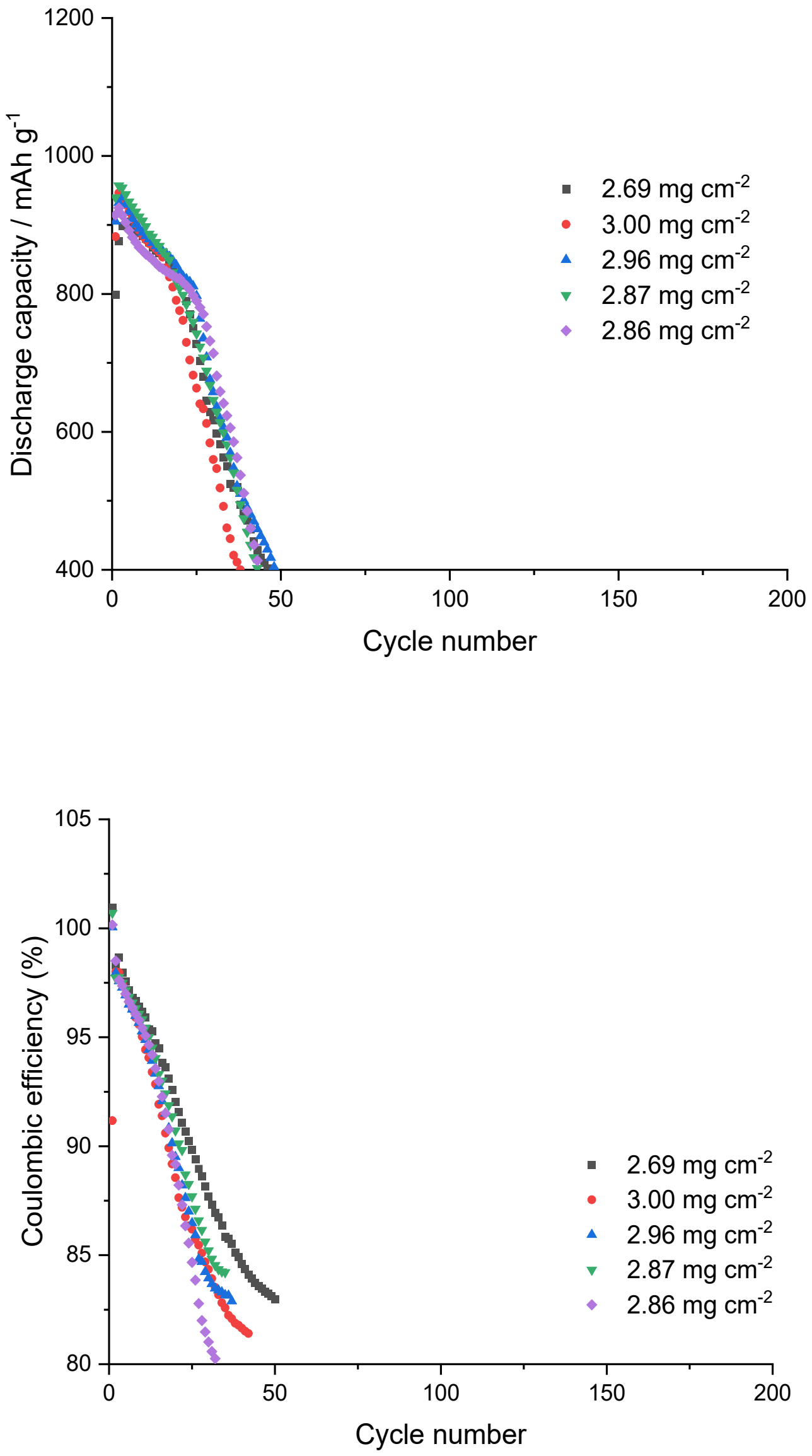

Figure S58. As in Figure S25 but in DME:TFPP 3:2.

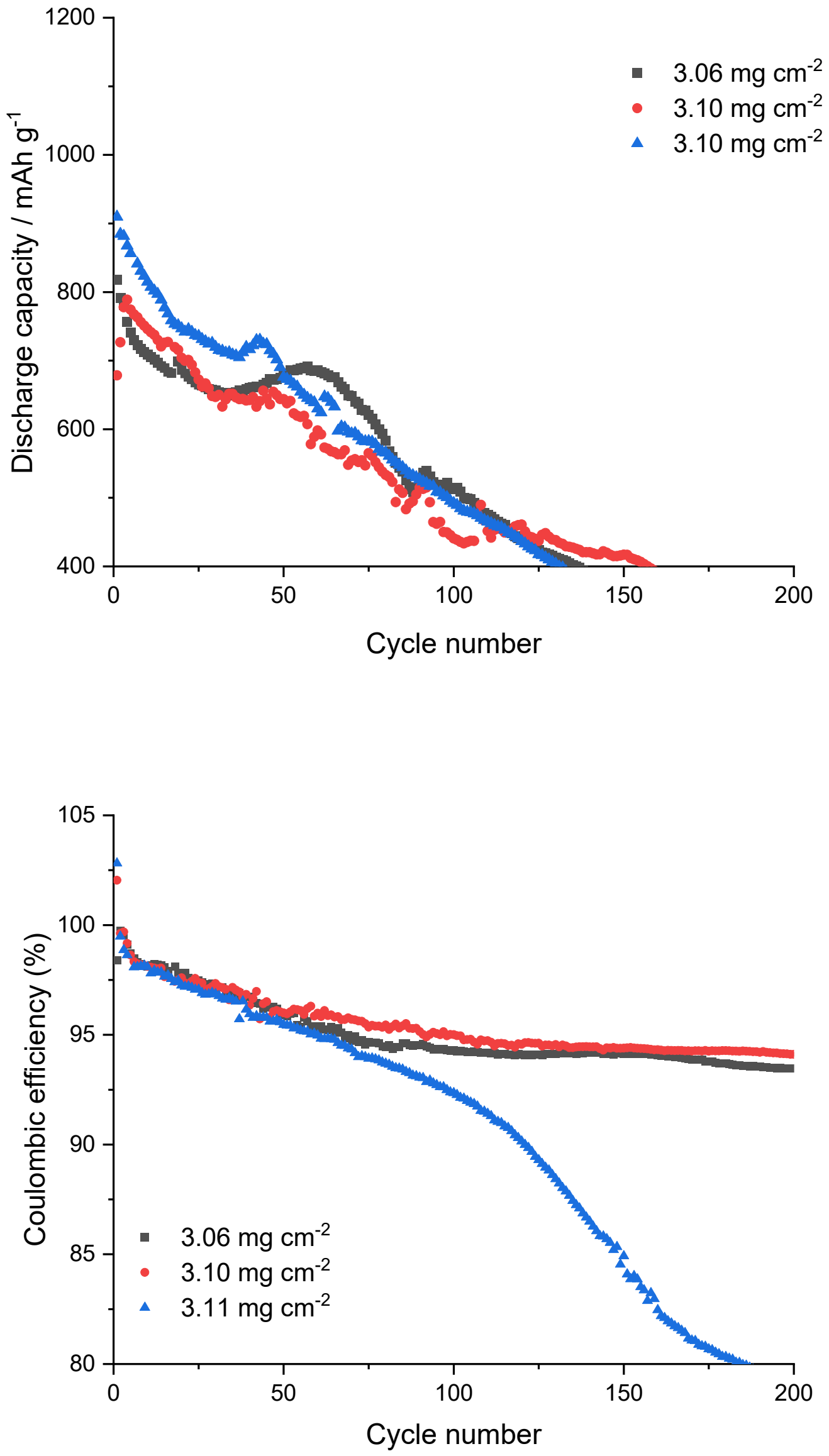

Figure S59. As in Figure S25 but in DME:TFEE 3:2.

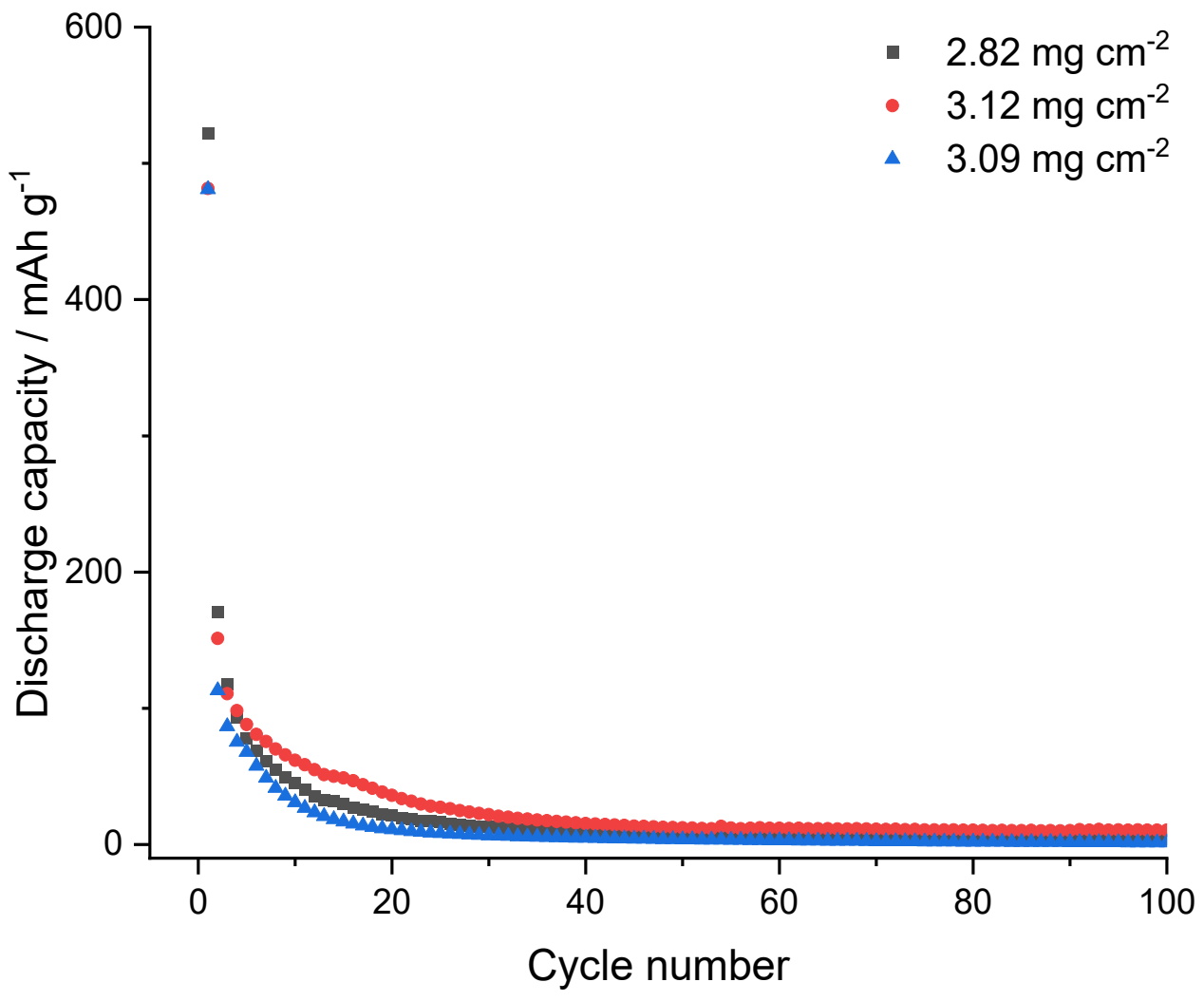

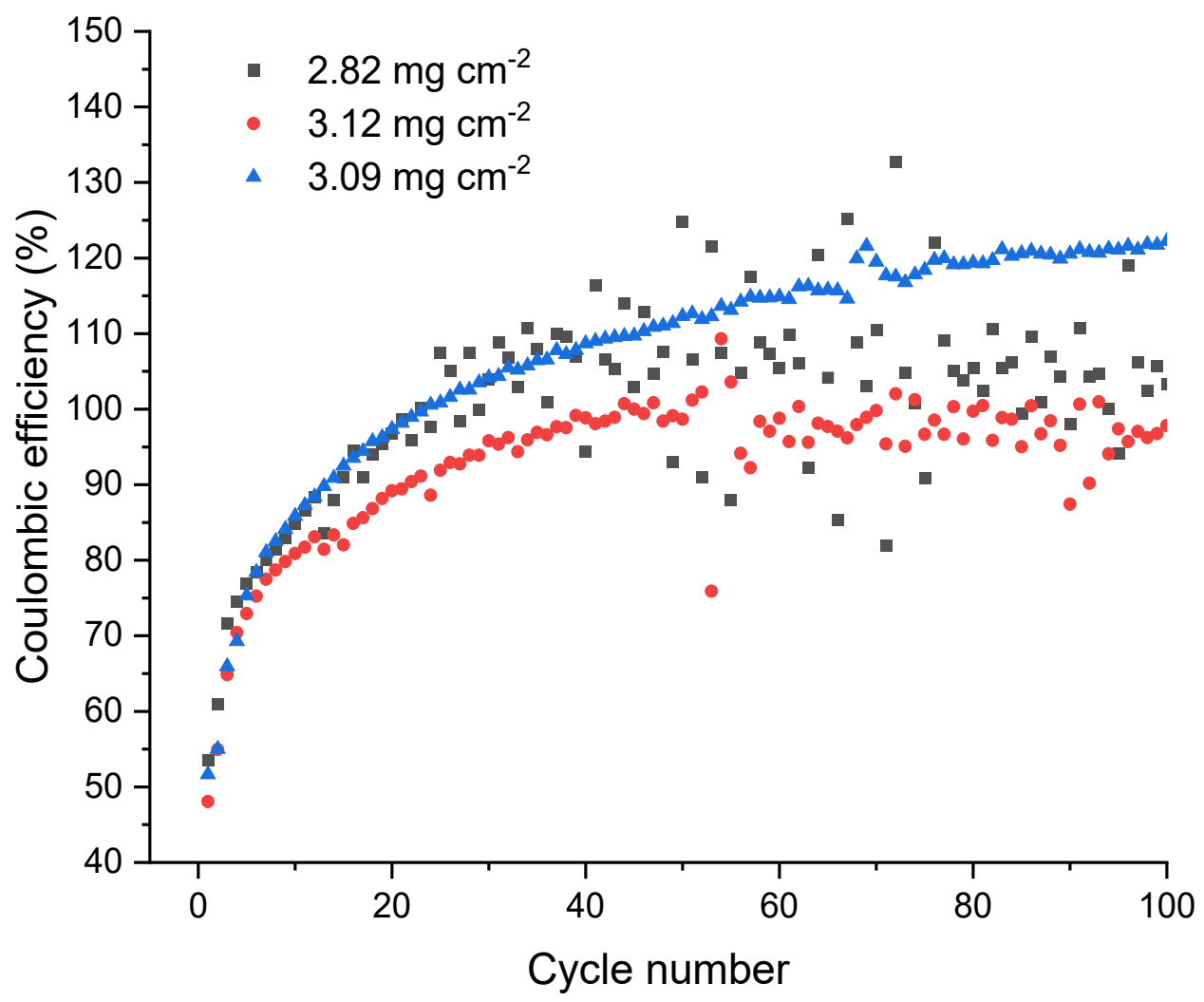

Figure S60. As in Figure S25 but in DME:TFFMP 3:2. Note the use of a different y-scale, compared to other Figures.

References:

(1) Lacey, M. J.; Österlund, V.; Bergfelt, A.; Jeschull, F.; Bowden, T.; Brandell, D. A Robust, Water-Based, Functional Binder Framework for High-Energy Lithium–Sulfur Batteries. *ChemSusChem* **2017**, *10* (13), 2758–2766. https://doi.org/10.1002/cssc.201700743.

(2) Dörfler, S.; Althues, H.; Härtel, P.; Abendroth, T.; Schumm, B.; Kaskel, S. Challenges and Key Parameters of Lithium-Sulfur Batteries on Pouch Cell Level. *Joule* **2020**, *4* (3), 539–554. https://doi.org/10.1016/j.joule.2020.02.006.

(3) Aguiar, P. M.; Jacquinot, J. F.; Sakellariou, D. Experimental and Numerical Examination of Eddy (Foucault) Currents in Rotating Micro-Coils: Generation of Heat and Its Impact on Sample Temperature. *J. Magn. Reson.* **2009**, *200* (1), 6–14. https://doi.org/10.1016/j.jmr.2009.05.010.

(4) Pecher, O.; Carretero-Gonzalez, J.; Griffith, K. J.; Grey, C. P. Materials' Methods: NMR in Battery Research. *Chem. Mater.* **2017**, *29* (1), 213–242. https://doi.org/10.1021/acs.chemmater.6b03183.

(5) Fritzke, J. B.; Ellison, J. H. J.; Brazel, L.; Horwitz, G.; Menkin, S.; Grey, C. P. Spiers Memorial Lecture: Lithium Air Batteries – Tracking Function and Failure. *Faraday Discuss.* **2024**, *248* (0), 9–28. https://doi.org/10.1039/d3fd00154g.

(6) Menkin, S.; Fritzke, J. B.; Larner, R.; de Leeuw, C.; Choi, Y.; Gunnarsdóttir, A. B.; Grey, C. P. Insights into Soft Short Circuit-Based Degradation of Lithium Metal Batteries. *Faraday Discuss.* **2024**, *248* (0), 277–297. https://doi.org/10.1039/d3fd00101f.

(7) https://github.com/neubifx/Battflow.

(8) Abraham, M. J.; Murtola, T.; Schulz, R.; Páll, S.; Smith, J. C.; Hess, B.; Lindah, E. GROMACS: High Performance Molecular Simulations through Multi-Level Parallelism from Laptops to Supercomputers. *SoftwareX* **2015**, *1–2*, 19–25. https://doi.org/10.1016/j.softx.2015.06.001.

(9) Martinez, L.; Andrade, R.; Birgin, E. G.; Martínez, J. M. PACKMOL: A Package for Building Initial Configurations for Molecular Dynamics Simulations. *J. Comput. Chem.* **2009**, *30* (13), 2157–2164. https://doi.org/10.1002/jcc.21224.

(10) Jorgensen, W. L.; Maxwell, D. S.; Tirado-Rives, J. Development and Testing of the OPLS All-Atom Force Field on Conformational Energetics and Properties of Organic Liquids. *J. Am. Chem. Soc.* **1996**, *118* (45), 11225–11236. https://doi.org/10.1021/ja9621760.

(11) Jorgensen, W. L.; Tirado-Rives, J. Potential Energy Functions for Atomic-Level Simulations of Water and Organic and Biomolecular Systems. *Proc. Natl. Acad. Sci.* **2005**, *102* (19), 6665–6670. https://doi.org/10.1073/pnas.0408037102.

(12) Dodda, L. S.; Vilseck, J. Z.; Tirado-Rives, J.; Jorgensen, W. L. 1.14∗CM1A-LBCC: Localized Bond-Charge Corrected CM1A Charges for Condensed-Phase Simulations. *J. Phys. Chem. B* **2017**, *121* (15), 3864–3870. https://doi.org/10.1021/acs.jpcb.7b00272.

(13) Dodda, L. S.; De Vaca, I. C.; Tirado-Rives, J.; Jorgensen, W. L. LigParGen Web Server: An Automatic OPLS-AA Parameter Generator for Organic Ligands. *Nucleic Acids Res.* **2017**, *45* (W1), W331–W336. https://doi.org/10.1093/nar/gkx312.

(14) Doherty, B.; Zhong, X.; Gathiaka, S.; Li, B.; Acevedo, O. Revisiting OPLS Force Field Parameters for Ionic Liquid Simulations. *J. Chem. Theory Comput.* **2017**, *13* (12), 6131–6135. https://doi.org/10.1021/acs.jctc.7b00520.

(15) Michaud-Agrawal, N.; Denning, E. J.; Woolf, T. B.; Beckstein, O. MDAnalysis: A Toolkit for the Analysis of Molecular Dynamics Simulations. *J. Comput. Chem.* **2011**, *32* (10), 2319–2327. https://doi.org/10.1002/jcc.21787.

(16) Cohen, O. A.; Macdermott-Opeskin, H.; Lee, L.; Hou, T.; Fong, K. D.; Kingsbury, R.; Wang, J.; Persson, K. A. SolvationAnalysis: A Python Toolkit for Understanding Liquid Solvation Structure in Classical Molecular Dynamics Simulations. *J. Open Source Softw.* **2023**, *8* (84), 5183. https://doi.org/10.21105/joss.05183.

(17) Allen, M. P.; Tildesley, D. J. *Computer Simulation of Liquids*; Oxford University Press, 2017. https://doi.org/10.1093/oso/9780198803195.001.0001.

(18) Lourenço, T. C.; Barros, L. M. S.; Anchieta, C. G.; Nepel, T. C. M.; Júlio, J. P. O.; Dias, L. G.; Filho, R. M.; Doubek, G.; Da Silva, J. L. F. Tuning Aprotic Solvent Properties with Long Alkyl Chain Ionic Liquid for Lithium-Based Electrolytes. *J. Mater. Chem. A Mater.* **2022**, *10* (21), 11684–11701. https://doi.org/10.1039/d1ta10592b.

(19) Lourenço, T. C.; Dias, L. G.; Da Silva, J. L. F. Theoretical Investigation of the Na^+ Transport Mechanism and the Performance of Ionic Liquid-Based Electrolytes in Sodium-Ion Batteries. *ACS Appl. Energy Mater.* **2021**, *4* (5), 4444–4458. https://doi.org/10.1021/acsaem.1c00059.

(20) Long, Z. C.; Wei, Z. Y.; Liu, K. W.; Li, X. L.; Xu, X. L.; Xu, H. G.; Zheng, W. J. Structures and Bonding Properties of Lithium Polysulfide Clusters $LiS_n^{-/0}$ (n = 3–5) and $Li_2S_4^{-/0}$: Size-Selected Anion Photoelectron Spectroscopy and Theoretical Calculations. *Phys. Chem. Chem. Phys.* **2023**, *25* (15), 10495–10503. https://doi.org/10.1039/d2cp06061b.

(21) Neese, F. Software Update: The ORCA Program System—Version 5.0. *Wiley Interdiscip. Rev. Comput. Mol. Sci.* **2022**, *12* (5), e1606. https://doi.org/10.1002/wcms.1606.

(22) Azimi, N.; Weng, W.; Takoudis, C.; Zhang, Z. Improved Performance of Lithium–Sulfur Battery with Fluorinated Electrolyte. *Electrochem. commun.* **2013**, *37*, 96–99. https://doi.org/10.1016/j.elecom.2013.10.020.

(23) Azimi, N.; Xue, Z.; Rago, N. D.; Takoudis, C.; Gordin, M. L.; Song, J.; Wang, D.; Zhang, Z. Fluorinated Electrolytes for Li-S Battery: Suppressing the Self-Discharge with an Electrolyte Containing Fluoroether Solvent. *J. Electrochem. Soc.* **2015**, *162* (1), A64–A68. https://doi.org/10.1149/2.0431501jes.

(24) Zu, C.; Azimi, N.; Zhang, Z.; Manthiram, A. Insight into Lithium–Metal Anodes in Lithium–Sulfur Batteries with a Fluorinated Ether Electrolyte. *J. Mater. Chem. A Mater.* **2015**, *3* (28), 14864–14870. https://doi.org/10.1039/c5ta03195h.

(25) Azimi, N.; Xue, Z.; Bloom, I.; Gordin, M. L.; Wang, D.; Daniel, T.; Takoudis, C.; Zhang, Z. Understanding the Effect of a Fluorinated Ether on the Performance of Lithium-Sulfur Batteries. *ACS Appl. Mater. Interfaces* **2015**, *7* (17), 9169–9177. https://doi.org/10.1021/acsami.5b01412.

(26) Jeddi, K.; Sarikhani, K.; Ghaznavi, M.; Zendehboodi, S.; Chen, P. Enhanced Cycling Performance of a High-Energy and Low-Cost Lithium–Sulfur Battery with a Sulfur/Hardwood Charcoal

Composite Cathode Material. *J. Solid State Electrochem.* **2015**, *19* (4), 1161–1169. https://doi.org/10.1007/s10008-014-2721-7.

(27) Zheng, J.; Fan, X.; Ji, G.; Wang, H.; Hou, S.; DeMella, K. C.; Raghavan, S. R.; Wang, J.; Xu, K.; Wang, C. Manipulating Electrolyte and Solid Electrolyte Interphase to Enable Safe and Efficient Li-S Batteries. *Nano Energy* **2018**, *50*, 431–440. https://doi.org/10.1016/j.nanoen.2018.05.065.

(28) Chen, Y.; Gong, Z.; Yang, Y. Enhanced Electrochemical Performance of High-Energy Lithium-Sulfur Batteries Using an Electrolyte with 1,1,2,2-Tetrafluoro-3-(1,1,2,2-Tetrafluoroethoxy)Propane. *J. Electrochem. Soc.* **2018**, *165* (9), A1915–A1919. https://doi.org/10.1149/2.1531809jes.

(29) Meisner, Q. J.; Rojas, T.; Dietz Rago, N. L.; Cao, J.; Bareño, J.; Glossmann, T.; Hintennach, A.; Redfern, P. C.; Pahls, D.; Zhang, L.; Bloom, I. D.; Ngo, A. T.; Curtiss, L. A.; Zhang, Z. Lithium–Sulfur Battery with Partially Fluorinated Ether Electrolytes: Interplay between Capacity, Coulombic Efficiency and Li Anode Protection. *J. Power Sources* **2019**, *438*, 226939. https://doi.org/10.1016/j.jpowsour.2019.226939.

(30) Su, C.-C.; He, M.; Amine, R.; Amine, K. A Selection Rule for Hydrofluoroether Electrolyte Cosolvent: Establishing a Linear Free-Energy Relationship in Lithium–Sulfur Batteries. *Angew. Chem. Int. Ed.* **2019**, *58* (31), 10591–10595. https://doi.org/10.1002/anie.201904240.

(31) Zhao, H.; Gu, J.; Gao, Y.; Hou, Q.; Ren, Z.; Qi, Y.; Zhang, K.; Shen, C.; Zhang, J.; Xie, K. A Multifunctional Electrolyte with Highly-Coordinated Solvation Structure-in-Nonsolvent for Rechargeable Lithium Batteries. *J. Energy Chem.* **2020**, *51*, 362–371. https://doi.org/10.1016/j.jechem.2020.04.044.

(32) Zhao, C.; Xu, G. L.; Zhao, T.; Amine, K. Beyond the Polysulfide Shuttle and Lithium Dendrite Formation: Addressing the Sluggish Sulfur Redox Kinetics for Practical High-Energy Li-S Batteries. *Angew. Chem. Int. Ed.* **2020**, *59* (40), 17634–17640. https://doi.org/10.1002/anie.202007159.

(33) Hou, L. P.; Li, Z.; Yao, N.; Bi, C. X.; Li, B. Q.; Chen, X.; Zhang, X. Q.; Zhang, Q. Weakening the Solvating Power of Solvents to Encapsulate Lithium Polysulfides Enables Long-Cycling Lithium–Sulfur Batteries. *Adv. Mater.* **2022**, *34* (45), 2205284. https://doi.org/10.1002/adma.202205284.

(34) Li, Z.; Hou, L. P.; Yao, N.; Li, X. Y.; Chen, Z. X.; Chen, X.; Zhang, X. Q.; Li, B. Q.; Zhang, Q. Correlating Polysulfide Solvation Structure with Electrode Kinetics towards Long-Cycling Lithium–Sulfur Batteries. *Angew. Chem. Int. Ed.* **2023**, *62* (43), e202309968. https://doi.org/10.1002/anie.202309968.

(35) Li, J.; Cong, J.; Ji, H.; Shi, T.; Yuan, L.; Huang, Y. Tuning the Solubility of Polysulfides for Constructing Practical Lithium-Sulfur Battery. *J. Energy Chem.* **2024**, *95*, 611–617. https://doi.org/10.1016/j.jechem.2024.04.009.

(36) Gordin, M. L.; Dai, F.; Chen, S.; Xu, T.; Song, J.; Tang, D.; Azimi, N.; Zhang, Z.; Wang, D. Bis(2,2,2-Trifluoroethyl) Ether as an Electrolyte Co-Solvent for Mitigating Self-Discharge in Lithium - Sulfur Batteries. *ACS Appl. Mater. Interfaces* **2014**, *6* (11), 8006–8010. https://doi.org/10.1021/am501665s.

(37) Lu, H.; Yuan, Y.; Zhang, K.; Qin, F.; Lai, Y.; Liu, Y. Application of Partially Fluorinated Ether for Improving Performance of Lithium/Sulfur Batteries. *J. Electrochem. Soc.* **2015**, *162* (8), A1460–A1465. https://doi.org/10.1149/2.0281508jes.

(38) Kim, I.; Kim, S.; Cho, H.; Jung, J.; Kwon, H.; Kim, D.; Shin, Y.; Kim, H.-T. Moderately Solvating Electrolyte with Fluorinated Cosolvents for Lean-Electrolyte Li–S Batteries. *Adv. Energy Mater.* **2025**, *15* (14), 2403828. https://doi.org/10.1002/aenm.202403828.

(39) Lu, H.; Zhang, K.; Yuan, Y.; Qin, F.; Zhang, Z.; Lai, Y.; Liu, Y. Lithium/Sulfur Batteries with Mixed Liquid Electrolytes Based on Ethyl 1,1,2,2-Tetrafluoroethyl Ether. *Electrochim. Acta* **2015**, *161*, 55–62. https://doi.org/10.1016/j.electacta.2015.02.031.

(40) Gu, S.; Qian, R.; Jin, J.; Wang, Q.; Guo, J.; Zhang, S.; Zhuo, S.; Wen, Z. Suppressing the Dissolution of Polysulfides with Cosolvent Fluorinated Diether towards High-Performance Lithium Sulfur Batteries. *Phys. Chem. Chem. Phys.* **2016**, *18* (42), 29293–29299. https://doi.org/10.1039/c6cp04775k.

(41) Gao, M.; Su, C. C.; He, M.; Glossmann, T.; Hintennach, A.; Feng, Z.; Huang, Y.; Zhang, Z. A High Performance Lithium–Sulfur Battery Enabled by a Fish-Scale Porous Carbon/Sulfur Composite and Symmetric Fluorinated Diethoxyethane Electrolyte. *J. Mater. Chem. A Mater.* **2017**, *5* (14), 6725–6733. https://doi.org/10.1039/c7ta01057e.

(42) Drvarič Talian, S.; Jeschke, S.; Vizintin, A.; Pirnat, K.; Arčon, I.; Aquilanti, G.; Johansson, P.; Dominko, R. Fluorinated Ether Based Electrolyte for High-Energy Lithium-Sulfur Batteries: Li+ Solvation Role behind Reduced Polysulfide Solubility. *Chem. Mater.* **2017**, *29* (23), 10037–10044. https://doi.org/10.1021/acs.chemmater.7b03654.

(43) Cao, J.; Tornheim, A.; Glossmann, T.; Hintennach, A.; Rojas, T.; Meisner, Q.; Sahore, R.; Liu, Q.; Wang, Y.; Ngo, A.; Curtiss, L. A.; Zhang, Z. Understanding the Impact of a Nonafluorinated Ether-Based Electrolyte on Li-S Battery. *J. Electrochem. Soc.* **2019**, *166* (15), A3653–A3659. https://doi.org/10.1149/2.0751915jes.

(44) Zhao, Y.; Fang, C.; Zhang, G.; Hubble, D.; Nallapaneni, A.; Zhu, C.; Zhao, Z.; Liu, Z.; Lau, J.; Fu, Y.; Liu, G. A Micelle Electrolyte Enabled by Fluorinated Ether Additives for Polysulfide Suppression and Li Metal Stabilization in Li-S Battery. *Front. Chem.* **2020**, *8*, 542987. https://doi.org/10.3389/fchem.2020.00484.

(45) Yue, Z.; Dunya, H.; Aryal, S.; Segre, C. U.; Mandal, B. Synthesis and Electrochemical Properties of Partially Fluorinated Ether Solvents for Lithium-Sulfur Battery Electrolytes. *J. Power Sources* **2018**, *401*, 271–277. https://doi.org/10.1016/j.jpowsour.2018.08.097.

(46) Zheng, J.; Ji, G.; Fan, X.; Chen, J.; Li, Q.; Wang, H.; Yang, Y.; Demella, K. C.; Raghavan, S. R.; Wang, C. High-Fluorinated Electrolytes for Li–S Batteries. *Adv. Energy Mater.* **2019**, *9* (16), 1803774. https://doi.org/10.1002/aenm.201803774.

(47) Robles-Fernández, A.; Moreno-Fernández, G.; Soria-Fernández, A.; Castillo, J.; Santiago, A.; Carriazo, D. Towards Practical Li–S Batteries through the Combination of a Nanostructured Graphene Composite Cathode and a Novel Sparingly Solvating Electrolyte. *Carbon.* **2024**, *229*, 119442. https://doi.org/10.1016/j.carbon.2024.119442.

(48) Gao, X.; Yu, Z.; Wang, J.; Zheng, X.; Ye, Y.; Gong, H.; Xiao, X.; Yang, Y.; Chen, Y.; Bone, S. E.; Greenburg, L. C.; Zhang, P.; Su, H.; Affeld, J.; Bao, Z.; Cui, Y. Electrolytes with Moderate Lithium Polysulfide Solubility for High-Performance Long-Calendar-Life Lithium–Sulfur Batteries. *Proc.*

Natl. Acad. Sci. U. S. A. **2023**, *120* (31), e2301260120. https://doi.org/10.1073/pnas.2301260120.

(49) Dokko, K.; Tachikawa, N.; Yamauchi, K.; Tsuchiya, M.; Yamazaki, A.; Takashima, E.; Park, J.-W.; Ueno, K.; Seki, S.; Serizawa, N.; Watanabe, M. Solvate Ionic Liquid Electrolyte for Li–S Batteries. *J. Electrochem. Soc.* **2013**, *160* (8), A1304–A1310. https://doi.org/10.1149/2.111308jes.

(50) Cuisinier, M.; Cabelguen, P. E.; Adams, B. D.; Garsuch, A.; Balasubramanian, M.; Nazar, L. F. Unique Behaviour of Nonsolvents for Polysulphides in Lithium–Sulphur Batteries. *Energy Environ. Sci.* **2014**, *7* (8), 2697–2705. https://doi.org/10.1039/c4ee00372a.

(51) Weller, C.; Thieme, S.; Härtel, P.; Althues, H.; Kaskel, S. Intrinsic Shuttle Suppression in Lithium-Sulfur Batteries for Pouch Cell Application. *J. Electrochem. Soc.* **2017**, *164* (14), A3766–A3771. https://doi.org/10.1149/2.0981714jes.

(52) Shin, M.; Wu, H. L.; Narayanan, B.; See, K. A.; Assary, R. S.; Zhu, L.; Haasch, R. T.; Zhang, S.; Zhang, Z.; Curtiss, L. A.; Gewirth, A. A. Effect of the Hydrofluoroether Cosolvent Structure in Acetonitrile-Based Solvate Electrolytes on the Li^+ Solvation Structure and Li-S Battery Performance. *ACS Appl. Mater. Interfaces* **2017**, *9* (45), 39357–39370. https://doi.org/10.1021/acsami.7b11566.

(53) Lu, H.; Chen, Z.; Yuan, Y.; Du, H.; Wang, J.; Liu, X.; Hou, Z.; Zhang, K.; Fang, J.; Qu, Y. A Rational Balance Design of Hybrid Electrolyte Based on Ionic Liquid and Fluorinated Ether in Lithium Sulfur Batteries. *J. Electrochem. Soc.* **2019**, *166* (12), A2453–A2458. https://doi.org/10.1149/2.0321912jes.

(54) Lu, H.; Zhu, Y.; Zheng, B.; Du, H.; Zheng, X.; Liu, C.; Yuan, Y.; Fang, J.; Zhang, K. A Hybrid Ionic Liquid-Based Electrolyte for High-Performance Lithium–Sulfur Batteries. *New J. Chem.* **2019**, *44* (2), 361–368. https://doi.org/10.1039/c9nj03790j.

(55) Pang, Q.; Shyamsunder, A.; Narayanan, B.; Kwok, C. Y.; Curtiss, L. A.; Nazar, L. F. Tuning the Electrolyte Network Structure to Invoke Quasi-Solid State Sulfur Conversion and Suppress Lithium Dendrite Formation in Li–S Batteries. *Nat. Energy 2018 3:9* **2018**, *3* (9), 783–791. https://doi.org/10.1038/s41560-018-0214-0.

(56) He, M.; Li, X.; Holmes, N. G.; Li, R.; Wang, J.; Yin, G.; Zuo, P.; Sun, X. Flame-Retardant and Polysulfide-Suppressed Ether-Based Electrolytes for High-Temperature Li-S Batteries. *ACS Appl. Mater. Interfaces* **2021**, *13* (32), 38296–38304. https://doi.org/10.1021/acsami.1c09492.

(57) Qi, X.; Yang, F.; Sang, P.; Zhu, Z.; Jin, X.; Pan, Y.; Ji, J.; Jiang, R.; Du, H.; Ji, Y.; Fu, Y.; Qie, L.; Huang, Y. Electrochemical Reactivation of Dead Li_2S for Li−S Batteries in Non-Solvating Electrolytes. *Angew. Chem. Int. Ed.* **2023**, *62* (9), e202218803. https://doi.org/10.1002/anie.202218803.

(58) Wang, L.; Xie, Y.; Qi, X.; Jiang, R.; Huang, K.; Qie, L.; Li, S. Ultralean Electrolyte Li-S Battery by Avoiding Gelation Catastrophe. *ACS Appl. Mater. Interfaces* **2022**, *14* (41), 46457–46470. https://doi.org/10.1021/acsami.2c10906.

(59) Castillo, J.; Soria-Fernández, A.; Rodriguez-Peña, S.; Rikarte, J.; Robles-Fernández, A.; Aldalur, I.; Cid, R.; Antonio González-Marcos, J.; Carrasco, J.; Armand, M.; Santiago, A.; Carriazo, D. Graphene-Based Sulfur Cathodes and Dual Salt-Based Sparingly Solvating Electrolytes: A

Perfect Marriage for High Performing, Safe, and Long Cycle Life Lithium-Sulfur Prototype Batteries. *Adv. Energy Mater.* **2024**, *14* (1), 2302378. https://doi.org/10.1002/aenm.202302378.

(60) Shi, Z.; Thomas, S.; Guo, D.; Tian, Z.; Zhao, Z.; Wang, Y.; Emwas, A. H.; Wehbe, N.; Melinte, G.; Bakr, O. M.; Mohammed, O. F.; Alshareef, H. N. Solvation Sheath Reorganization by Alkyl Chain Tuning Promises Lean-Electrolyte Li-S Batteries. *ACS Energy Lett.* **2024**, *21*, 5391–5402. https://doi.org/10.1021/acsenergylett.4c02049.

(61) Bruce, P. G.; Vincent, C. A. Effect of Ion Association on Transport in Polymer Electrolytes. *Faraday Discuss. Chem. Soc.* **1989**, *88* (0), 43–54. https://doi.org/10.1039/DC9898800043.

(62) Evans, J.; Vincent, C. A.; Bruce, P. G. Electrochemical Measurement of Transference Numbers in Polymer Electrolytes. *Polymer (Guildf).* **1987**, *28* (13), 2324–2328. https://doi.org/10.1016/0032-3861(87)90394-6.

(63) Bruce, P. G.; Vincent, C. A. Steady State Current Flow in Solid Binary Electrolyte Cells. *J. Electroanal. Chem. Interfacial Electrochem.* **1987**, *225* (1–2), 1–17. https://doi.org/10.1016/0022-0728(87)80001-3.

(64) Wohde, F.; Balabajew, M.; Roling, B. Li^+ Transference Numbers in Liquid Electrolytes Obtained by Very-Low-Frequency Impedance Spectroscopy at Variable Electrode Distances. *J. Electrochem. Soc.* **2016**, *163* (5), A714. https://doi.org/10.1149/2.0811605JES.

(65) Ma, Y.; Doyle, M.; Fuller, T. F.; Doeff, M. M.; De Jonghe, L. C.; Newman, J. The Measurement of a Complete Set of Transport Properties for a Concentrated Solid Polymer Electrolyte Solution. *J. Electrochem. Soc.* **1995**, *142* (6), 1859–1868. https://doi.org/10.1149/1.2044206.

(66) Bergstrom, H. K.; Fong, K. D.; D. McCloskey, B. Interfacial Effects on Transport Coefficient Measurements in Li-Ion Battery Electrolytes. *J. Electrochem. Soc.* **2021**, *168* (6), 060543. https://doi.org/10.1149/1945-7111/ac0994.

(67) Liu, J.; Monroe, C. W. On the Characterization of Battery Electrolytes with Polarization Cells. *Electrochim. Acta* **2015**, *167*, 357–363. https://doi.org/10.1016/j.electacta.2015.03.104.

(68) Ishfaq, H. A.; Cruz Cardona, C.; Tchernychova, E.; Johansson, P.; Gaberšček, M.; Dominko, R.; Drvarič Talian, S. Transport Number Determination and Relevance for Lithium Metal Batteries Using Localized Highly Concentrated Electrolytes. *Chem. Mater.* **2025**, *37* (7), 2485–2495. https://doi.org/10.1021/acs.chemmater.4C03067.

(69) Doyle, M.; Fuller, T. F.; Newman, J. The Importance of the Lithium Ion Transference Number in Lithium/Polymer Cells. *Electrochim. Acta* **1994**, *39* (13), 2073–2081. https://doi.org/10.1016/0013-4686(94)85091-7.

(70) Diederichsen, K. M.; McShane, E. J.; McCloskey, B. D. Promising Routes to a High Li+ Transference Number Electrolyte for Lithium Ion Batteries. *ACS Energy Lett.* **2017**, *2* (11), 2563–2575. https://doi.org/10.1021/acsenergylett.7b00792.

(71) Choi, I. R.; Chen, Y.; Shah, A.; Florian, J.; Serrao, C.; Holoubek, J.; Lyu, H.; Zhang, E.; Lee, J. H.; Lin, Y.; Kim, S. C.; Park, H.; Zhang, P.; Lee, J.; Qin, J.; Cui, Y.; Bao, Z. Asymmetric Ether Solvents for High-Rate Lithium Metal Batteries. *Nat. Energy 2025 10:3* **2025**, *10* (3), 365–379. https://doi.org/10.1038/s41560-025-01716-w.

(72) Takeshita, K.; Tatara, R.; Tsuzuki, S.; Ishikawa, M.; Dokko, K. How Does Hydrofluoroether Affect the Liquid Structure, Transport Properties, and Electrochemistry of Localized High-Concentration Electrolytes? *J. Chem. Phys.* **2025**, *163* (17). https://doi.org/10.1063/5.0293467/3370670.

(73) Amanchukwu, C. V.; Yu, Z.; Kong, X.; Qin, J.; Cui, Y.; Bao, Z. A New Class of Ionically Conducting Fluorinated Ether Electrolytes with High Electrochemical Stability. *J. Am. Chem. Soc.* **2020**, *142* (16), 7393–7403. https://doi.org/10.1021/JACS.9B11056.

(74) Watanabe, Y.; Ugata, Y.; Ueno, K.; Watanabe, M.; Dokko, K. Does Li-Ion Transport Occur Rapidly in Localized High-Concentration Electrolytes? *Phys. Chem. Chem. Phys.* **2023**, *25* (4), 3092–3099. https://doi.org/10.1039/D2CP05319E.

(75) Holoubek, J.; Liu, H.; Wu, Z.; Yin, Y.; Xing, X.; Cai, G.; Yu, S.; Zhou, H.; Pascal, T. A.; Chen, Z.; Liu, P. Tailoring Electrolyte Solvation for Li Metal Batteries Cycled at Ultra-Low Temperature. *Nat. Energy 2021 6:3* **2021**, *6* (3), 303–313. https://doi.org/10.1038/s41560-021-00783-z.

(76) Chazalviel, J. N. Electrochemical Aspects of the Generation of Ramified Metallic Electrodeposits. *Phys. Rev. A (Coll. Park).* **1990**, *42* (12), 7355. https://doi.org/10.1103/PhysRevA.42.7355.

(77) Bai, P.; Li, J.; Brushett, F. R.; Bazant, M. Z. Transition of Lithium Growth Mechanisms in Liquid Electrolytes. *Energy Environ. Sci.* **2016**, *9* (10), 3221–3229. https://doi.org/10.1039/c6ee01674j.

(78) Chen, K. H.; Wood, K. N.; Kazyak, E.; Lepage, W. S.; Davis, A. L.; Sanchez, A. J.; Dasgupta, N. P. Dead Lithium: Mass Transport Effects on Voltage, Capacity, and Failure of Lithium Metal Anodes. *J. Mater. Chem. A Mater.* **2017**, *5* (23), 11671–11681. https://doi.org/10.1039/C7TA00371D.

(79) Hong, Z.; Viswanathan, V. Phase-Field Simulations of Lithium Dendrite Growth with Open-Source Software. *ACS Energy Lett.* **2018**, *3* (7), 1737–1743. https://doi.org/10.1021/acsenergylett.8B01009.

(80) Bai, P.; Guo, J.; Wang, M.; Kushima, A.; Su, L.; Li, J.; Brushett, F. R.; Bazant, M. Z. Interactions between Lithium Growths and Nanoporous Ceramic Separators. *Joule* **2018**, *2* (11), 2434–2449. https://doi.org/10.1016/j.joule.2018.08.018.

(81) Jana, A.; Woo, S. I.; Vikrant, K. S. N.; García, R. E. Electrochemomechanics of Lithium Dendrite Growth. *Energy Environ. Sci.* **2019**, *12* (12), 3595–3607. https://doi.org/10.1039/C9EE01864F.

(82) Counihan, M. J.; Chavan, K. S.; Barai, P.; Powers, D. J.; Zhang, Y.; Srinivasan, V.; Tepavcevic, S. The Phantom Menace of Dynamic Soft-Shorts in Solid-State Battery Research. *Joule* **2024**, *8* (1), 64–90. https://doi.org/10.1016/j.joule.2023.11.007.

(83) Wood, K. N.; Kazyak, E.; Chadwick, A. F.; Chen, K. H.; Zhang, J. G.; Thornton, K.; Dasgupta, N. P. Dendrites and Pits: Untangling the Complex Behavior of Lithium Metal Anodes through Operando Video Microscopy. *ACS Cent. Sci.* **2016**, *2* (11), 790–801. https://doi.org/10.1021/acscentsci.6b00260.

(84) Lasia, A. Electrochemical Impedance Spectroscopy and Its Applications. *Modern Aspects of Electrochemistry* **2002**, 143–248. https://doi.org/10.1007/0-306-46916-2_2.

(85) Miller, J. R. Introduction to Electrochemical Capacitor Technology. *IEEE Electrical Insulation Magazine* **2010**, *26* (4), 40–47. https://doi.org/10.1109/MEI.2010.5511188.

(86) Gunnarsdóttir, A. B.; Amanchukwu, C. V.; Menkin, S.; Grey, C. P. Noninvasive in Situ NMR Study of "Dead Lithium" Formation and Lithium Corrosion in Full-Cell Lithium Metal Batteries. *J. Am. Chem. Soc.* **2020**, *142* (49), 20814–20827. https://doi.org/10.1021/jacs.0c10258.

(87) Chien, Y. C.; Li, H.; Lampkin, J.; Hall, S.; Garcia-Araez, N.; Brant, W. R.; Brandell, D.; Lacey, M. J. Impact of Compression on the Electrochemical Performance of the Sulfur/Carbon Composite Electrode in Lithium-Sulfur Batteries. *Batter. Supercaps* **2022**, *5* (7), e202200058. https://doi.org/10.1002/BATT.202200058.

(88) https://www.sigmaaldrich.com.

(89) https://www.celgard.com.

(90) https://mtixtl.com.

(91) https://www.chemicalbook.com.

(92) https://fluoryx.com.
